# Electroacupuncture inhibited neuronal apoptosis through PGAM5/FUNDC1-dependent mitophagy after ischemic stroke

**DOI:** 10.1186/s13020-026-01383-3

**Published:** 2026-04-03

**Authors:** Li Zhou, Yicheng Peng, Mingchao Fu, Mei Zhou, Chengcai Zhang, Xichen Yang, Yongdan Cun, Simei Zhang, Na Chen, Rong Ning, Yaju Jin, Zuhong Wang, Hong Xin, Pengyue Zhang

**Affiliations:** 1 Key Laboratory of Acupuncture and Massage for Treatment of Encephalopathy, College of Acupuncture, Tuina and Rehabilitation, Yunnan University of Chinese Medicine, No. 1076 Yuhua Road, Chenggong District, Kunming City, 650500 Yunnan Province China; 2 Zhaotong Municipal Hospital of Traditional Chinese Medicine, Zhaotong, 657000 China; 3 Kunming Municipal Hospital of Traditional Chinese Medicine, No. 25 Dongfeng East Road, Panlong District, Kunming City, 650000 Yunnan Province China; 4 The Hong Kong University of Science and Technology - Guangzhou Campus, Guangzhou, 511453 China; 5 Yuxi Municipal Hospital of Traditional Chinese Medicine, No. 53 Nie’er Road, Hongta District, Yuxi City, 653100 Yunnan Province China

**Keywords:** Electroacupuncture, Ischemic stroke, Autophagy, Mitophagy, PGAM5/FUNDC1 pathway, Apoptosis

## Abstract

Neuronal apoptosis persists throughout ischemic stroke. This leads to massive neuron loss and severely impairs recovery of neurological function. Clinical evidence has confirmed that electroacupuncture (EA) effectively improves neurological function after ischemic stroke; however, the underlying mechanism remains to be fully elucidated. In this study, we found that apoptosis and autophagy were activated after ischemic stroke. EA further upregulated autophagy and inhibited neuronal apoptosis in the ischemic stroke model. Furthermore, EA’s neuroprotective effect was linked to mitophagy activation by upregulating PGAM5 expression, which promoted FUNDC1 dephosphorylation and enhanced the interaction between FUNDC1 and LC3, ultimately activating PGAM5/FUNDC1-dependent mitophagy. Enhanced mitochondrial autophagy reduced ROS production and Cyt c release from damaged mitochondria, thereby inhibiting Caspase3 activation and subsequent neuronal apoptosis. Meanwhile, EA also upregulated the level of FUNDC1 and further promoted mitophagy through the PGAM5/FUNDC1 pathway. Notably, lateral ventricle injection of 3-MA inhibited mitophagy and significantly reversed the neuroprotective effect of EA. In summary, the neuroprotective effect of EA against ischemic stroke might be achieved by inhibiting neuronal apoptosis through PGAM5/FUNDC1-dependent mitophagy.

**Supplementary Information:**

The online version contains supplementary material available at 10.1186/s13020-026-01383-3.

## Introduction

Stroke occurs due to either an interruption of blood flow to the brain or a sudden rupture of blood vessels within the brain, leading to different types of stroke [10]. Stroke can be classified as ischemic or hemorrhagic. Ischemic stroke accounts for 87% of all cases [9]. Restoring blood flow as soon as possible after ischemic stroke is the most effective treatment [39]. However, sudden reperfusion of blood flow may exacerbate brain damage, a phenomenon known as cerebral ischemia reperfusion injury (CIRI) [2]. CIRI affects multiple functions, including motor abilities [29], sensory abilities [7], speech abilities [32], and cognitive abilities [15]. These impairments severely disrupt patients' daily activities and place a heavy burden on their families and society. Recent studies have demonstrated that CIRI causes severe mitochondrial damage [12]. Damaged mitochondria release apoptosis-inducing factors, such as reactive oxygen species (ROS) and cytochrome C (Cyt c) [33], which activate apoptosis and accelerate cell death. Therefore, it is essential to inhibit neuronal apoptosis induced by damaged mitochondrial and provide a favorable intracellular environment for cell survival after ischemic stroke.

Mitophagy is a specific cellular process that degrades excess or partially senescent damaged mitochondria under normal conditions. This process helps maintain the mitochondrial quality control system [1]. Under ischemic and hypoxic conditions, bioenergetic metabolic dysfunction leads to ATP depletion and a surge in calcium ion influx. This leads to the collapse of mitochondrial membrane potential and excessive production of ROS. These changes trigger a series of pathological cascades, including glutamate excitotoxicity, necrotic cell death, oxidative stress, and ER stress [52]. Consequently, the opening of the mitochondrial permeability transition pore is further exacerbated, releasing cytochrome c into the cytoplasm. This release ultimately activates the caspase cascade and initiates programmed cell death [41]. After revascularization, mitochondria generate excessive ROS, which further induce oxidative stress injury [58]. Moreover, excessive ROS production and Ca^2+^ overload can promote pro-inflammatory factor secretion, leading to an inflammatory response in brain tissue [36]. Therefore, activation of mitophagy can prevents the accumulation of damaged mitochondria, protects neurons from oxidative stress injury, inflammation, and apoptosis.

FUN14 domain-containing protein 1 (FUNDC1) is an important receptor protein. It mediates mitophagy specifically under hypoxic conditions [12]. In the physiological state, SRC kinase and casein kinase II (CK2) phosphorylate FUNDC1 at Tyr18 and Ser13 sites, decreasing its affinity for microtubule-associated protein light chain 3 (LC3) and inhibiting mitophagy [21, 50]. After ischemia and hypoxia, CK2 activity decreases. Subsequently, phosphoglycerate mutase 5 (PGAM5) catalyzes the dephosphorylation of FUNDC1 at the Ser13 site [38], enhancing its affinity for LC3 and activating mitophagy. Thus, the dephosphorylation of FUNDC1 catalyzed by PGAM5 may serve as a protective mechanism that facilitates neural repair following ischemic stroke.

Electroacupuncture (EA) is an alternative therapy combining traditional acupuncture with modern electrical stimulation. It was approved by the World Health Organization as early as 1979 for treating neurological disorders such as pain, nausea, and stroke [60]. Several clinical studies have shown that EA can significantly improve neurological dysfunctions caused by ischemic stroke, including motor [47], sensory [25], speech [20, 22], and swallowing [13]. However, the neuroprotective mechanism of EA in ischemic stroke is not fully understood. Huanyuan et al. demonstrated that EA has neuroprotective effects. It promotes selective autophagy in damaged mitochondria [43]. Furthermore, it has been shown that EA regulates autophagy levels differently at different stages of ischemic stroke [16]. Therefore, in this study, we investigated the effects of EA on autophagy and mitophagy in MCAO rats at three time points. We also explored whether EA inhibits neuronal apoptosis induced by CIRI through activating PGAM5/FUNDC1-dependent mitophagy. Our results showed that autophagy peaked on day 6 and gradually declined, similar to a sinusoidal curve. Furthermore, EA upregulated the level of mitophagy, thereby inhibiting Cyt c-induced apoptosis. Finally, our finding demonstrated that the enhanced mitophagy induced by EA was dependent on PGAM5/FUNDC1 pathway.

## Materials and methods

### Animals and grouping

**Fig. 1:**
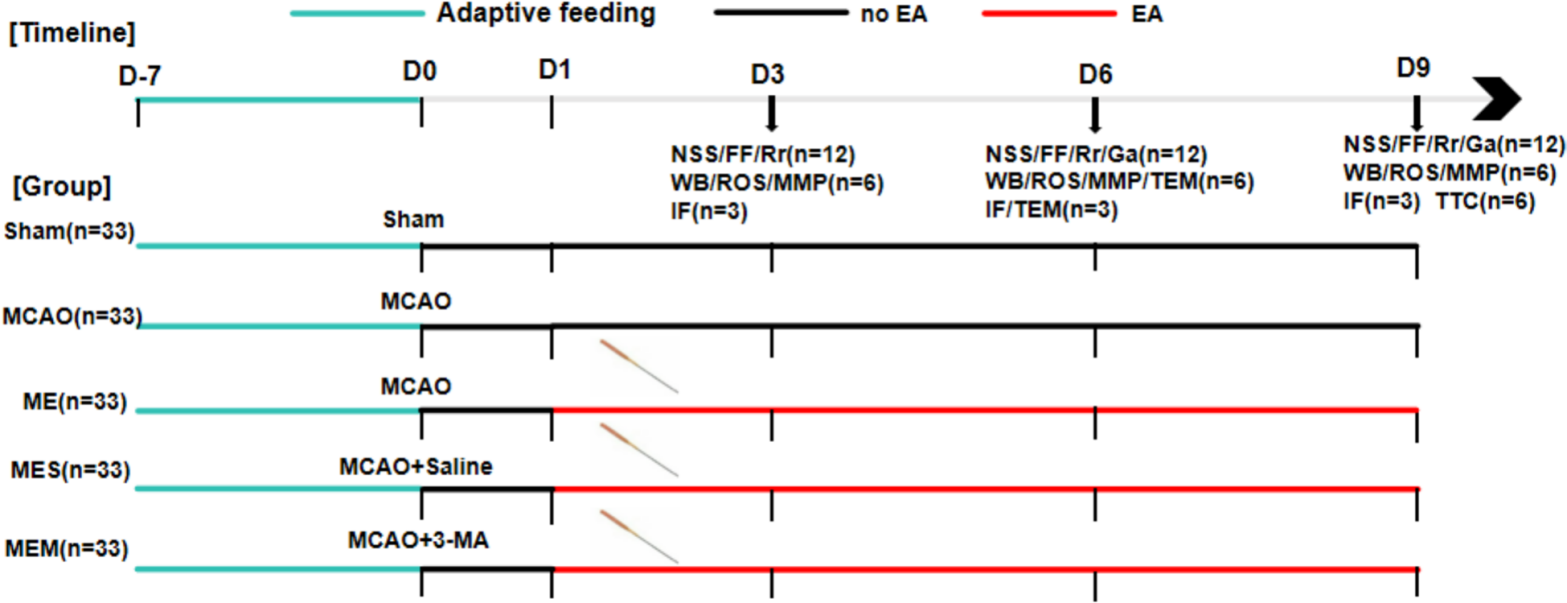
Schematic illustration of the experimental design. Sham, rats with sham operation; MCAO, middle cerebral artery occlusion; EA, electroacupuncture; ME, MCAO + EA; MES, MCAO + EA + Saline; MEM, MCAO + EA + 3-MA; NSS, neurological severity score; FF, foot fault test; Rr, Rota rod test; Ga, Gait analysis; WB, Western blot; ROS, reactive oxygen species; MMP, mitochondrial membrane potential; lF, immunofluorescence; TEM, transmission electron microscopy; n, the number of rats in this test

Adult male Sprague–Dawley rats (purchased from Hunan Chushang Animal Center) were used in this study. 165 rats were randomly divided into five groups: sham-operated group (Sham), MCAO model group (MCAO), EA group (MCAO + EA), autophagy inhibitor group (MCAO + EA + 3-MA), and saline control group (MCAO + EA + Saline). Each group included 33 rats. Among them, 6 rats were used for TTC staining. Eighteen rats were used to detect Western blot, mitochondrial membrane potential (MMP), and ROS at days 3, 6, and 9 after MCAO, with 6 rats assessed at each time point. The remaining 9 rats were used for TUNEL staining and immunofluorescence, with 3 rats evaluated at each time point. To minimize the use of experimental animals, rats used for TTC staining and Western blot on day 9 after MCAO (12 rats per group) were also evaluated for neurobehavioral recovery. Additionally, 3 rats from each group on day 6 were used for transmission electron microscopy (TEM). The experimental design including group and intervention time points for tests in present experiment are depicted in Fig. 1. The Experimental Animal Ethics Committee of Yunnan University of Traditional Chinese Medicine (R-062022LH061) approved the experiments, and all experimental operations on animals were performed in accordance with relevant national regulations.

### Brain stereotactic injection

Lateral ventricular injections were performed according to previously reported methods [28]. After anesthetizing the animals, the incision site was disinfected. An incision was made along the sagittal plane to expose the anterior and posterior fontanelles. The coordinate axes were adjusted to locate the injection point of the lateral ventricle (1.5 mm from the midline on the center-right lateral side, − 0.8 mm from the anterior thorax, and − 3.5 mm dorsal–ventral from the cranial bone). The autophagy inhibitor 3-MA (M9281 Sigma-Aldrich USA) was microscopically injected using a 10-µl Hamilton microsyringe at a rate of 2 µl/min. The 3-MA was administered at a concentration of 200 nmol in a volume of 10 µl [35].

### Model preparation

We used the internationally recognized monofilament method [24] to generate the middle cerebral artery occlusion (MCAO) model [24]. The monofilament (2636-A4, Sinonics, China) completely occluded the origin of the middle cerebral artery for 60 min, after which it was withdrawn to allow reperfusion following ischemia. The ischemic injury was primarily localized to the vascular territory of the middle cerebral artery, namely the striatum and cerebral cortex. The sham surgery group underwent the same surgical steps, except that the middle cerebral artery was not occluded.

### Electroacupuncture treatment

In clinical practice, the frequency of acupoint electroacupuncture treatment is mostly 2 Hz, with some cases at 1 Hz. Continuous wave is used for the upper limbs, while sparse-dense wave is commonly applied to other areas. The treatment duration ranges from 15 to 30 min. Animal experiments, such as rats, often use a 2 Hz, 1 mA sparse-dense wave with treatment durations similar to clinical practice [14, 30, 54]. Therefore, the parameters for present study are set as: 2 Hz, 1 mA, sparse-dense wave, once daily for 30 min. In terms of acupoint selection, “GV20” and “DU14” are commonly used for treatment of ischemic stroke, “LI11” is suitable for qi stagnation and blood stasis, and “PC6” has effects of warming the meridians and calming the mind. Current research indicates that electroacupuncture treatment on these acupoints can promote BDNF expression and increase cerebral blood flow [19, 20, 22, 53], thus these acupoints are selected in present study.

EA treatment started 24 h after reperfusion. Rats were anesthetized with isoflurane (induced under 3–4%, maintained under 1.5–2%) using a gas anesthesia machine (ZS-M, Beijing Zhongshidichuang Technology Development Co., Ltd., China), and then an acupuncture needles (0.35 × 25 mm, Hwatuo, Suzhou Medical Supplies Factory Co., LTD, China) were used to puncture GV20 and DU14 perpendicularly, while LI11 and PC6 on the right limb were punctured obliquely. And then the acupuncture needles were connect to an electric acupuncture apparatus (G6805-II, Shanghai Medical Electronic Equipment, China) with sparse-dense wave, frequency 2 Hz, current 2 mA, for 30 min. The treatment was conducted once daily for continuous 9 days.

### Neurological severity scores (NSS)

A 7-point scoring system was used, and the rat neurological severity scores were blindly assessed at each scheduled time point (Fig. 1), with higher scores indicating more severe neurological deficits.

### Foot-fault scores

Foot-fault scoring was performed at each scheduled time point (Fig. 1). During testing, rats passed the horizontal ladder at a uniform speed and were scored three consecutive times based on their grip. The mean score was used for statistical analysis.

### Rotating rod fatigue test

Before the formal test, mice undergo continuous rod training for 2 days. Each day includes 3 sessions, where the speed accelerates from 4 to 40 revolutions per minute(rpm) over 5 min. A 30-min interval is given between sessions to ensure adequate rest. During the formal test, the rod runs at a constant speed of 30 revolutions per minute for 5 min to assess fatigue. The average duration of sustained movement on the rod across three repeated trials was used for statistical analysis.

### CatWalk gait analysis experiment

CatWalk gait analysis experiments were performed at each detection time point, where rats were placed into the gait channel and allowed to pass through at a constant speed. Measurements were repeated three times, and the average left and right side forelimb support duration was taken for statistical analysis.

### TTC staining

After the rats were deeply anesthetized with 4% isoflurane, 50 ml of saline was perfused transcardially, then the brains were taken and put into molds. Six 2-mm-thick coronal brain slices were cut with a razor blade (RWD Life Science, China), followed by staining with 2% 2,3,5-triphenyl tetrazolium chloride (TTC) staining (T8877 sigma-Aldrich USA). Finally, photographs were taken, and cerebral infarct volumes were calculated with Image-Pro Plus 6.0 image software (Media Cybernetics, Rockville, MD, USA).

### Western blot

The rats have been sacrificed by decapitation under profound anesthesia with 4% isoflurance in accordance with IACUC guidelines at the scheduled testing time point. Then, the brains were severed to isolate the cortex of the ischemic penumbra region of the left middle cerebral artery, which was immediately snap-frozen in dry ice and then transferred to a − 80 ℃ freezer for preservation until assay. Mitochondria were isolated from brain tissue samples using a mitochondrial isolation kit (KGA827 Key GEN BioTECH China), and mitochondrial proteins were extracted. Total protein extraction (High-Efficiency RIPA Lysate R0010-100 ml) and quantification (ZJ102 Yase China) were performed after sample collection. This was followed by SDS-PAGE separation, membrane transfer, blocking (PS112L Yase China), primary antibody incubation, secondary antibody incubation, signal development (BL520B biosharp China), and finally data analysis and statistical evaluation (Raw Data link: [10.57760/sciencedb.15644]).

### Mitochondrial membrane potential assay (MMP)

The rats were euthanized with isoflurane anesthesia. The cerebral cortex of the infarcted penumbra region was isolated. Mitochondria were then extracted using a mitochondrial kit. The extracted mitochondria were mixed with JC-1 staining working solution (C2003S, Beyotime, China). The samples were loaded into three replicate wells. Fluorescence intensity values were then measured using a multifunctional enzyme labeling instrument (Tecan Infinite M200 PRO, Tecan, Switzerland). The average fluorescence values were used for statistical analysis.

### ROS detecting

The rats were anesthetized with isoflurane. The cerebral cortex of the infarcted penumbra area was isolated and immediately rinsed with PBS. The cell suspension was collected using the net rubbing method followed by centrifugation at 500 g. Then, the cell suspension was resuspended with the ROS probe (E004-1-1, Nanjing Jianjian, China). Finally, fluorescence intensity was detected using a multifunctional enzyme labeling instrument.

### Immunofluorescence (IF) co-localization

Rats were deeply anesthetized with 4% isoflurance and transcardially perfused with physiological saline and 4% paraformaldehyde. And then the brain was harvested and underwent sucrose gradient dehydration. Then, tissues were embedded in OCT compound to prepare frozen sections 10–30 μm thick. During the experiment, antigen retrieval solution (G1202, Servicebio, China) was applied, followed by blocking non-specific binding sites with bovine serum albumin (BSA). We incubated the samples separately with LC3 and TOMM20 specific primary antibodies, then added fluorescently labeled secondary antibodies. After the cell nuclei were stained with DAPI, the slides were mounted with anti-fluorescence quenching mounting medium. Finally, the samples were observed and imaged under a fluorescence microscope. The co-localization of LC3 and TOMM20 was quantified using Image-Pro Plus 6.0 software.

### Transmission electron microscopy (TEM) of mitochondria and autophagic vesicles

Rats were euthanized under deep anesthesia with isoflurane. Approximately 1 mm^3^ of tissue was taken from the ischemic penumbra of the frontal and parietal cortex. The tissue was fixed with 1% osmium tetroxide, dehydrated using an ethanol-acetone gradient, embedded in epoxy resin, and then ultrathin sections were prepared (Leica UC7, Germany). After double staining with uranyl acetate and lead citrate, the number of autophagosomes and autolysosomes, as well as mitochondrial morphology, were observed under a transmission electron microscope (HT7700, Hitachi, Japan). During quantitative analysis, three rats were included for each group, and three non-overlapping fields were randomly selected for imaging in each rat. The experimenters counted autophagosomes and autolysosomes while blinded to group allocation (single-blind). Statistical analysis was based on the average counts from multiple visual fields and cells in each sample.

### TUNEL staining

The procedure included sucrose dehydration, OCT embedding, sectioning (10–30 µm), and TUNEL staining. The staining process consisted of fixation, proteinase K treatment, membrane disruption, equilibration, incubation with reaction solution, BSA blocking, antibody incubation, followed by DAPI re-staining and sealing. Sections were observed under a fluorescence microscope to capture images. The neuronal apoptosis rate was then calculated using Image-Pro Plus 6.0 software.

### Statistical analysis

SPSS software was used to analyze the data, and GraphPad Prism 9.0 for graphing. Data are shown as mean ± standard error of the mean (SEM). For time series data, one-way repeated measures ANOVA is used, followed by Bonferroni correction for post-hoc comparisons if significant. Forelimb support lengths were compared using a paired *t*-test. The statistics of TTC staining and autophagosome counts are analyzed using one-way ANOVA, with significant results followed by Tukey HSD test for post hoc comparisons. A *p* value of <0.05 was considered statistically significant.

## Results

### EA inhibits apoptosis in the ischemic penumbra to attenuate neurological deficits after MCAO

**Fig. 2:**
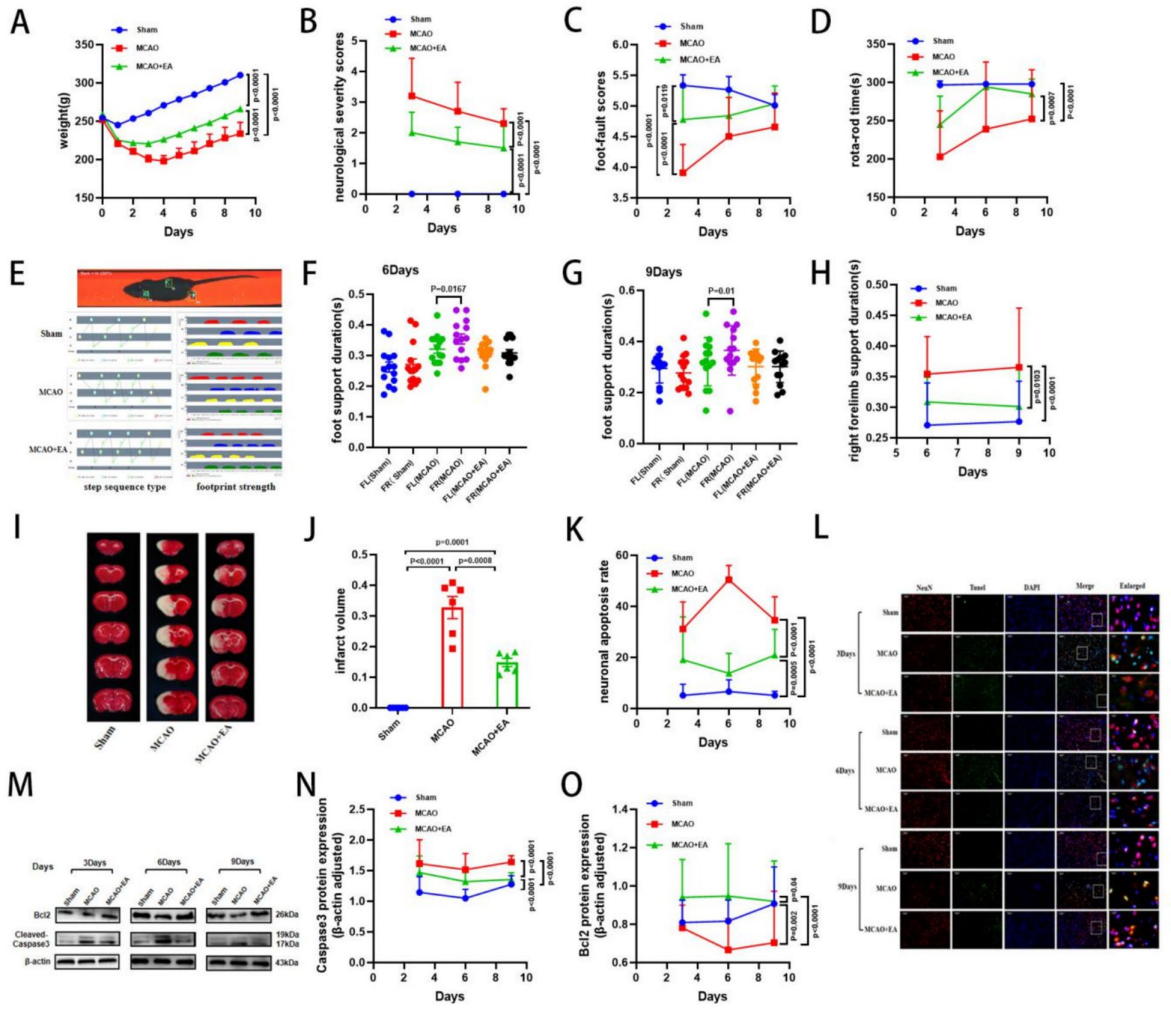
EA inhibits apoptosis to promote improvement in neurological function. **A–D** Weight change after MCAO and the neurobehavioral tests of NSS score, Foot-fault score and Rota-rod Test. **e **Step sequence type and footprint intensity map, and the quantification of right and left lateral forelimb support duration results on day 6, 9 (**F, G**) and right forelimb support duration outcome (**H**). **I, J** Representative images of TTC staining brain slices and quantification of cerebral infarct volume. **M–O** Representative Western blot images of Bcl2 and cleaved caspase3 and the quantitative results on day 3, 6 and 9. **K, L** Neuronal apoptosis rate and representative images of TUNEL staining (Scale bar: 50 μm). (*n* = 12 per group for neurobehavioral tests, *n* = 6 per group for TTC staining and West blot, *n* = 3 per group for TUNEL staining, *P < 0.05, **P < 0.01, ***P < 0.001, ****P < 0.0001)

We established the MCAO model in SD rats using the thread bolus method to investigate the neuroprotective effect of EA. We also examined whether this effect was related to apoptosis. Next, we recorded the body weights of the rats and found a continuous loss of body weight in the first 4 days (Fig. 2A). Subsequently, Neurobehavioral assessments, TTC staining, and the expression of Caspase3 and B cell lymphoma/leukemia-2 protein (Bcl2) in ischemic brain tissues were evaluated on days 3, 6, and 9 after MCAO. Neurobehavioral results showed that MCAO increased neurological severity scores, decreased Foot-fault scores, and shortened Rota-rod-time (Fig. 2B–D) duration in SD rats. Gait analysis experiments showed that MCAO prolonged the support time of the forelimb on the hemiplegic side and increased the gap in support duration between bilateral forelimbs (Fig. 2E–H). TTC staining results (Fig. 2I, J) showed a significant increase in cerebral infarct volume in MCAO rats. Furthermore, the expression of caspase3 was increased and the expression of Bcl2 was decreased in the MCAO group compared with the Sham group (Fig. 2M–O). TUNEL staining results (Fig. 2K, L) also showed that MCAO resulted in an elevated rate of neuronal apoptosis. These results indicate that apoptosis is activated after MCAO, leading to severe impairment of neurological function in SD rats. Therefore, in the following experiments, we evaluated the effects of EA on neural function and apoptosis in the penumbra region of rats. The results showed that EA slowed weight loss in MCAO rats. Meanwhile, it decreased the neurological severity scores, elevated the Foot-Fault scores (Fig. 2B–D), and prolonged the duration of the Rota-Rod-Test in MCAO rats. It also shortened the forelimb support time on the hemiplegic side, closed the gap in support time between the bilateral forelimbs, and gradually corrected gait abnormalities (Fig. 2E–H). TTC staining (Fig. 2I, J) showed that EA significantly reduced the volume of cerebral infarction in MCAO rats. Western blot results showed that EA downregulated the expression of Caspase3 and upregulated the expression of Bcl2 in rats compared with the MCAO group (Fig. 2M–O). Meanwhile, it also significantly reduced neuronal apoptosis in MCAO rats. These data confirmed that EA at GV20, DU14, LI11, and PC6 can effectively inhibit apoptogenesis and attenuate neurological deficits after ischemic stroke.

### EA upregulates the level of autophagy in the ischemic penumbra of MCAO rats

**Fig. 3:**
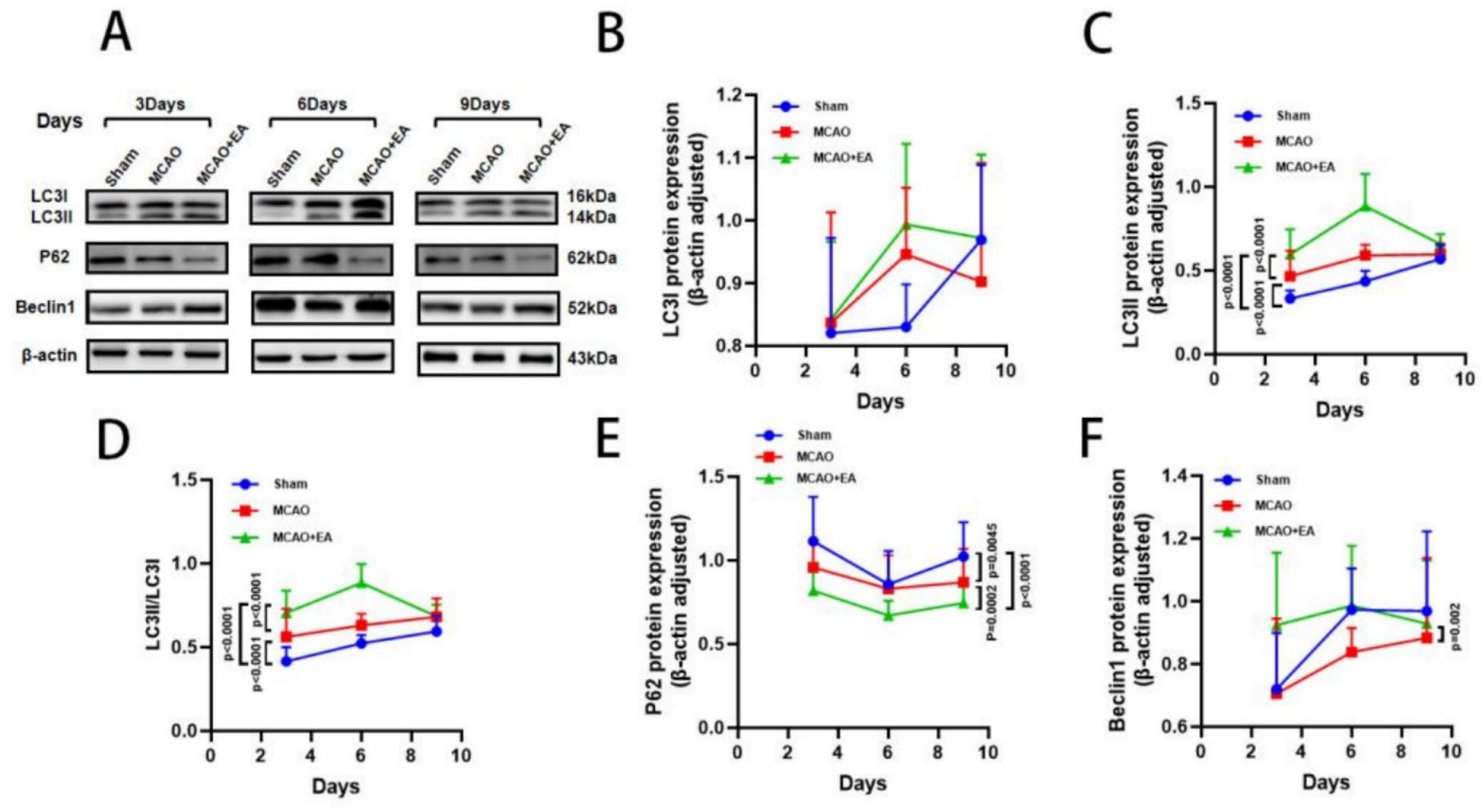
EA upregulates the level of autophagy in the ischemic penumbra of MCAO rats. **A** Representative Western blot images of LC3, P62, Beclin1 and **B–F** their quantitative results on day 3, 6 and 9. (*n* = 6 per group for West blot, *P < 0.05, **P < 0.01, ***P < 0.001, ****P < 0.0001)

To verify whether EA inhibition of apoptosis is related to autophagy, we detected LC3, P62, and Beclin1 expression in brain tissues of the penumbra area using Western blot (Fig. 3A). The results showed that autophagy levels increased, peaked on day 6, and then gradually decreased on day 9. LC3I and LC3II expression were significantly upregulated on day 6, while only LC3II expression was significantly increased on days 3 and 9 (Fig. 3B–D). Furthermore, P62 expression was significantly downregulated in rats of the MCAO group compared with the Sham group, while EA treatment further downregulated P62 expression (Fig. 3E). Similarly, Beclin1 was downregulated in the MCAO group on day 6 compared with the Sham group, while EA treatment upregulated Beclin1 expression (Fig. 3F). These results suggest that EA activates autophagy via different molecular mechanisms at various stages of ischemic stroke.

### EA enhances mitophagy in the ischemic penumbra of MCAO rats

**Fig. 4:**
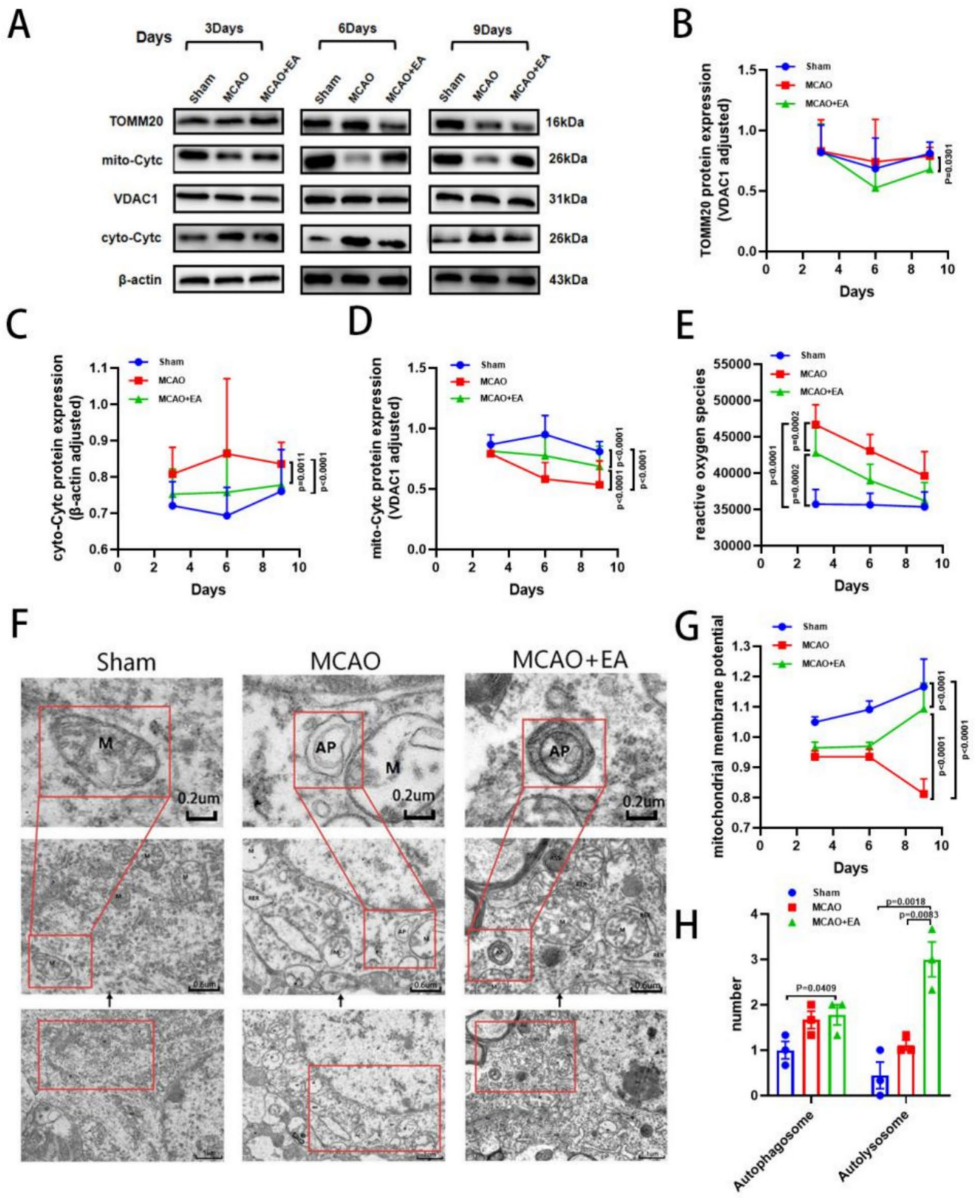
EA enhances mitophagy in the ischemic penumbra of MCAO rats. **A** Representative Western blot images of TOMM20, mito-Cyt c, cyto-Cyt c and** B–D** their quantification results on day 3, 6 and 9. **E** ROS content. **G** Quantification of mitochondrial membrane potential. **F, H** Representative TEM images in the cerebral cortex in the penumbra region and the quantitative analysis of autophagic vesicles and autophagic lysosomes. (*n* = 6 per group for West blot, MMP and ROS content detection, *n* = 3 per group for TEM, *P < 0.05, **P < 0.01, ***P < 0.001, ****P < 0.0001)

To further verify whether EA inhibits apoptosis by activating mitophagy, we examined the expression of the mitophagy marker protein TOMM20. Additionally, we assessed mitochondrial structural functions. Western blot results showed that compared with the MCAO group, except for day 3, EA significantly downregulated Translocase of outer mitochondrial membrane 20 (TOMM20) expression. MCAO also downregulated mitochondrial cytochrome C (mito-Cyt c) expression and upregulated cytoplasmic cytochrome C (cyto-Cyt c) expression (Fig. 4A–D). Conversely, EA treatment significantly reversed the changes in mito-Cyt c and cyto-Cyt c expression caused by MCAO. Furthermore, ROS and mitochondrial membrane potential (MMP) assays demonstrated that EA reduced the extent of MMP decline and decreased the excessive release of ROS compared with the MCAO group (Fig. 4E, G). Finally, transmission electron microscopy results showed that MCAO caused severe mitochondrial swelling, massive loss of cristae, and vacuolization in the penumbra region. In the EA group, mitochondrial swelling and cristae breakage were significantly reduced. Additionally, the number of autophagic lysosomes increased, and autophagic vesicles engulfing damaged mitochondria were observed (Fig. 4F, H). These results confirm that EA activates mitophagy to inhibit the Cyt c/Caspase3 pathway apoptosis.

### EA activates PGAM5/FUNDC1-dependent mitophagy signaling pathway

**Fig. 5:**
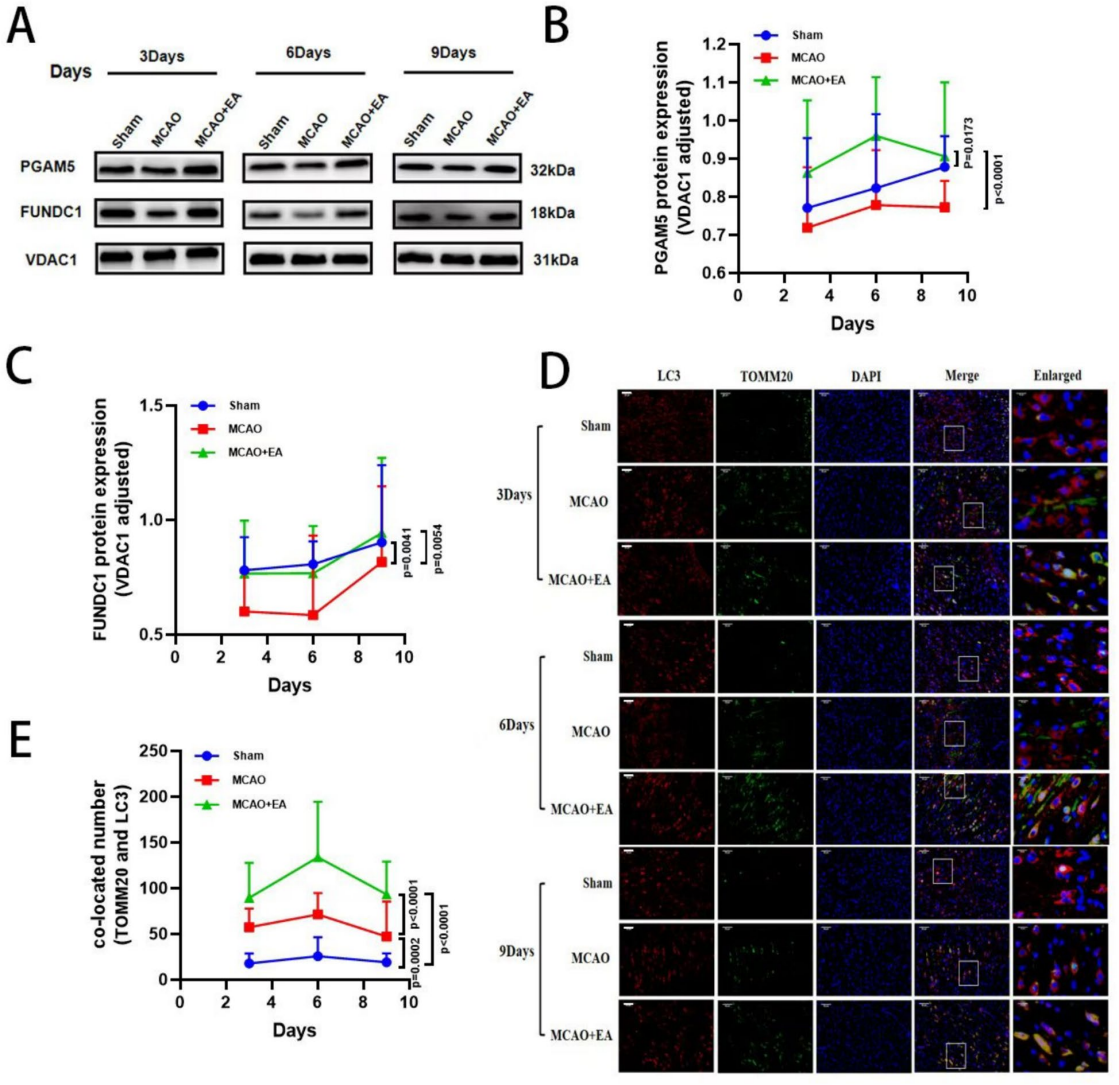
EA activates the PGAM5/FUNDC1 signaling pathway. **A** Representative Western blot images of PGAM5, FUNDC1 and **B, C** their quantification results on day 3, 6 and 9. **D, E** Representative co-localization of TOMM20 and LC3 and their quantitative results (Scale bar: 50 μm). (*n* = 6 per group for West blot, *n* = 3 per group for IF, *P < 0.05, **P < 0.01, ***P < 0.001, ****P < 0.0001)

FUNDC1 is a vital receptor protein for mediated mitophagy [40]. It has been shown that hypoxia induces down-regulation of the mRNA level of FUNDC1 [56]. Therefore, we aimed to verify whether EA inhibits apoptosis by activating mitophagy through the PGAM5/FUNDC1 pathway. Western blot analysis revealed that MCAO downregulated PGAM5 and FUNDC1 expression (Fig. 5A–C). Conversely, EA upregulated PGAM5 and FUNDC1 expression. In addition, the results of immunofluorescence co-localization showed that EA significantly increased the number of TOMM20 co-localized with LC3 (Fig. 5D, E). These results confirmed that EA upregulated PGAM5, which subsequently promoted FUNDC1 dephosphorylation and activated mitophagy via the PGAM5/FUNDC1 pathway. At the same time, EA upregulated FUNDC1 expression to provide a substrate for the PGAM5 dephosphorylation process. However, on day 9, EA upregulated only PGAM5 expression without affecting FUNDC1 levels, suggesting that mitophagy activation at this stage mainly depended on PGAM5 upregulation and FUNDC1 dephosphorylation.

### 3-MA reverses the upregulation of mitophagy in the ischemic penumbra of MCAO rats by EA

**Fig. 6:**
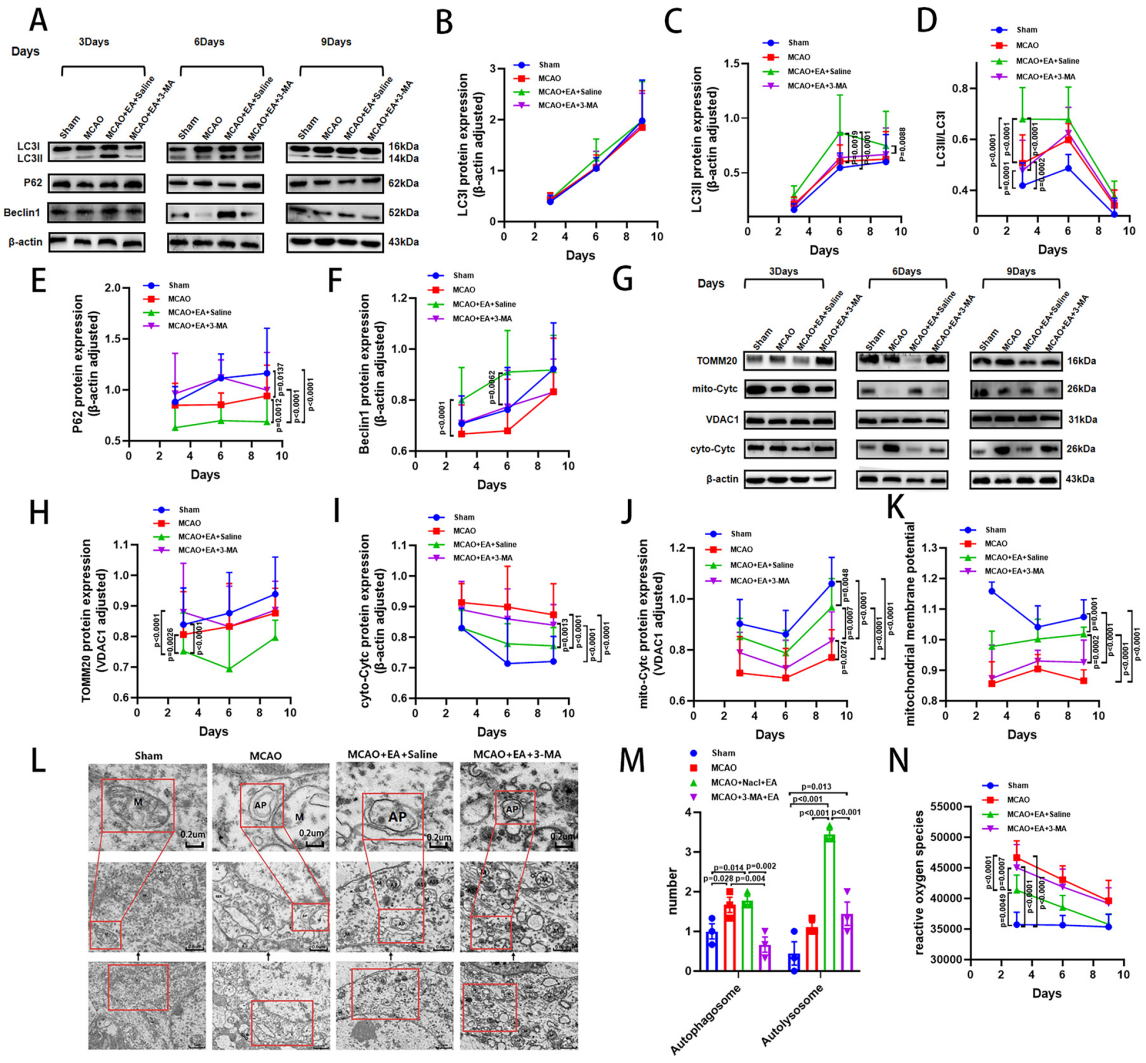
3-MA reverses the activation of mitophagy induced by EA. **A** Representative Western blot images of LC3, P62, Beclin1, **G** TOMM20, mito-Cyt c, and cyto-Cyt c and **B–F, H–J** their quantification results on day 3, 6 and 9. **N** ROS content. **K** Quantification of mitochondrial membrane potential. **L, M** Representative TEM images in the cerebral cortex in the penumbra region and the quantitative analysis of autophagic vesicles and autophagic lysosomes. (*n* = 6 per group for West blot, MMP and ROS content detection, *n* = 3 per group for TEM, *P < 0.05, **P < 0.01, ***P < 0.001, ****P < 0.0001)

Western blot results showed that 3-MA downregulated the expression of LC3II and Beclin1, and the LC3II/LC3I ratio was decreased, while P62 expression was upregulated in the MCAO + EA + 3-MA group compared with the MCAO + EA + Saline group. Interestingly, we found that the effect of 3-MA significantly weakened on day 9 and did not reverse the changes in expression of LC3II, Beclin1, or the LC3II/LC3I ratio (Fig. 6A–F). Transmission electron microscopy revealed more severe mitochondrial swelling and vacuolization in the MCAO + EA + 3-MA group compared to the MCAO + EA + Saline group. Furthermore, the number of autophagosomes and autolysosomes was significantly reduced in the MCAO + EA + 3-MA group (Fig. 6L, M). The expression of TOMM20 was upregulated, mito-Cyt c downregulated, and cyto-Cyt c upregulated in the MCAO + EA + 3-MA group compared to the MCAO + EA + Saline group (Fig. 6G–J). Furthermore, mitochondrial membrane potential decreased (Fig. 6K), and ROS content increased (Fig. 6N) in the MCAO + EA + 3-MA group compared with the MCAO + EA + Saline group. These results indicate that 3-MA can reverse EA-induced activation of mitophagy and suppression of apoptosis.

### 3-MA reverses the activation of PGAM5/FUNDC1-dependent mitophagy by of EA

**Fig. 7.**
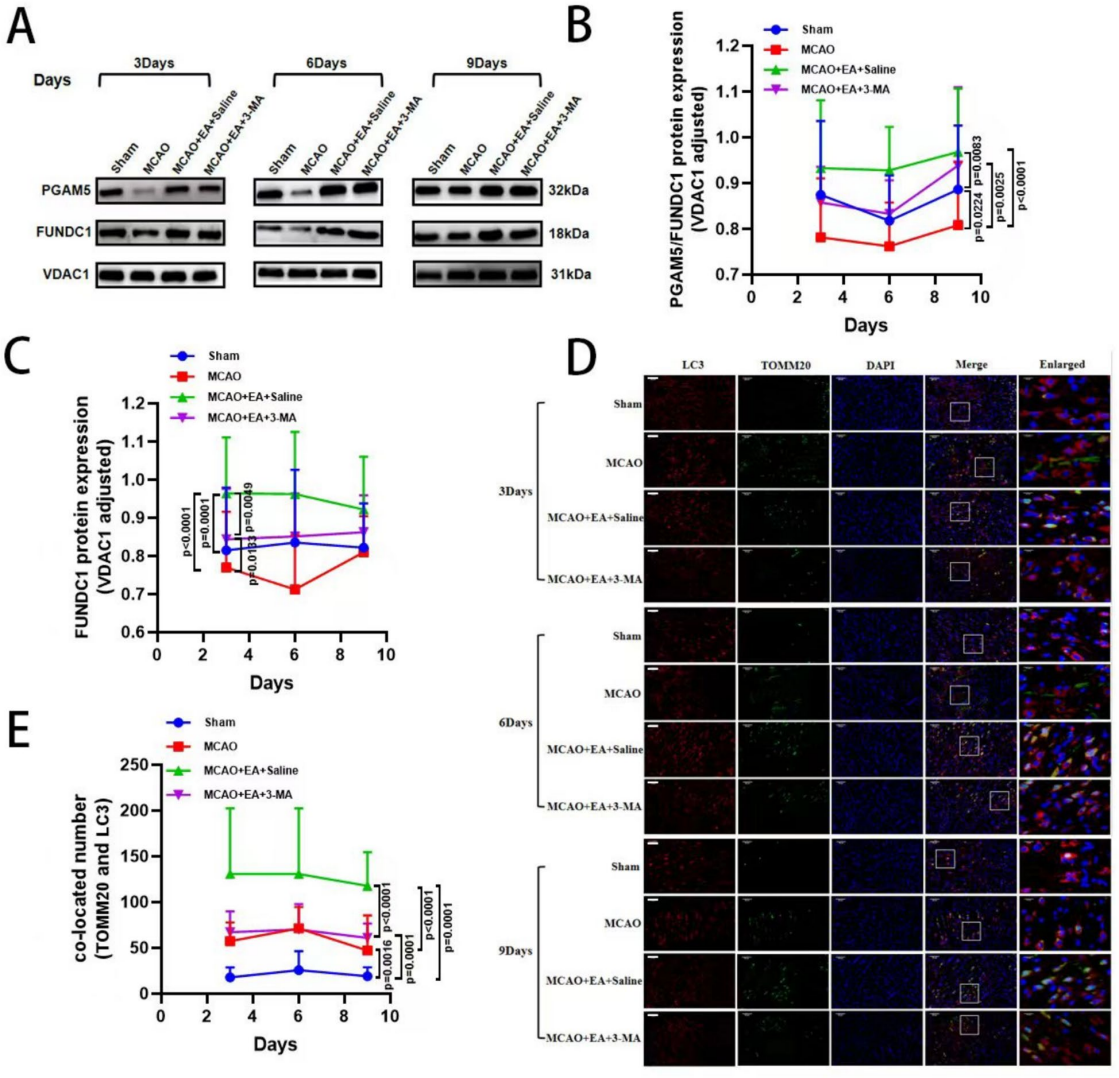
3-MA reverses the activation of PGAM5/FUNDC1 signaling pathway by EA. **A** Representative Western blot images of PGAM5, FUNDC1 and **B, C** their quantification results on day 3, 6 and 9. **D, E** Representative co-localization of TOMM20 and LC3 and their quantitative results (Scale bar: 50 μm). (*n* = 6 per group for West blot, *n* = 3 per group for IF, *P < 0.05, **P < 0.01, ***P < 0.001, ****P < 0.0001)

Further results of Western blot showed that PGAM5 and FUNDC1 expression was significantly increased in the MCAO + EA + Saline group compared to the MCAO group (Fig. 7A–C). PGAM5 and FUNDC1 expression was significantly decreased in the MCAO + EA + 3-MA group compared with the MCAO + EA + Saline group. In addition, immunofluorescence co-localization showed that the number of LC3 puncta co-localized with TOMM20 was significantly reduced in the MCAO + EA + 3-MA group compared with the MCAO + EA + Saline group (Fig. 7D, E). These results confirmed that the activation of the PGAM5/FUNDC1 signaling pathway by EA, which exerts its neuroprotective effect, could be reversed by 3-MA.

### 3-MA reverses the inhibitory effect of EA on apoptosis and its neuroprotective effect

**Fig. 8.**
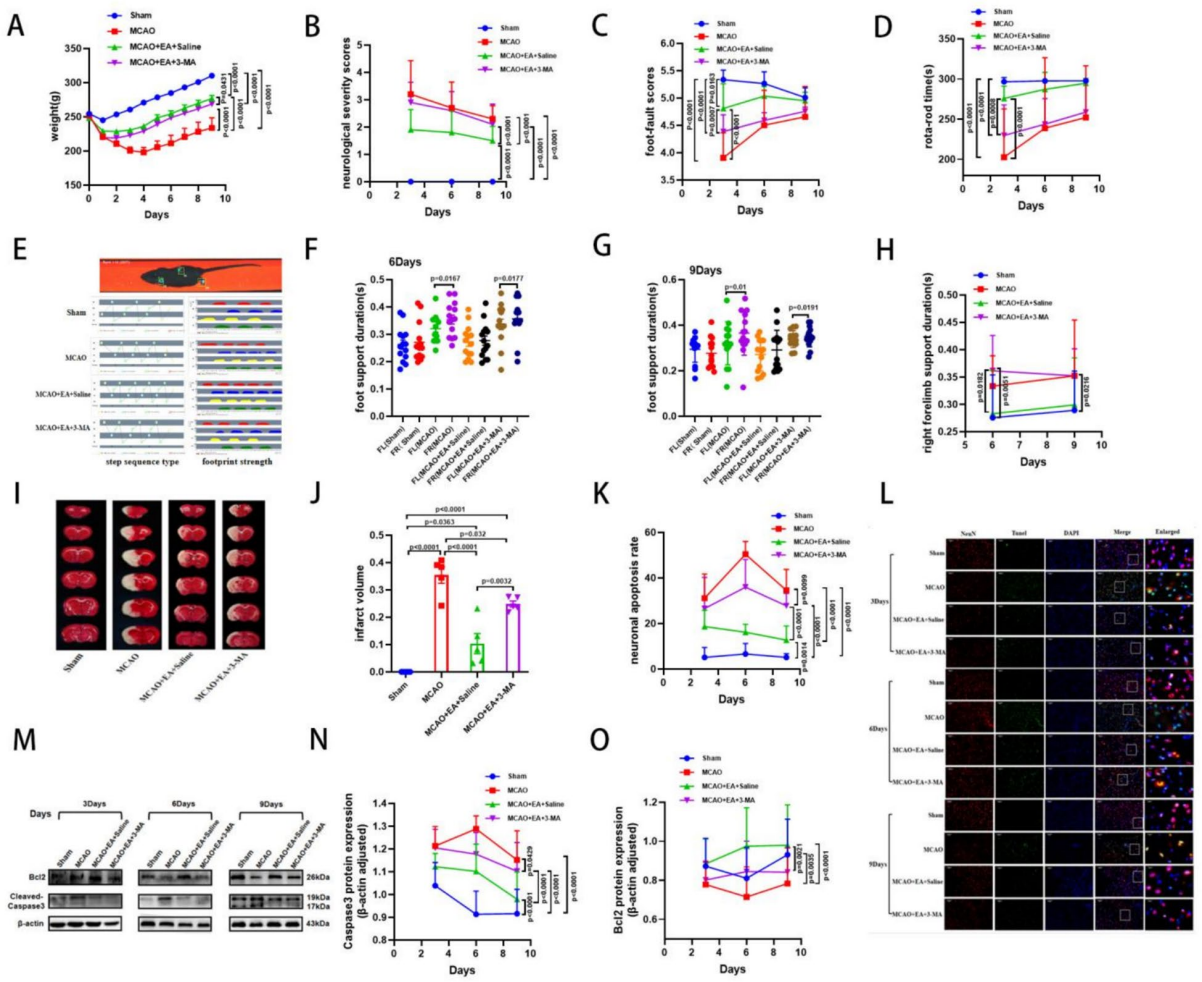
3-MA reverses the neuroprotective effects of EA. **A–D** Weight change after MCAO and the neurobehavioral tests of NSS score, Foot-fault score and Rota-rod Test. **E** Step sequence type and footprint intensity map, and **F, G** the quantification of right and left lateral forelimb support duration results on day 6, 9 and right forelimb support duration outcome (**H**). **I, J** Representative images of TTC staining brain slices and quantification of cerebral infarct volume. **M–O** Representative Western blot images of Bcl2 and cleaved caspase3 and the quantitative results on day 3, 6 and 9. **K, L** Neuronal apoptosis rate and representative images of TUNEL staining (Scale bar: 50 μm). (*n* = 12 per group for neurobehavioral tests, *n* = 6 per group for West blot, *n* = 3 per group for TUNEL, *P < 0.05, **P < 0.01, ***P < 0.001, ****P < 0.0001)

To further investigate the relationship between EA’s inhibition of neuronal apoptosis and the PGAM5/FUNDC1 signaling pathway, we injected the mitophagy inhibitor 3-MA (3-methyladenine) into the lateral ventricle. Western blot (Fig. 8M–O) and TUNEL (Fig. 8K, L) results showed that, compared to the MCAO group, the MCAO + EA + Saline group had increased Bcl2 expression, decreased Caspase3 expression, and reduced neuronal apoptosis. Compared with the MCAO + EA + Saline group, the MCAO + EA + 3-MA group showed downregulated Bcl2 expression and upregulated Caspase3 expression. Additionally, the apoptosis rate was increased in this group. Similarly, we evaluated whether 3-MA attenuated the neuroprotective effects of EA. The results showed that rats in the MCAO + EA + 3-MA group lost body weight more rapidly than those in the MCAO + EA + Saline group (Fig. 8A). The MCAO + EA + 3-MA group showed higher neurologic deficit scores, lower Foot-fault scores, and shorter Rota-rod time durations (Fig. 8B–D). Furthermore, gait analysis showed that the length of limb support on the hemiplegic side was prolonged in the MCAO + EA + 3-MA group compared to the MCAO + EA + Saline group. This prolongation led to an increased support phase gap between the bilateral forelimbs (Fig. 8E–H). Finally, TTC staining revealed a larger cerebral infarction volume in the MCAO + EA + 3-MA group compared to the MCAO + EA + Saline group (Fig. 8I, J). These results confirm that the ability of EA to inhibit apoptosis and improve neurological dysfunction after ischemic stroke is closely related to autophagy.

## Discussion

**Fig. 9:**
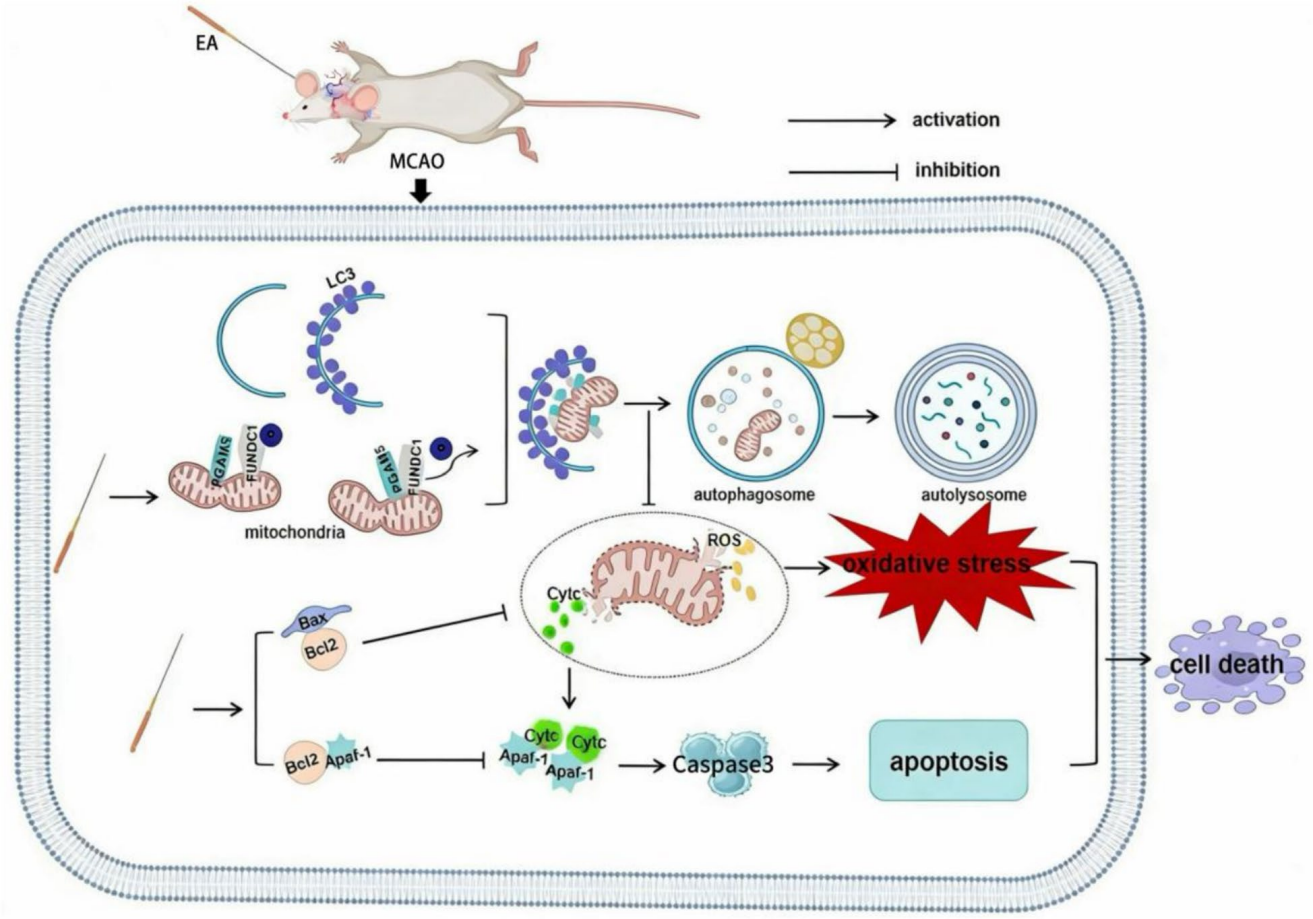
Schematic diagram of the molecular signal regulation mechanism of electroacupuncture to promote neurological function improvement after ischemic stroke. EA, electroacupuncture. MCAO, middle cerebral artery occlusion. ROS, reactive oxygen species; Cytc, cytochrome c; arrow, activation; solid bar, inhibition

Our results confirmed that EA on GV20, DU14, LI11, and PC6 effectively alleviate neurological dysfunction after ischemic stroke. Mechanistically (Fig. 9), the neuroprotective effect of EA is mainly dependent on the activation of mitophagy in the PGAM5/FUNDC1 pathway, which inhibits the release of Cyt c from damaged mitochondria into the cytosol, thereby inhibiting apoptosis in the Cyt c/Caspase3 pathway. Our finding indicated that EA on GV20, DU14, LI11, and PC6 are promising therapies for treatment after ischemic stroke.

In present study, we observe that the PGAM5/FUNDC1 signaling axis play a critical roles during EA-induced mitophagy and neuronal apoptosis inhibition. Our work confirms that electroacupuncture upregulates the expression of both PGAM5 and FUNDC1. More importantly, it reveals their synergistic function: PGAM5, as a phosphatase, may activate FUNDC1 through dephosphorylation, thereby initiating FUNDC1-dependent mitophagy. This "dual regulation" mechanism targeting the upstream key regulatory molecule (PGAM5) and its effector (FUNDC1) provides in-depth and specificity for understanding the neuroprotective effects of electroacupuncture. Recent studies have also emphasized that precise regulation of specific mitophagy pathways (such as FUNDC1) is a new strategy for treating ischemic brain injury [4], our findings are highly consistent with this direction and pinpointed the mechanism of electroacupuncture to this target. In summary, this study goes beyond merely observing associations by revealing the specific signaling pathways through which electroacupuncture exerts its effects. It provides a solid theoretical basis and new therapeutic targets for clinical application.

The autophagy-lysosome system acts as a double-edged sword in ischemic stroke [5, 6, 62]. Nucleation, elongation, and maturation of autophagosomes play an essential role in post-stroke neurorepair [57, 59]. In general, activation of autophagy for removing damaged organelles or proteins in the acute phase of ischemic stroke contributes to cell survival [49, 51]. However, excessive autophagy leads to degradation of healthy organelles and accelerates neuronal cell death [37]. Studies have shown that EA may exert its effects on ischemic stroke through the autophagy-lysosomal pathway [5, 6, 42]. Thus, autophagy may be a potential therapeutic target for neuroprotection after ischemic stroke.

Mitochondria are the energy centers of cells and key regulators of apoptosis. They play a crucial role in the survival of neurons after stroke. Maintaining their functional integrity is essential. Mitophagy, the process of selectively eliminating damaged mitochondria, is a core mechanism for maintaining mitochondrial quality. Increasing evidence emphasizes the critical role of mitophagy in the pathological progression and functional recovery of ischemic stroke [44]. Our findings align with and extend the work in this field, indicating that EA provides neuroprotection by enhancing mitochondrial function and promoting the clearance of damaged mitochondria. Previous pioneering studies identified that EA mainly benefits by activating two mitophagy pathways: the PINK1/Parkin-dependent pathway and the BNIP3-mediated pathway [34, 48]. For example, Wang et al. showed that EA alleviates oxidative stress and mitochondrial damage by upregulating PINK1/Parkin-mediated mitophagy [43]. While Guan et al. demonstrated that EA inhibited apoptosis at the GV20 and GB7 acupoints by activating BNIP3-dependent mitophagy, an effect that could be reversed by the autophagy inhibitor 3-MA [11].

This study highlights the complex neuroprotective mechanisms that EA triggers. Our results suggest that the PGAM5/FUNDC1 signaling axis plays a key role in EA-regulated mitophagy. As previously described [44], our findings support the point that EA effects involve a dual regulatory mechanism. This axis serves as a mitophagy initiation pathway, which is especially important under anoxic conditions. However, it remains insufficiently explored [8]. Our finding further show that EA treatment significantly enhances the interaction between PGAM5 and FUNDC1. This likely involves dephosphorylation and activation of FUNDC1, which promotes the clearance of dysfunctional mitochondria.

In our study, EA significantly upregulated PGAM5 expression, which promotes FUNDC1 dephosphorylation. Meanwhile, EA also upregulated FUNDC1 expression, continuously providing substrates for this process. It should be noted that the upregulated FUNDC1 refers to the total protein level, including both dephosphorylated and phosphorylated forms. Since the anti-FUNDC1 (Ser13) antibody is not yet commercially available, it remains unclear whether EA upregulates phosphorylated FUNDC1 expression. It is the shortcoming of this experiment. However, on day 9, EA significantly upregulated PGAM5 expression, while FUNDC1 upregulation was not significant. This suggests that PGAM5-driven dephosphorylation of FUNDC1 does not substantially contribute to the increase in total FUNDC1 levels. Thus, EA likely upregulates both phosphorylated and dephosphorylated FUNDC1, rather than solely promoting PGAM5-mediated dephosphorylation of FUNDC1.

Furthermore, TOMM20 is a marker protein for mitophagy. It is inversely correlated with the degree of mitophagy [3]. Specifically, lower TOMM20 expression is associated with higher levels of mitophagy [55]. In our results, EA significantly upregulated PGAM5 and FUNDC1 expression for three days, but TOMM20 expression was not significantly decreased. This phenomenon may result from mitochondrial quality control homeostasis, where mitochondrial biogenesis and mitophagy are balanced at this stage. Therefore, TOMM20 expression was not significantly downregulated. In conclusion, EA upregulated PGAM5 and FUNDC1 expression and activated PGAM5/FUNDC1-dependent mitophagy. It also reduced excessive release of ROS and Cyt c from damaged mitochondria, thereby inhibiting apoptosis via the Cyt c/Caspase3 pathway. These effects contributed to improved neurological function after ischemic stroke.

In conclusion, our study not only confirmed the role of mitophagy in EA-mediated neuroprotection but also revealed the key role of the PGAM5/FUNDC1 signaling pathway. This provided a new perspective on the mechanism of EA in treatment for ischemic stroke. However, both EA parameters—including frequency, waveform, current intensity, voltage intensity, duration, acupoints, and start time—and the duration of cerebral ischemia affect the regulation of autophagy levels [23, 61]. For instance, Liu et al. used a rat model of cerebral ischemia–reperfusion injury and showed that three consecutive days of EA treatment significantly downregulated Atg13 and upregulated mTORC1 expression, which protected neuronal cells from CIRI [23]. Using a rat MCAO model, Mei et al. found that continuous EA administered for three days after a 2-h ischemic event significantly reduced cerebral infarction volume and protected neurons from CIRI [26]. In contrast, thr group from Wang [46] reported that EA treatment significantly upregulated the expression of the pro-autophagy protein Beclin1 as early as 2 h after reperfusion in a rodent model of ischemia–reperfusion. In our study, we standardized the EA parameters and the duration of cerebral ischemia to compare the effects of EA on autophagy levels at different treatment times. The results were consistent with those of Wang et al. We found that autophagy levels were upregulated after 24 h of cerebral ischemia and reperfusion in MCAO rats [46]. In contrast, EA further upregulated the expression of LC3 and Beclin1, downregulated the expression of P62, and increased autophagic flux. In addition, our study also showed that autophagy levels peaked on day 6 of EA, with LC3I, LC3II, and Beclin1 expression upregulated. Compared with day 6, autophagy levels slightly decreased on days 3 and 9, with LC3II and Beclin1 expressions upregulated on day three and only LC3II expressions upregulated on day 9. These findings suggest that EA regulates autophagy levels through different mechanisms at various stages. This variation may contribute to the sinusoidal pattern observed in autophagy levels.

Additionally, the inference of autophagic activity in this study mainly relies on changes in steady-state levels of LC3-II and p62 proteins. As emphasized by authoritative guidelines and classic studies in the field, the isolated changes in these two markers may stem from enhanced formation of autophagosomes (upstream of autophagic flux) or may result from the blockage of autophagosome-lysosome fusion or degradation of their contents (downstream of autophagic flux) [17, 27]. Therefore, our data more accurately suggest alterations in the autophagic process rather than directly proving increased autophagic flux. This methodological limitation is a common issue in many current autophagy studies based on marker detection. To better define the autophagic response, future research should use dynamic autophagic flux measurement methods. For example, detection under conditions with or without lysosomal inhibitors such as Bafilomycin A1 can clearly distinguish between autophagy induction and degradation inhibition [17]. This will be a crucial step in confirming the exact mechanisms underlying the observations in this study.

In the present study, we observed that EA intervention upregulated PGAM5, FUNDC1, and Bcl2 expression, while downregulated TOMM20, Caspase3, and Cyt c levels. Moreover, EA increased the colocalization of LC3 and TOMM20 and reduced neuronal apoptosis, which improved neurological function. To verify whether EA protects by activating PGAM5/FUNDC1-dependent mitochondrial autophagy, we used the commonly applied autophagy regulator 3-MA. Researchers often use 3-MA as an autophagy inhibitor in studies. It reduces the LC3II/LC3I ratio and counteracts the molecular and functional benefits of EA. Nevertheless, these results should be interpreted with caution. 3-MA affects not only autophagy but also other signaling pathways like PI3K, making its effects nonspecific to mitochondrial autophagy [45]. Our data suggest EA’s protective effect involves PGAM5/FUNDC1-dependent mitochondrial autophagy, with 3-MA effects offering indirect support. This viewpoint is consistent with previous studies. For example, Guan et al. [11] reported that EA could improve neurological function in rats with cerebral ischemia by activating BNIP3-mediated mitochondrial autophagy, and this effect could be eliminated by 3-MA, similarly suggesting the possible involvement of the autophagy pathway. Moreover, our results showed that 3-MA did not reverse autophagy marker expression on day 9, suggesting its effects may be limited by its half-life, which could impact long-term intervention. Further research is needed to clarify its pharmacokinetic characteristics to determine the duration of action. In summary, the present study suggests that EA may inhibit apoptosis and improve neurological function after stroke by regulating PGAM5/FUNDC1-dependent mitochondrial autophagy, while the experimental results of 3-MA provide indirect clues for this mechanism. Future research should use more specific tools—such as gene knockout models and selective inhibitors—to verify this pathway’s role. Additionally, pharmacokinetic analyses are needed to determine its duration.

This study has several limitations that need to be further optimized in future work. First, the experimental design failed to systematically compare the efficacy of different EA frequencies, current intensities, or acupoint combinations, while lacking sham acupuncture, simple acupuncture, and more adequate control group settings, which makes it challenging to determine the optimal treatment plan and accurately elucidate its mechanisms. Second, regarding mechanism exploration, although we used the autophagy inhibitor 3-MA for intervention, this tool is non-specific and does not include specific validation methods such as gene knockdown or mutation targeting PGAM5 or FUNDC1. Therefore, the conclusions about the role of the PGAM5/FUNDC1 axis in electroacupuncture neuroprotection still require more direct evidence. Additionally, the research focuses on the neuronal response in the areas affected by the MCAO model (mainly the striatum and cortex), without delving into the potential effects of astrocytes and microglia on neuronal autophagy and apoptosis through pathways such as regulating neuroinflammation and maintaining mitochondrial homeostasis. Furthermore, the present study only used male animal models to control hormonal variations, without examining the impact of gender differences, which is a known influencing factor for stroke prognosis [18, 31]. Thus, future efforts should focus on improving experimental design, systematically optimizing acupuncture parameters, and employing multicellular strategies such as conditional gene knockout and RNA interference, as well as validating in both male and female models, thereby providing more solid evidence for a comprehensive and in-depth elucidation of the neuroprotective mechanisms of acupuncture.

## Supplementary Information

Additional file 1.

Additional file 2.

Additional file 3.

Additional file 4.

Additional file 5.

Additional file 6.

Additional filze 7.

Additional file 8.

Additional file 9.

Additional file 10.

Additional file 11.

Additional file 12.

Additional file 13.

Additional file 14.

Additional file 15.

Additional file 16.

Additional file 17.

Additional file 18.

Additional file 19.

Additional file 20.

Additional file 21.

Additional file 22.

Additional file 23.

Additional file 24.

Additional file 25.

Additional file 26.

Additional file 27.

Additional file 28.

Additional file 29.

Additional file 30.

Additional file 31.

Additional file 32.

Additional file 33.

Additional file 34.

Additional file 35.

Additional file 36.

Additional file 37.

Additional file 38.

Additional file 39.

Additional file 40.

Additional file 41.

Additional file 42.

Additional file 43.

Additional file 44.

Additional file 45.

Additional file 46.

Additional file 47.

Additional file 48.

Additional file 49.

Additional file 50.

Additional file 51.

Additional file 52.

Additional file 53.

Additional file 54.

Additional file 55.

Additional file 56.

Additional file 57.

Additional file 58.

Additional file 59.

Additional file 60.

Additional file 61.

Additional file 62.

Additional file 63.

Additional file 64.

Additional file 65.

Additional file 66.

Additional file 67.

Additional file 68.

Additional file 69.

Additional file 70.

Additional file 71.

Additional file 72.

Additional file 73.

Additional file 74.

Additional file 75.

Additional file 76.

Additional file 77.

Additional file 78.

Additional file 79.

Additional file 80.

Additional file 81.

Additional file 82.

Additional file 83.

Additional file 84.

Additional file 85.

Additional file 86.

Additional file 87.

Additional file 88.

Additional file 89.

Additional file 90.

Additional file 91.

Additional file 92.

Additional file 93.

Additional file 94.

Additional file 95.

Additional file 96.

Additional file 97.

Additional file 98.

Additional file 99.

Additional file 100.

Additional file 101.

Additional file 102.

Additional file 103.

Additional file 104.

Additional file 105.

Additional file 106.

Additional file 107.

Additional file 108.

Additional file 109.

Additional file 110.

Additional file 111.

Additional file 112.

Additional file 113.

Additional file 114.

Additional file 115.

Additional file 116.

Additional file 117.

Additional file 118.

Additional file 119.

Additional file 120.

Additional file 121.

Additional file 122.

Additional file 123.

Additional file 124.

Additional file 125.

Additional file 126.

Additional file 127.

Additional file 128.

Additional file 129.

Additional file 130.

Additional file 131.

Additional file 132.

Additional file 133.

Additional file 134.

## Data Availability

No datasets were generated or analysed during the current study.

## Abbreviations

3-MA: 3-methyladenine
ANOVA: analysis of variance
ATP: adenosine triphosphate
BNIP3: BCL2/adenovirus E1B 19kDa interacting protein 3
BSA: bovine serum albumin
CIRI: cerebral ischemia reperfusion injury
CIRI: casein kinase II
Cyt c: cytochrome C
DAPI: 4,6-diamidino-2-phenylindole
EA: electroacupuncture
ER: endoplasmic reticulum
FUNDC1: FUN14 domain-containing protein 1
IACUC: institutional animal care and use committee
LC3: microtubule-associated protein light chain 3
MCAO: middle cerebral artery occlusion
MMP: mitochondrial membrane potential
mTORC1: mechanistic target of rapamycin complex 1
OCT: optimal cutting temperature compound
PGAM5: phosphoglycerate mutase 5
PINK1: PTEN induced putative kinase 1
ROS: reactive oxygen species
SD: sprague dawley
SDS-PAGE: sodium dodecyl sulfate-polyacrylamide gel electrophoresis
SEM: standard error of the mean
TME: transmission electron microscopy
TOMM20: translocase of outer mitochondrial membrane 20
TTC: 2,3,5-triphenyl tetrazolium chloride
TUNEL: terminal deoxynucleotidyl transferase dUTP nick end labeling staining

## References

[1] An H, Zhou B, Ji X. Mitochondrial quality control in acute ischemic stroke. J Cereb Blood Flow Metab. 2021;41(12):3157–70. 10.1177/0271678x211046992. 34551609 10.1177/0271678X211046992 PMC8669286

[2] Andrabi SS, Parvez S, Tabassum H. Ischemic stroke and mitochondria: mechanisms and targets. Protoplasma. 2020;257(2):335–43. 10.1007/s00709-019-01439-2. 31612315 10.1007/s00709-019-01439-2

[3] Baechler BL, Bloemberg D, Quadrilatero J. Mitophagy regulates mitochondrial network signaling, oxidative stress, and apoptosis during myoblast differentiation. Autophagy. 2019;15(9):1606–19. 10.1080/15548627.2019.1591672. 30859901 10.1080/15548627.2019.1591672 PMC6693454

[4] Cai C, Guo Z, Chang X, Li Z, Wu F, He J, et al. Empagliflozin attenuates cardiac microvascular ischemia/reperfusion through activating the AMPKα1/ULK1/FUNDC1/mitophagy pathway. Redox Biol. 2022;52:102288. 10.1016/j.redox.2022.102288. 35325804 10.1016/j.redox.2022.102288 PMC8938627

[5] Chen Y, Tang W, Huang X, An Y, Li J, Yuan S, et al. Mitophagy in intracerebral hemorrhage: a new target for therapeutic intervention. Neural Regen Res. 2024;19(2):316–23. 10.4103/1673-5374.379019. 37488884 10.4103/1673-5374.379019 PMC10503626

[6] Chen H, Yang X, Gao Y, Jiang H, Guo M, Zhou Y, et al. Electroacupuncture ameliorates cognitive impairment in APP/PS1 mouse by modulating TFEB levels to relieve ALP dysfunction. Brain Res. 2024;1823:148683. 10.1016/j.brainres.2023.148683. 37992796 10.1016/j.brainres.2023.148683

[7] Cheng Y, Wu B, Huang J, Chen Y. Research progress on the mechanisms of central post-stroke pain: a review. Cell Mol Neurobiol. 2023;43(7):3083–98. 10.1007/s10571-023-01360-6. 37166685 10.1007/s10571-023-01360-6 PMC11409963

[8] Chu C, Wang X, Yang C, Chen F, Shi L, Xu W, et al. Neutrophil extracellular traps drive intestinal microvascular endothelial ferroptosis by impairing Fundc1-dependent mitophagy. Redox Biol. 2023;67:102906. 10.1016/j.redox.2023.102906. 37812880 10.1016/j.redox.2023.102906 PMC10579540

[9] Danaei G, Finucane MM, Lu Y, Singh GM, Cowan MJ, Paciorek CJ, et al. National, regional, and global trends in fasting plasma glucose and diabetes prevalence since 1980: systematic analysis of health examination surveys and epidemiological studies with 370 country-years and 2·7 million participants. Lancet. 2011;378(9785):31–40. 10.1016/s0140-6736(11)60679-x. 21705069 10.1016/S0140-6736(11)60679-X

[10] Feske SK. Ischemic stroke. Am J Med. 2021;134(12):1457–64. 10.1016/j.amjmed.2021.07.027. 34454905 10.1016/j.amjmed.2021.07.027

[11] Guan R, Li Z, Dai X, Zou W, Yu X, Liu H, et al. Electroacupuncture at GV20-GB7 regulates mitophagy to protect against neurological deficits following intracerebral hemorrhage via inhibition of apoptosis. Mol Med Rep. 2021. 10.3892/mmr.2021.12131. 10.3892/mmr.2021.12131 PMC8127033 33955500

[12] Guan R, Zou W, Dai X, Yu X, Liu H, Chen Q, et al. Mitophagy, a potential therapeutic target for stroke. J Biomed Sci. 2018;25(1):87. 10.1186/s12929-018-0487-4. 30501621 10.1186/s12929-018-0487-4 PMC6271612

[13] Han CH, Kim JH, Kim M, Kim HR, Kim SY, Choi HY, et al. Electroacupuncture for post-stroke dysphagia: a protocol for systematic review and meta-analysis of randomized controlled trials. Medicine (Baltimore). 2020;99(38):e22360. 10.1097/md.0000000000022360. 32957409 10.1097/MD.0000000000022360 PMC7505295

[14] Han JZ, Yang Y, Wang YF, Feng JH, Song CN, Wu WJ, et al. Effectiveness and safety of Governor vessel acupuncture therapy for post-stroke cognitive impairment: a meta-analysis of randomized controlled trials. Ageing Res Rev. 2024;99:102355. 10.1016/j.arr.2024.102355. 38942201 10.1016/j.arr.2024.102355

[15] Hobden G, Demeyere N. Information needs of stroke survivors and their family members regarding post-stroke cognition: a scoping review protocol. JBI Evid Synth. 2024;22(4):720–6. 10.11124/jbies-23-00045. 37975430 10.11124/JBIES-23-00045

[16] Huang YG, Tao W, Yang SB, Wang JF, Mei ZG, Feng ZT. Autophagy: novel insights into therapeutic target of electroacupuncture against cerebral ischemia/ reperfusion injury. Neural Regen Res. 2019;14(6):954–61. 10.4103/1673-5374.250569. 30761999 10.4103/1673-5374.250569 PMC6404501

[17] Ichimura Y, Komatsu M. Pathophysiological role of autophagy: lesson from autophagy-deficient mouse models. Exp Anim. 2011;60(4):329–45. 10.1538/expanim.60.329. 21791873 10.1538/expanim.60.329

[18] Kuppuswamy S, Watson NJ, Ledford WL, Pavri BA, Zhi W, Gbadebo M, et al. Brain proteome changes after intracerebral hemorrhage in aged male and female mice. Neurobiol Dis. 2025;212:106936. 10.1016/j.nbd.2025.106936. 40320180 10.1016/j.nbd.2025.106936 PMC12376891

[19] Lang J, Luo J, Lang J, Wang L, Xu W, Jia J, et al. Electroacupuncture alleviated post-stroke cognitive impairment via the mTOR/NLRP3-mediated autophagy-inflammatory pathway. Eur J Med Res. 2024;29(1):532. 10.1186/s40001-024-02131-9. 39497200 10.1186/s40001-024-02131-9 PMC11536957

[20] Li SS, Hua XY, Zheng MX, Wu JJ, Ma ZZ, Xing XX, et al. Electroacupuncture treatment improves motor function and neurological outcomes after cerebral ischemia/reperfusion injury. Neural Regen Res. 2022;17(7):1545–55. 10.4103/1673-5374.330617. 34916440 10.4103/1673-5374.330617 PMC8771092

[21] Li G, Li J, Shao R, Zhao J, Chen M. FUNDC1: A promising mitophagy regulator at the mitochondria-associated membrane for cardiovascular diseases. Front Cell Dev Biol. 2021;9:788634. 10.3389/fcell.2021.788634. 35096821 10.3389/fcell.2021.788634 PMC8797154

[22] Li F, Quan J, Wen Q. Effect of electroacupuncture and scalp acupuncture combined with language rehabilitation training on cognitive and speech functions of aphasia patients after craniocerebral injury. Am J Transl Res. 2022;14(8):5923–30. 36105057 PMC9452345

[23] Liu W, Shang G, Yang S, Huang J, Xue X, Lin Y, et al. Electroacupuncture protects against ischemic stroke by reducing autophagosome formation and inhibiting autophagy through the mTORC1-ULK1 complex-Beclin1 pathway. Int J Mol Med. 2016;37(2):309–18. 10.3892/ijmm.2015.2425. 26647915 10.3892/ijmm.2015.2425 PMC4716798

[24] Longa EZ, Weinstein PR, Carlson S, Cummins R. Reversible middle cerebral artery occlusion without craniectomy in rats. Stroke. 1989;20(1):84–91. 10.1161/01.str.20.1.84. 2643202 10.1161/01.str.20.1.84

[25] Magnusson M, Johansson K, Johansson BB. Sensory stimulation promotes normalization of postural control after stroke. Stroke. 1994;25(6):1176–80. 10.1161/01.str.25.6.1176. 8202976 10.1161/01.str.25.6.1176

[26] Mei ZG, Huang YG, Feng ZT, Luo YN, Yang SB, Du LP, et al. Electroacupuncture ameliorates cerebral ischemia/reperfusion injury by suppressing autophagy via the SIRT1-FOXO1 signaling pathway. Aging (Albany NY). 2020;12(13):13187–205. 10.18632/aging.103420. 32620714 10.18632/aging.103420 PMC7377856

[27] Mizushima N, Yamamoto A, Matsui M, Yoshimori T, Ohsumi Y. In vivo analysis of autophagy in response to nutrient starvation using transgenic mice expressing a fluorescent autophagosome marker. Mol Biol Cell. 2004;15(3):1101–11. 10.1091/mbc.e03-09-0704. 14699058 10.1091/mbc.E03-09-0704 PMC363084

[28] Paxinos G, Watson CR, Emson PC. AChE-stained horizontal sections of the rat brain in stereotaxic coordinates. J Neurosci Methods. 1980;3(2):129–49. 10.1016/0165-0270(80)90021-7. 6110810 10.1016/0165-0270(80)90021-7

[29] Plomaritis P, Theodorou A, Michalaki V, Stefanou MI, Palaiodimou L, Papagiannopoulou G, et al. Periodic limb movements during sleep in acute stroke: prevalence, severity and impact on post-stroke recovery. J Clin Med. 2023. 10.3390/jcm12185881. 10.3390/jcm12185881 PMC10531709 37762823

[30] Ren X, Gao X, Li Z, Ding Y, Xu A, Du L, et al. Electroacupuncture ameliorates neuroinflammation by inhibiting TRPV4 channel in ischemic stroke. CNS Neurosci Ther. 2024;30(2):e14618. 10.1111/cns.14618. 38334061 10.1111/cns.14618 PMC10853892

[31] Roy-O’Reilly M, McCullough LD. Sex differences in stroke: the contribution of coagulation. Exp Neurol. 2014;259:16–27. 10.1016/j.expneurol.2014.02.011. 24560819 10.1016/j.expneurol.2014.02.011 PMC4127336

[32] Saul H, Cassidy S, Deeney B, Imison C, Brady M. Early, intense therapy for language problems after a stroke is linked to the greatest benefits. BMJ. 2023;383:2560. 10.1136/bmj.p2560. 37963608 10.1136/bmj.p2560

[33] Shao Z, Dou S, Zhu J, Wang H, Xu D, Wang C, et al. The role of mitophagy in ischemic stroke. Front Neurol. 2020;11:608610. 10.3389/fneur.2020.608610. 33424757 10.3389/fneur.2020.608610 PMC7793663

[34] She R, Liu D, Liao J, Wang G, Ge J, Mei Z. Mitochondrial dysfunctions induce PANoptosis and ferroptosis in cerebral ischemia/reperfusion injury: from pathology to therapeutic potential. Front Cell Neurosci. 2023;17:1191629. 10.3389/fncel.2023.1191629. 37293623 10.3389/fncel.2023.1191629 PMC10244524

[35] Sheng R, Zhang LS, Han R, Liu XQ, Gao B, Qin ZH. Autophagy activation is associated with neuroprotection in a rat model of focal cerebral ischemic preconditioning. Autophagy. 2010;6(4):482–94. 10.4161/auto.6.4.11737. 20400854 10.4161/auto.6.4.11737

[36] Song M, Zhou Y, Fan X. Mitochondrial quality and quantity control: mitophagy is a potential therapeutic target for ischemic stroke. Mol Neurobiol. 2022;59(5):3110–23. 10.1007/s12035-022-02795-6. 35266113 10.1007/s12035-022-02795-6

[37] Su J, Zhang T, Wang K, Zhu T, Li X. Autophagy activation contributes to the neuroprotection of remote ischemic perconditioning against focal cerebral ischemia in rats. Neurochem Res. 2014;39(11):2068–77. 10.1007/s11064-014-1396-x. 25082119 10.1007/s11064-014-1396-x

[38] Sugo M, Kimura H, Arasaki K, Amemiya T, Hirota N, Dohmae N, et al. Syntaxin 17 regulates the localization and function of PGAM5 in mitochondrial division and mitophagy. EMBO J. 2018. 10.15252/embj.201798899. 10.15252/embj.201798899 PMC6213275 30237312

[39] Sun Y, Zhu Y, Zhong X, Chen X, Wang J, Ying G. Crosstalk between autophagy and cerebral ischemia. Front Neurosci. 2018;12:1022. 10.3389/fnins.2018.01022. 30692904 10.3389/fnins.2018.01022 PMC6339887

[40] Tan N, Liu T, Wang X, Shao M, Zhang M, Li W, et al. The multi-faced role of FUNDC1 in mitochondrial events and human diseases. Front Cell Dev Biol. 2022;10:918943. 10.3389/fcell.2022.918943. 35959490 10.3389/fcell.2022.918943 PMC9358025

[41] Tian H, Chen X, Liao J, Yang T, Cheng S, Mei Z, et al. Mitochondrial quality control in stroke: from the mechanisms to therapeutic potentials. J Cell Mol Med. 2022;26(4):1000–12. 10.1111/jcmm.17189. 35040556 10.1111/jcmm.17189 PMC8831937

[42] Wan W, Wang Y, Li L, Ma C, Wang Y, You F. Electroacupuncture improves learning and memory abilities via activating AMPK/mTOR-induced autophagy in APP/PS1 mice. Biochem Genet. 2024;62(4):2540–52. 10.1007/s10528-023-10503-9. 37980310 10.1007/s10528-023-10503-9

[43] Wang H, Chen S, Zhang Y, Xu H, Sun H. Electroacupuncture ameliorates neuronal injury by Pink1/Parkin-mediated mitophagy clearance in cerebral ischemia-reperfusion. Nitric Oxide. 2019;91:23–34. 10.1016/j.niox.2019.07.004. 31323277 10.1016/j.niox.2019.07.004

[44] Wang X, Jiang Y, Zhang Y, Sun Q, Ling G, Jiang J, et al. The roles of the mitophagy inducer Danqi pill in heart failure: a new therapeutic target to preserve energy metabolism. Phytomedicine. 2022;99:154009. 10.1016/j.phymed.2022.154009. 35217438 10.1016/j.phymed.2022.154009

[45] Wang Z, Li Q, Yang H, Zhang D, Zhang Y, Wang J, et al. 5-Heptadecylresorcinol ameliorates obesity-associated skeletal muscle mitochondrial dysfunction through SIRT3-mediated mitophagy. J Agric Food Chem. 2023;71(43):16032–42. 10.1021/acs.jafc.3c01452. 37862266 10.1021/acs.jafc.3c01452

[46] Wang HL, Liu FL, Li RQ, Wan MY, Li JY, Shi J, et al. Electroacupuncture improves learning and memory functions in a rat cerebral ischemia/reperfusion injury model through PI3K/Akt signaling pathway activation. Neural Regen Res. 2021;16(6):1011–6. 10.4103/1673-5374.300454. 33269744 10.4103/1673-5374.300454 PMC8224106

[47] Wong AM, Su TY, Tang FT, Cheng PT, Liaw MY. Clinical trial of electrical acupuncture on hemiplegic stroke patients. Am J Phys Med Rehabil. 1999;78(2):117–22. 10.1097/00002060-199903000-00006. 10088585 10.1097/00002060-199903000-00006

[48] Wu X, Zheng Y, Liu M, Li Y, Ma S, Tang W, et al. BNIP3L/NIX degradation leads to mitophagy deficiency in ischemic brains. Autophagy. 2021;17(8):1934–46. 10.1080/15548627.2020.1802089. 32722981 10.1080/15548627.2020.1802089 PMC8386707

[49] Xie Y, Zhou Y, Wang J, Du L, Ren Y, Liu F. Ferroptosis, autophagy, tumor and immunity. Heliyon. 2023;9(9):e19799. 10.1016/j.heliyon.2023.e19799. 37810047 10.1016/j.heliyon.2023.e19799 PMC10559173

[50] Xu J, Deng Z, Shang S, Wang C, Han H. FUNDC1 collaborates with PINK1 in regulating mitochondrial fission and compensating for PINK1 deficiency. Biochem Biophys Res Commun. 2023;687:149210. 10.1016/j.bbrc.2023.149210. 37931419 10.1016/j.bbrc.2023.149210

[51] Xu Y, Shen J, Ran Z. Emerging views of mitophagy in immunity and autoimmune diseases. Autophagy. 2020;16(1):3–17. 10.1080/15548627.2019.1603547. 30951392 10.1080/15548627.2019.1603547 PMC6984455

[52] Yang K, Wu J, Li S, Wang S, Zhang J, Wang YP, et al. NTRK1 knockdown induces mouse cognitive impairment and hippocampal neuronal damage through mitophagy suppression via inactivating the AMPK/ULK1/FUNDC1 pathway. Cell Death Discov. 2023;9(1):404. 10.1038/s41420-023-01685-7. 37907480 10.1038/s41420-023-01685-7 PMC10618268

[53] Yang G, Zhang L, Yuan Y, Mazhar M, Zhang D, Liu Y, et al. Electroacupuncture improves microglial polarization induced-inflammation by regulating the TGF-β/Smad-3 signaling pathway in ischemic stroke mice. CNS Neurosci Ther. 2025;31(8):e70567. 10.1111/cns.70567. 40831324 10.1111/cns.70567 PMC12365391

[54] Yao L, Ye Q, Liu Y, Yao S, Yuan S, Xu Q, et al. Electroacupuncture improves swallowing function in a post-stroke dysphagia mouse model by activating the motor cortex inputs to the nucleus tractus solitarii through the parabrachial nuclei. Nat Commun. 2023;14(1):810. 10.1038/s41467-023-36448-6. 36781899 10.1038/s41467-023-36448-6 PMC9925820

[55] Ye H, Li D, Wei X, Yu L, Jia L. Focused low-intensity pulsed ultrasound alleviates osteoarthritis via restoring impaired FUNDC1-mediated mitophagy. iScience. 2023;26(10):107772. 10.1016/j.isci.2023.107772. 37720103 10.1016/j.isci.2023.107772 PMC10504546

[56] Yu W, Chang X, Liao J, Quan J, Liu S, He T, et al. Long-term oral tribasic copper chloride exposure impedes cognitive function and disrupts mitochondrial metabolism by inhibiting mitophagy in rats. Environ Pollut. 2023;336:122474. 10.1016/j.envpol.2023.122474. 37652230 10.1016/j.envpol.2023.122474

[57] Zeng Q, He H, Wang XB, Zhou YQ, Lin HX, Tan ZP, et al. Electroacupuncture preconditioning improves myocardial infarction injury via enhancing AMPK-dependent autophagy in rats. Biomed Res Int. 2018;2018:1238175. 10.1155/2018/1238175. 30175112 10.1155/2018/1238175 PMC6106955

[58] Zhang Y, Cao Y, Liu C. Autophagy and ischemic stroke. Adv Exp Med Biol. 2020;1207:111–34. 10.1007/978-981-15-4272-5_7. 32671742 10.1007/978-981-15-4272-5_7

[59] Zhang J, He X, Mi N. A lipid membrane-centric role of the SQSTM1/p62 body during autophagosome formation. Autophagy. 2024;20(5):1192–3. 10.1080/15548627.2023.2297622. 38115546 10.1080/15548627.2023.2297622 PMC11135812

[60] Zhang S, Wu B, Liu M, Li N, Zeng X, Liu H, et al. Acupuncture efficacy on ischemic stroke recovery: multicenter randomized controlled trial in China. Stroke. 2015;46(5):1301–6. 10.1161/strokeaha.114.007659. 25873601 10.1161/STROKEAHA.114.007659

[61] Zhang GF, Yang P, Yin Z, Chen HL, Ma FG, Wang B, et al. Electroacupuncture preconditioning protects against focal cerebral ischemia/reperfusion injury via suppression of dynamin-related protein 1. Neural Regen Res. 2018;13(1):86–93. 10.4103/1673-5374.224373. 29451211 10.4103/1673-5374.224373 PMC5840997

[62] Zou Y, Tao Z, Li P, Yang J, Xu Q, Xu X, et al. Clemastine attenuates subarachnoid haemorrhage pathology in a mouse model via Nrf2/SQSTM1-mediated autophagy. Br J Pharmacol. 2025;182(12):2730–53. 10.1111/bph.17465. 40052261 10.1111/bph.17465

