# Supplementary figures and images for: Electroacupuncture inhibited neuronal apoptosis through PGAM5/FUNDC1-dependent mitophagy after ischemic stroke (part 1 of 2)
Source: Chin Med. 2026 Apr 3;21:110. doi: 10.1186/s13020-026-01383-3 (PMC13047806; doi:10.1186/s13020-026-01383-3)

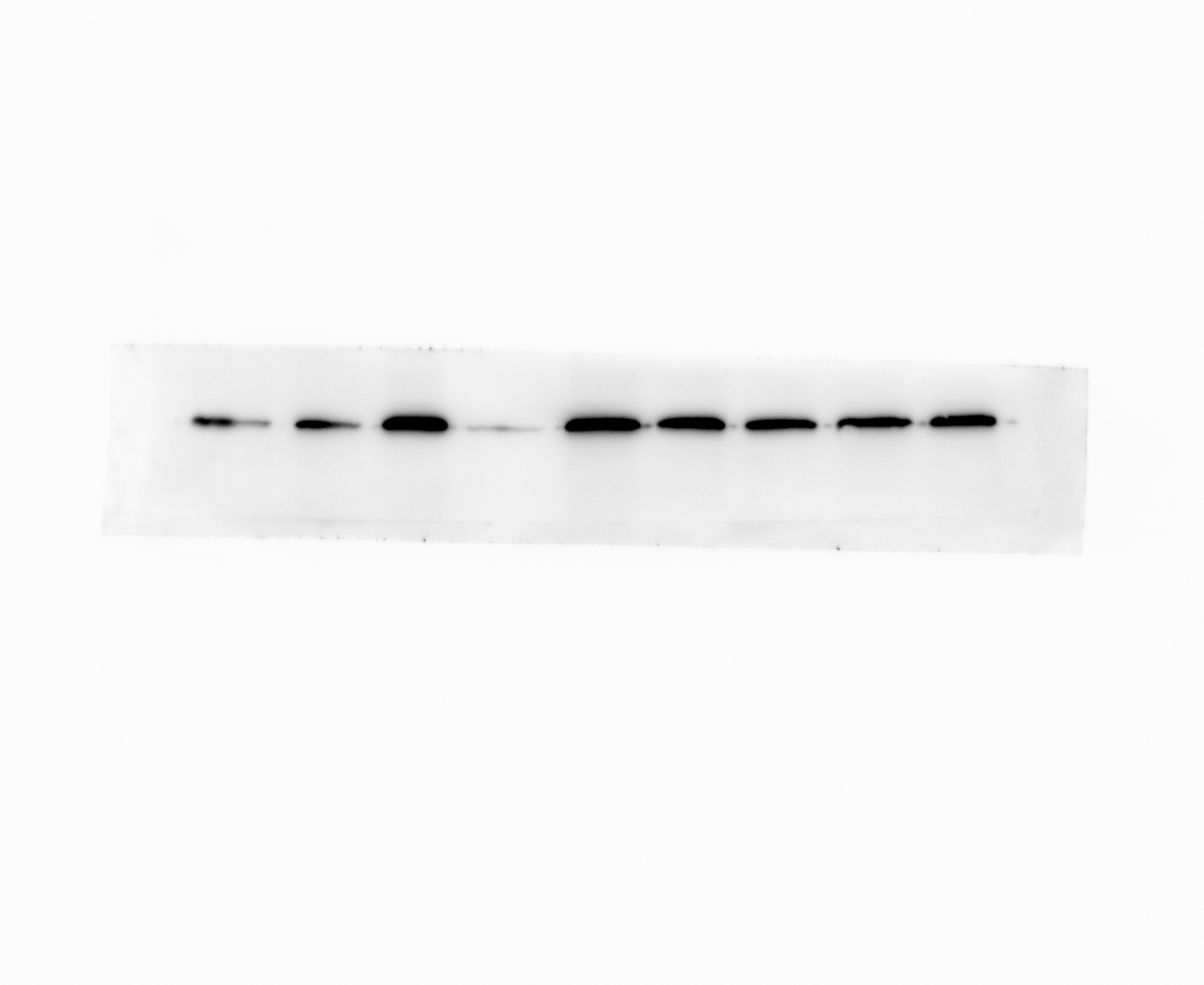

Supplement: Supplementary file 1 — Additional file 1. [file 13020_2026_1383_MOESM1_ESM.tif]

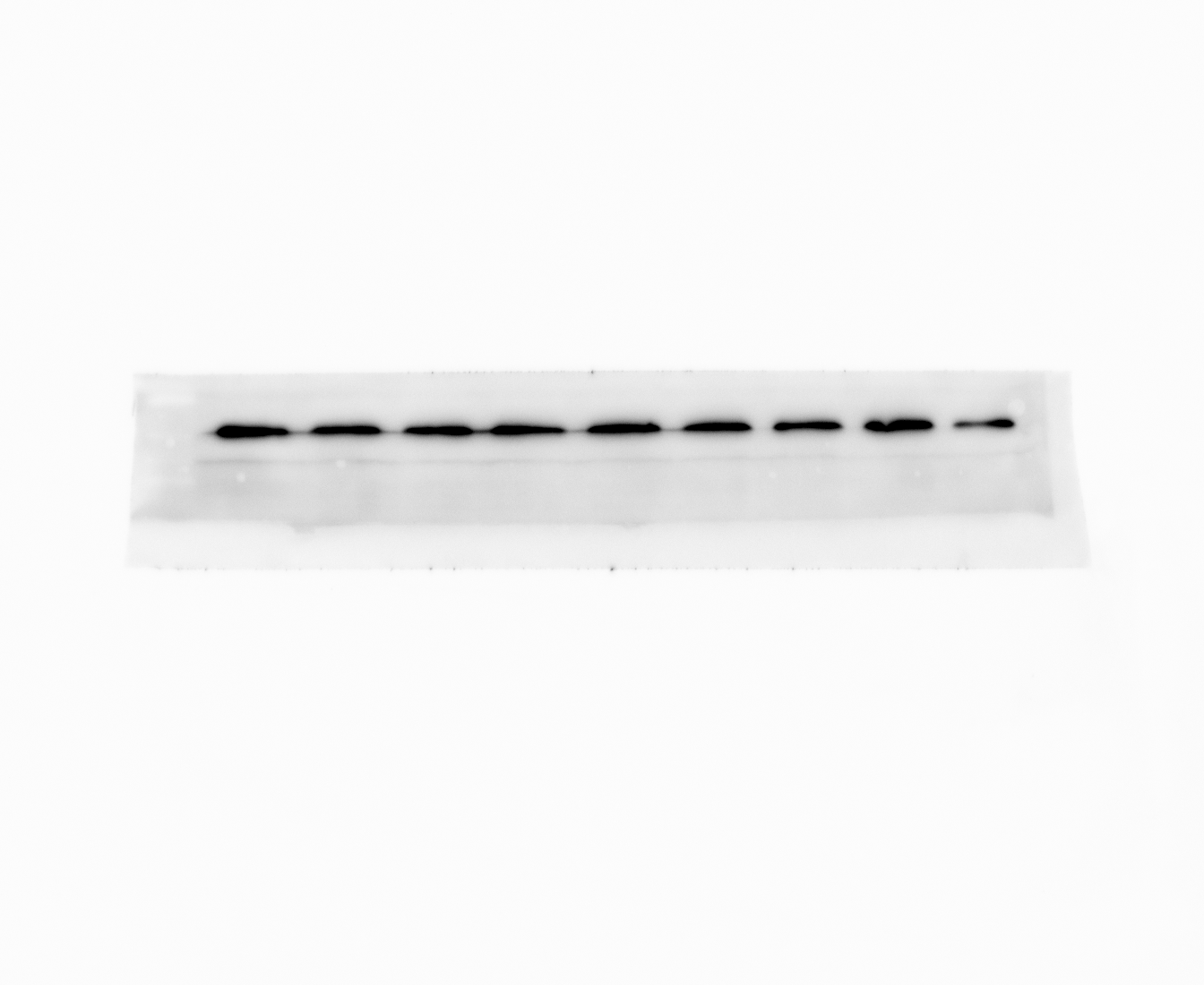

Supplement: Supplementary file 2 — Additional file 2. [file 13020_2026_1383_MOESM2_ESM.tif]

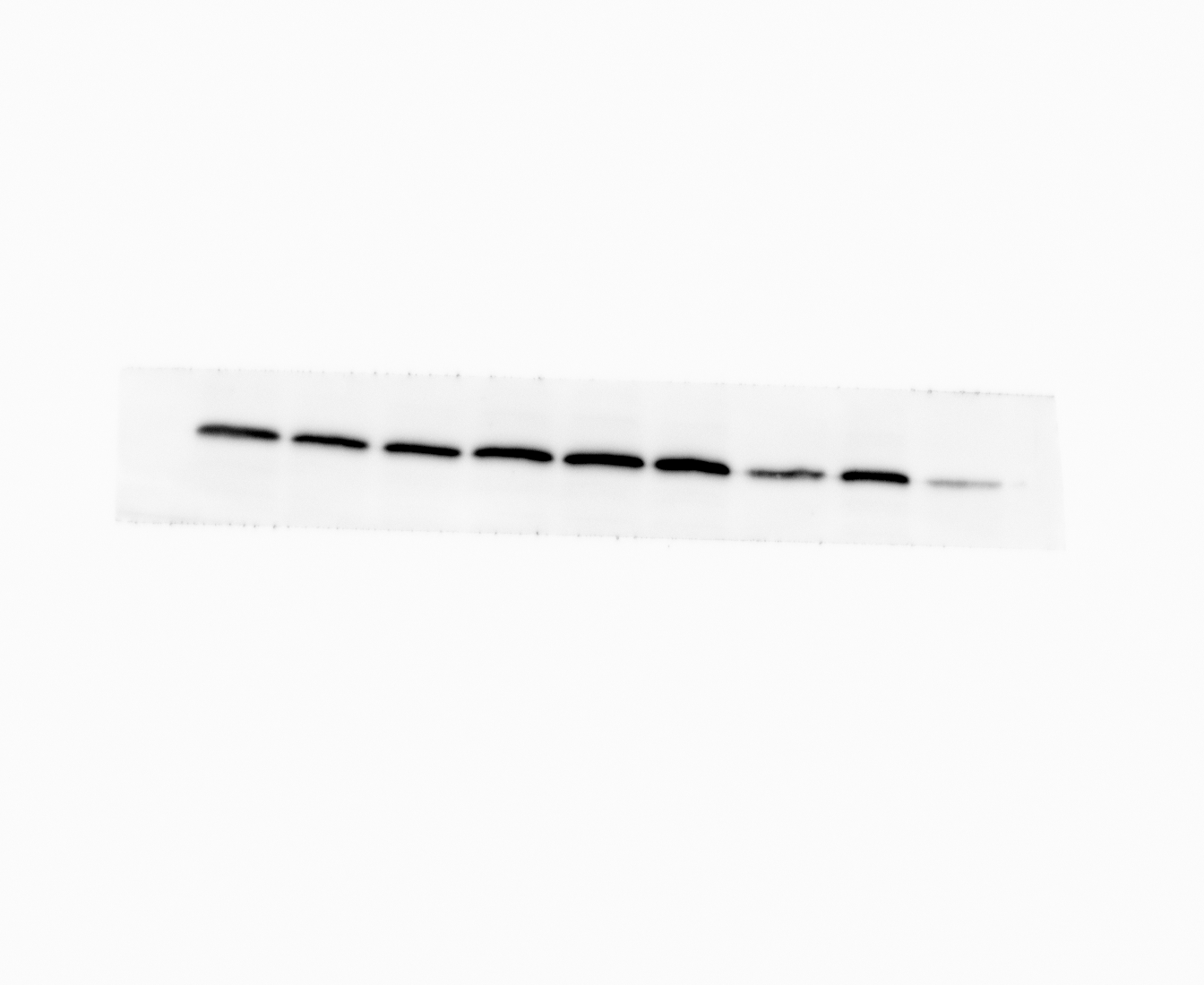

Supplement: Supplementary file 3 — Additional file 3. [file 13020_2026_1383_MOESM3_ESM.tif]

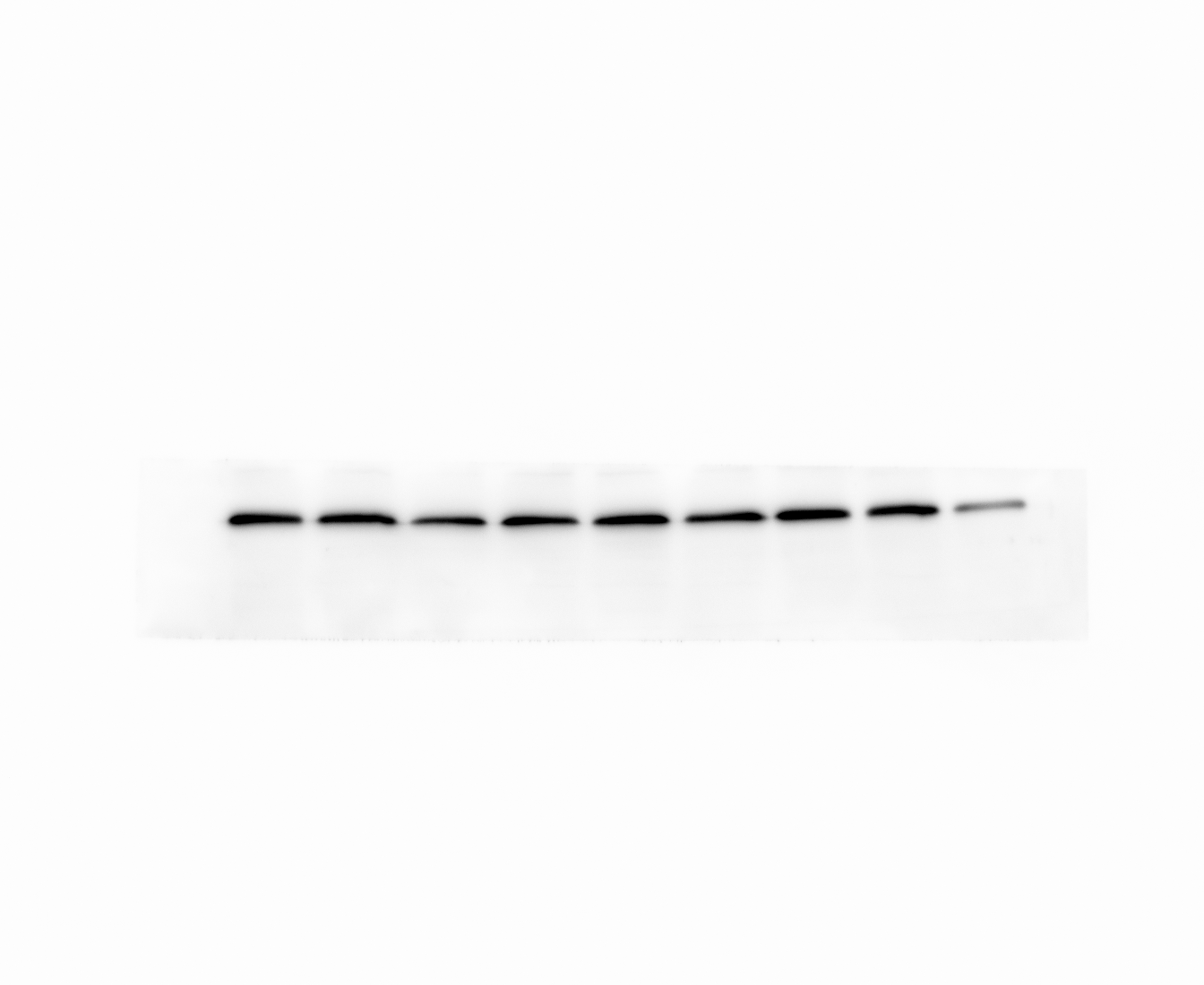

Supplement: Supplementary file 4 — Additional file 4. [file 13020_2026_1383_MOESM4_ESM.tif]

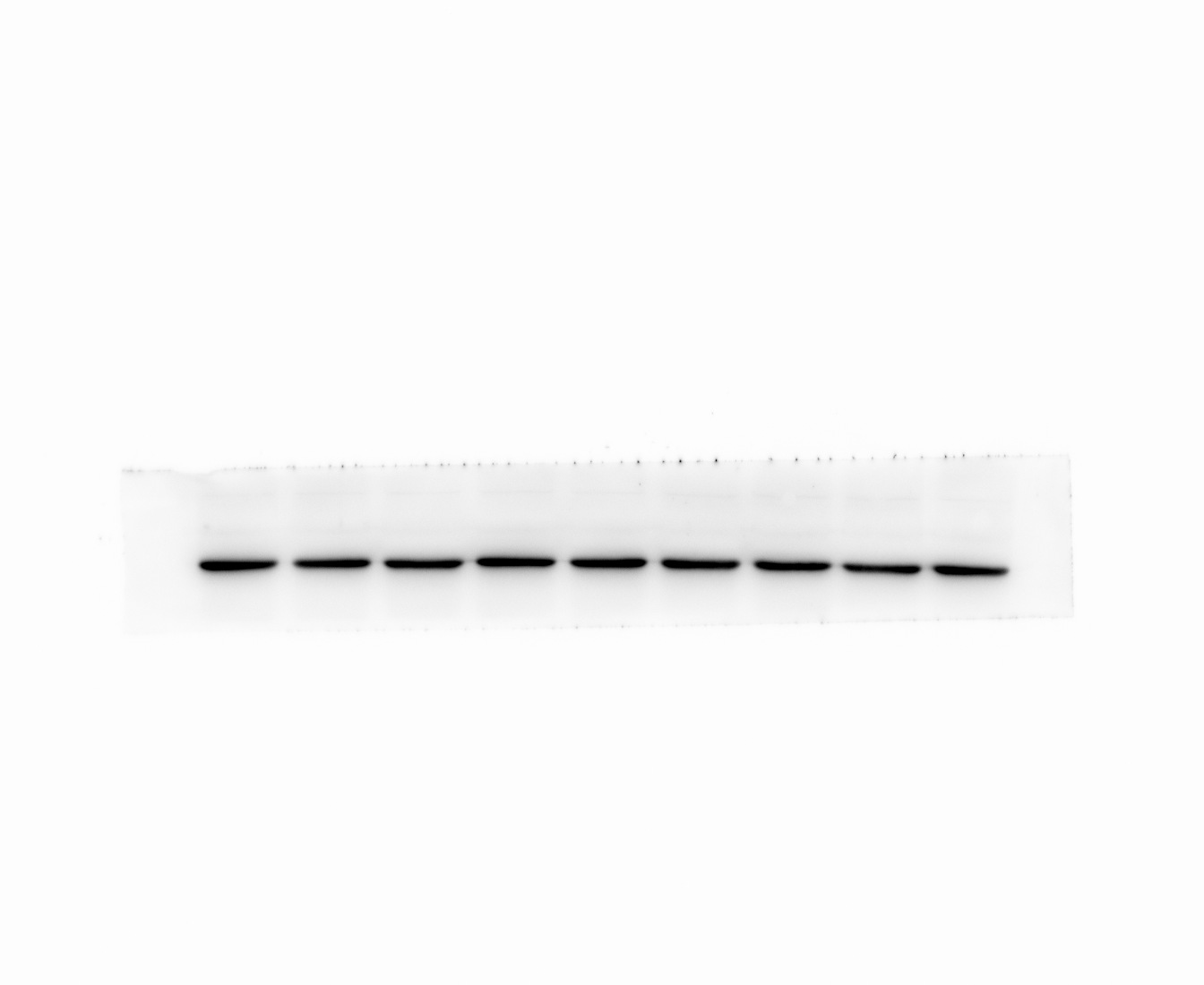

Supplement: Supplementary file 5 — Additional file 5. [file 13020_2026_1383_MOESM5_ESM.tif]

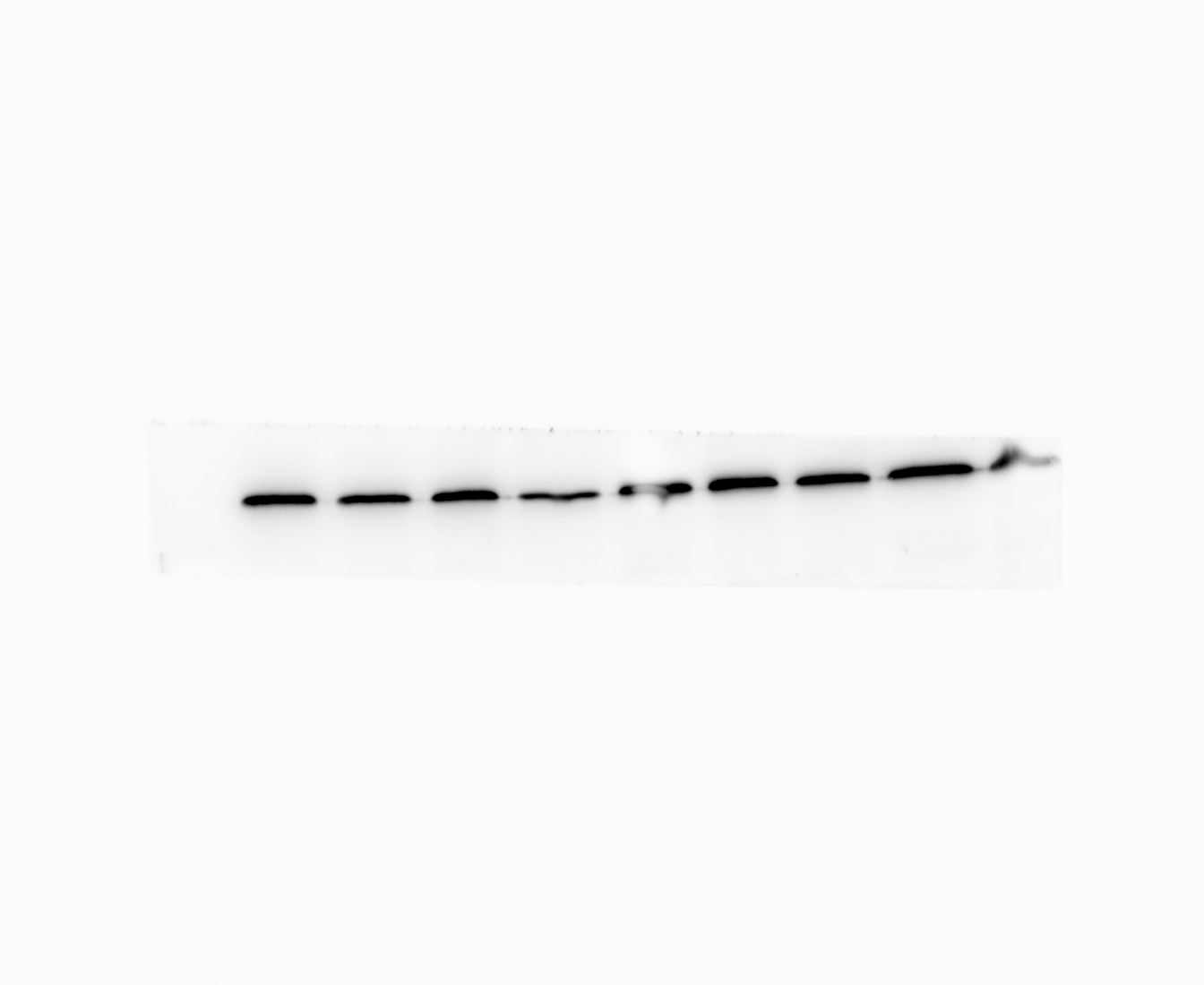

Supplement: Supplementary file 6 — Additional file 6. [file 13020_2026_1383_MOESM6_ESM.tif]

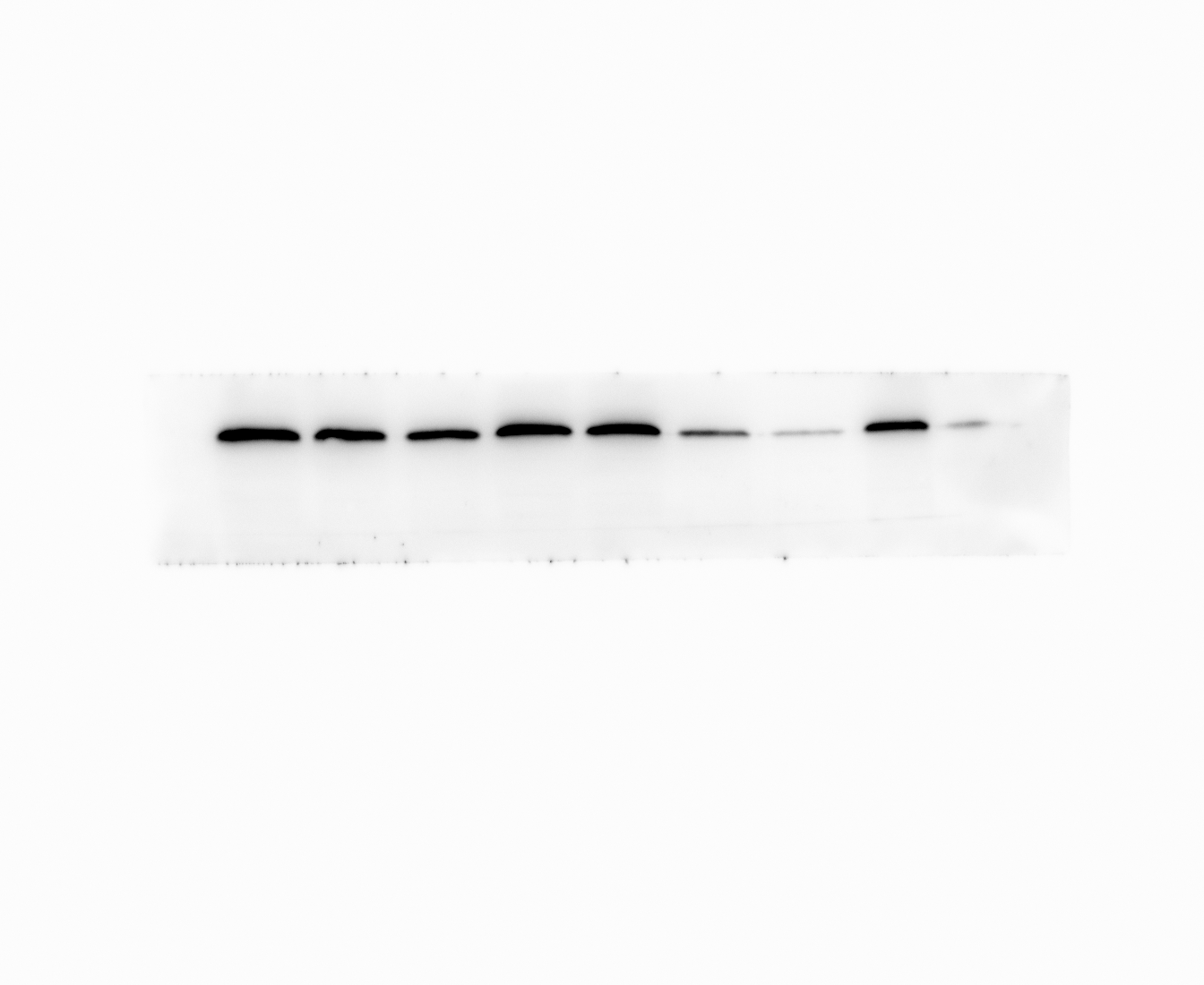

Supplement: Supplementary file 7 — Additional filze 7. [file 13020_2026_1383_MOESM7_ESM.tif]

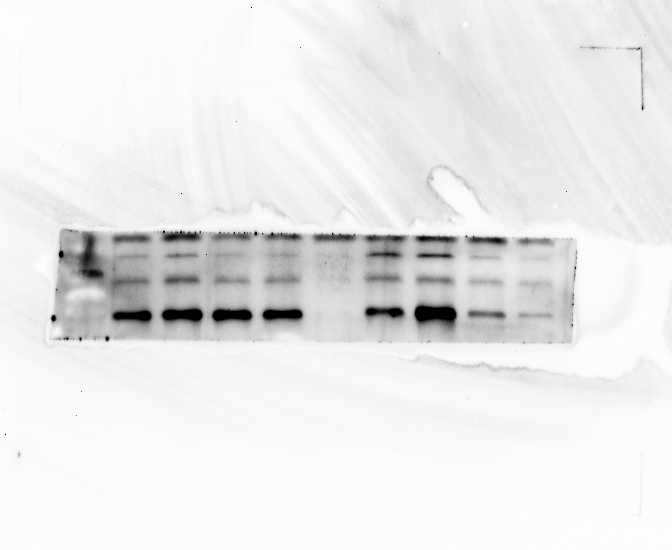

Supplement: Supplementary file 8 — Additional file 8. [file 13020_2026_1383_MOESM8_ESM.tif]

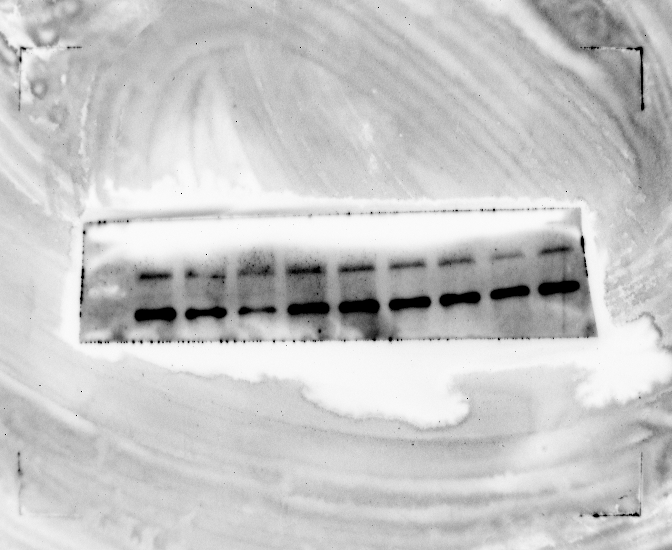

Supplement: Supplementary file 9 — Additional file 9. [file 13020_2026_1383_MOESM9_ESM.tif]

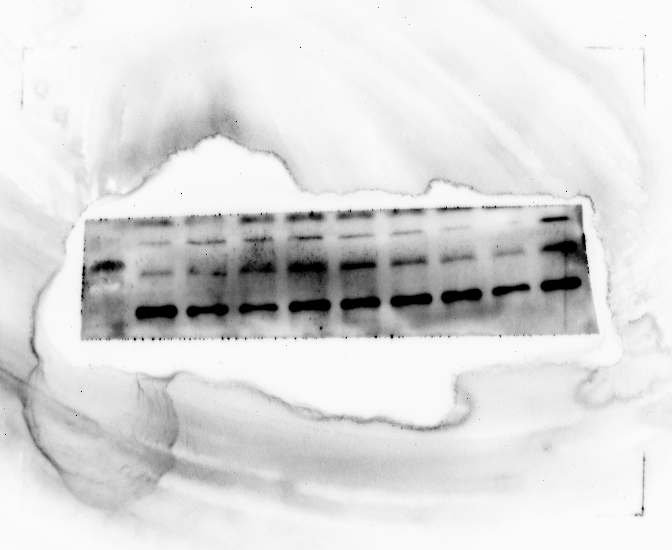

Supplement: Supplementary file 10 — Additional file 10. [file 13020_2026_1383_MOESM10_ESM.tif]

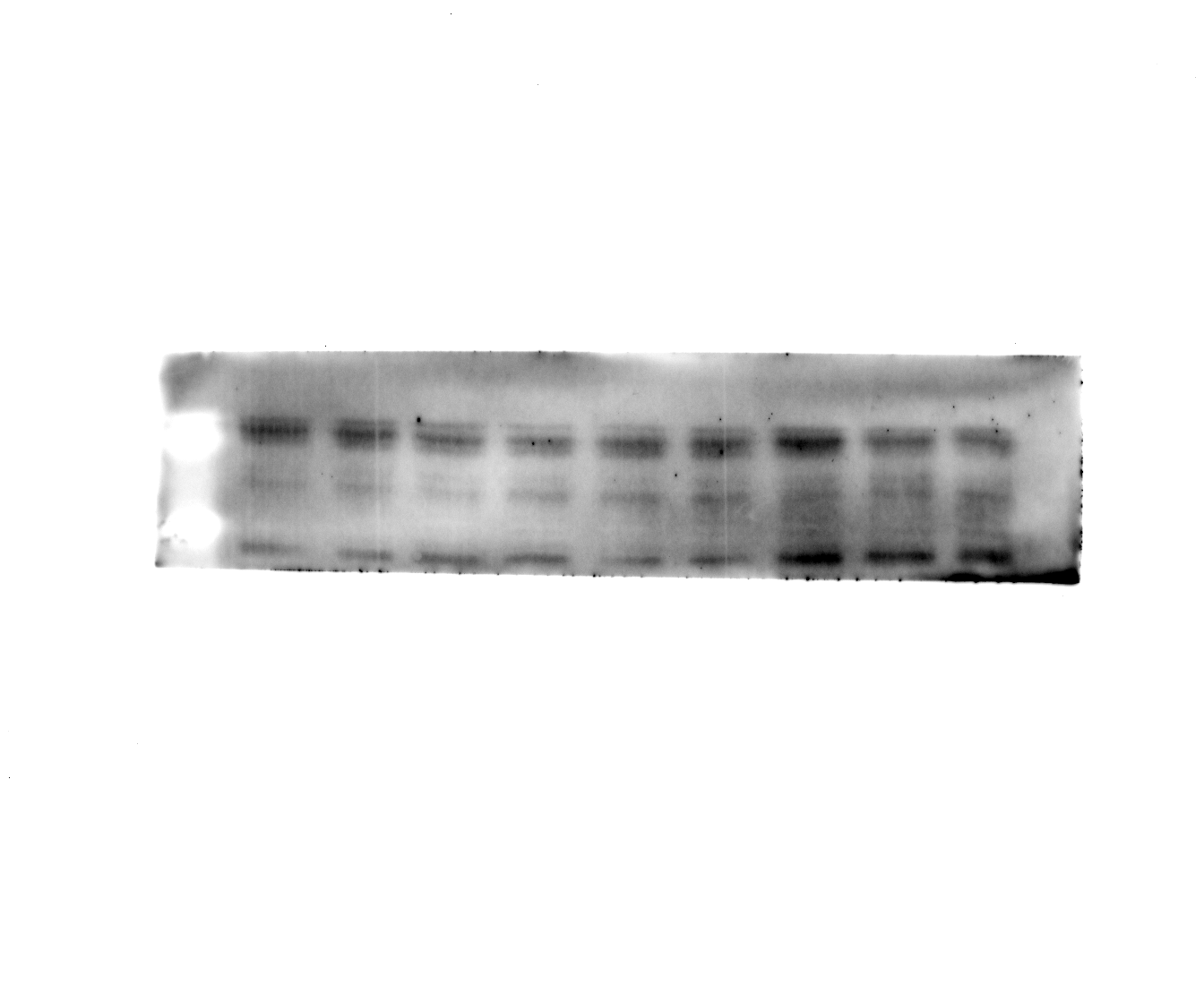

Supplement: Supplementary file 11 — Additional file 11. [file 13020_2026_1383_MOESM11_ESM.tif]

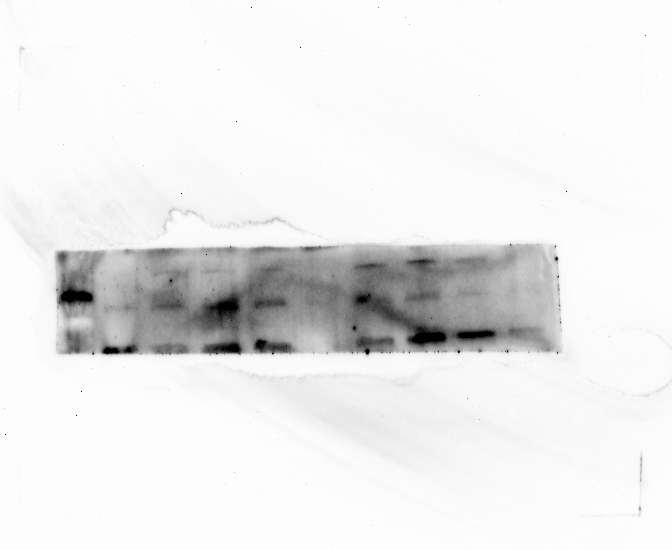

Supplement: Supplementary file 12 — Additional file 12. [file 13020_2026_1383_MOESM12_ESM.tif]

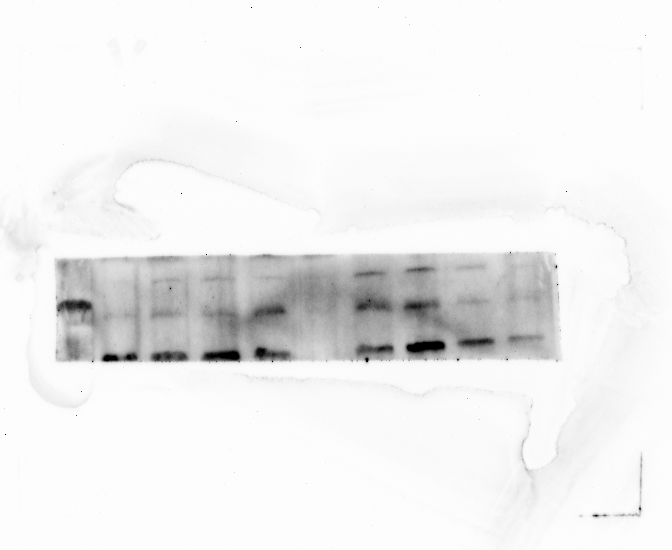

Supplement: Supplementary file 13 — Additional file 13. [file 13020_2026_1383_MOESM13_ESM.tif]

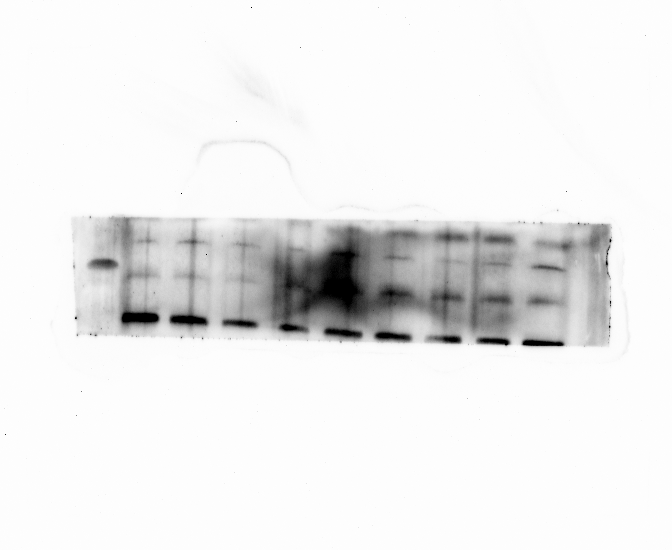

Supplement: Supplementary file 14 — Additional file 14. [file 13020_2026_1383_MOESM14_ESM.tif]

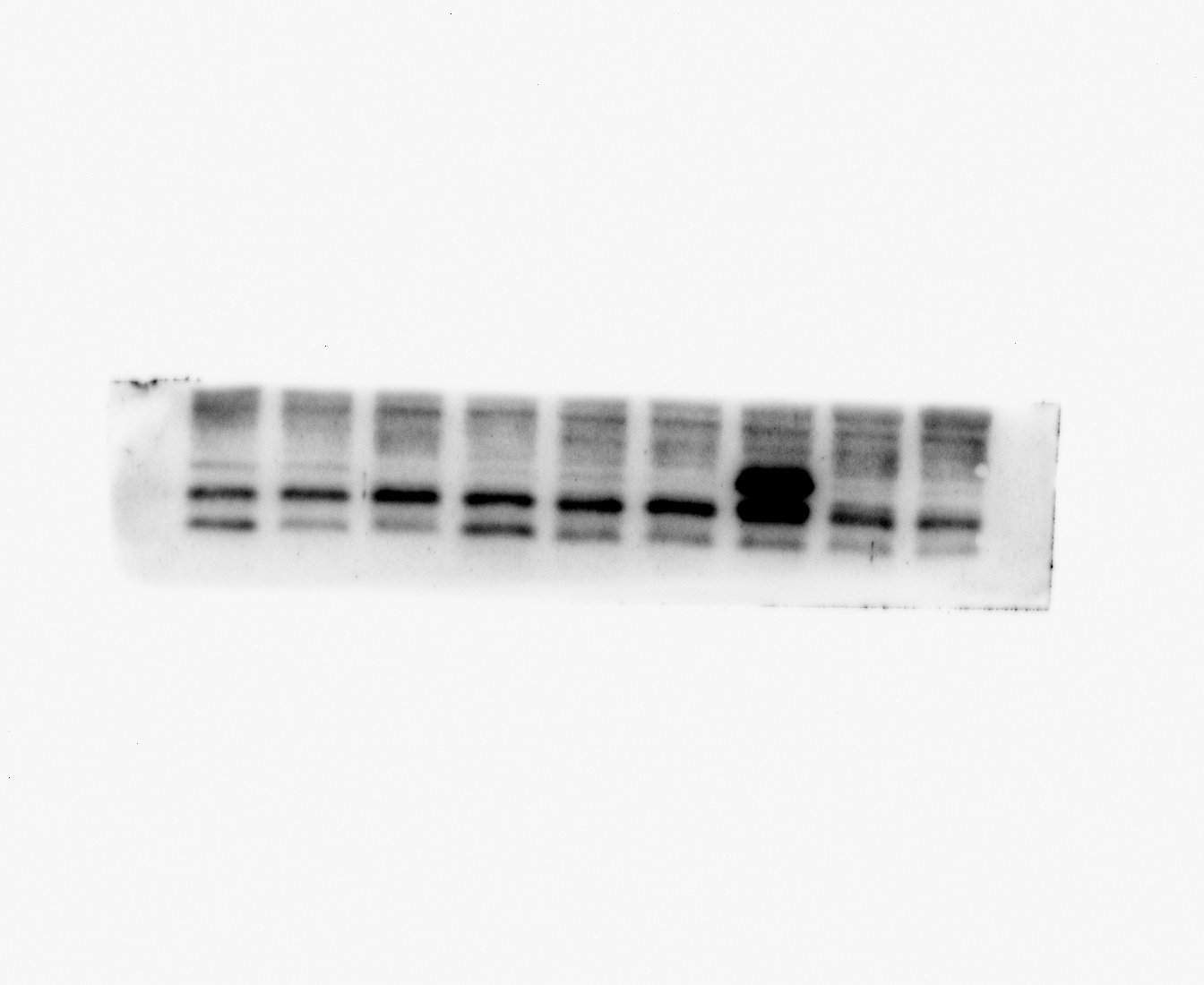

Supplement: Supplementary file 15 — Additional file 15. [file 13020_2026_1383_MOESM15_ESM.tif]

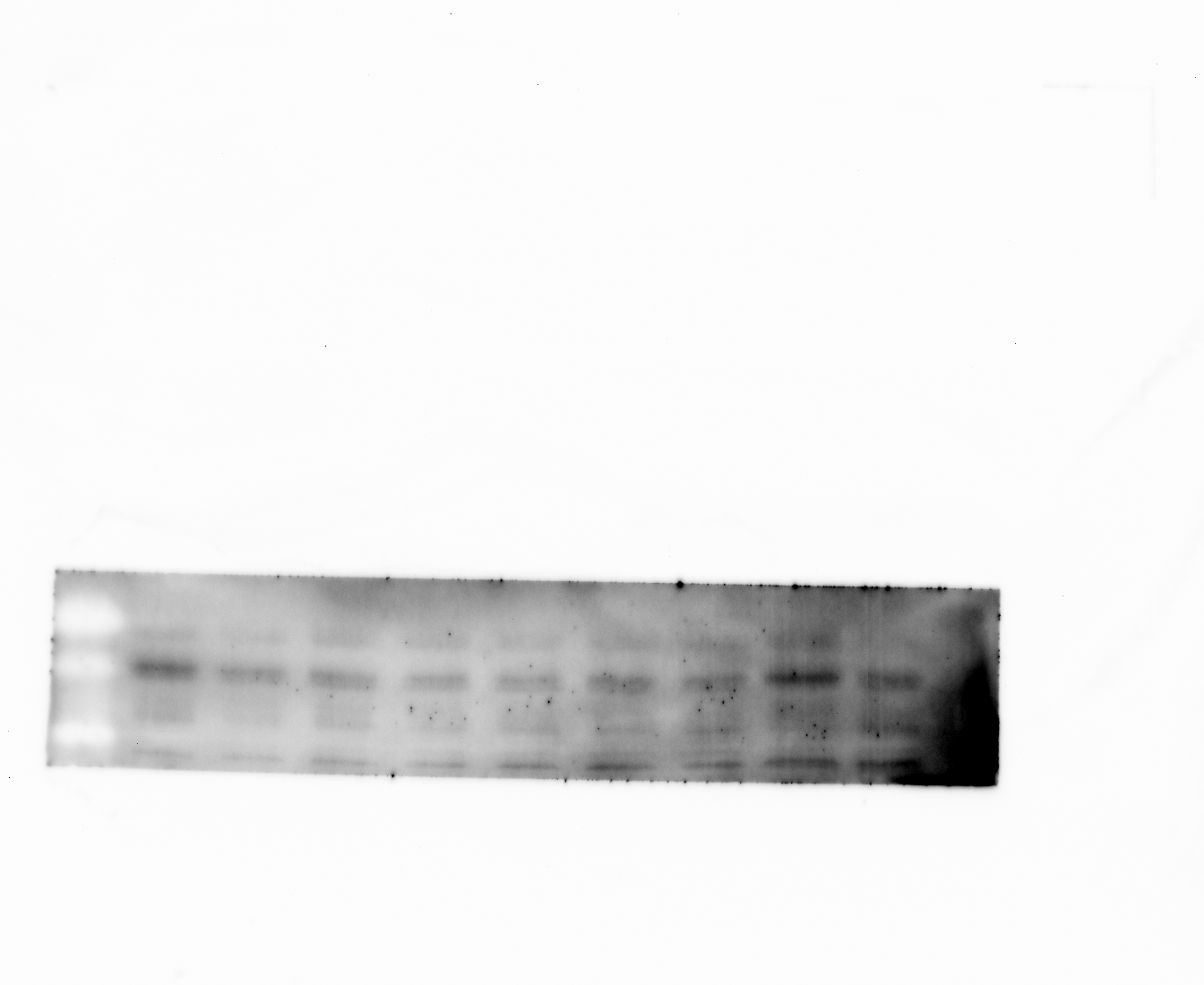

Supplement: Supplementary file 16 — Additional file 16. [file 13020_2026_1383_MOESM16_ESM.tif]

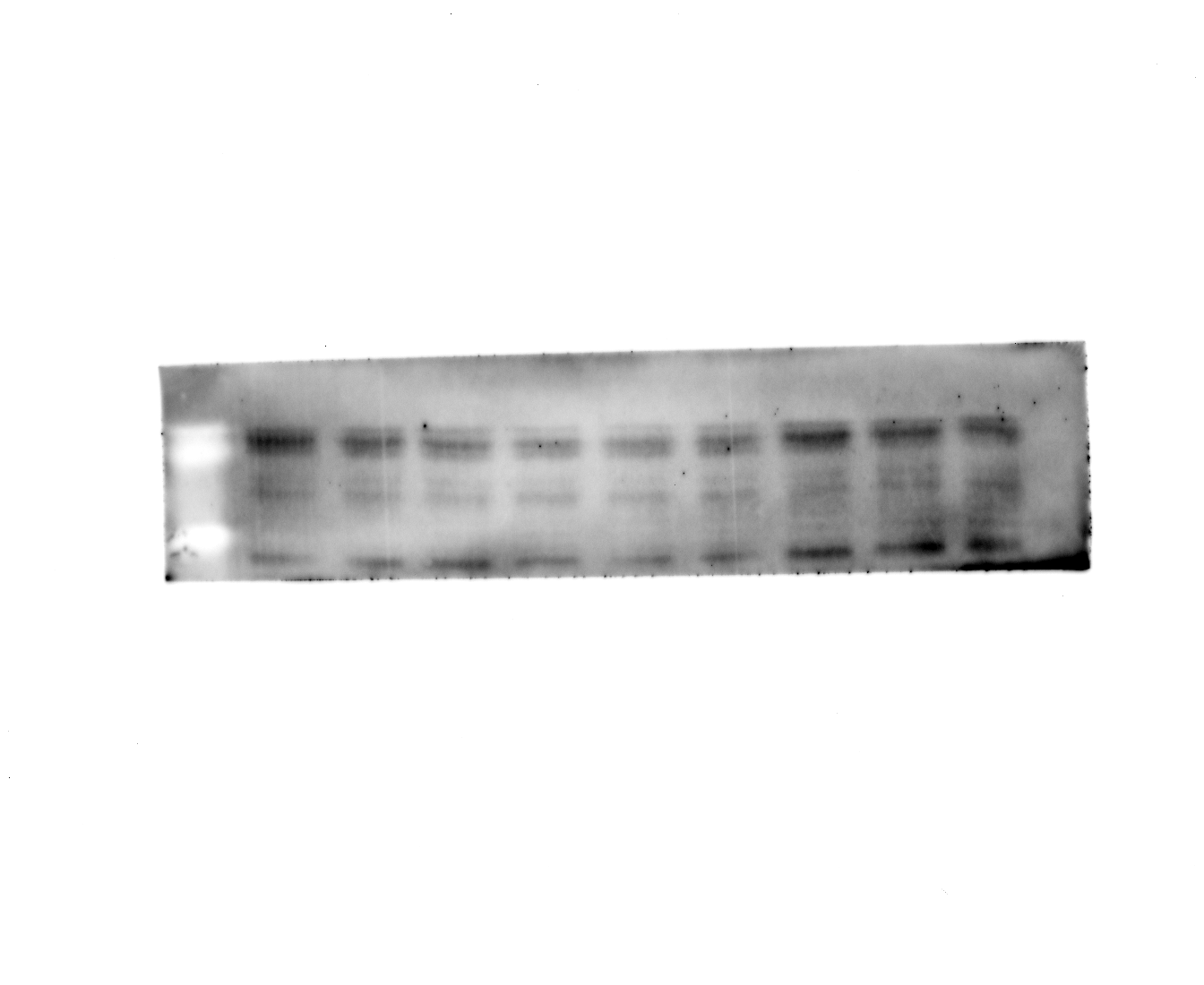

Supplement: Supplementary file 17 — Additional file 17. [file 13020_2026_1383_MOESM17_ESM.tif]

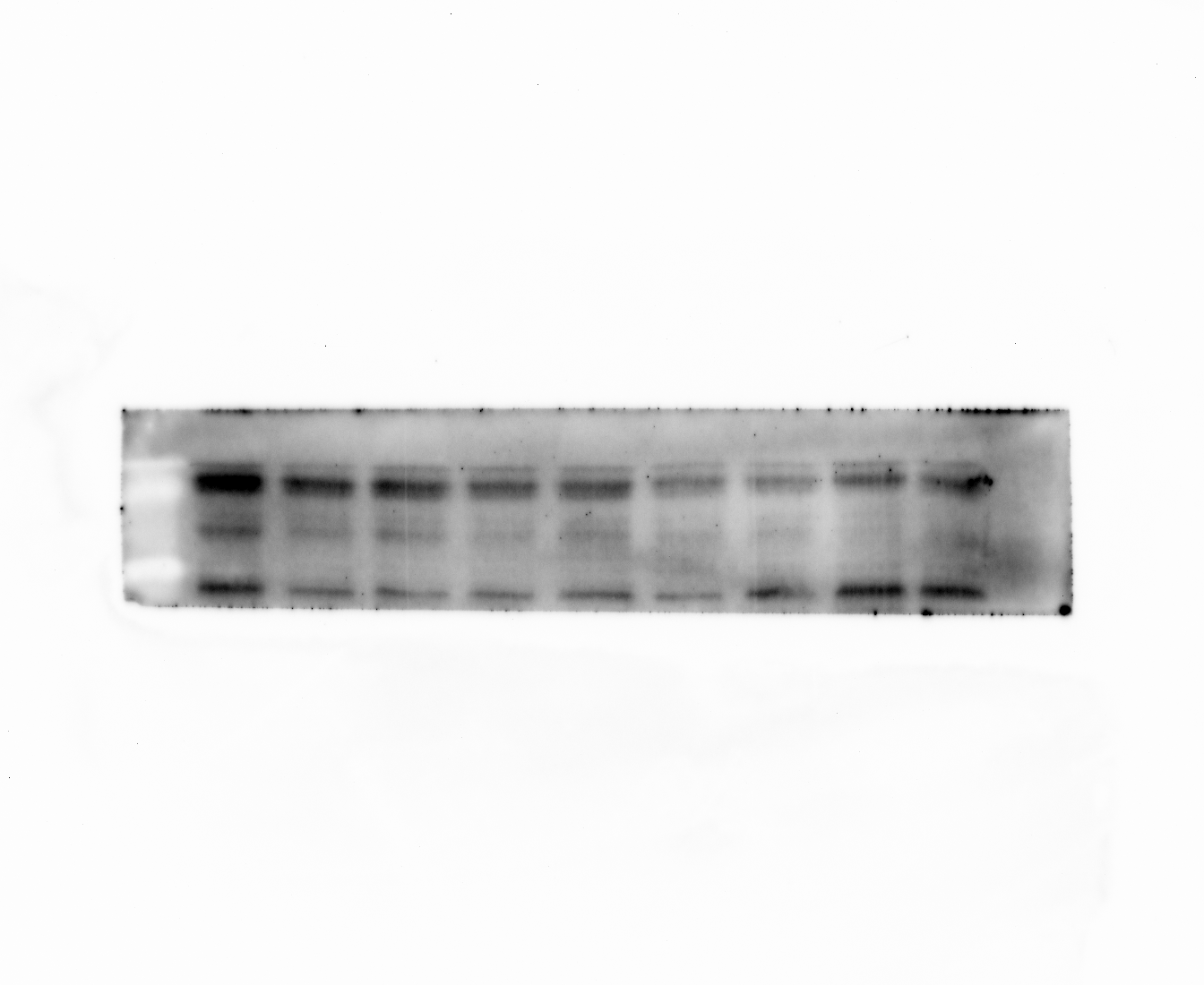

Supplement: Supplementary file 18 — Additional file 18. [file 13020_2026_1383_MOESM18_ESM.tif]

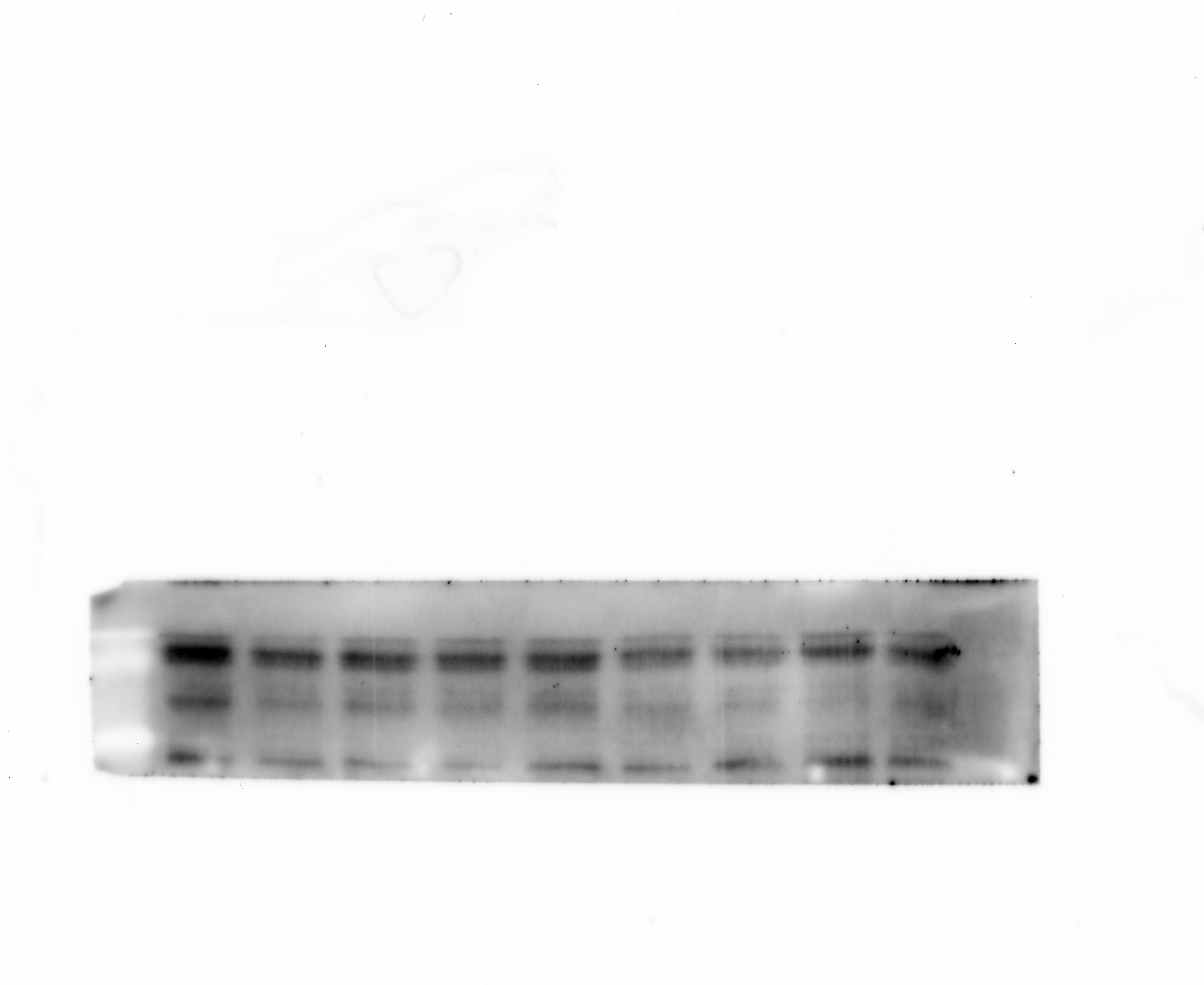

Supplement: Supplementary file 19 — Additional file 19. [file 13020_2026_1383_MOESM19_ESM.tif]

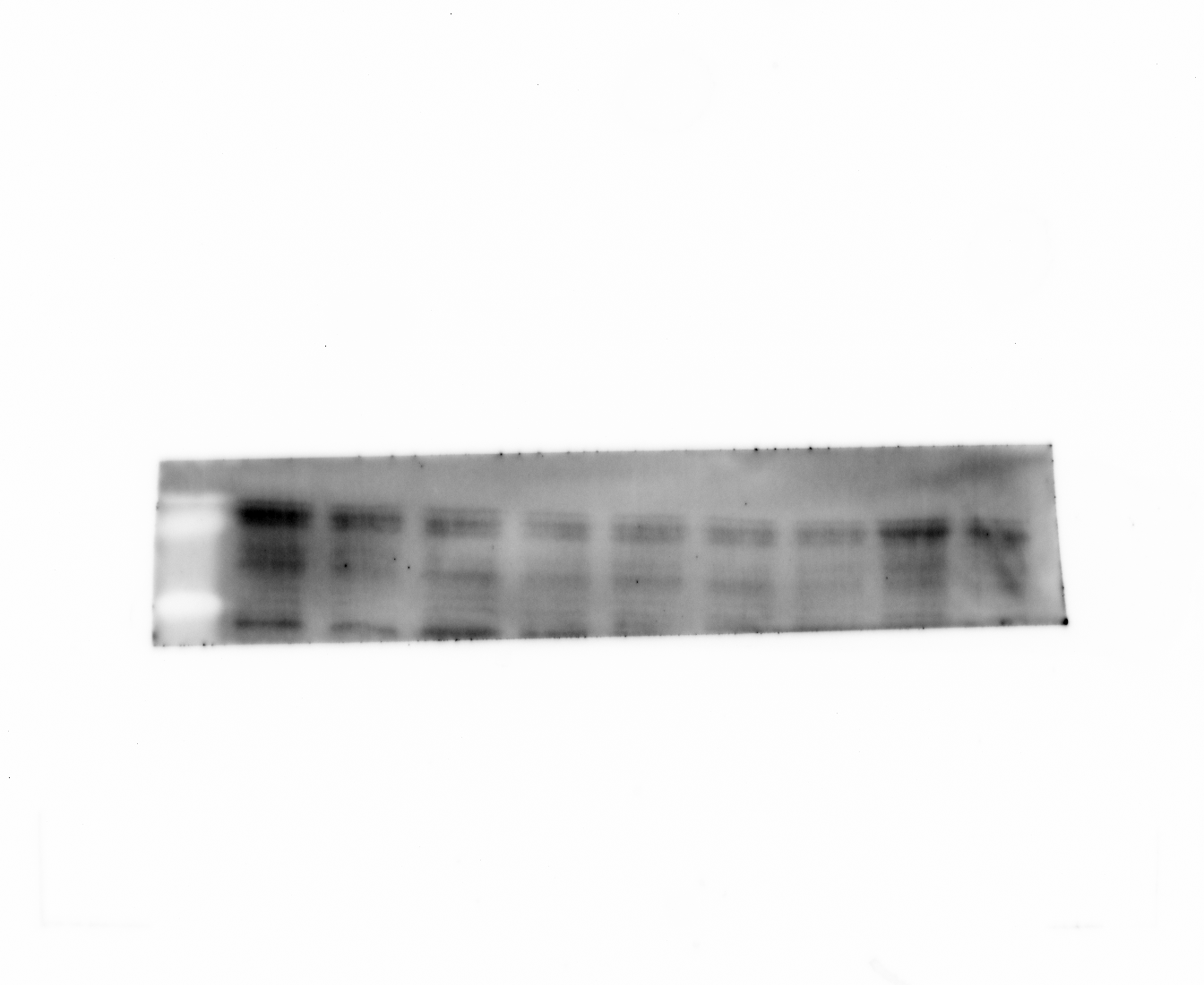

Supplement: Supplementary file 20 — Additional file 20. [file 13020_2026_1383_MOESM20_ESM.tif]

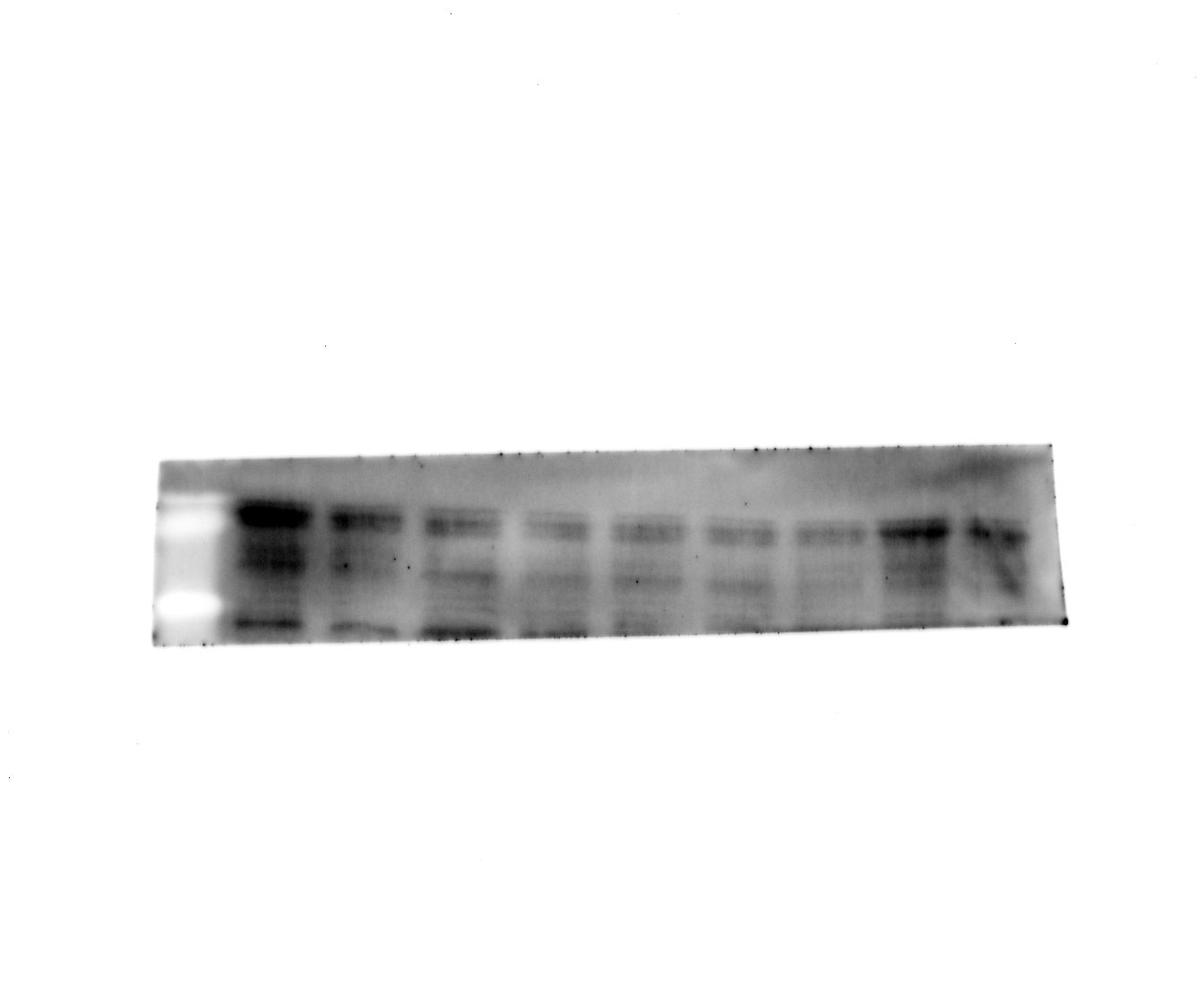

Supplement: Supplementary file 21 — Additional file 21. [file 13020_2026_1383_MOESM21_ESM.tif]

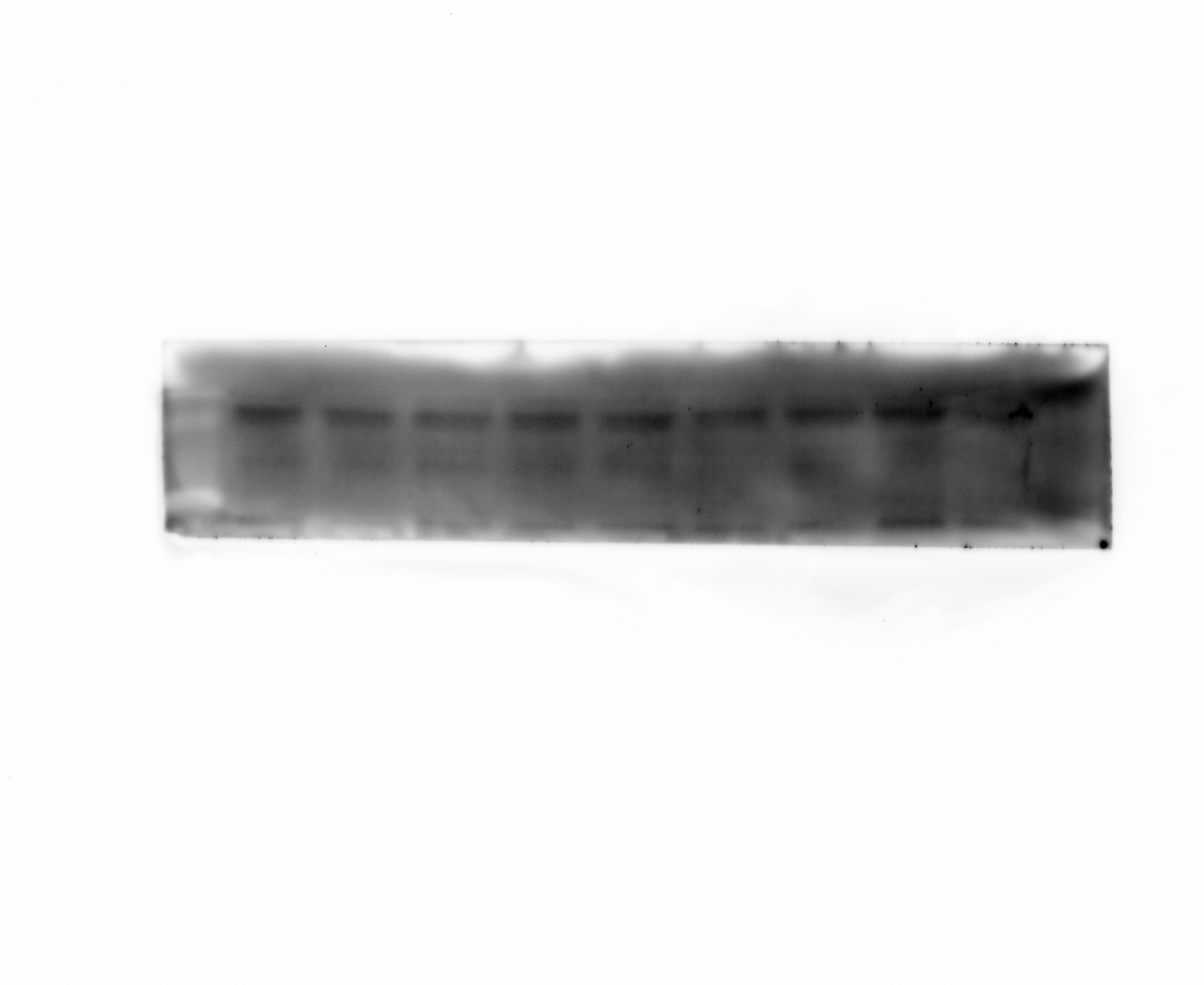

Supplement: Supplementary file 22 — Additional file 22. [file 13020_2026_1383_MOESM22_ESM.tif]

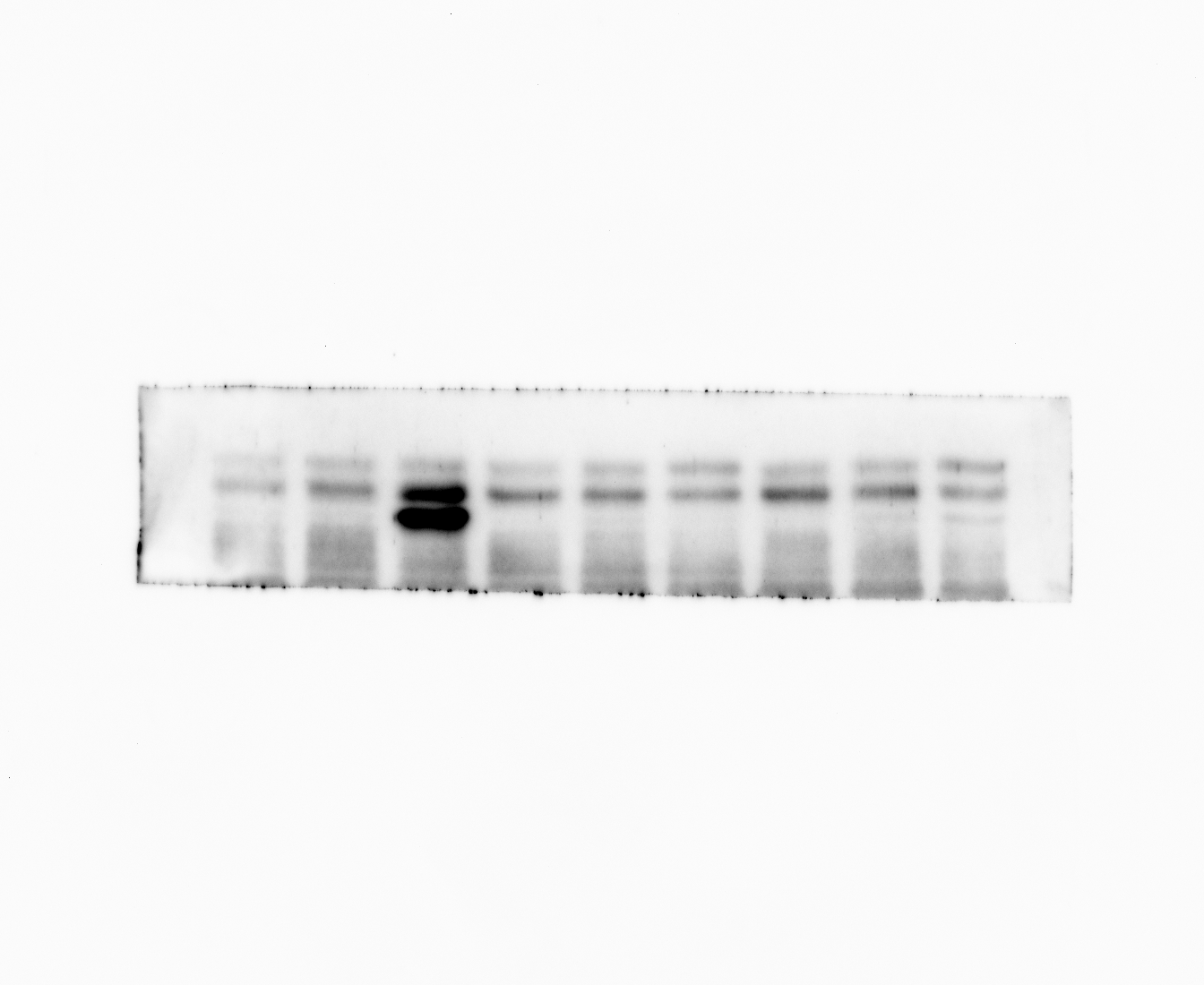

Supplement: Supplementary file 23 — Additional file 23. [file 13020_2026_1383_MOESM23_ESM.tif]

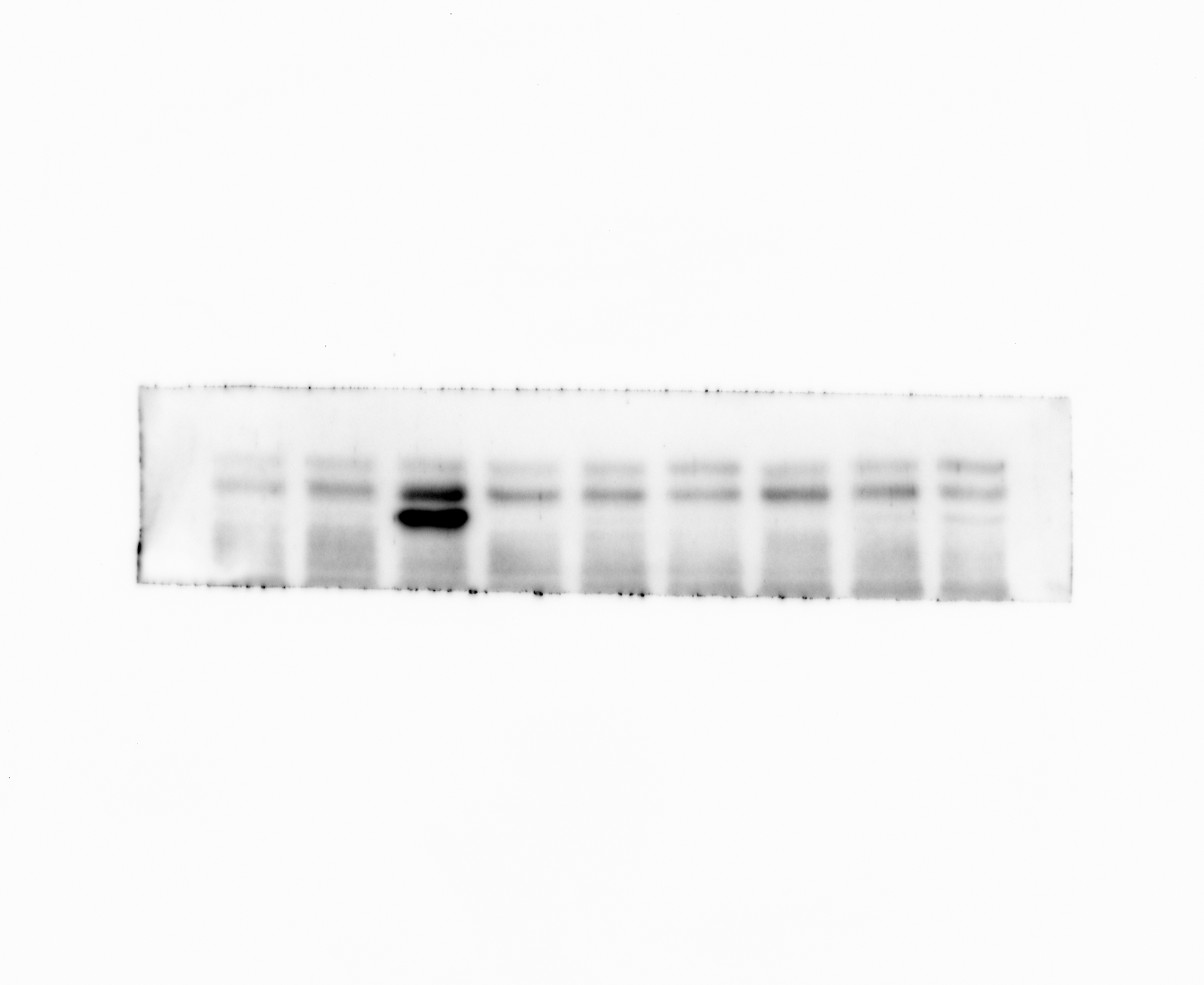

Supplement: Supplementary file 24 — Additional file 24. [file 13020_2026_1383_MOESM24_ESM.tif]

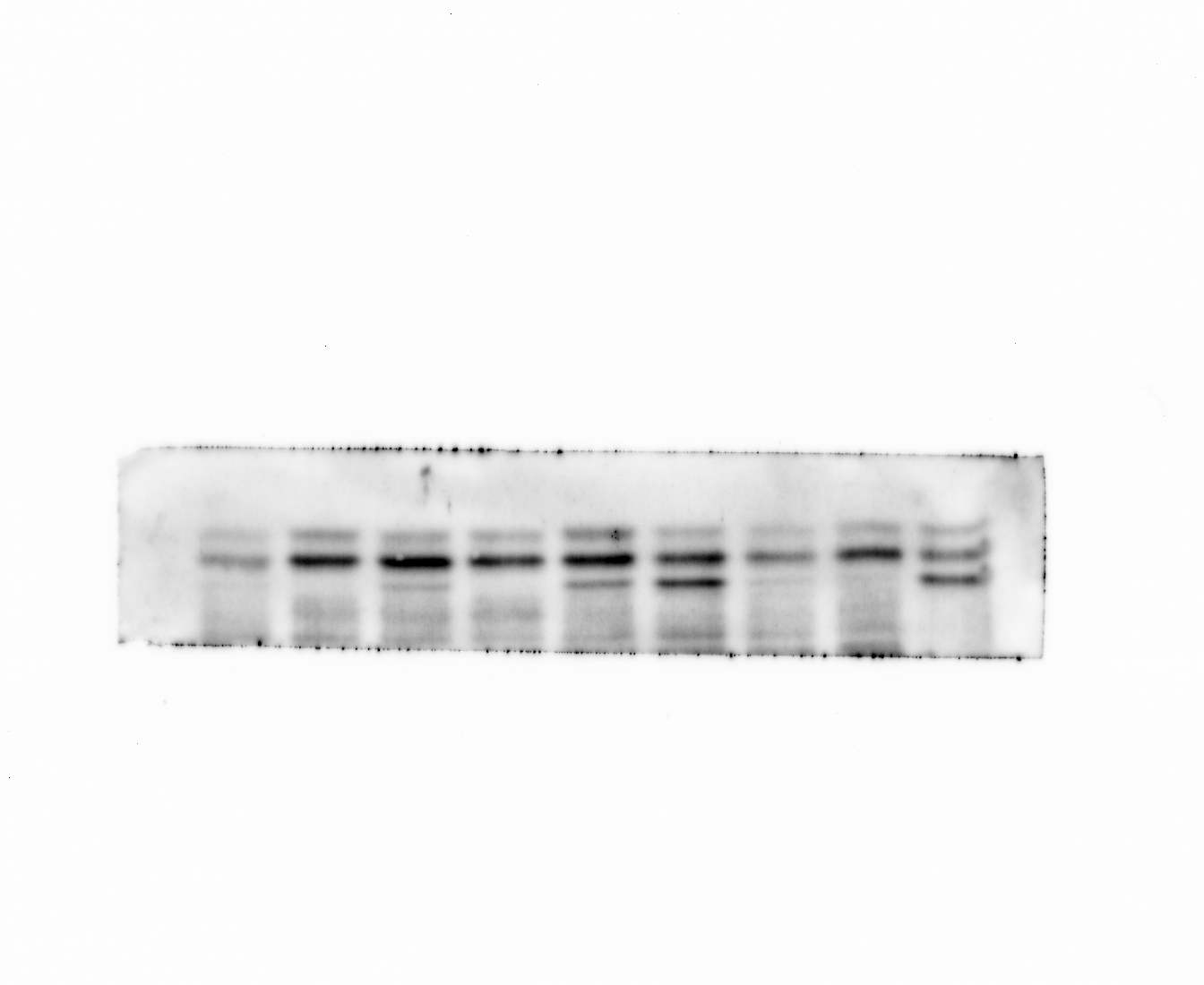

Supplement: Supplementary file 25 — Additional file 25. [file 13020_2026_1383_MOESM25_ESM.tif]

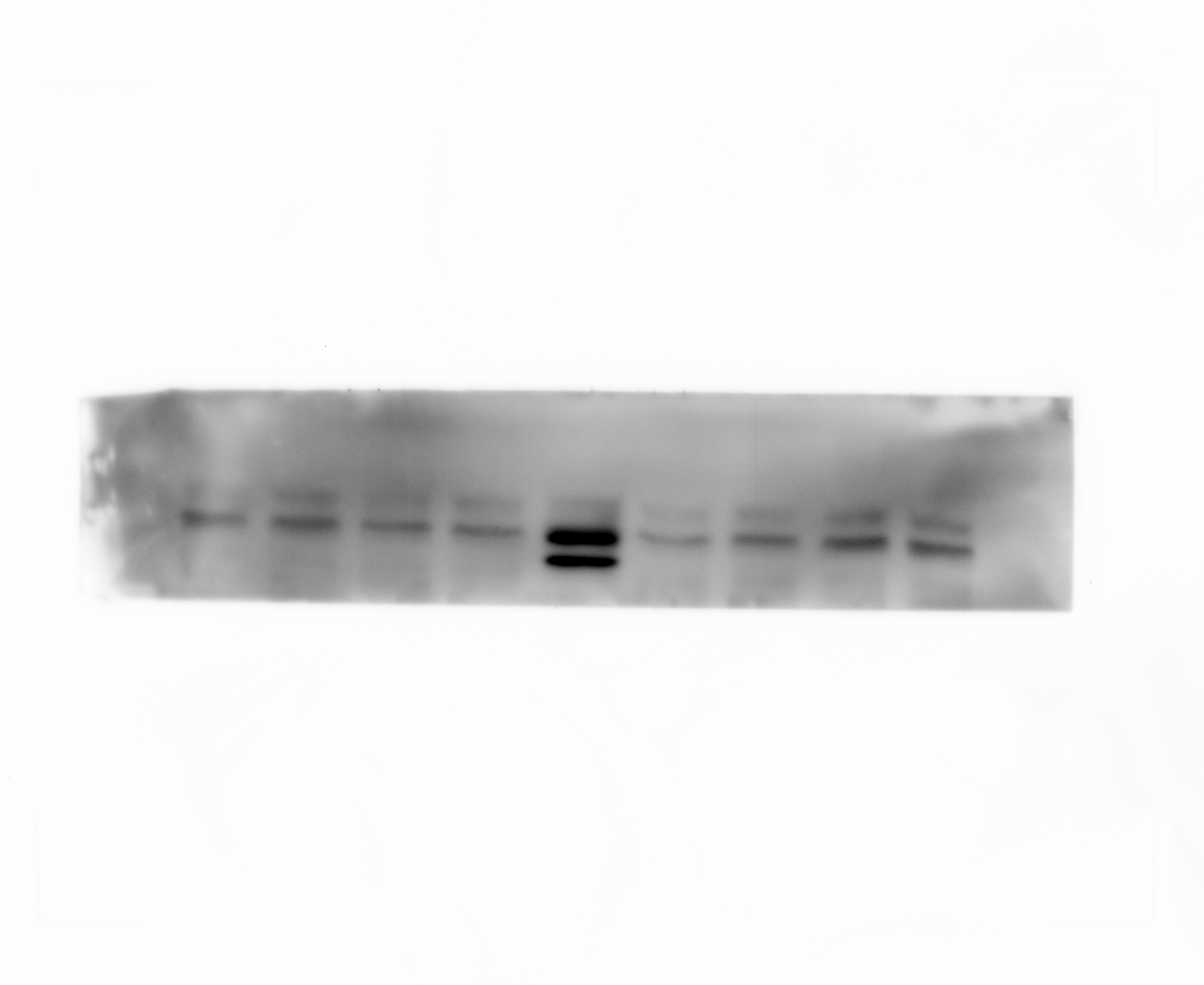

Supplement: Supplementary file 26 — Additional file 26. [file 13020_2026_1383_MOESM26_ESM.tif]

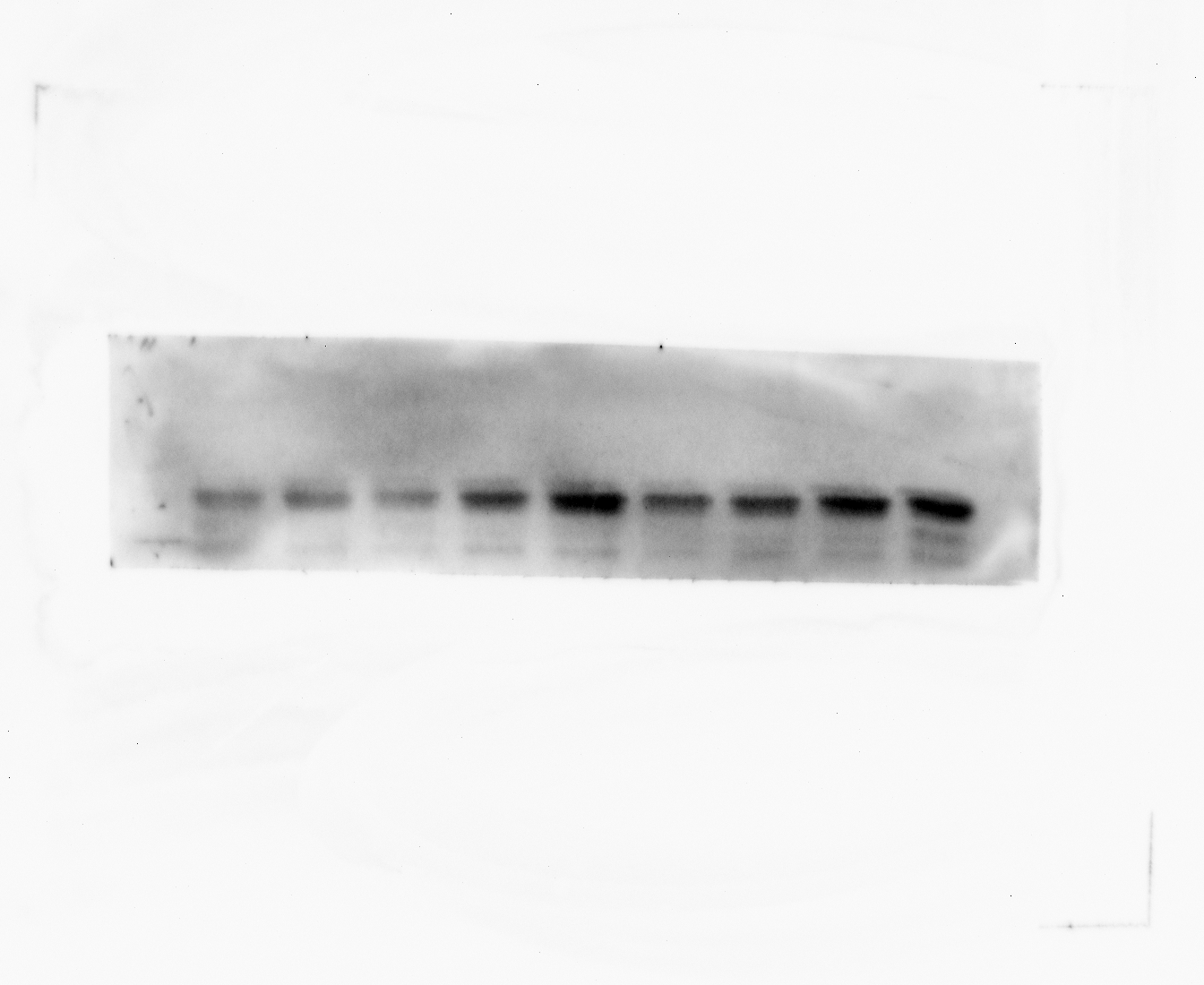

Supplement: Supplementary file 27 — Additional file 27. [file 13020_2026_1383_MOESM27_ESM.tif]

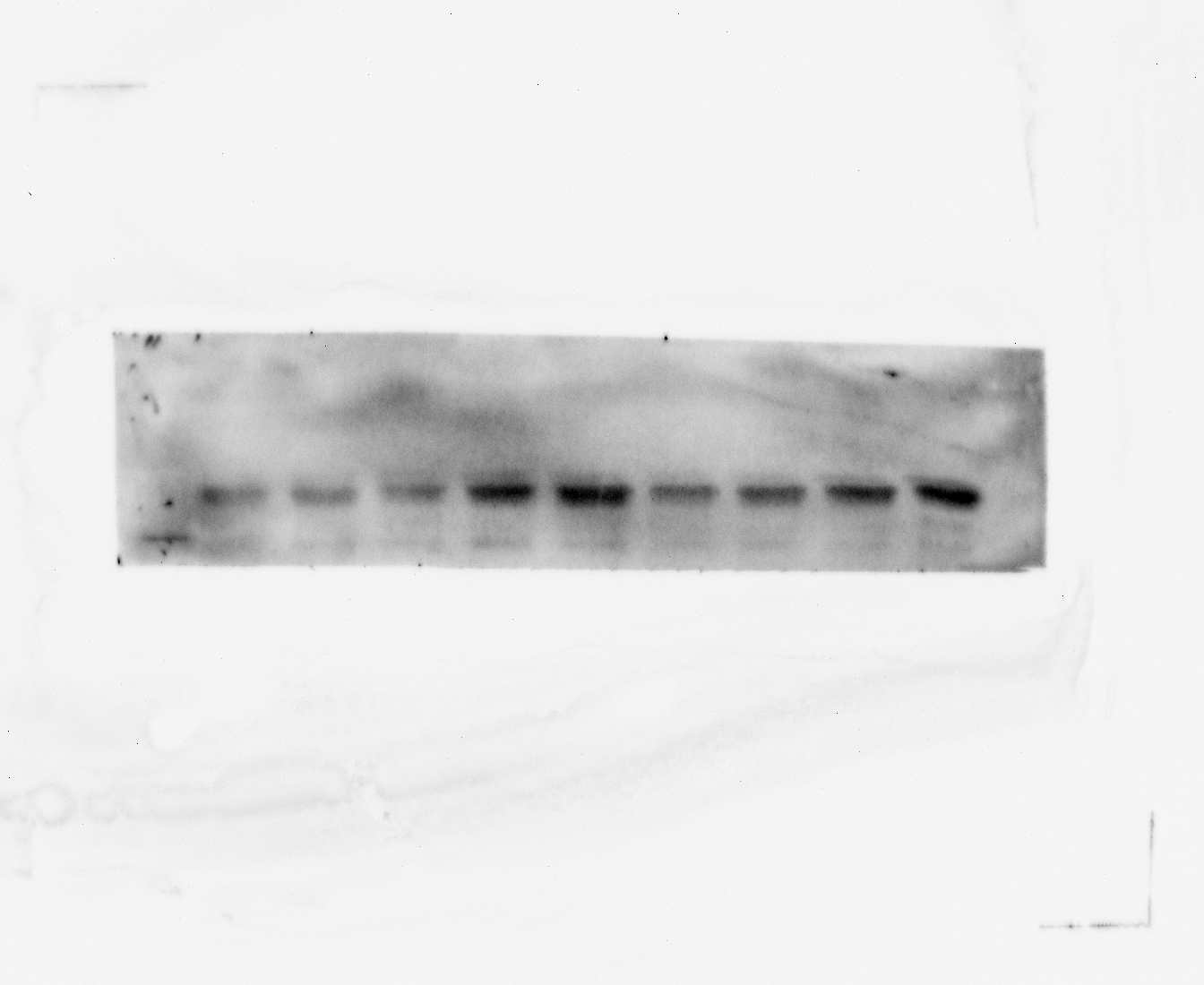

Supplement: Supplementary file 28 — Additional file 28. [file 13020_2026_1383_MOESM28_ESM.tif]

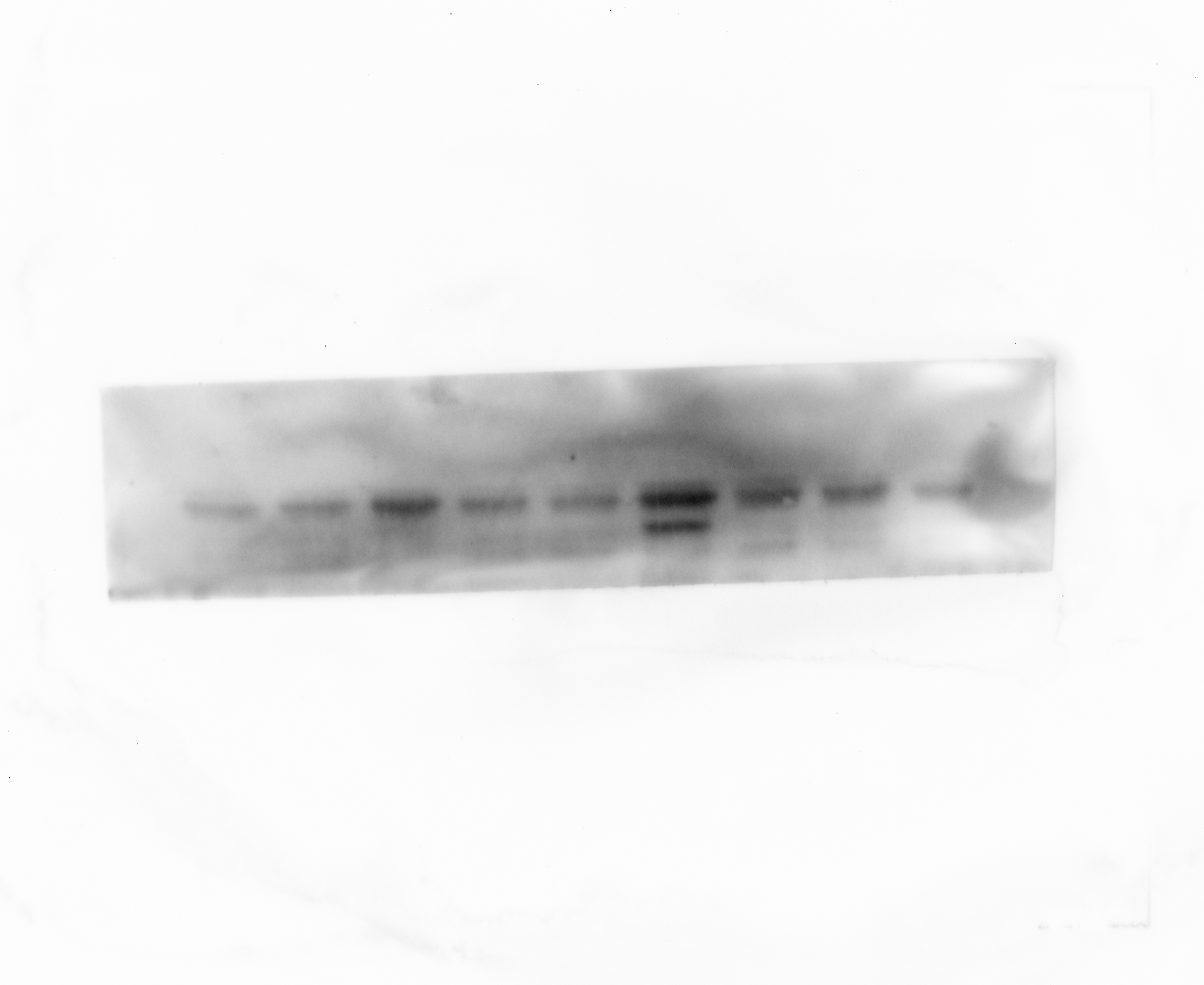

Supplement: Supplementary file 29 — Additional file 29. [file 13020_2026_1383_MOESM29_ESM.tif]

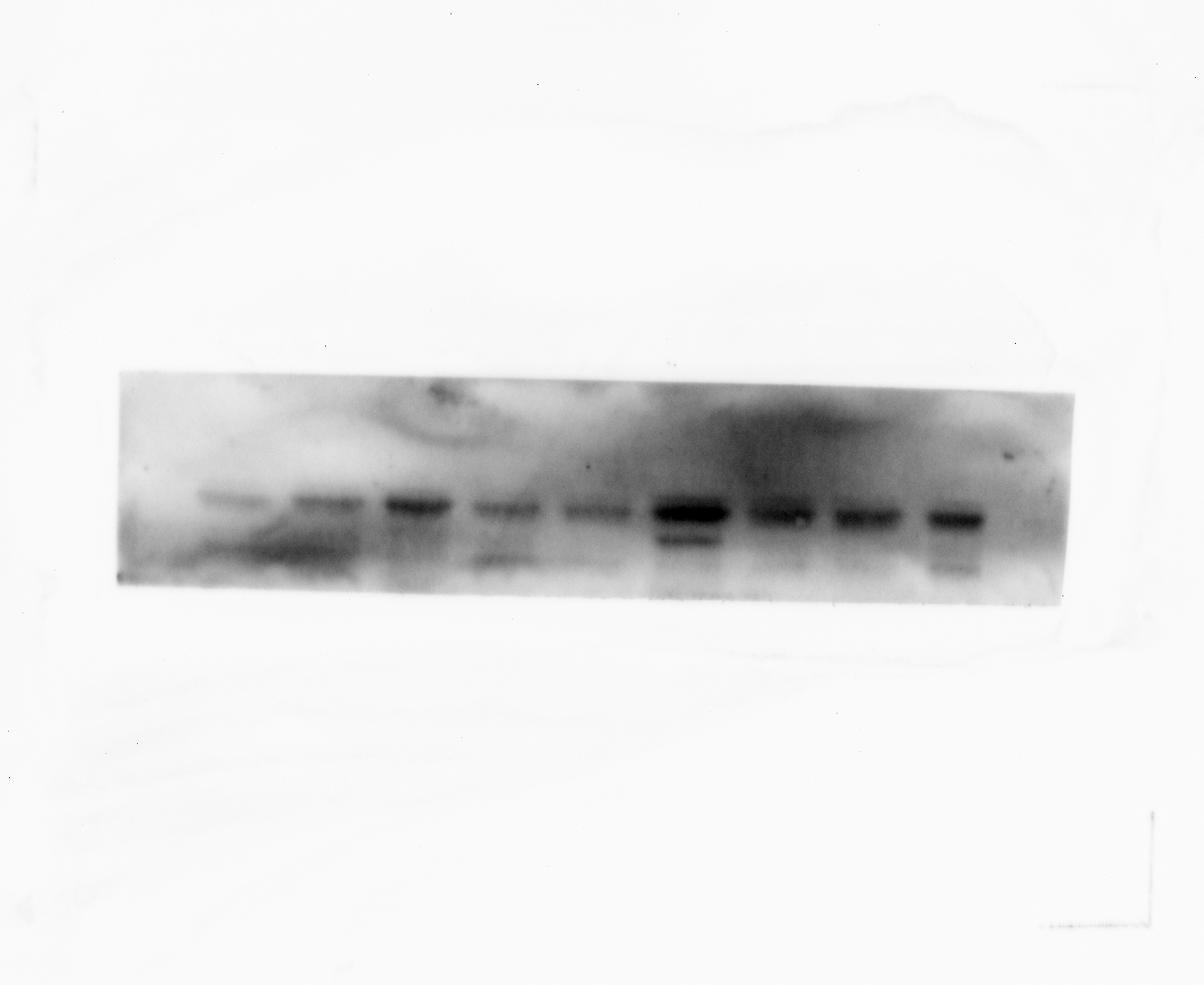

Supplement: Supplementary file 30 — Additional file 30. [file 13020_2026_1383_MOESM30_ESM.tif]

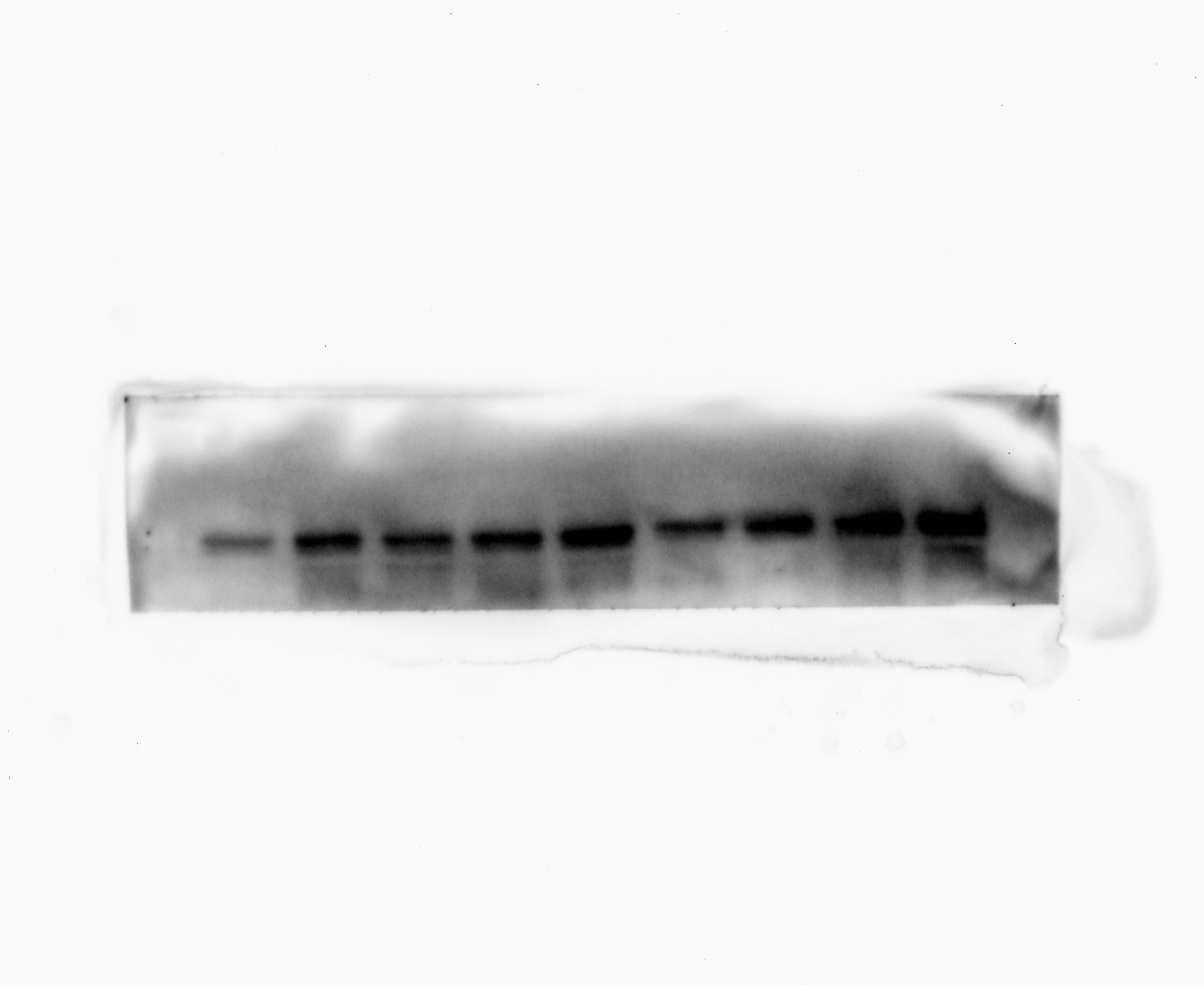

Supplement: Supplementary file 31 — Additional file 31. [file 13020_2026_1383_MOESM31_ESM.tif]

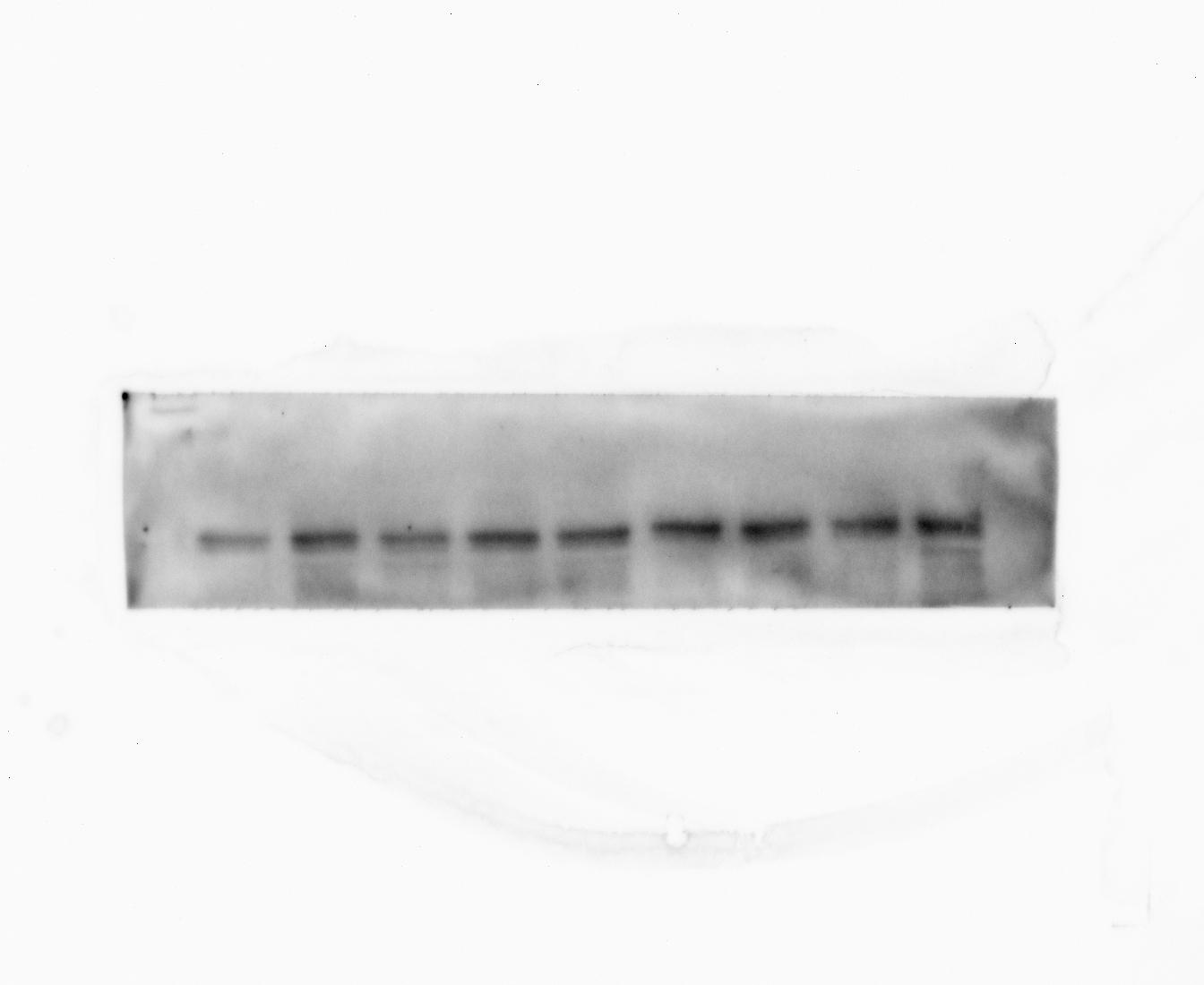

Supplement: Supplementary file 32 — Additional file 32. [file 13020_2026_1383_MOESM32_ESM.tif]

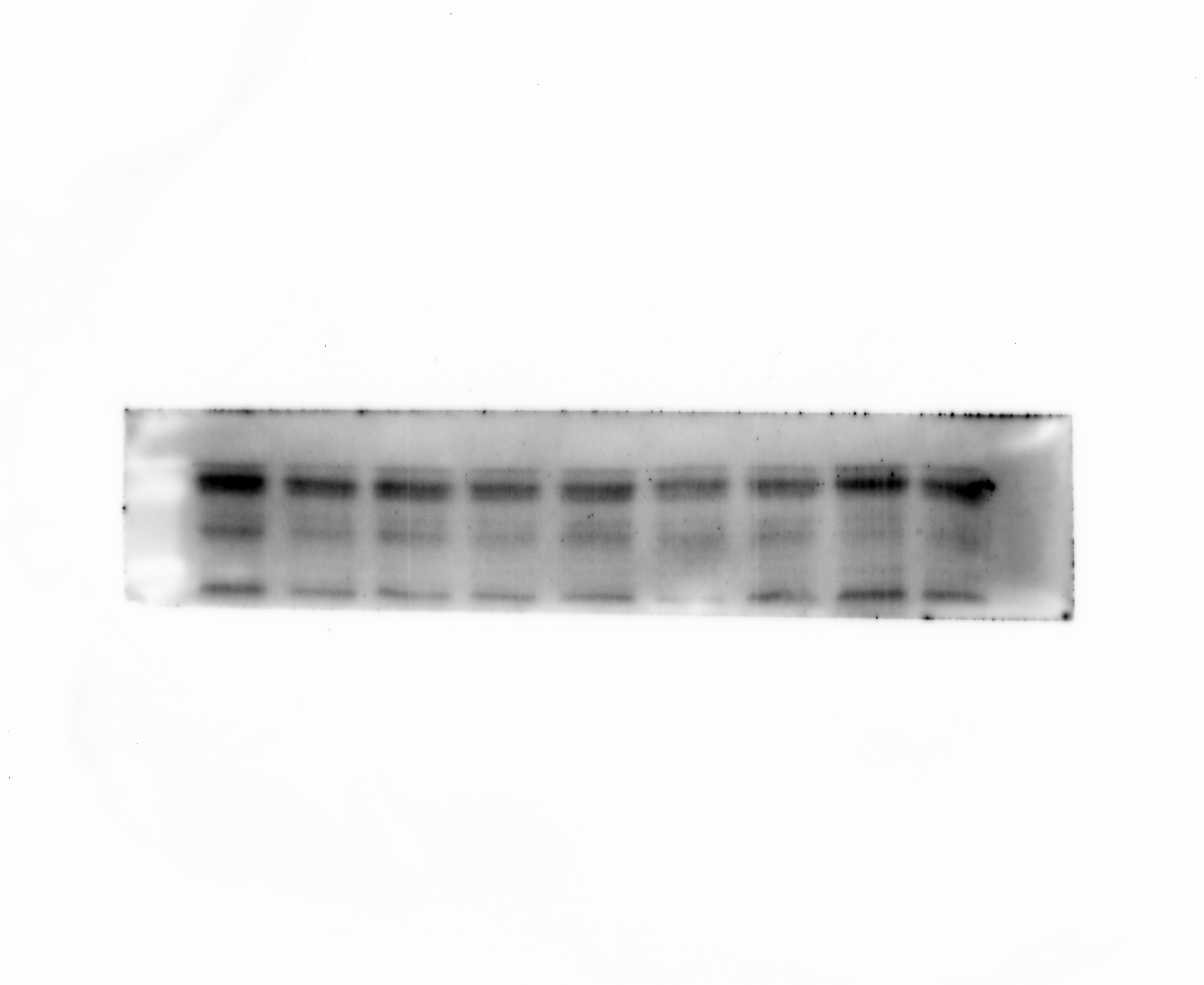

Supplement: Supplementary file 33 — Additional file 33. [file 13020_2026_1383_MOESM33_ESM.tif]

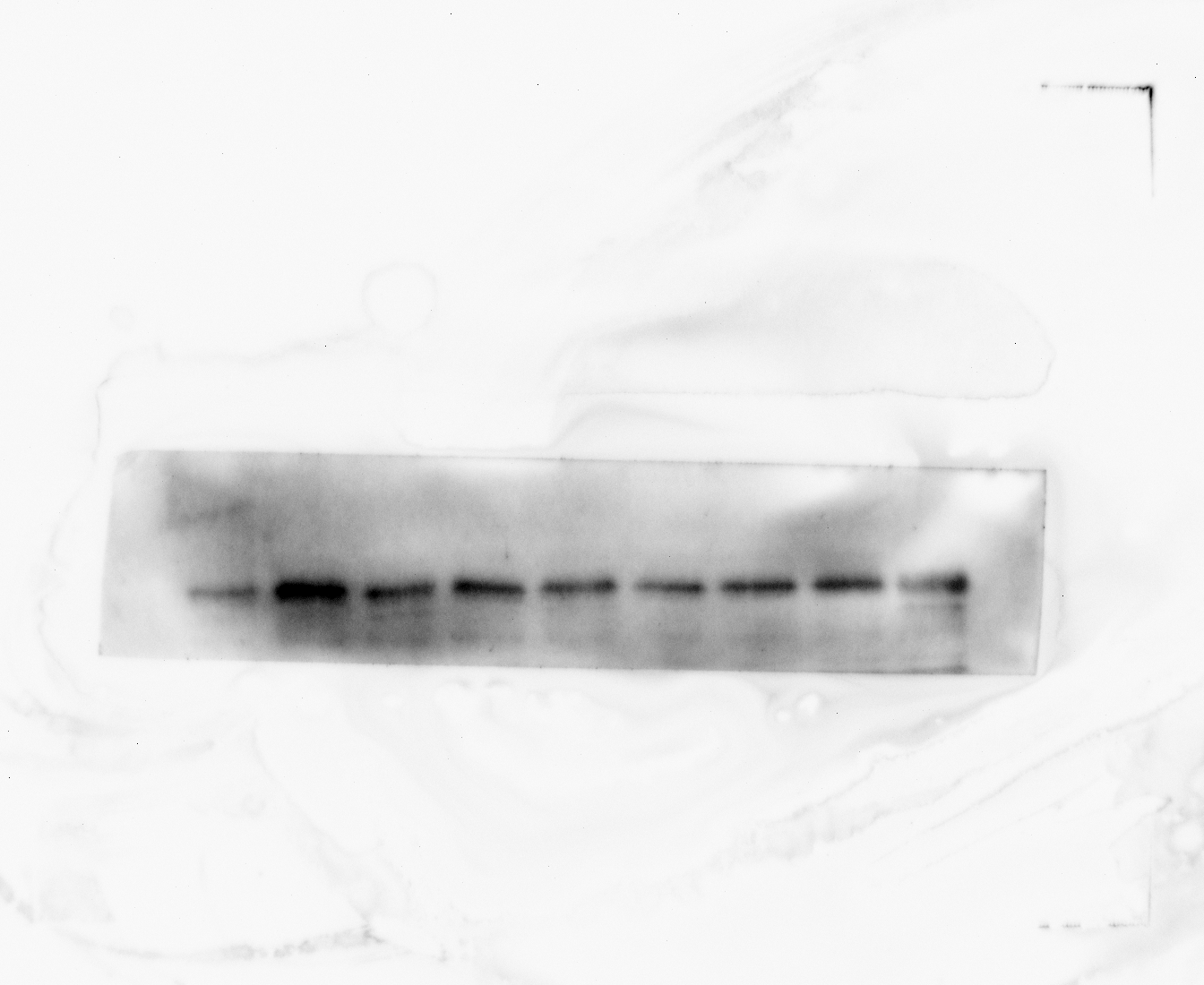

Supplement: Supplementary file 34 — Additional file 34. [file 13020_2026_1383_MOESM34_ESM.tif]

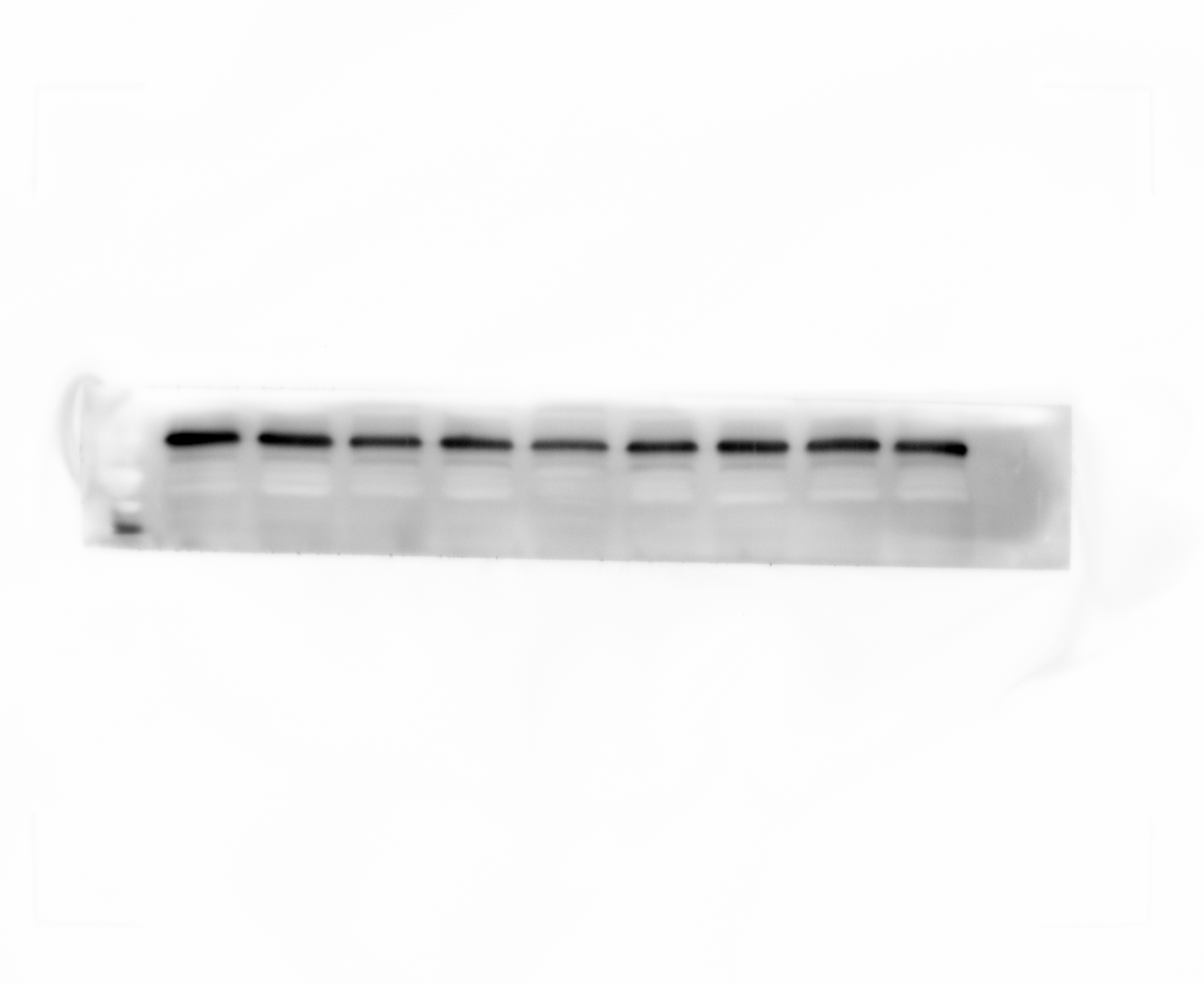

Supplement: Supplementary file 35 — Additional file 35. [file 13020_2026_1383_MOESM35_ESM.tif]

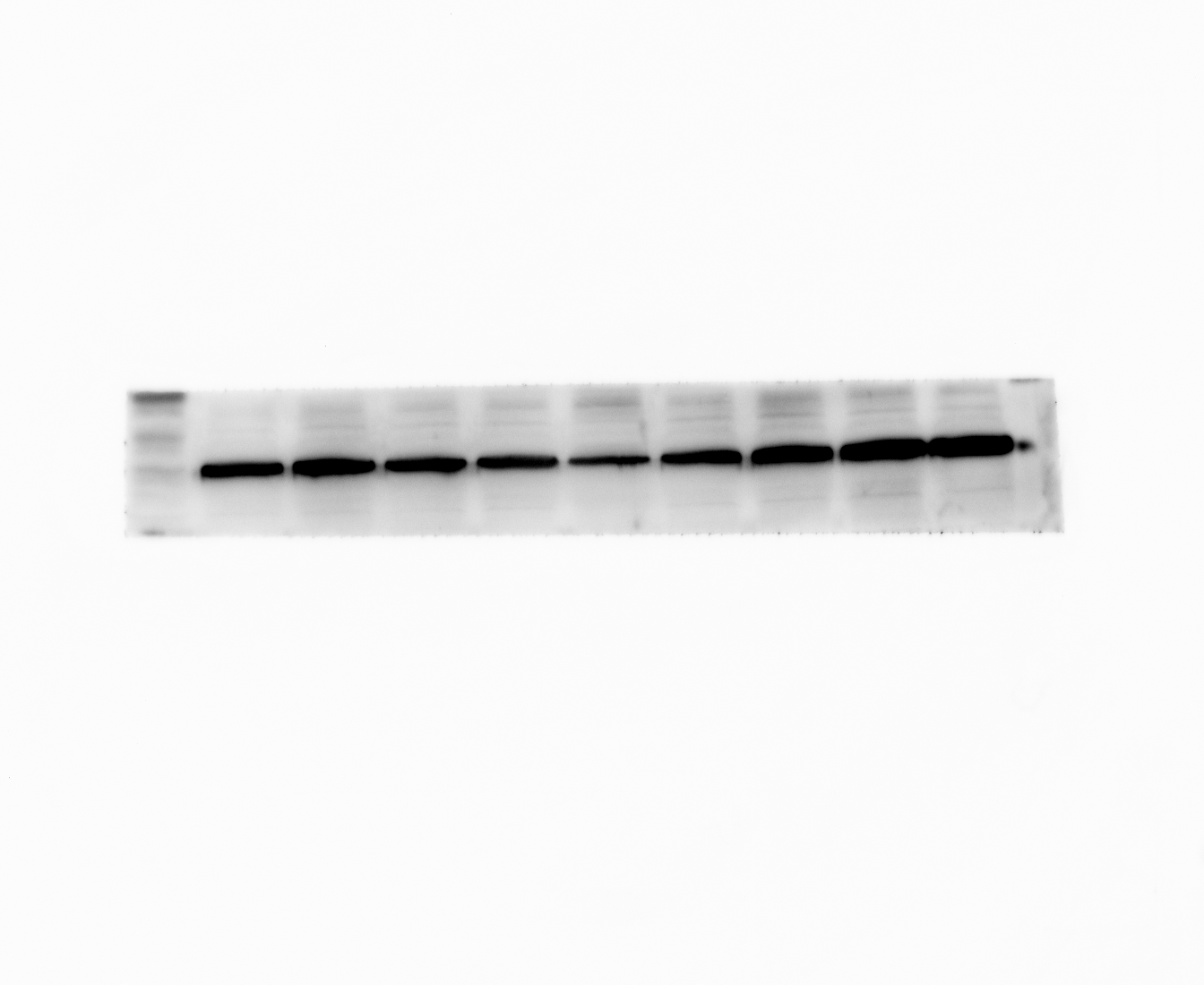

Supplement: Supplementary file 36 — Additional file 36. [file 13020_2026_1383_MOESM36_ESM.tif]

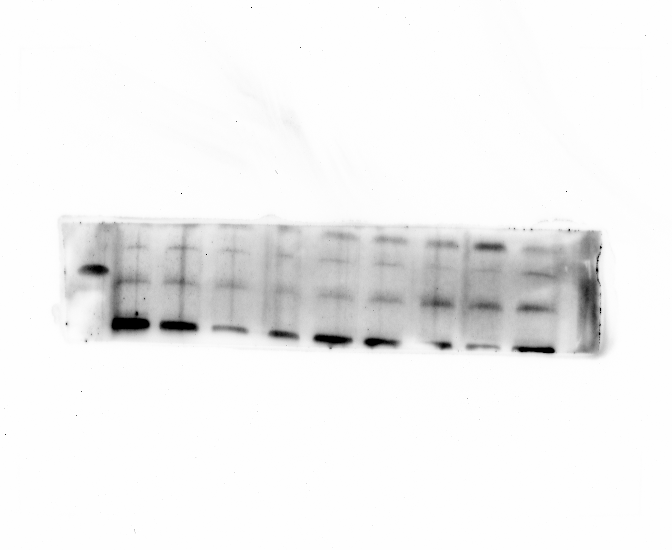

Supplement: Supplementary file 37 — Additional file 37. [file 13020_2026_1383_MOESM37_ESM.tif]

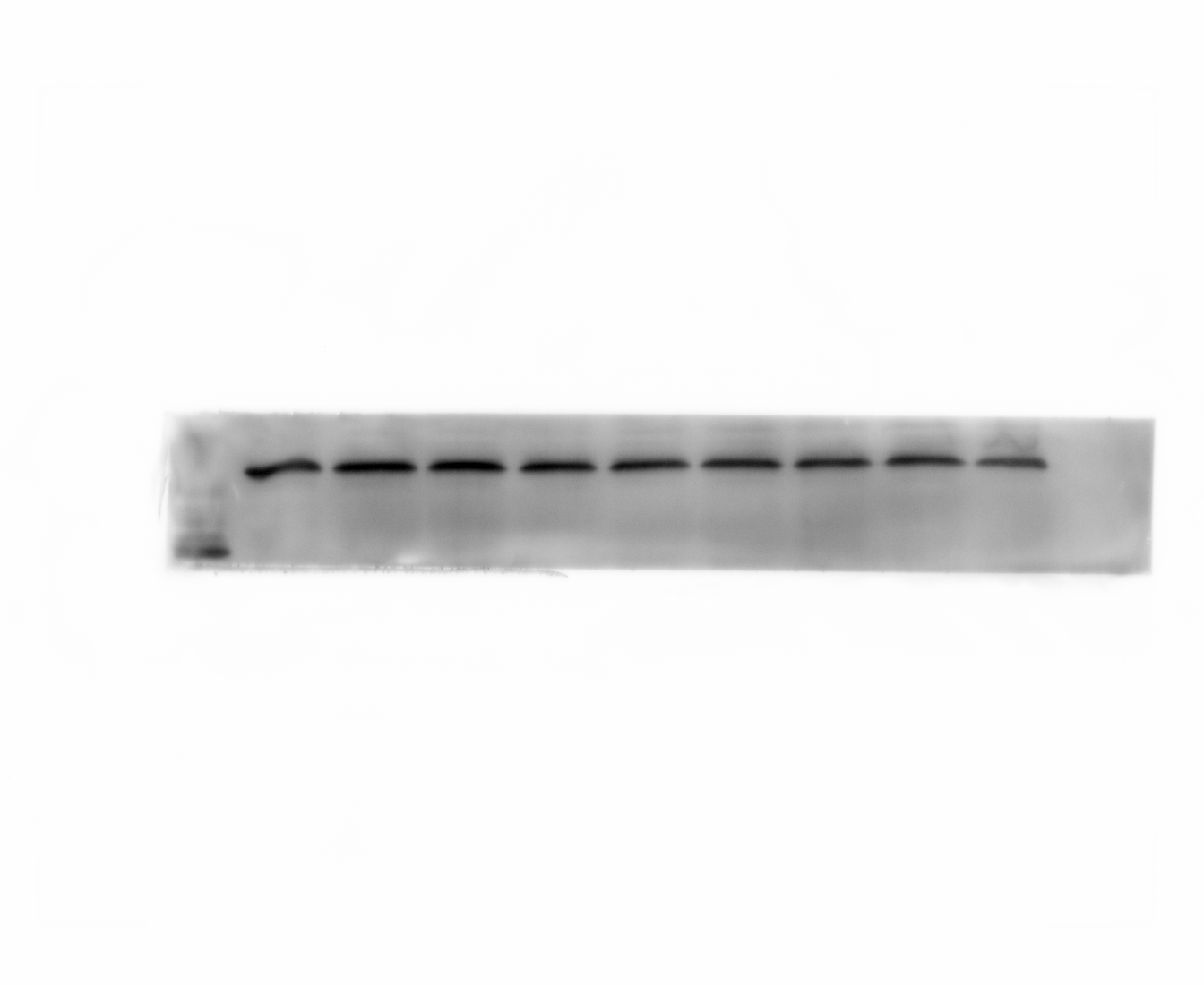

Supplement: Supplementary file 38 — Additional file 38. [file 13020_2026_1383_MOESM38_ESM.tif]

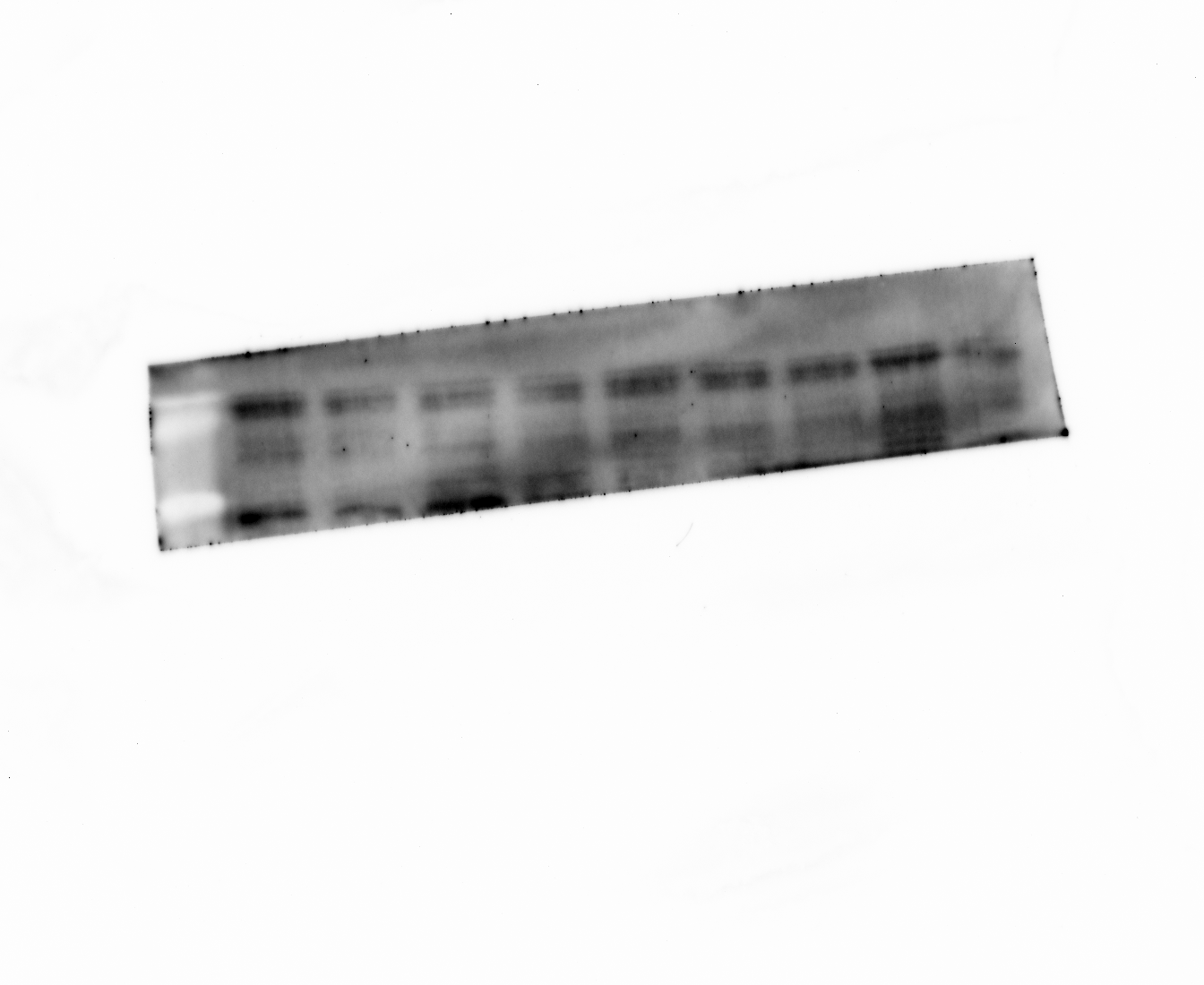

Supplement: Supplementary file 39 — Additional file 39. [file 13020_2026_1383_MOESM39_ESM.tif]

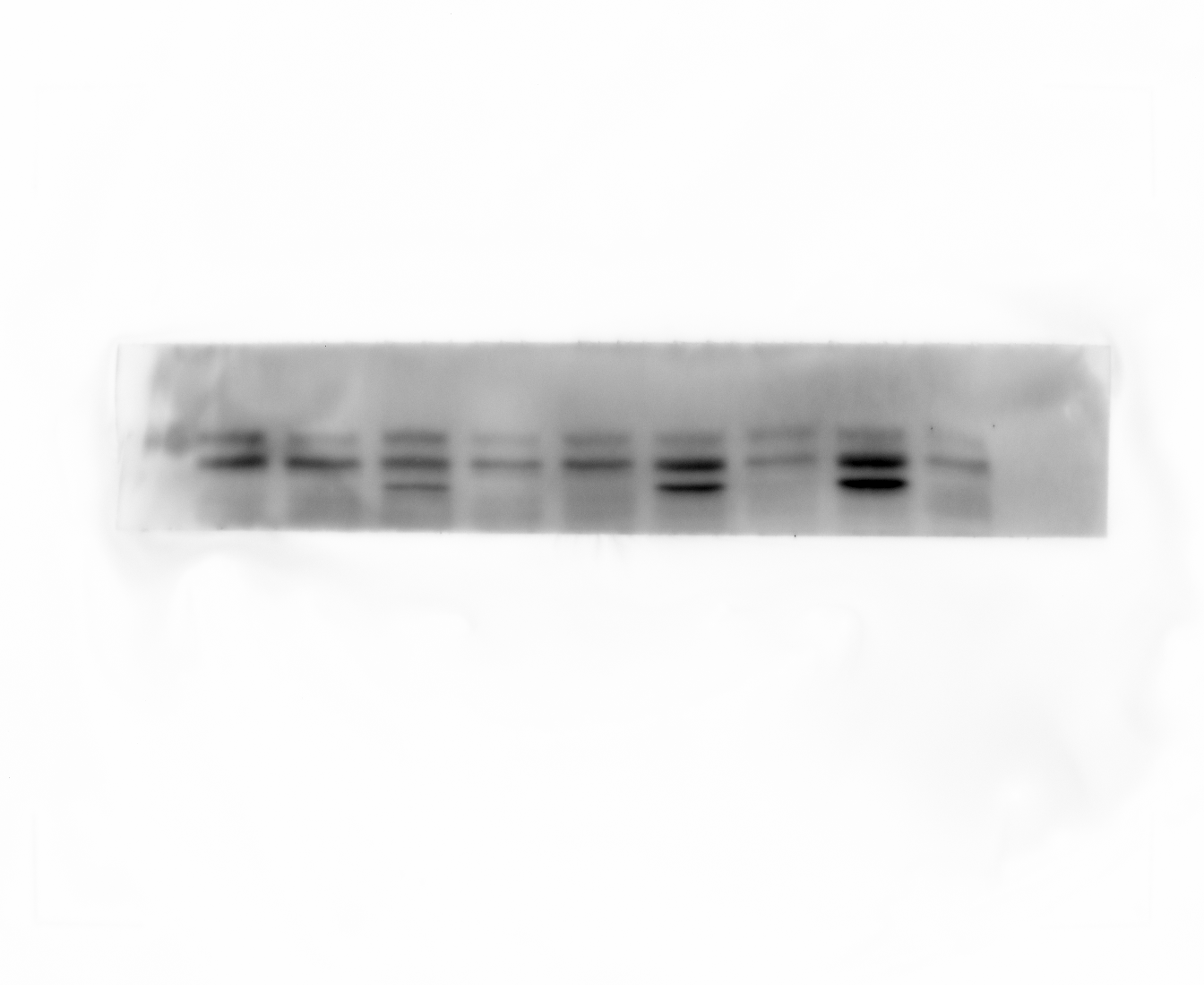

Supplement: Supplementary file 40 — Additional file 40. [file 13020_2026_1383_MOESM40_ESM.tif]

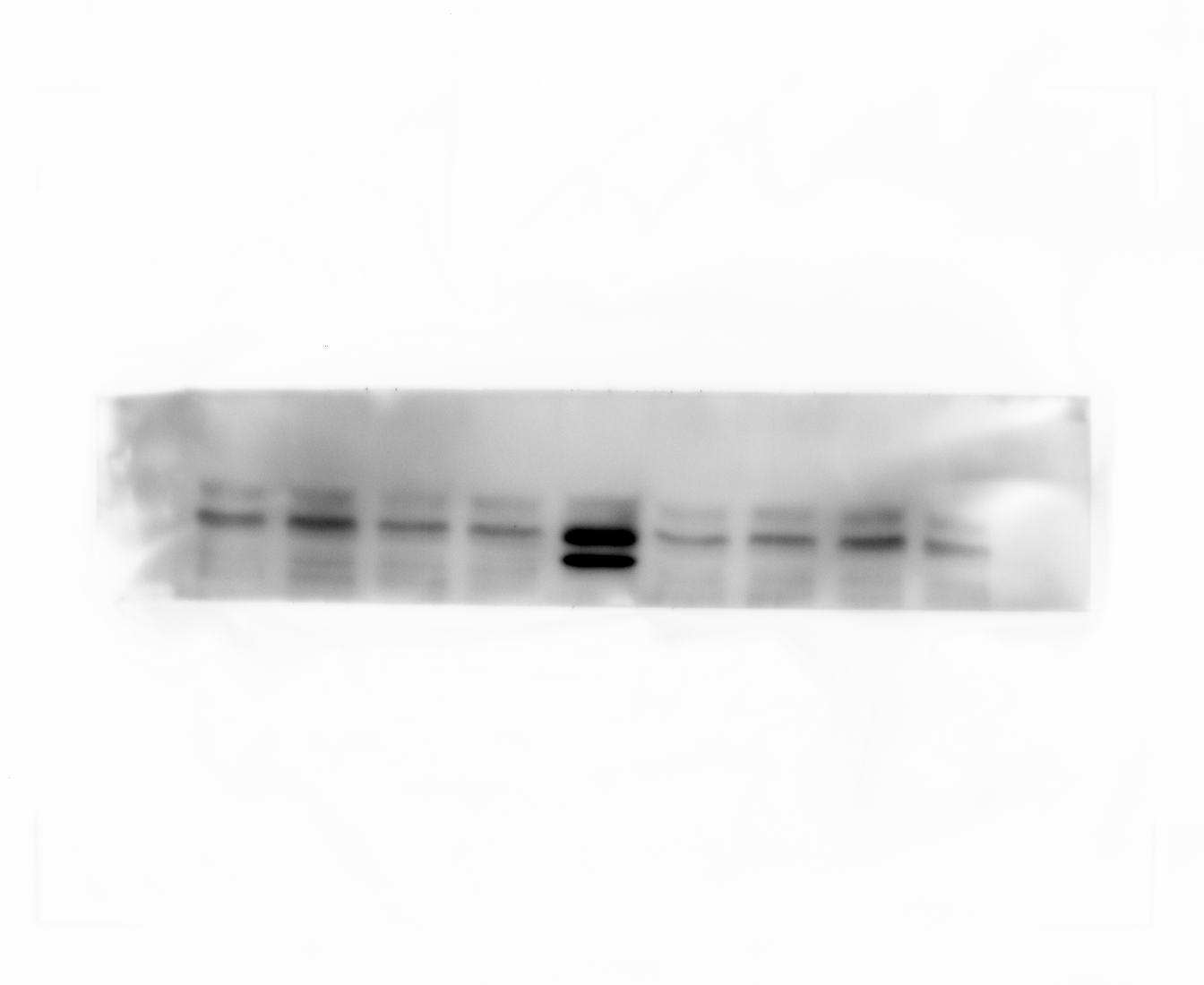

Supplement: Supplementary file 41 — Additional file 41. [file 13020_2026_1383_MOESM41_ESM.tif]

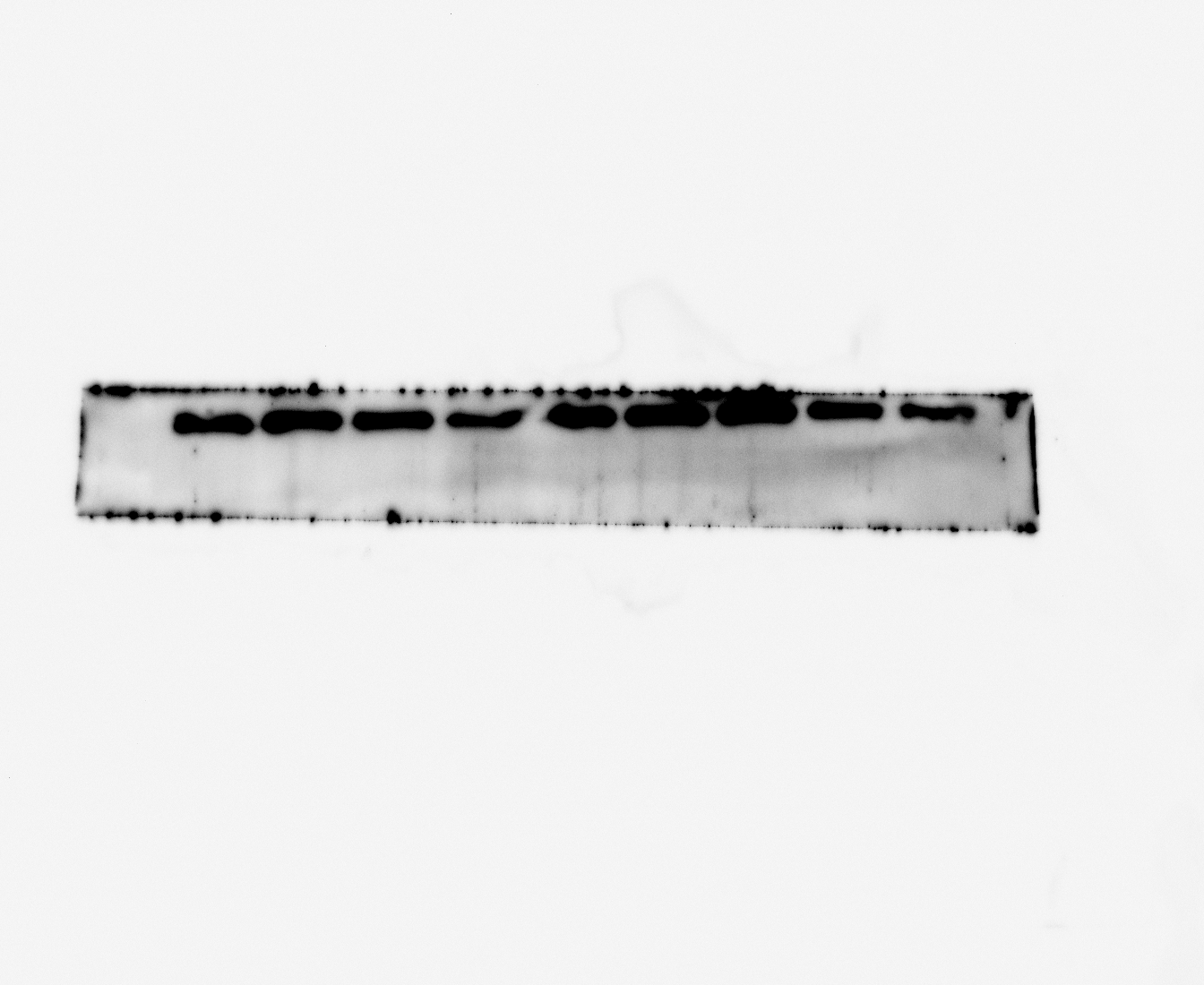

Supplement: Supplementary file 42 — Additional file 42. [file 13020_2026_1383_MOESM42_ESM.tif]

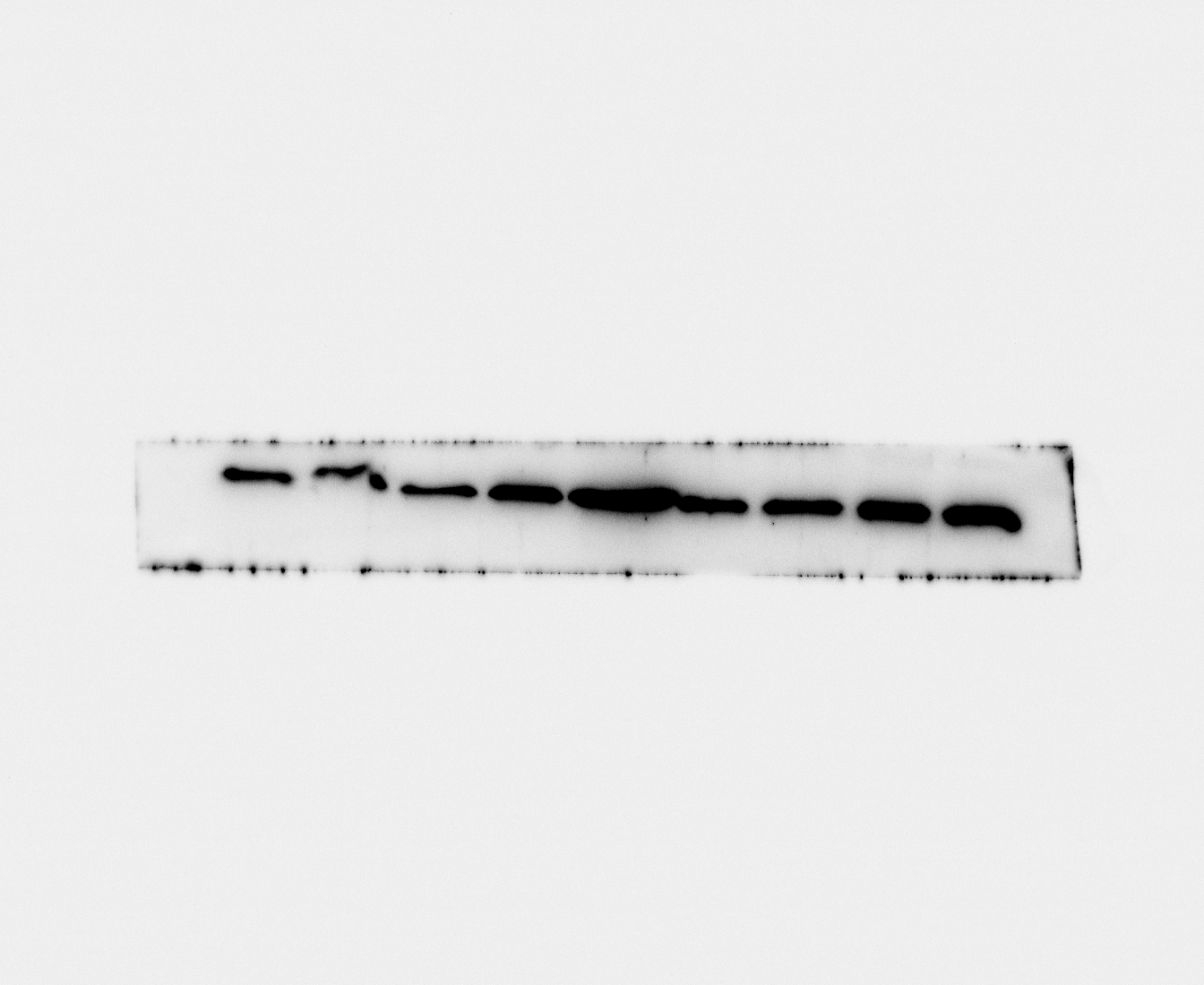

Supplement: Supplementary file 43 — Additional file 43. [file 13020_2026_1383_MOESM43_ESM.tif]

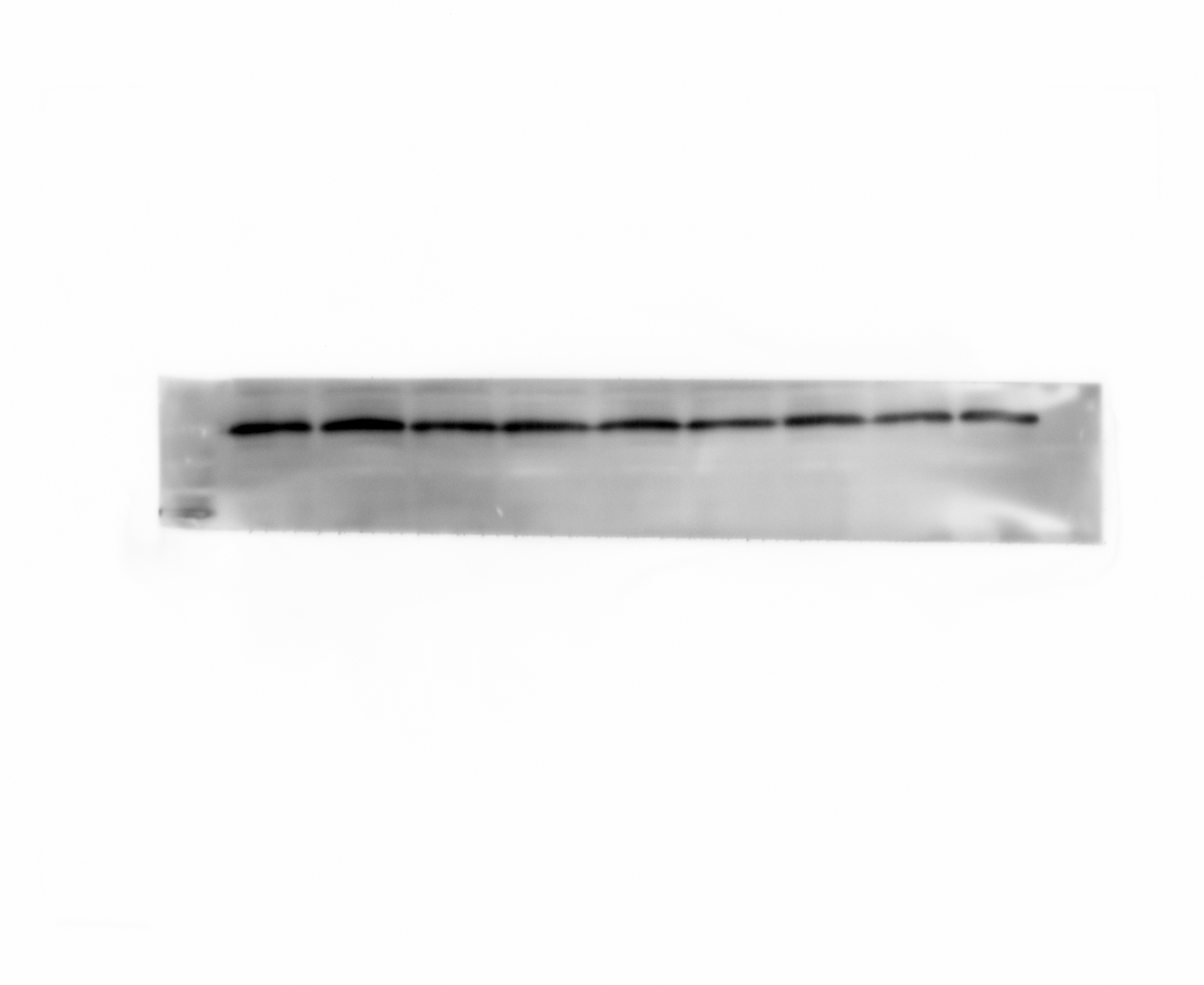

Supplement: Supplementary file 44 — Additional file 44. [file 13020_2026_1383_MOESM44_ESM.tif]

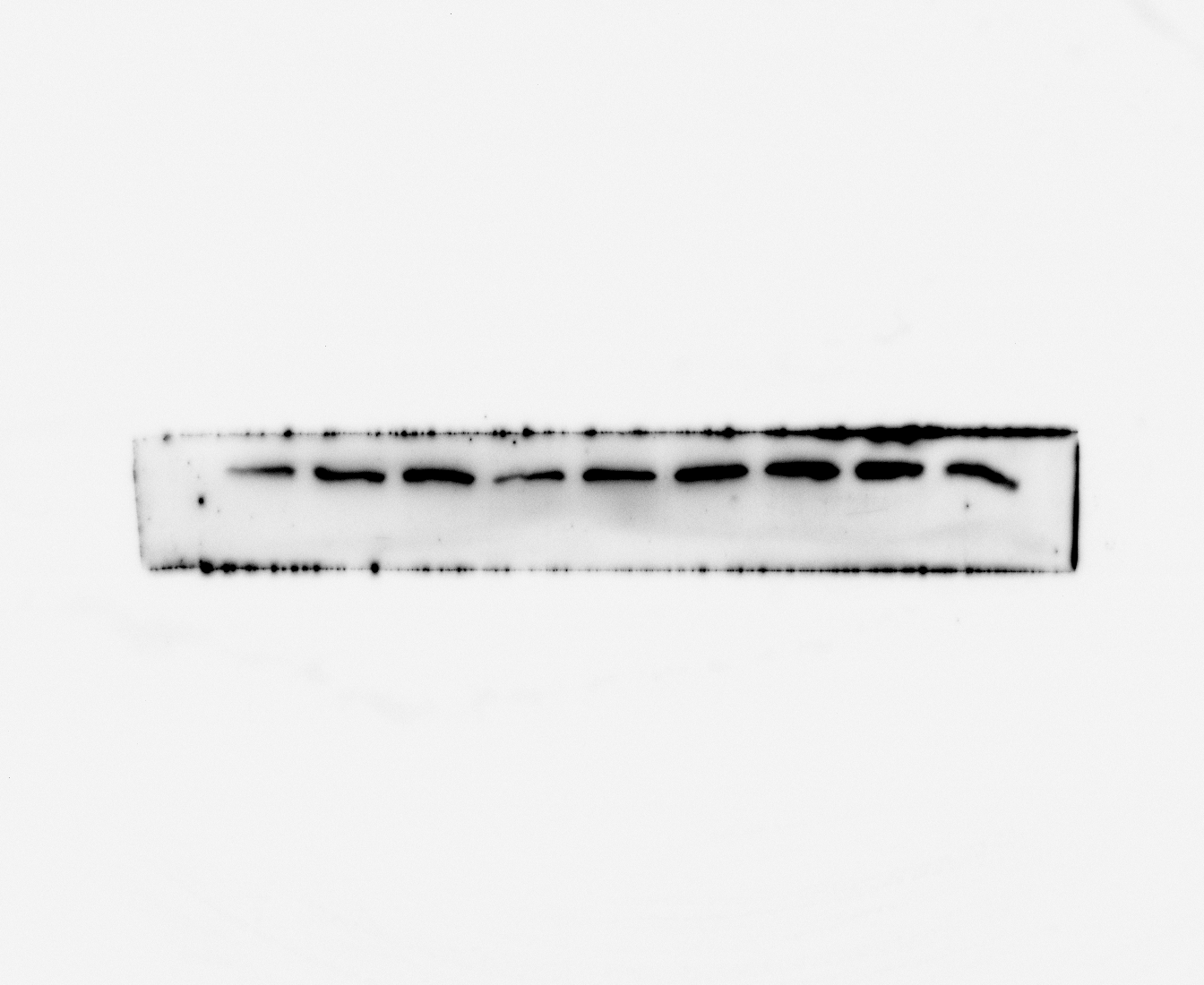

Supplement: Supplementary file 45 — Additional file 45. [file 13020_2026_1383_MOESM45_ESM.tif]

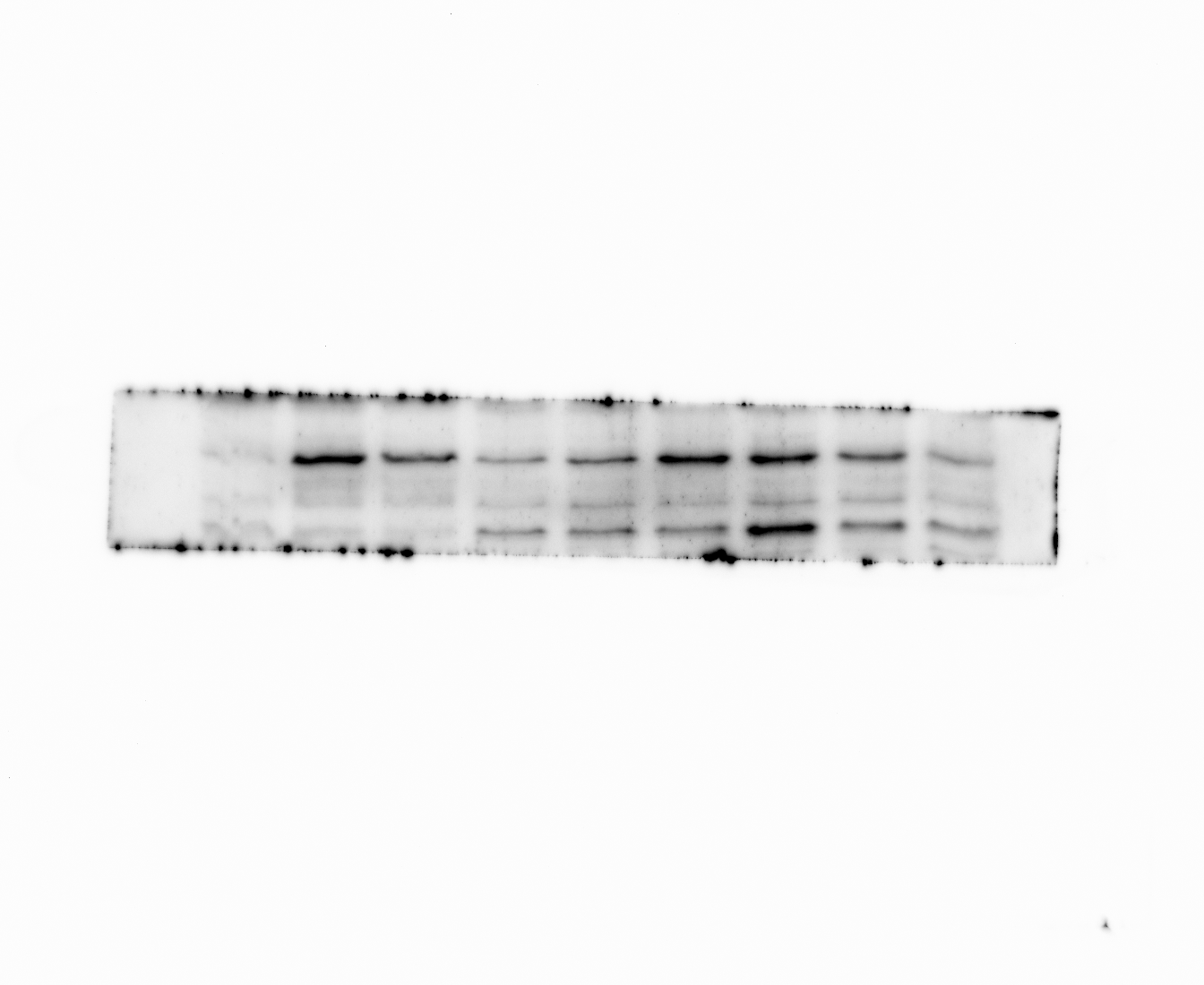

Supplement: Supplementary file 46 — Additional file 46. [file 13020_2026_1383_MOESM46_ESM.tif]

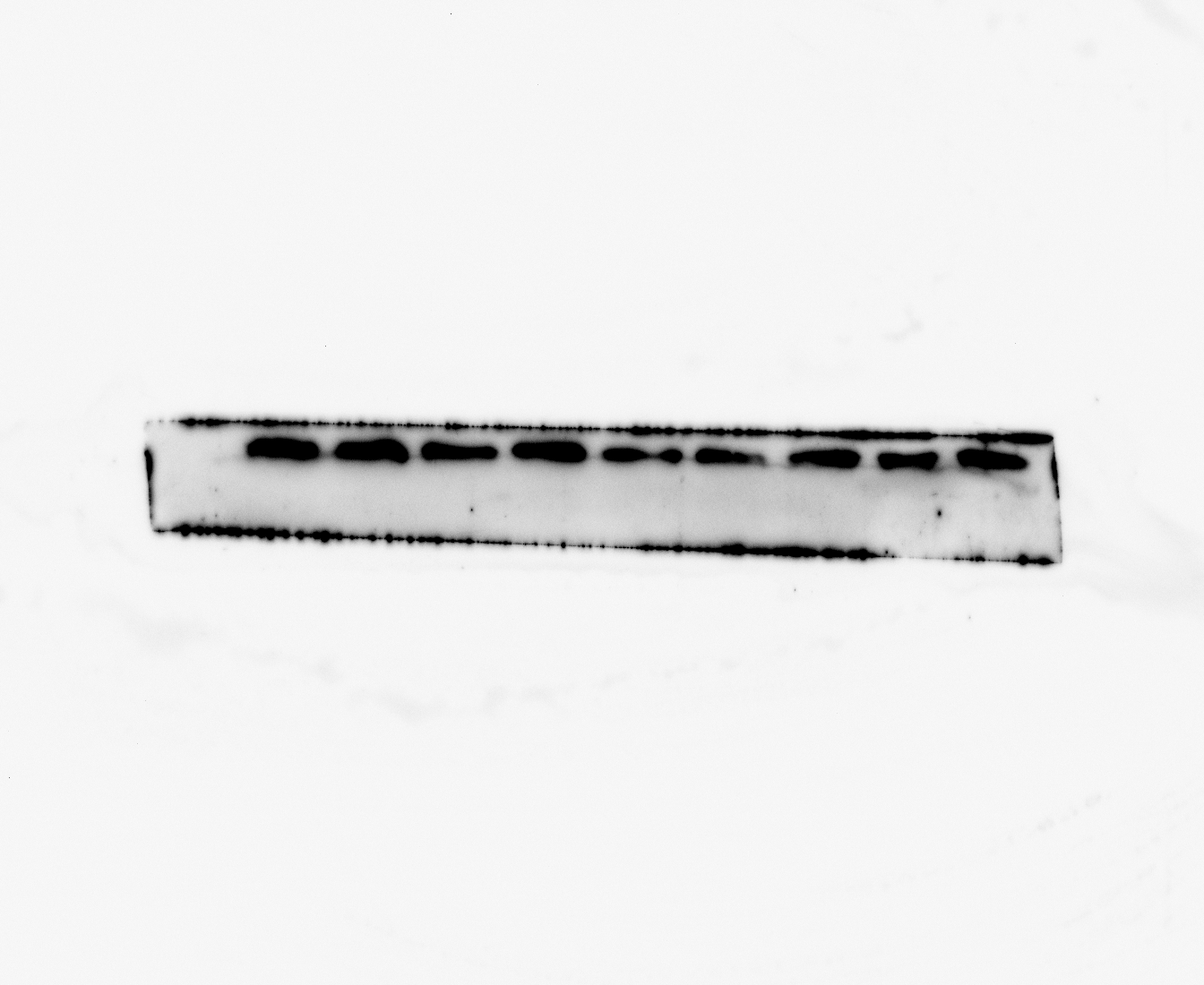

Supplement: Supplementary file 47 — Additional file 47. [file 13020_2026_1383_MOESM47_ESM.tif]

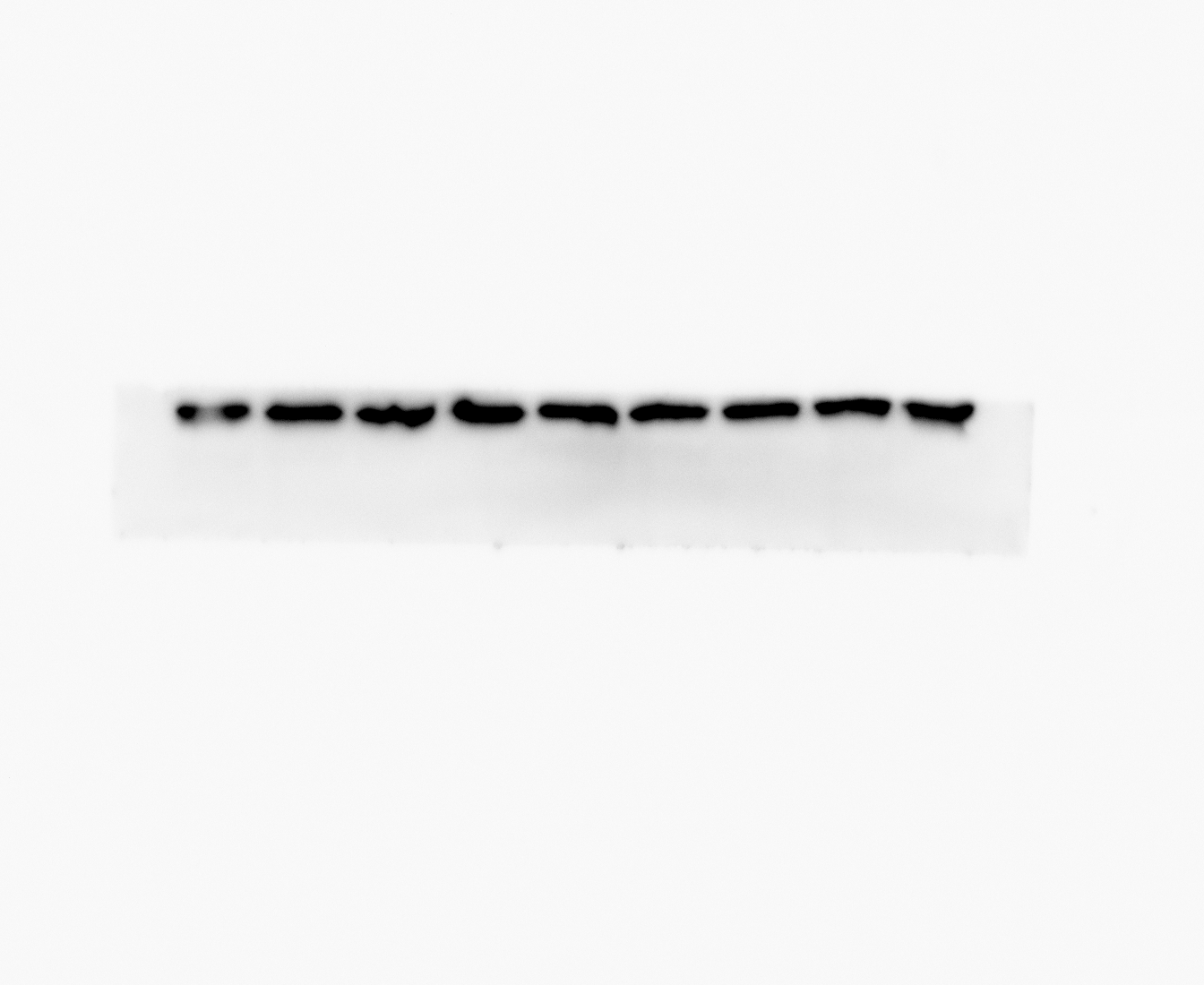

Supplement: Supplementary file 48 — Additional file 48. [file 13020_2026_1383_MOESM48_ESM.tif]

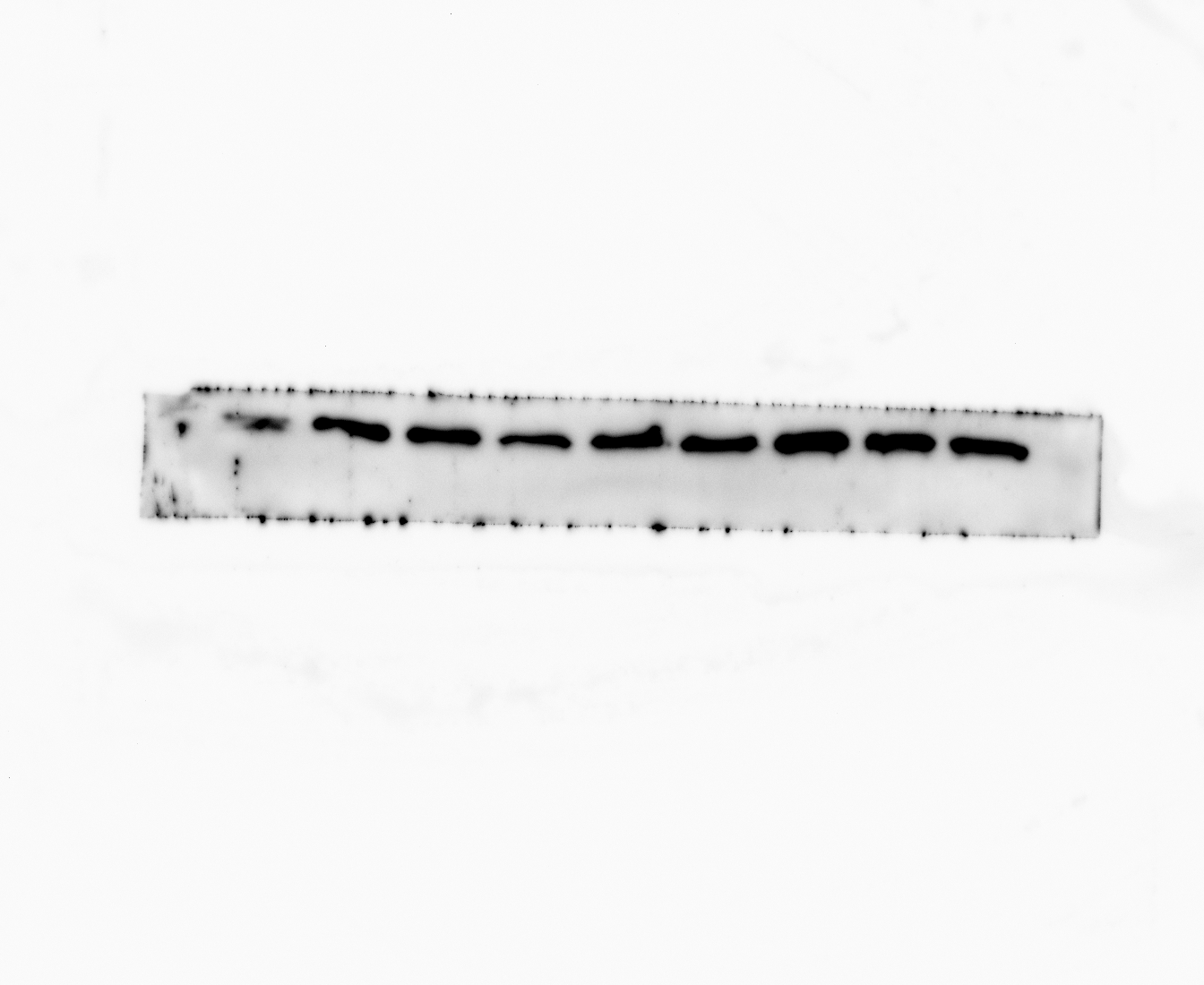

Supplement: Supplementary file 49 — Additional file 49. [file 13020_2026_1383_MOESM49_ESM.tif]

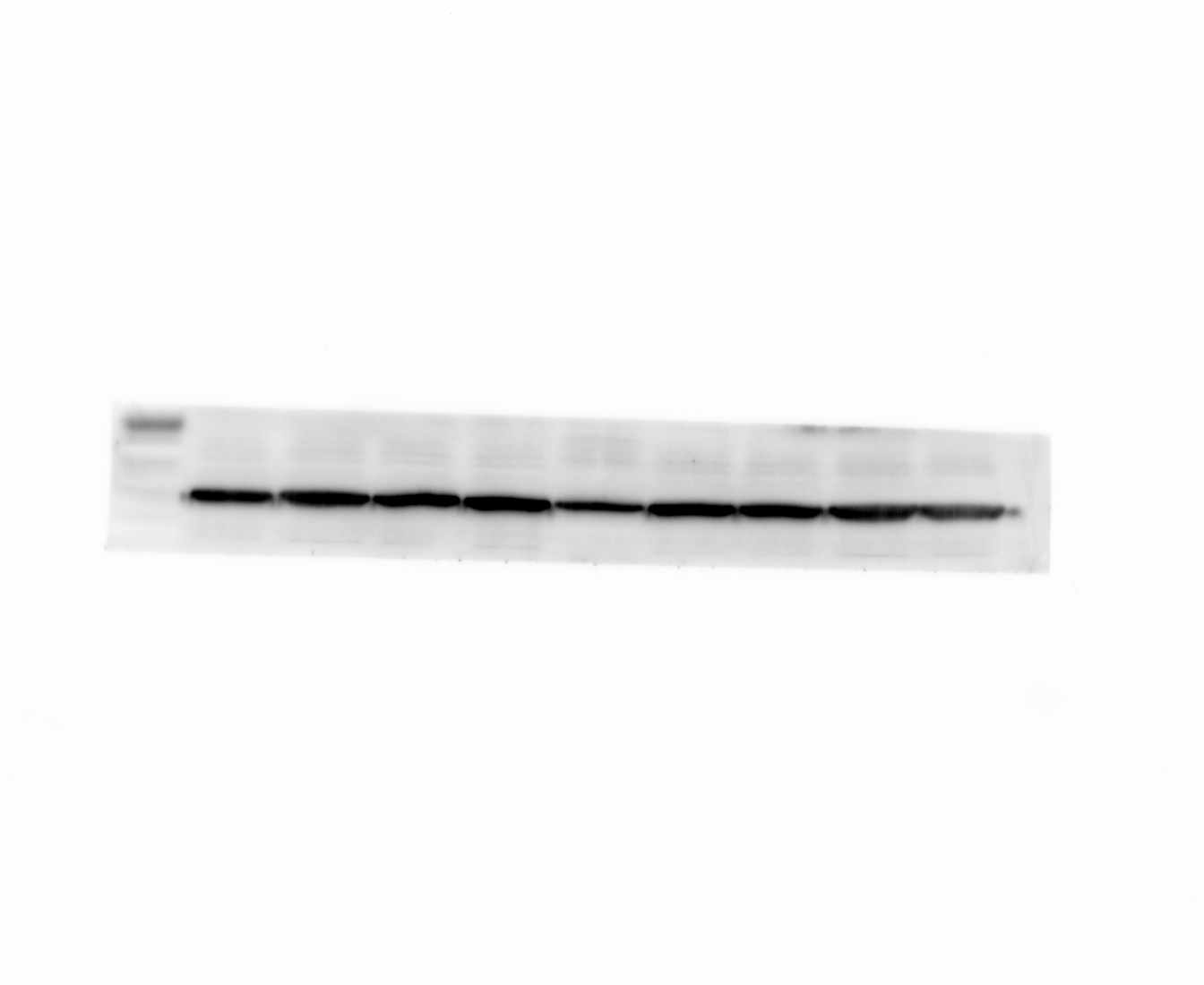

Supplement: Supplementary file 50 — Additional file 50. [file 13020_2026_1383_MOESM50_ESM.tif]

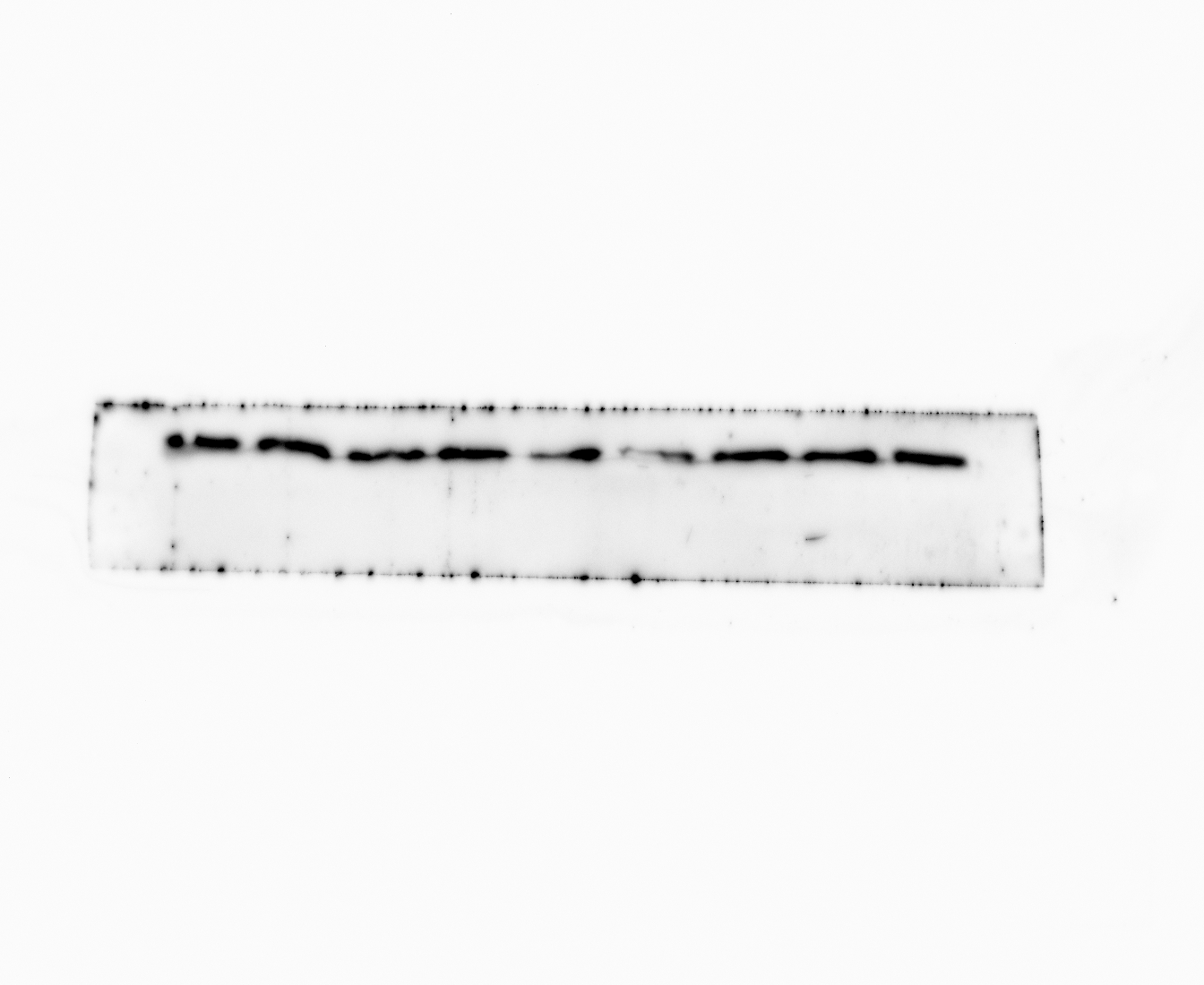

Supplement: Supplementary file 51 — Additional file 51. [file 13020_2026_1383_MOESM51_ESM.tif]

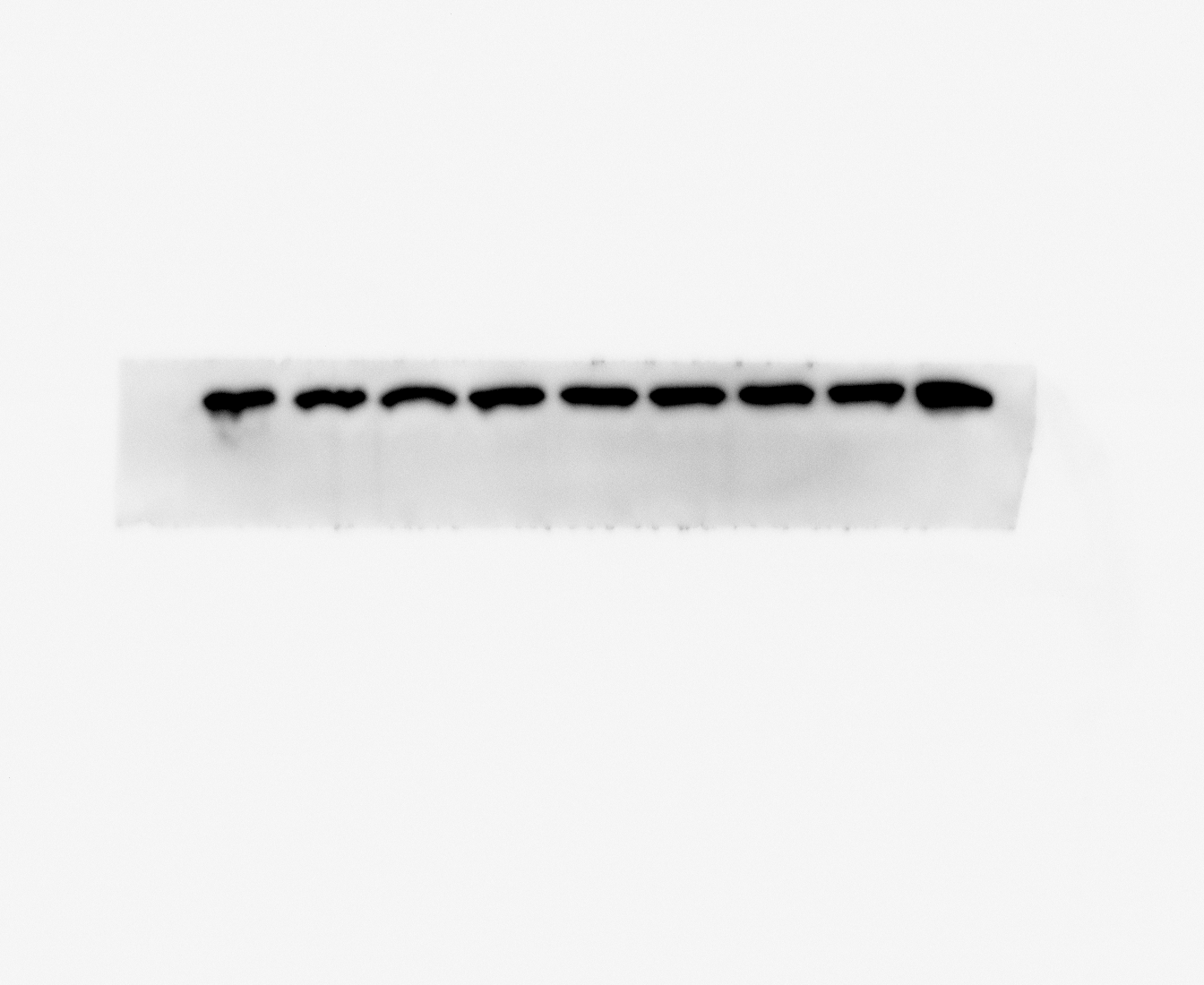

Supplement: Supplementary file 52 — Additional file 52. [file 13020_2026_1383_MOESM52_ESM.tif]

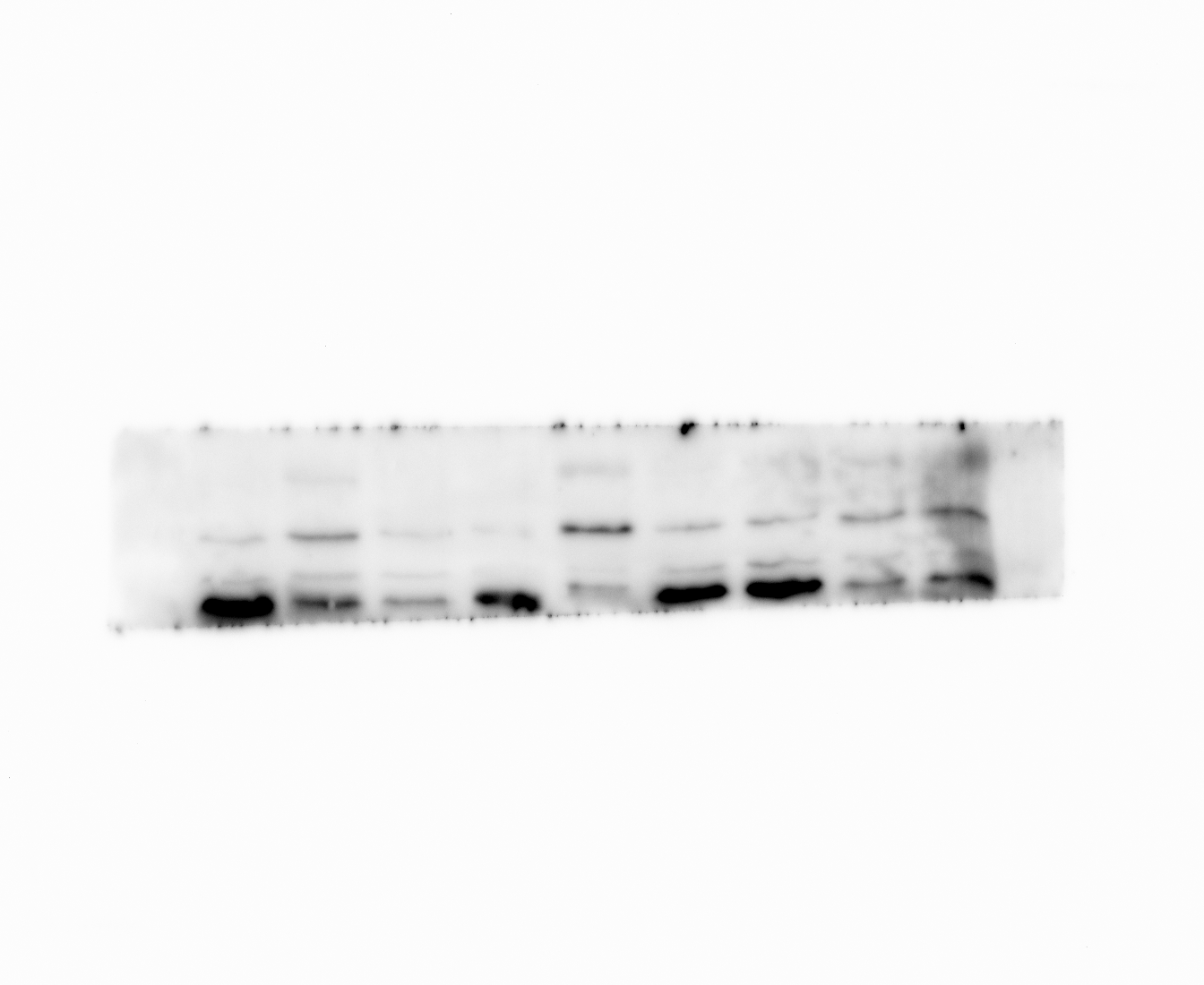

Supplement: Supplementary file 53 — Additional file 53. [file 13020_2026_1383_MOESM53_ESM.tif]

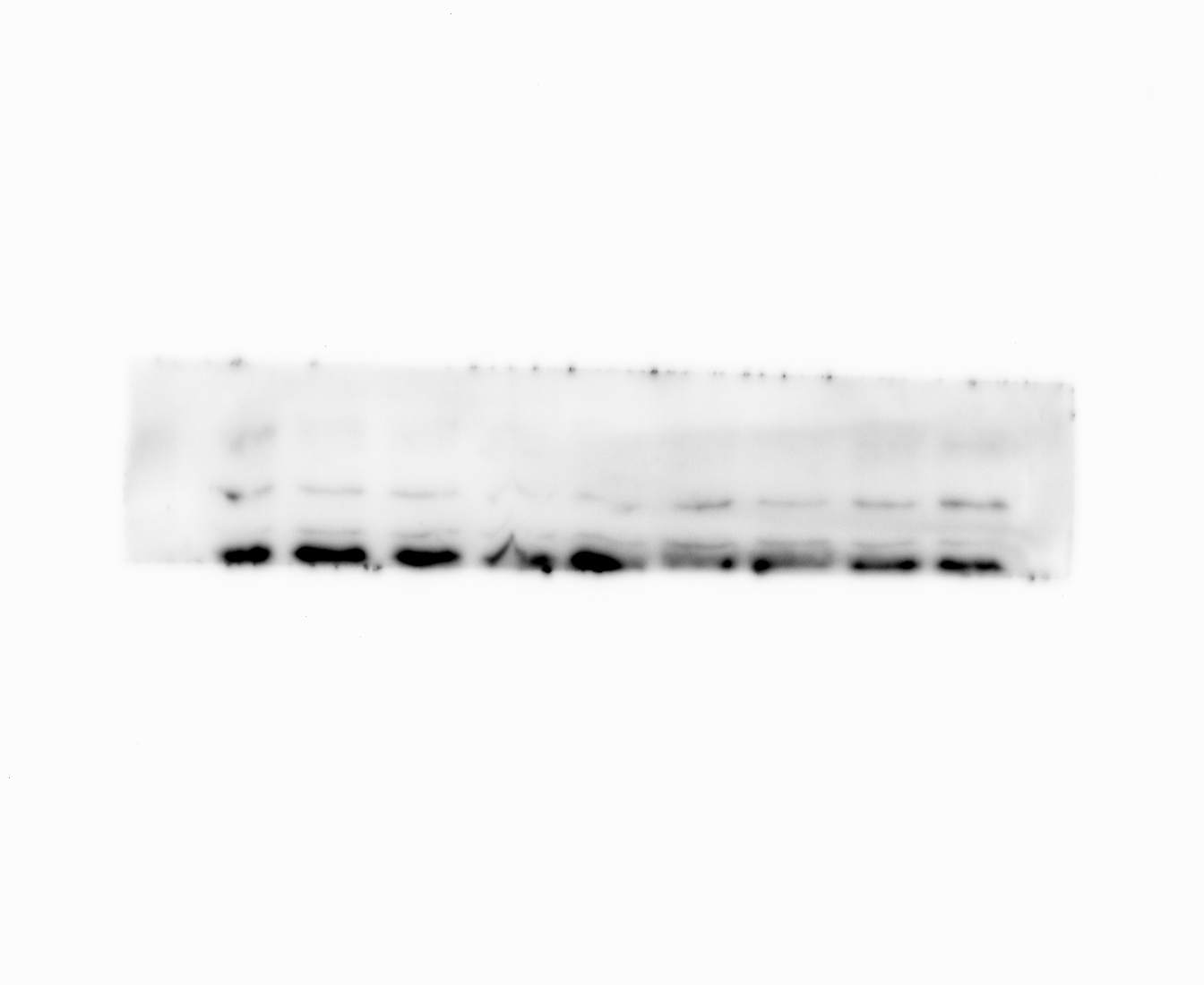

Supplement: Supplementary file 54 — Additional file 54. [file 13020_2026_1383_MOESM54_ESM.tif]

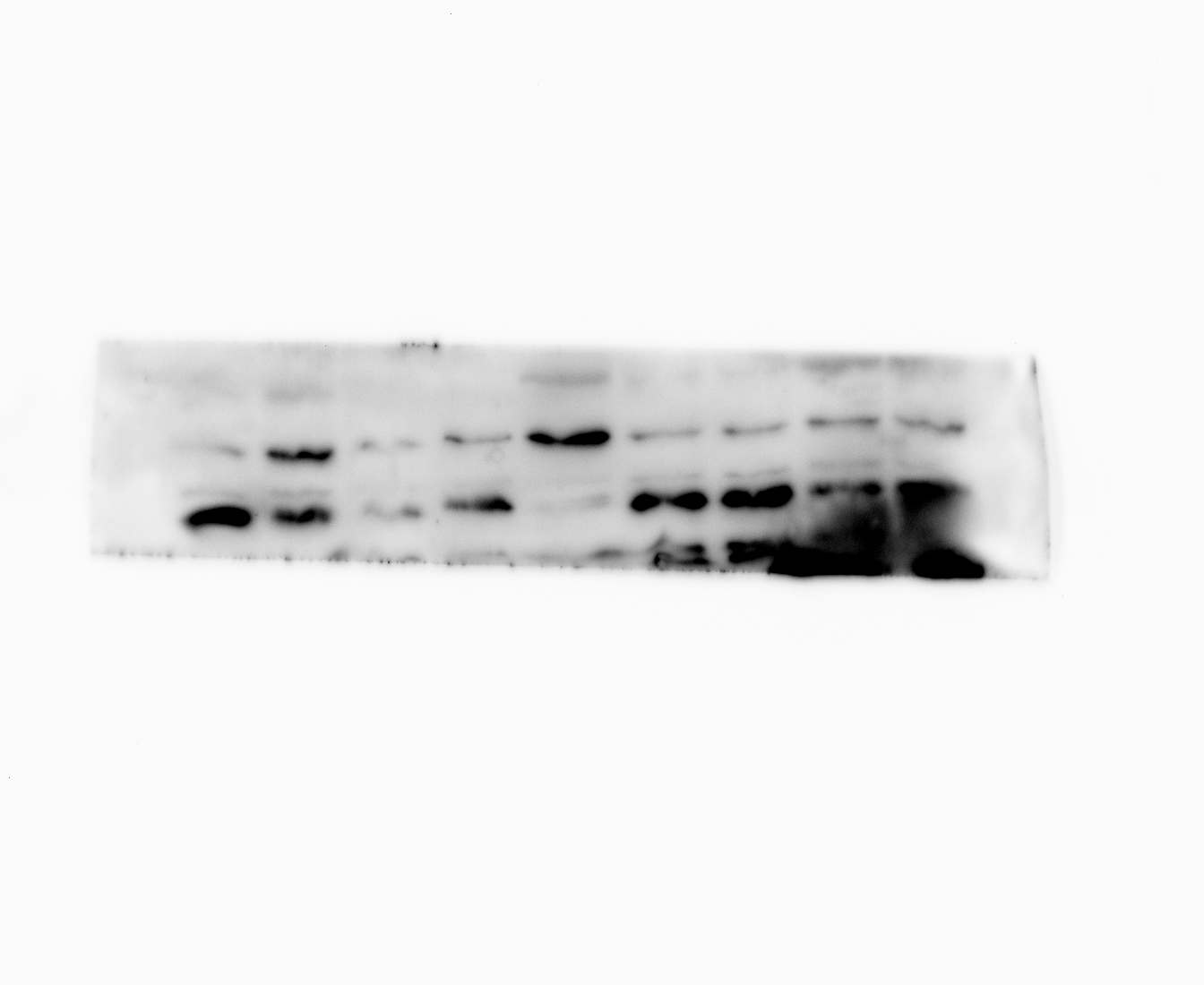

Supplement: Supplementary file 55 — Additional file 55. [file 13020_2026_1383_MOESM55_ESM.tif]

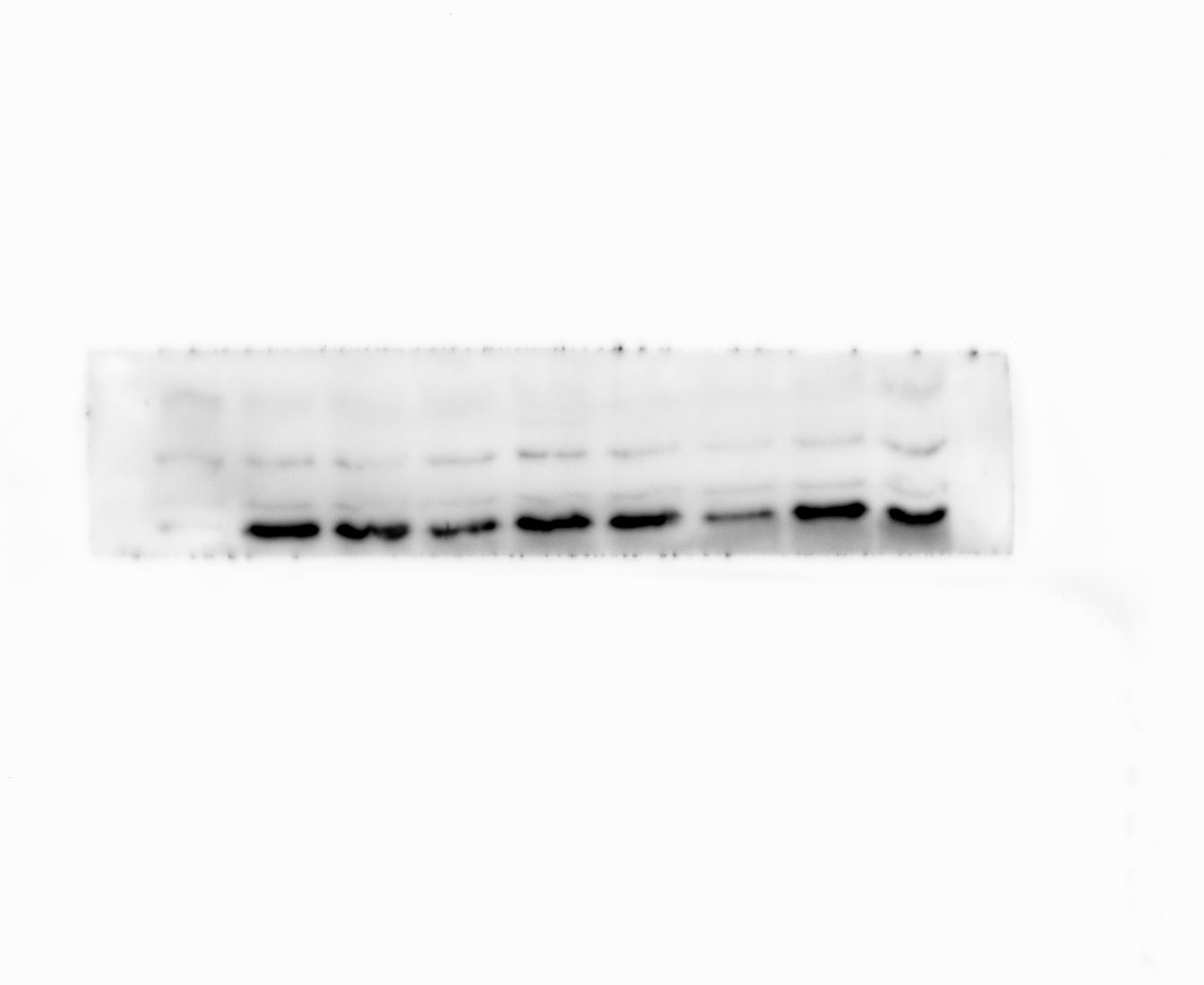

Supplement: Supplementary file 56 — Additional file 56. [file 13020_2026_1383_MOESM56_ESM.tif]

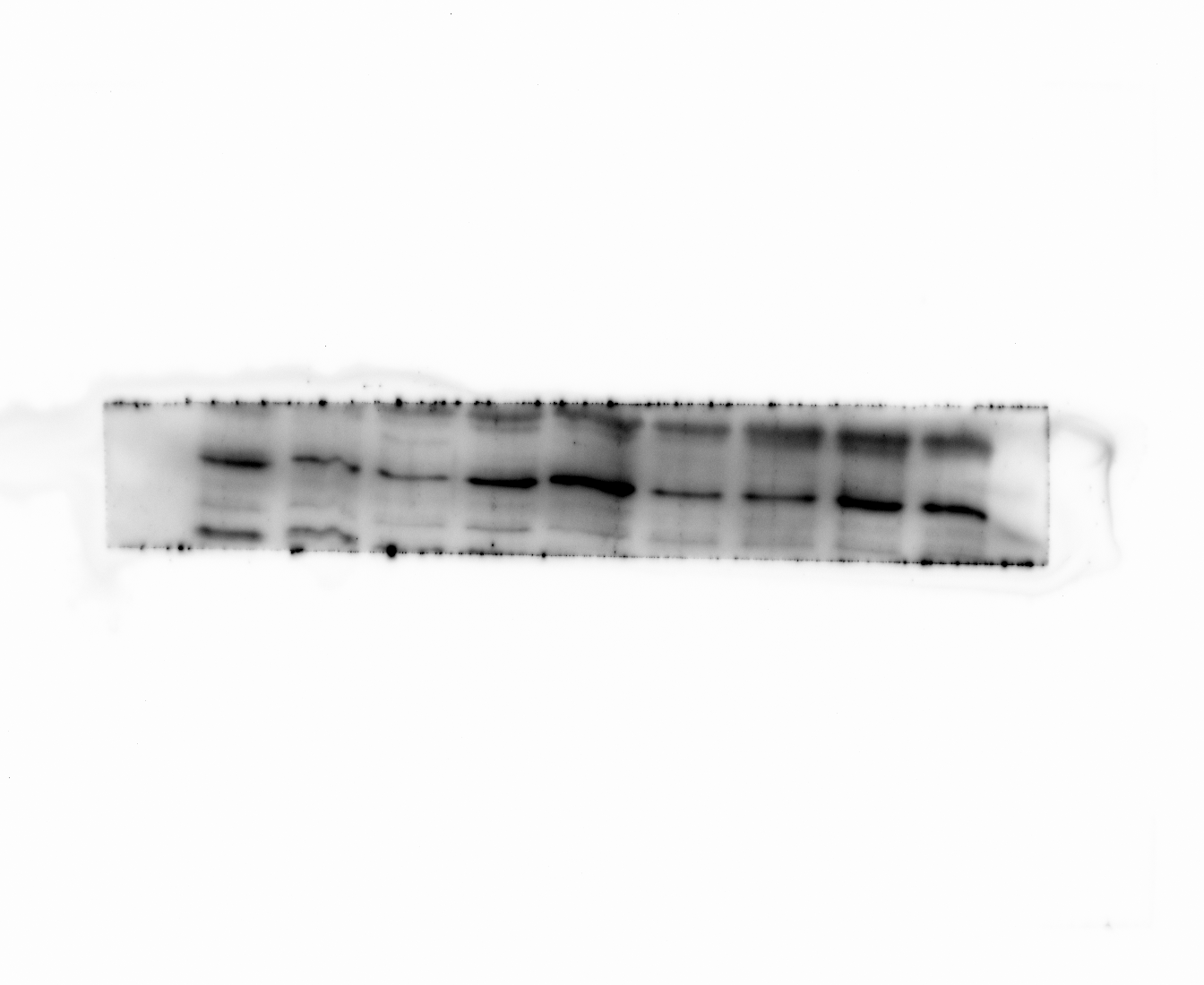

Supplement: Supplementary file 57 — Additional file 57. [file 13020_2026_1383_MOESM57_ESM.tif]

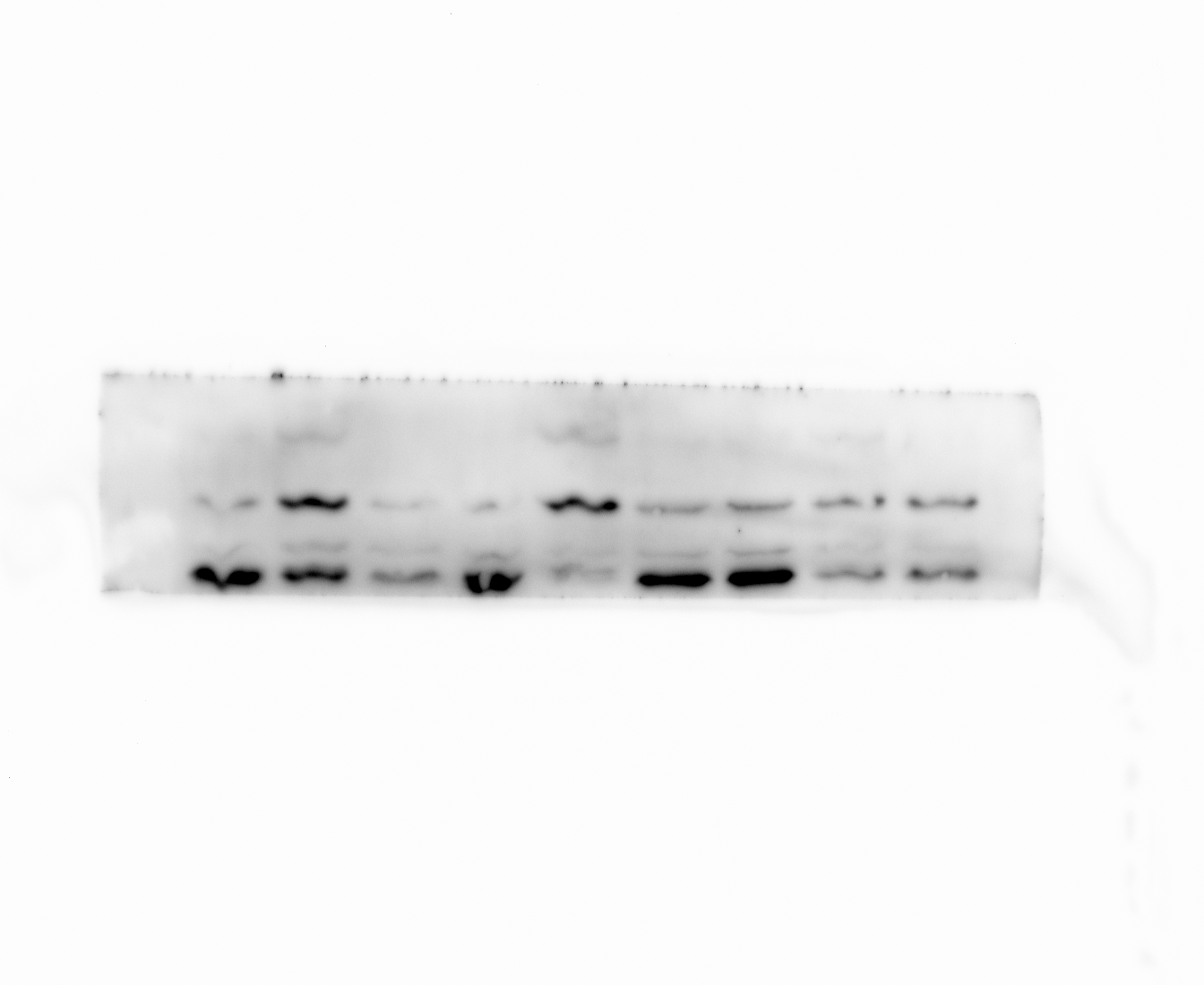

Supplement: Supplementary file 58 — Additional file 58. [file 13020_2026_1383_MOESM58_ESM.tif]

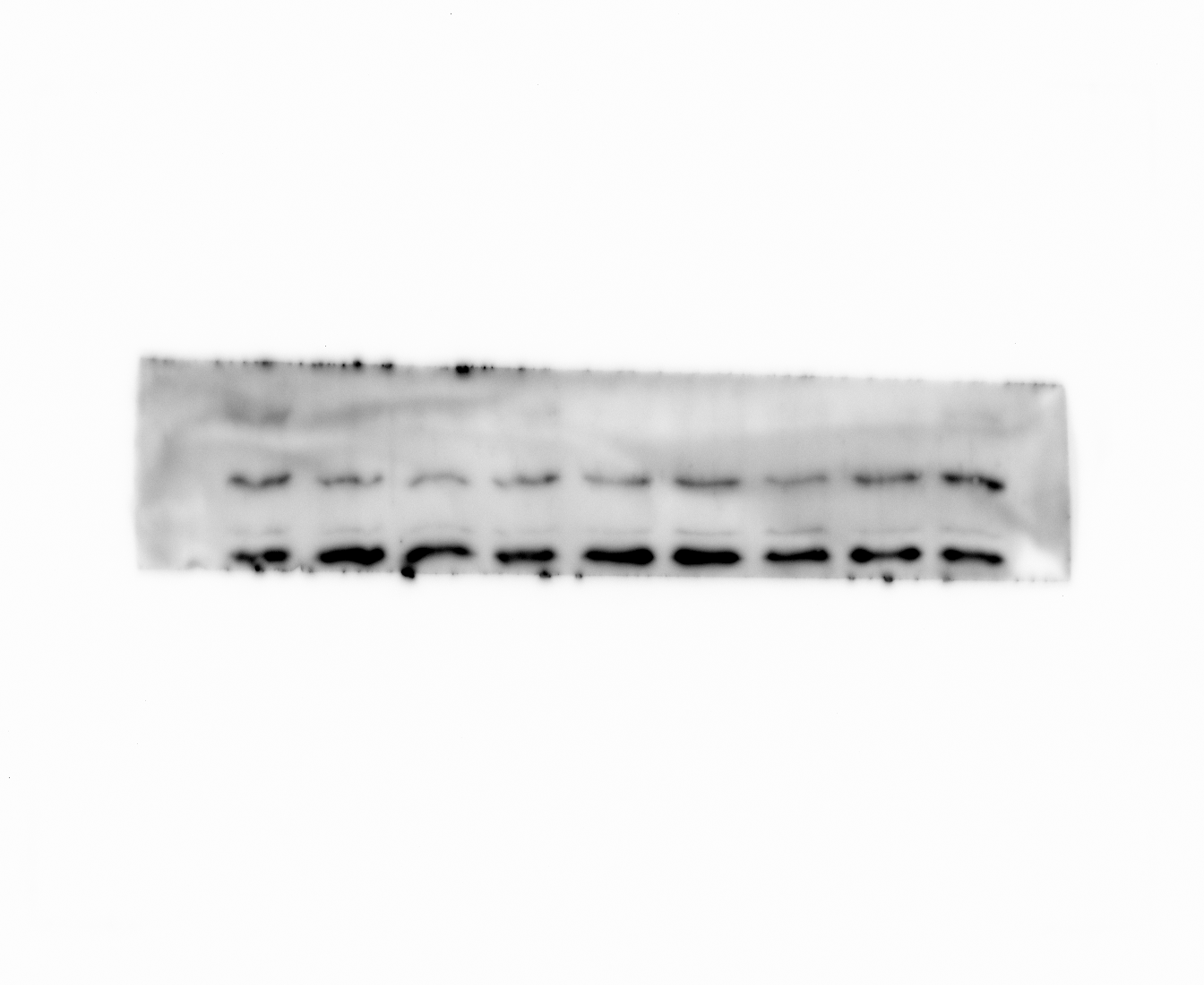

Supplement: Supplementary file 59 — Additional file 59. [file 13020_2026_1383_MOESM59_ESM.tif]

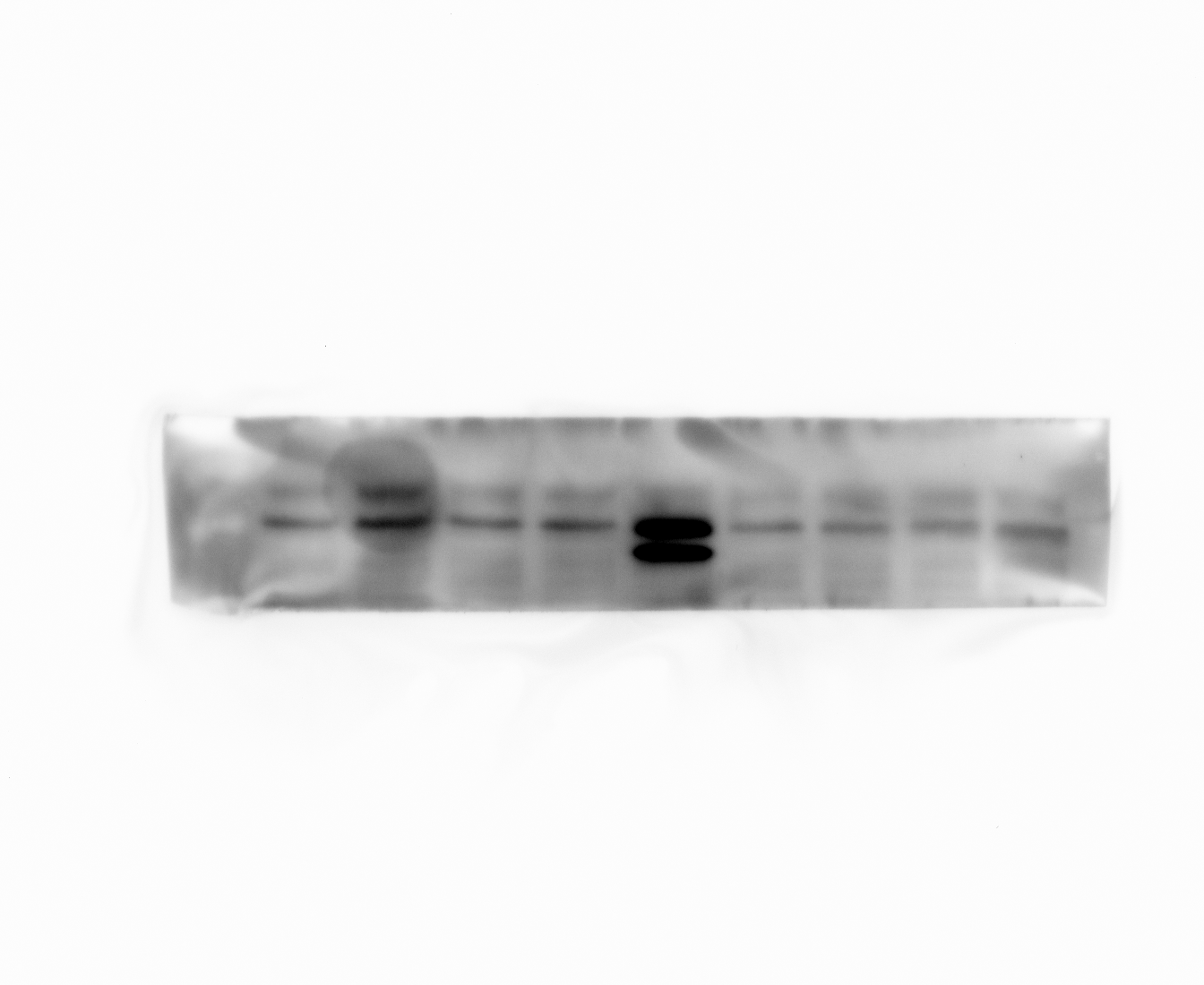

Supplement: Supplementary file 60 — Additional file 60. [file 13020_2026_1383_MOESM60_ESM.tif]

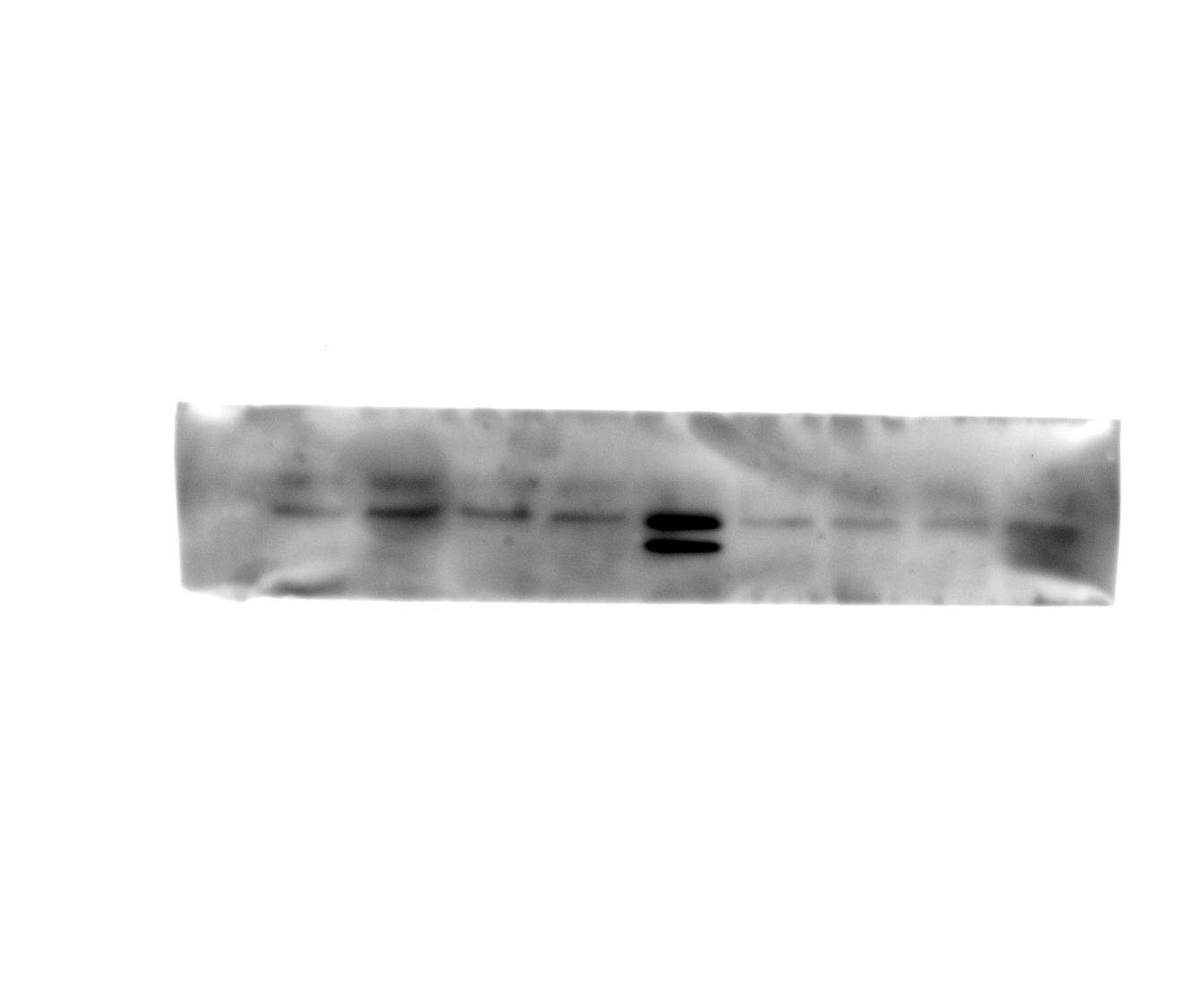

Supplement: Supplementary file 61 — Additional file 61. [file 13020_2026_1383_MOESM61_ESM.tif]

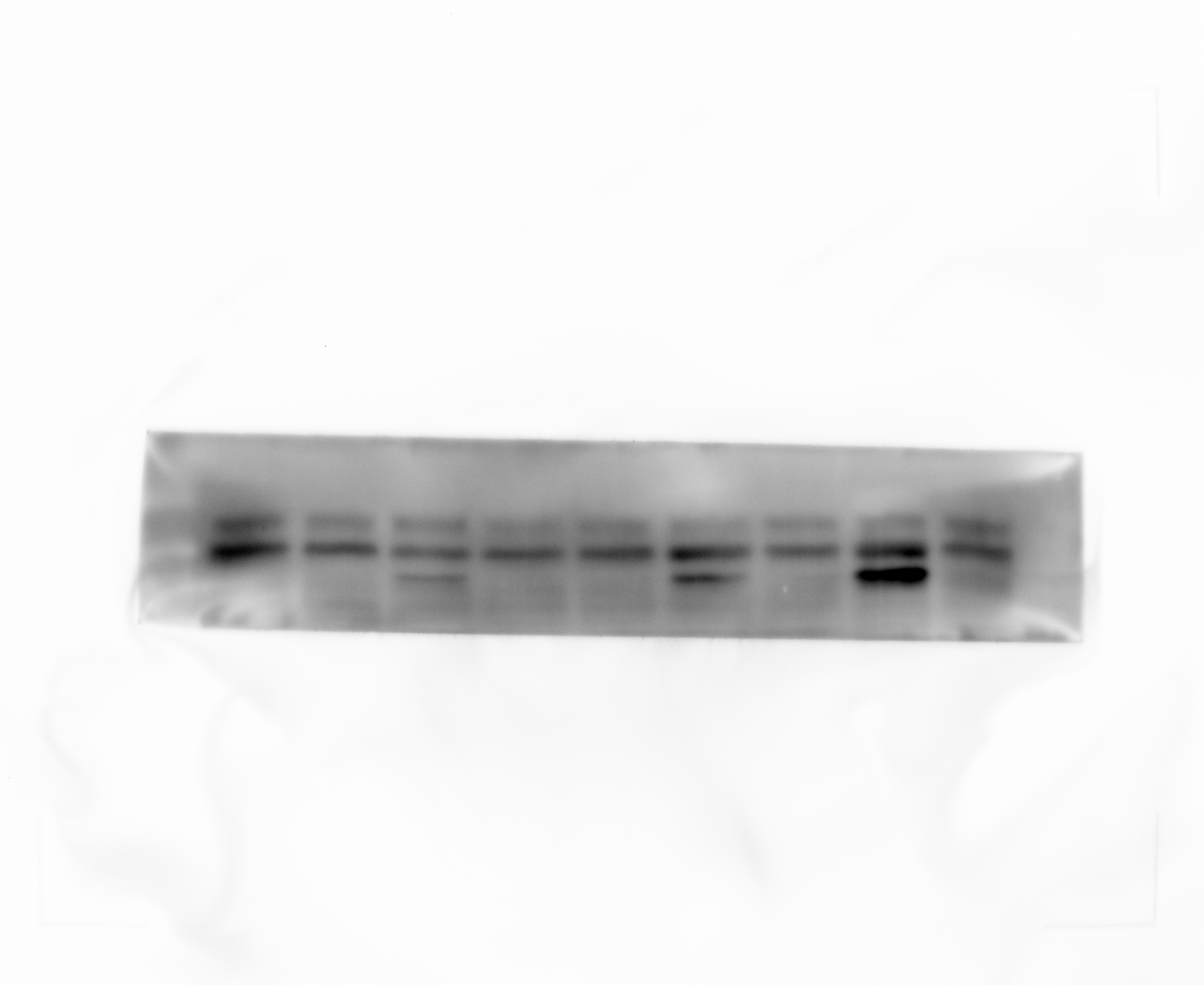

Supplement: Supplementary file 62 — Additional file 62. [file 13020_2026_1383_MOESM62_ESM.tif]

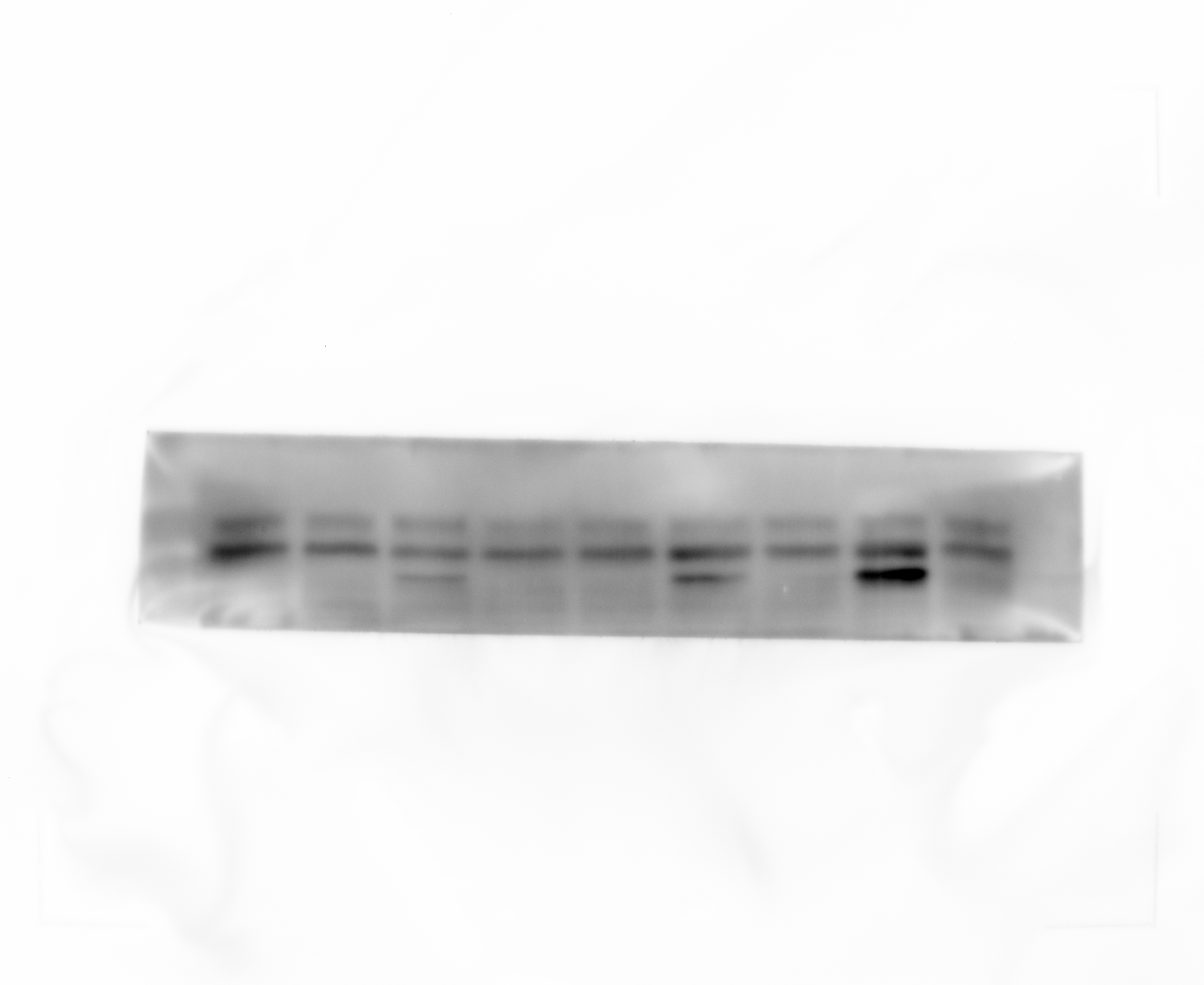

Supplement: Supplementary file 63 — Additional file 63. [file 13020_2026_1383_MOESM63_ESM.tif]

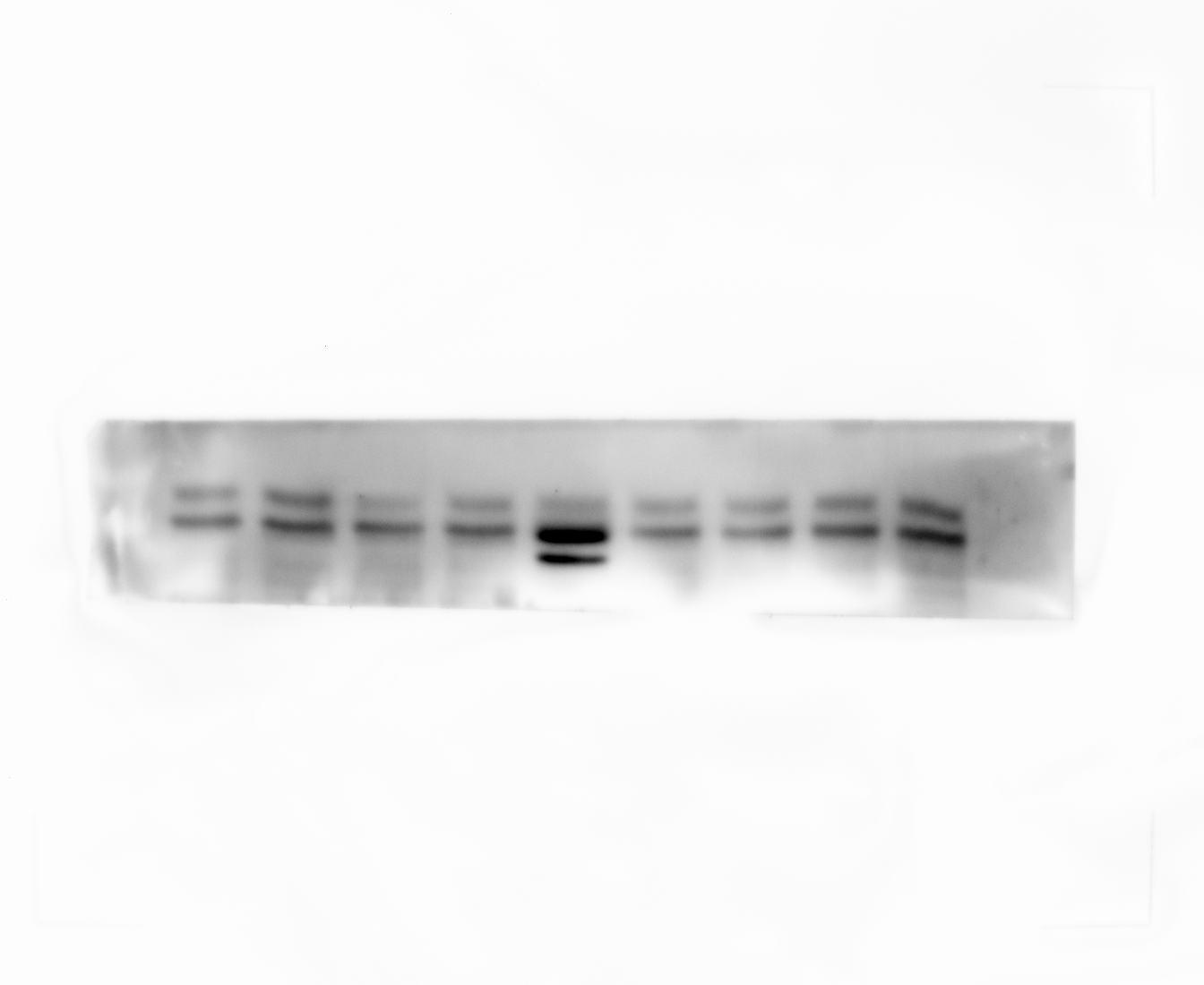

Supplement: Supplementary file 64 — Additional file 64. [file 13020_2026_1383_MOESM64_ESM.tif]

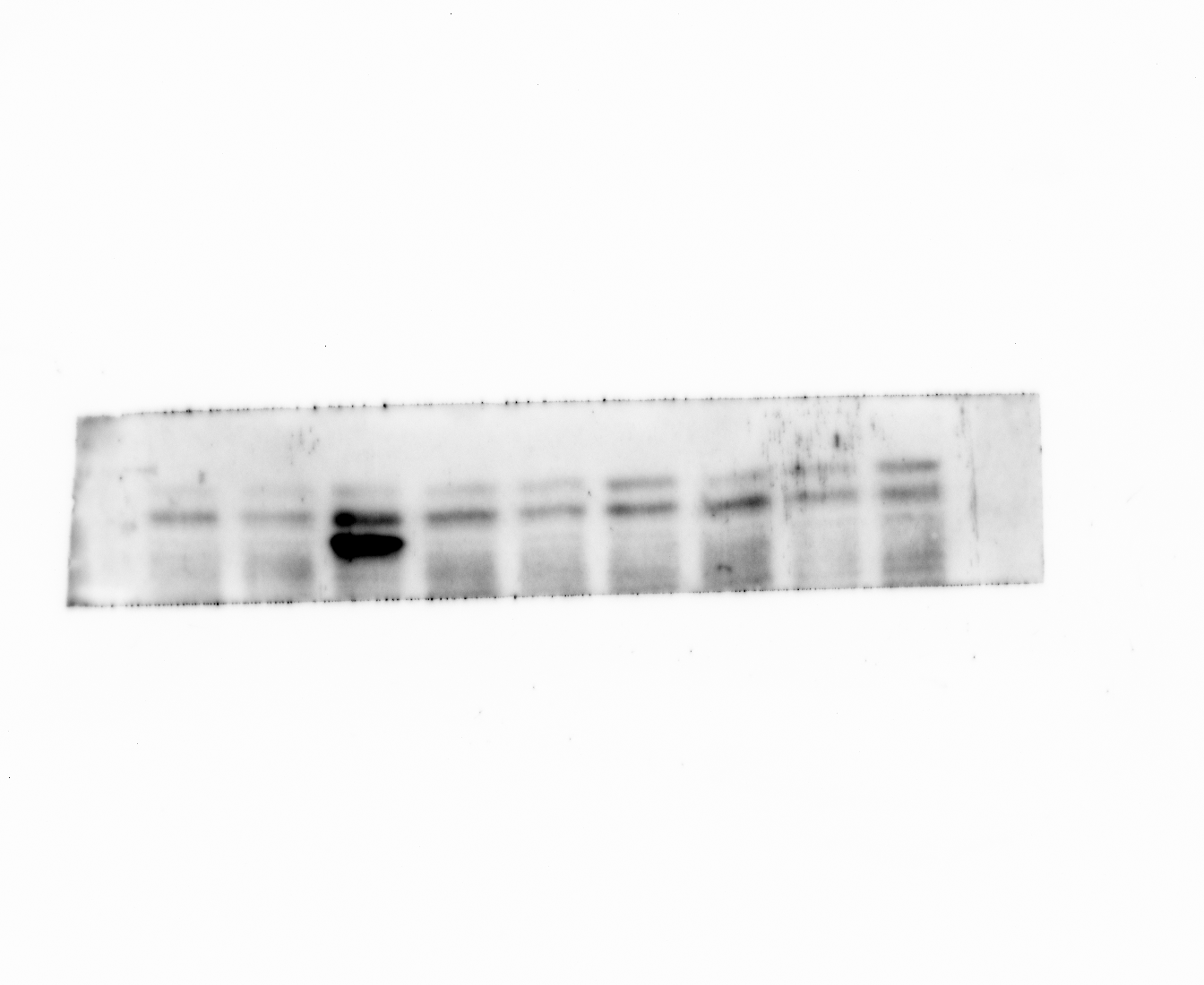

Supplement: Supplementary file 65 — Additional file 65. [file 13020_2026_1383_MOESM65_ESM.tif]

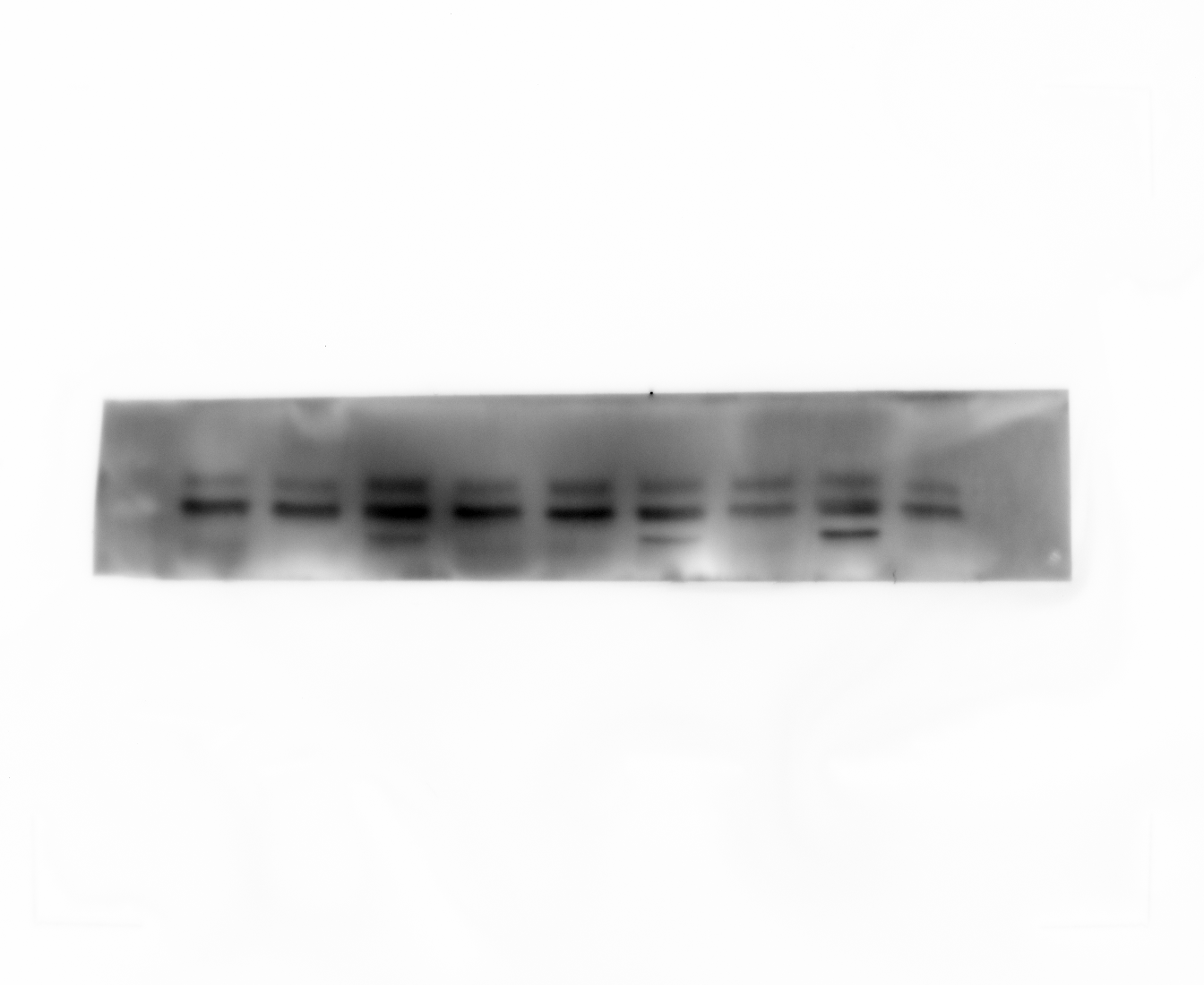

Supplement: Supplementary file 66 — Additional file 66. [file 13020_2026_1383_MOESM66_ESM.tif]

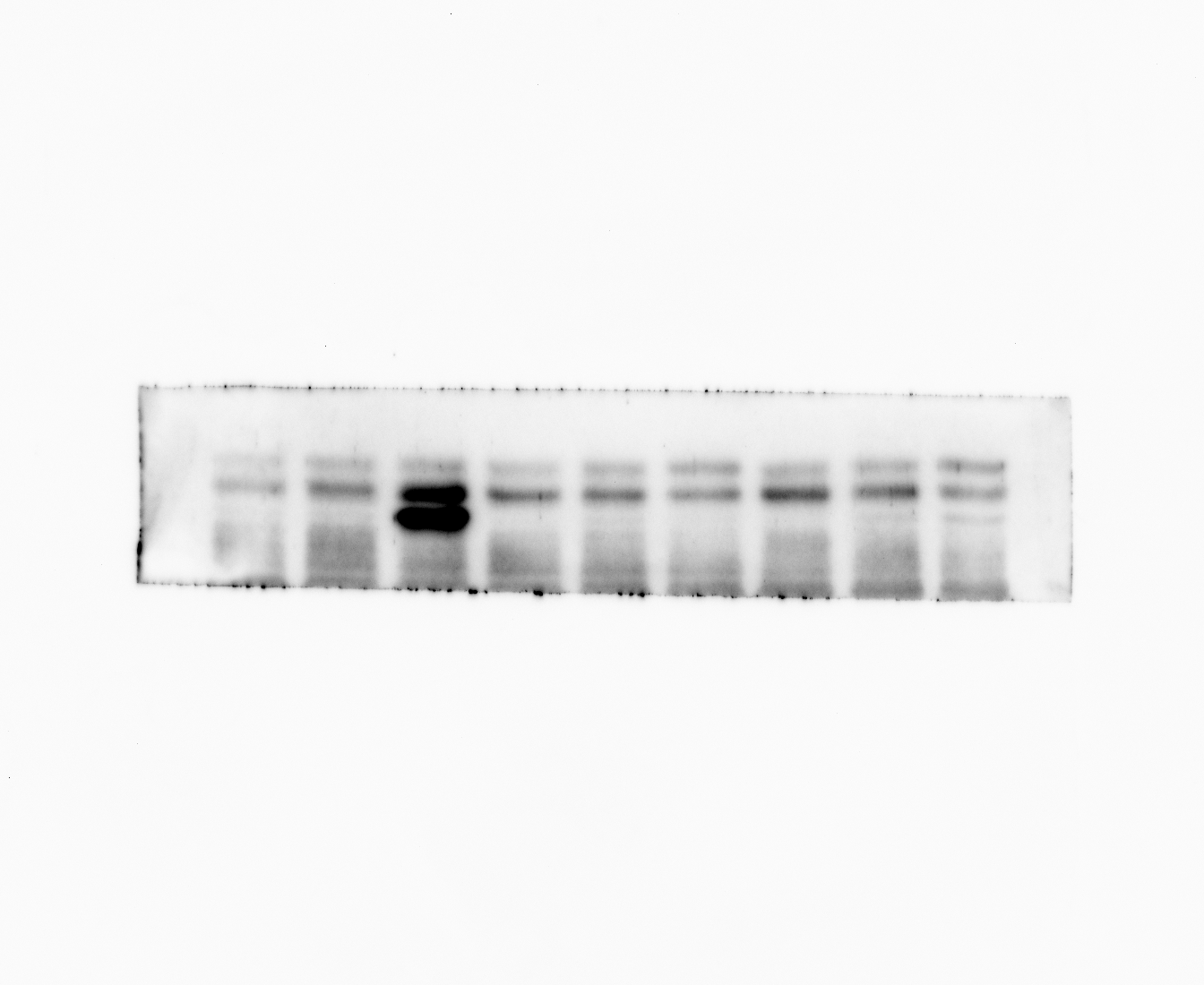

Supplement: Supplementary file 67 — Additional file 67. [file 13020_2026_1383_MOESM67_ESM.tif]

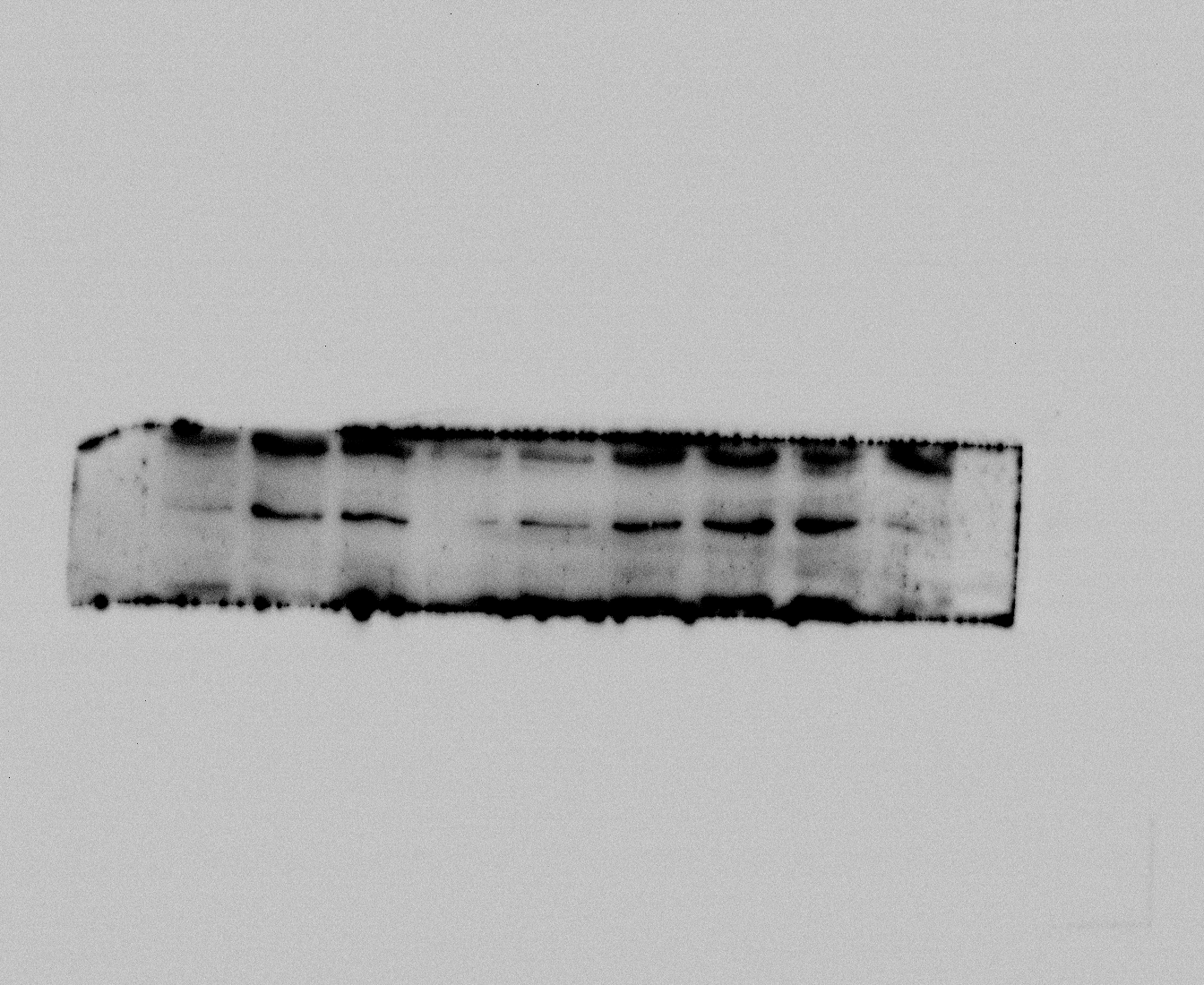

Supplement: Supplementary file 68 — Additional file 68. [file 13020_2026_1383_MOESM68_ESM.tif]

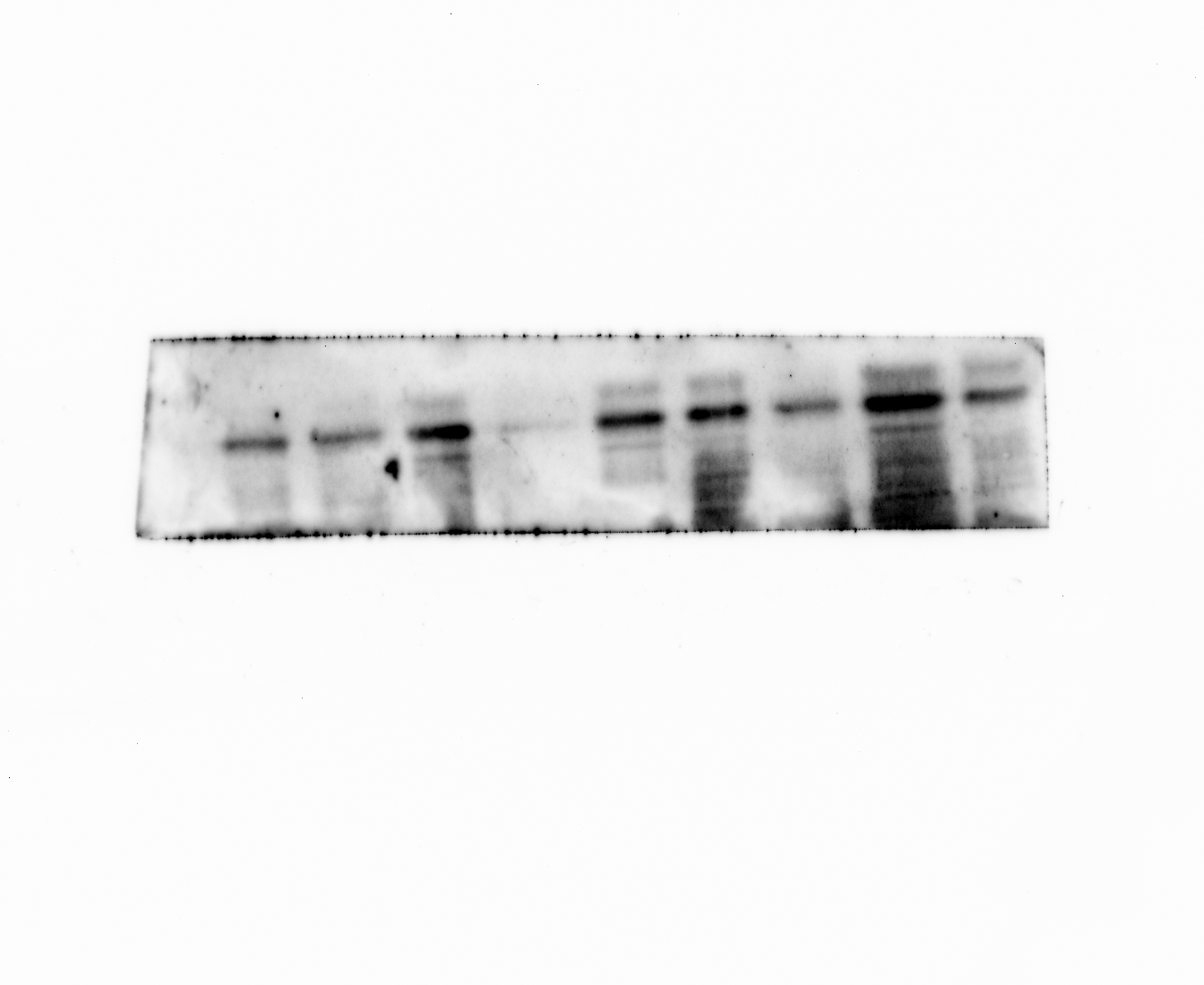

Supplement: Supplementary file 69 — Additional file 69. [file 13020_2026_1383_MOESM69_ESM.tif]

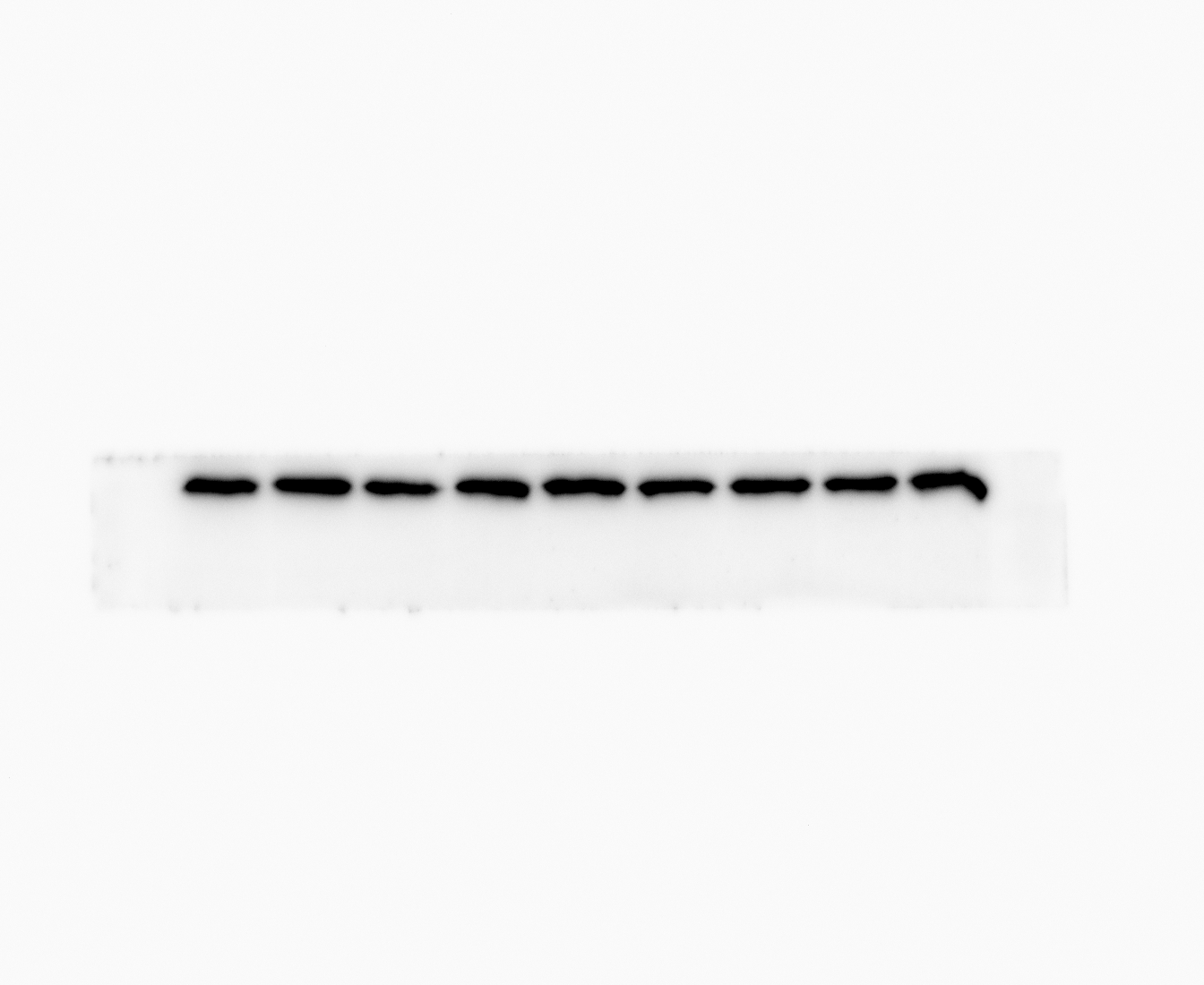

Supplement: Supplementary file 70 — Additional file 70. [file 13020_2026_1383_MOESM70_ESM.tif]

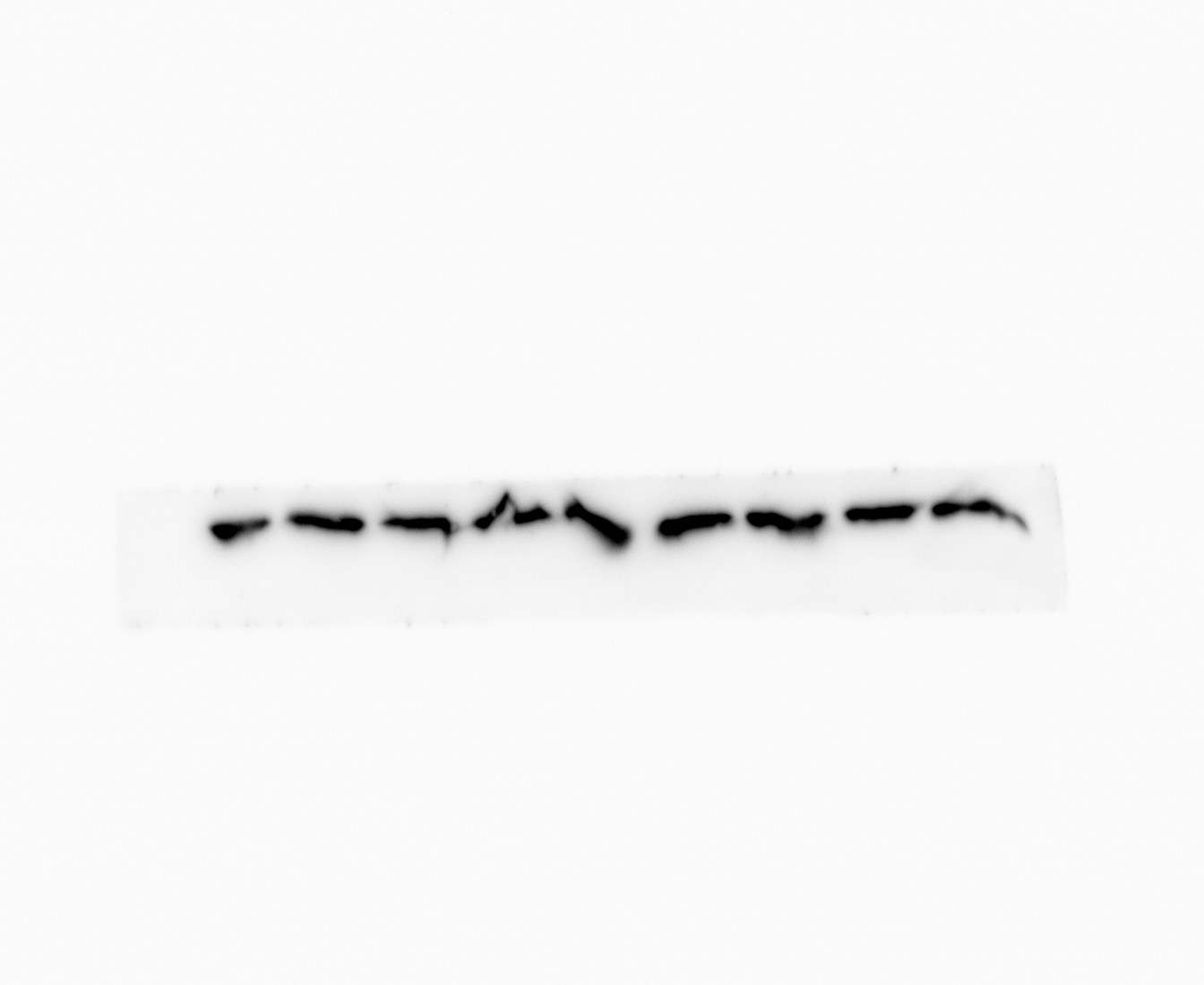

Supplement: Supplementary file 71 — Additional file 71. [file 13020_2026_1383_MOESM71_ESM.tif]

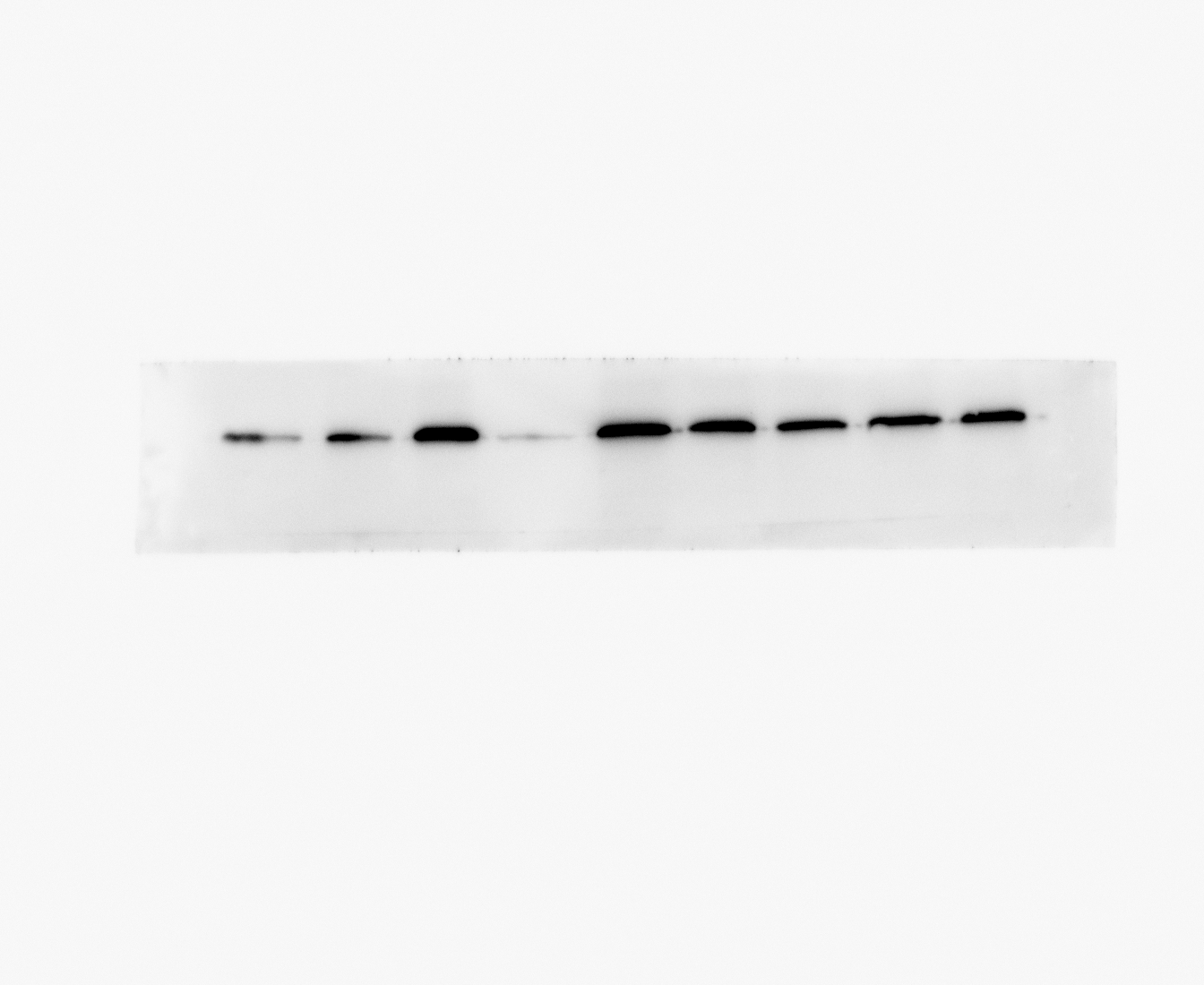

Supplement: Supplementary file 72 — Additional file 72. [file 13020_2026_1383_MOESM72_ESM.tif]

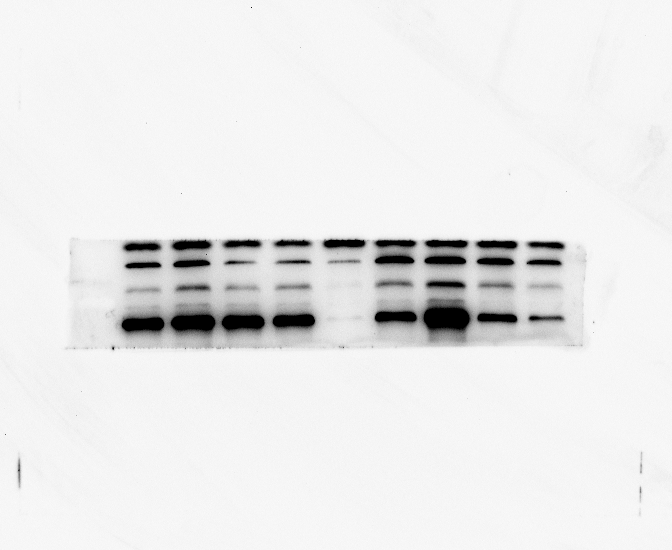

Supplement: Supplementary file 73 — Additional file 73. [file 13020_2026_1383_MOESM73_ESM.tif]

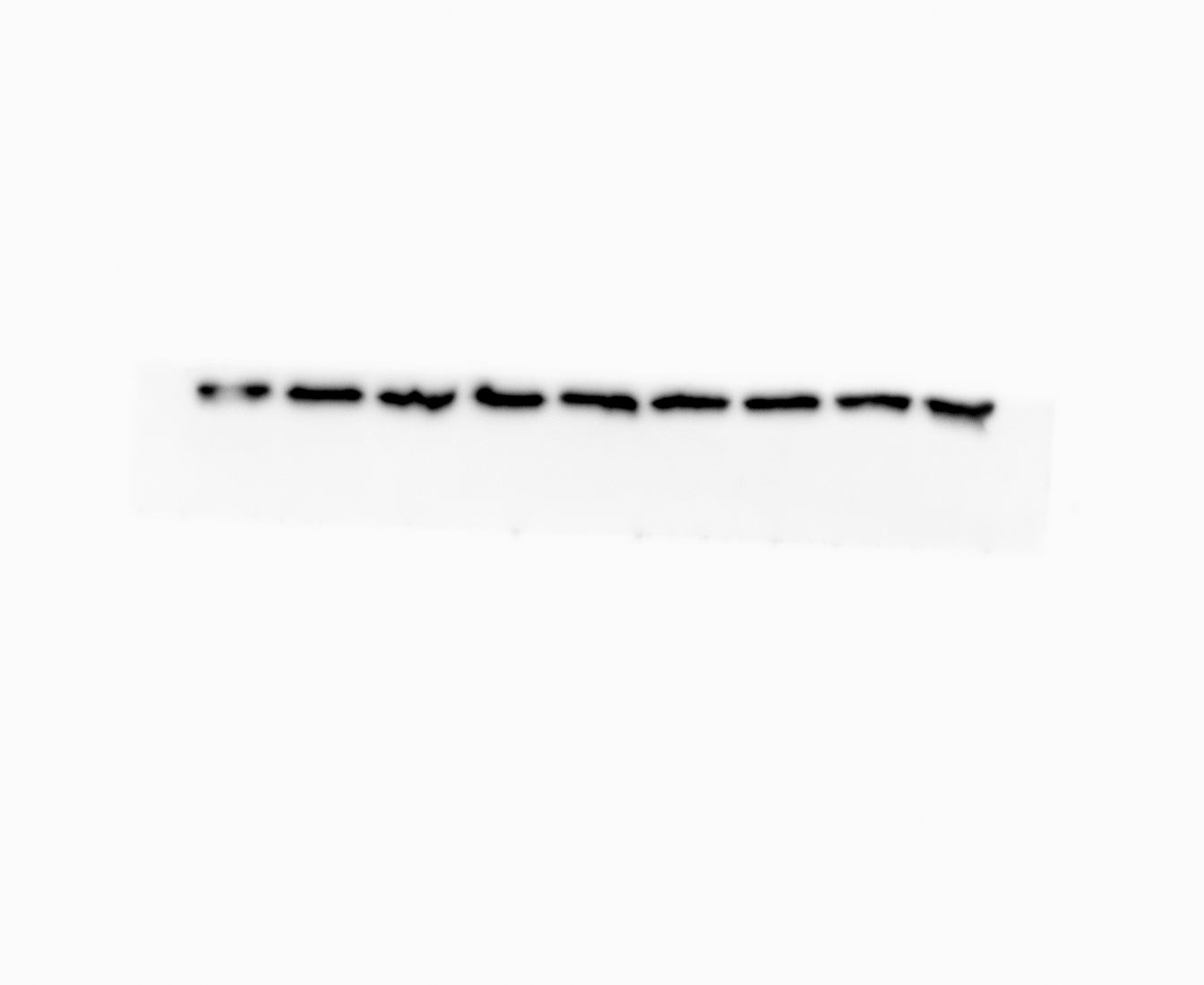

Supplement: Supplementary file 74 — Additional file 74. [file 13020_2026_1383_MOESM74_ESM.tif]

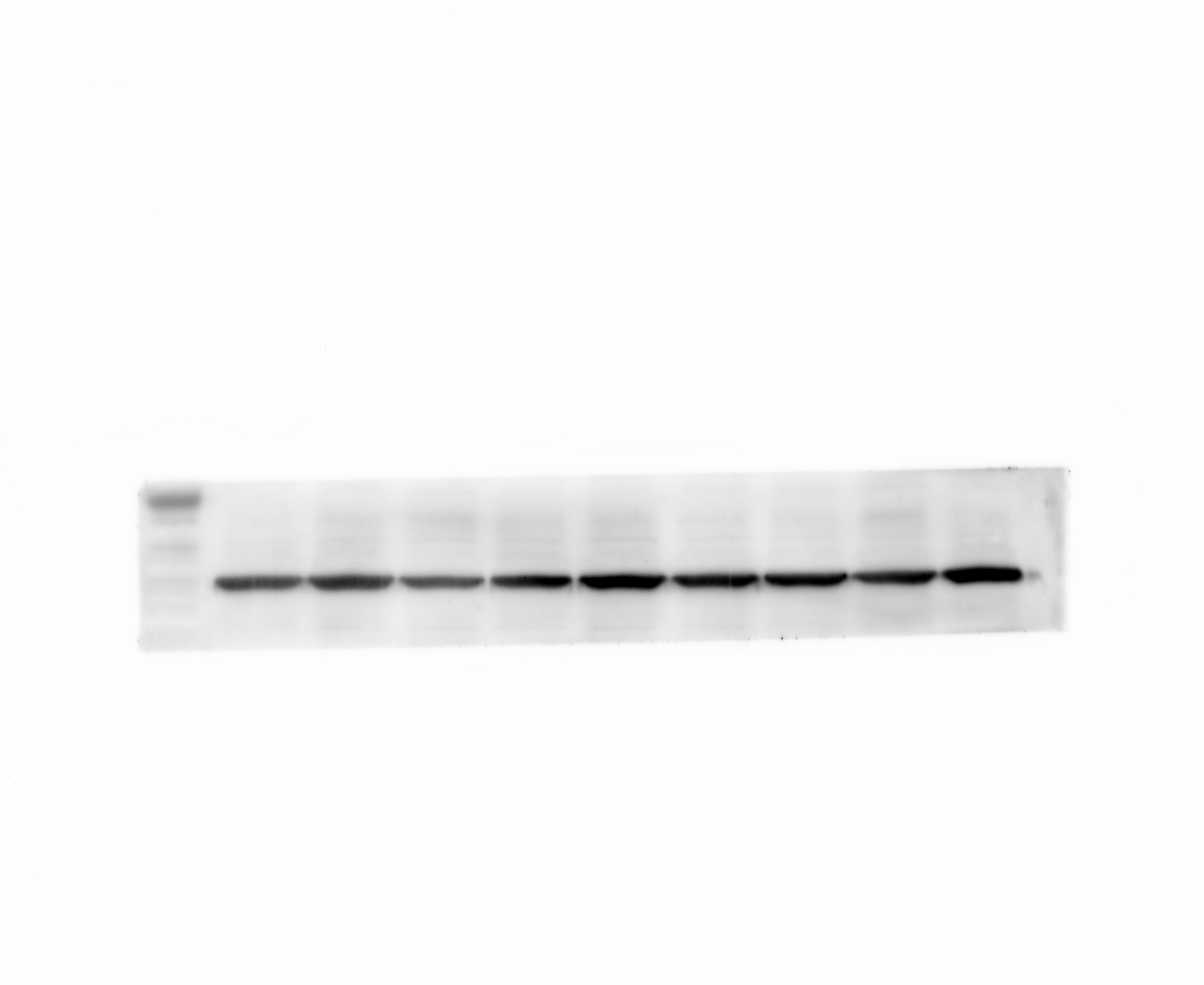

Supplement: Supplementary file 75 — Additional file 75. [file 13020_2026_1383_MOESM75_ESM.tif]

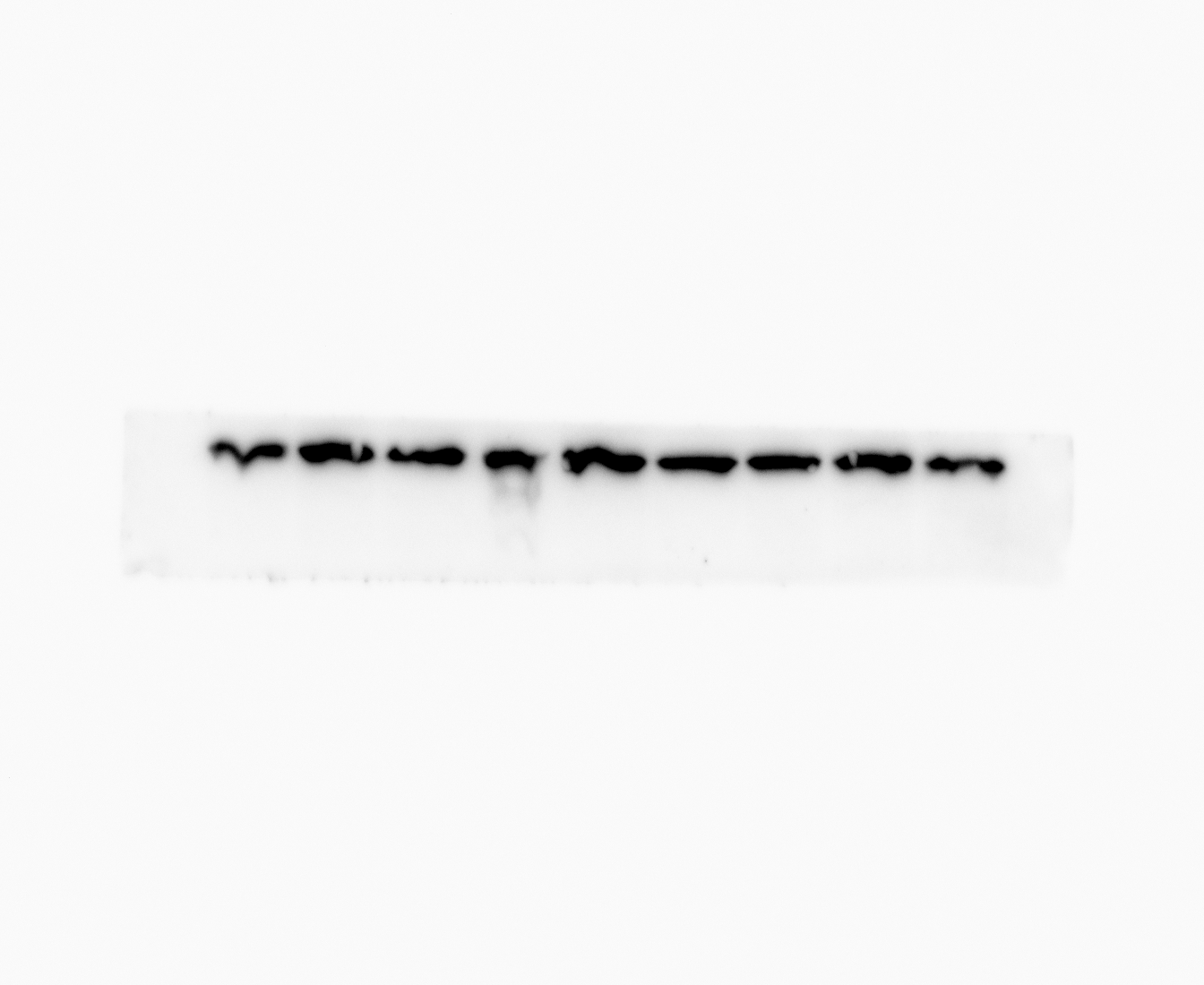

Supplement: Supplementary file 76 — Additional file 76. [file 13020_2026_1383_MOESM76_ESM.tif]

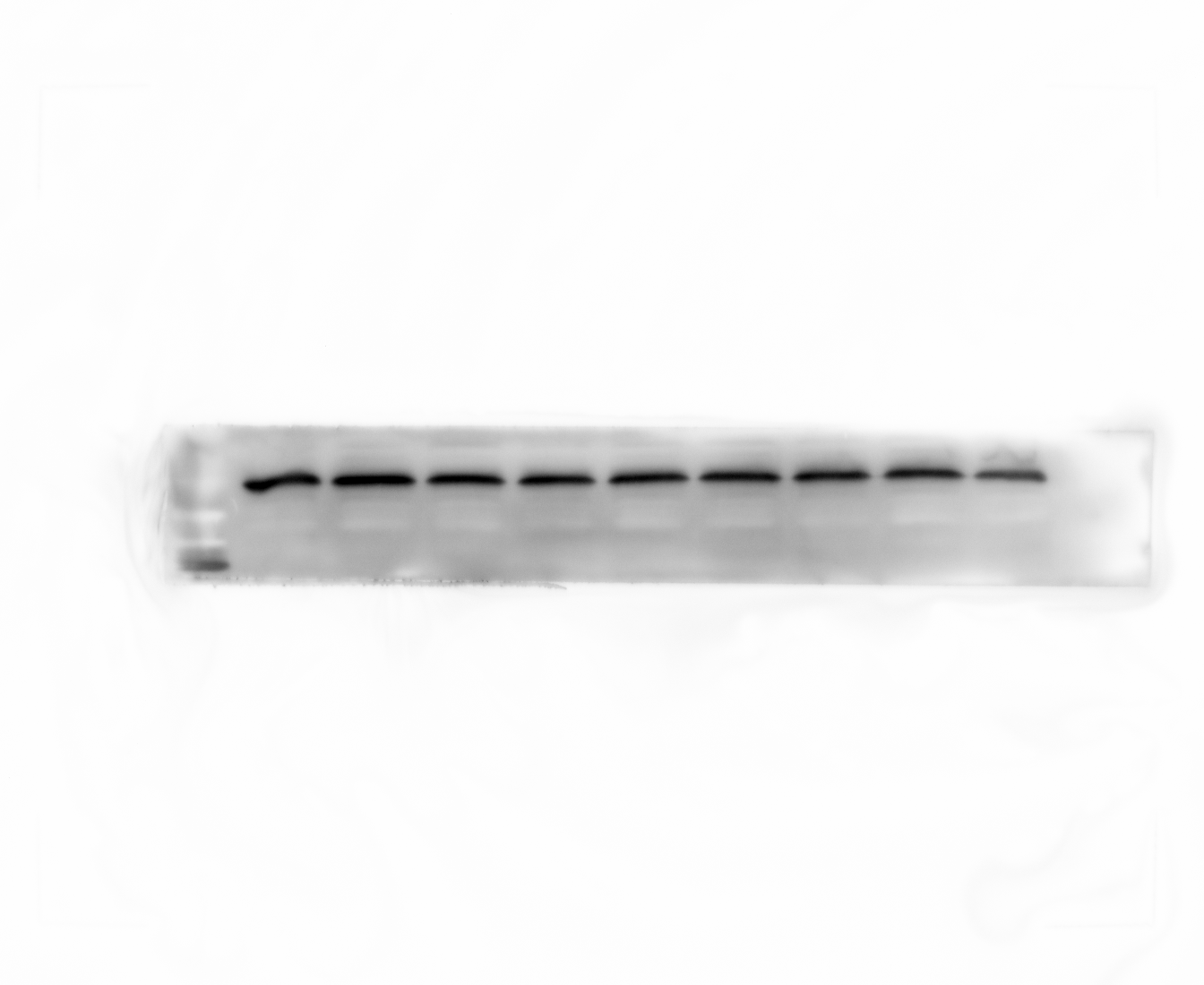

Supplement: Supplementary file 77 — Additional file 77. [file 13020_2026_1383_MOESM77_ESM.tif]

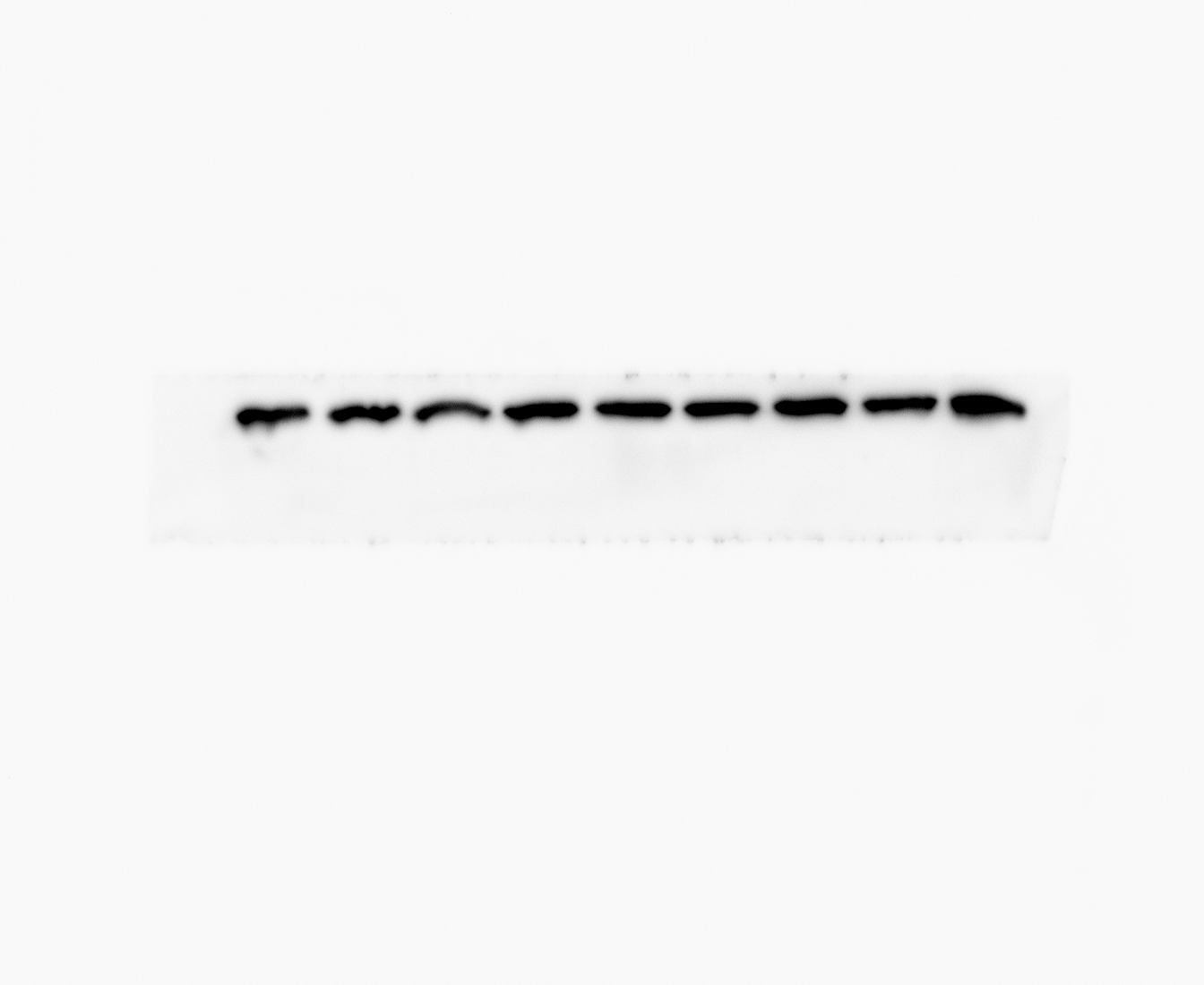

Supplement: Supplementary file 78 — Additional file 78. [file 13020_2026_1383_MOESM78_ESM.tif]

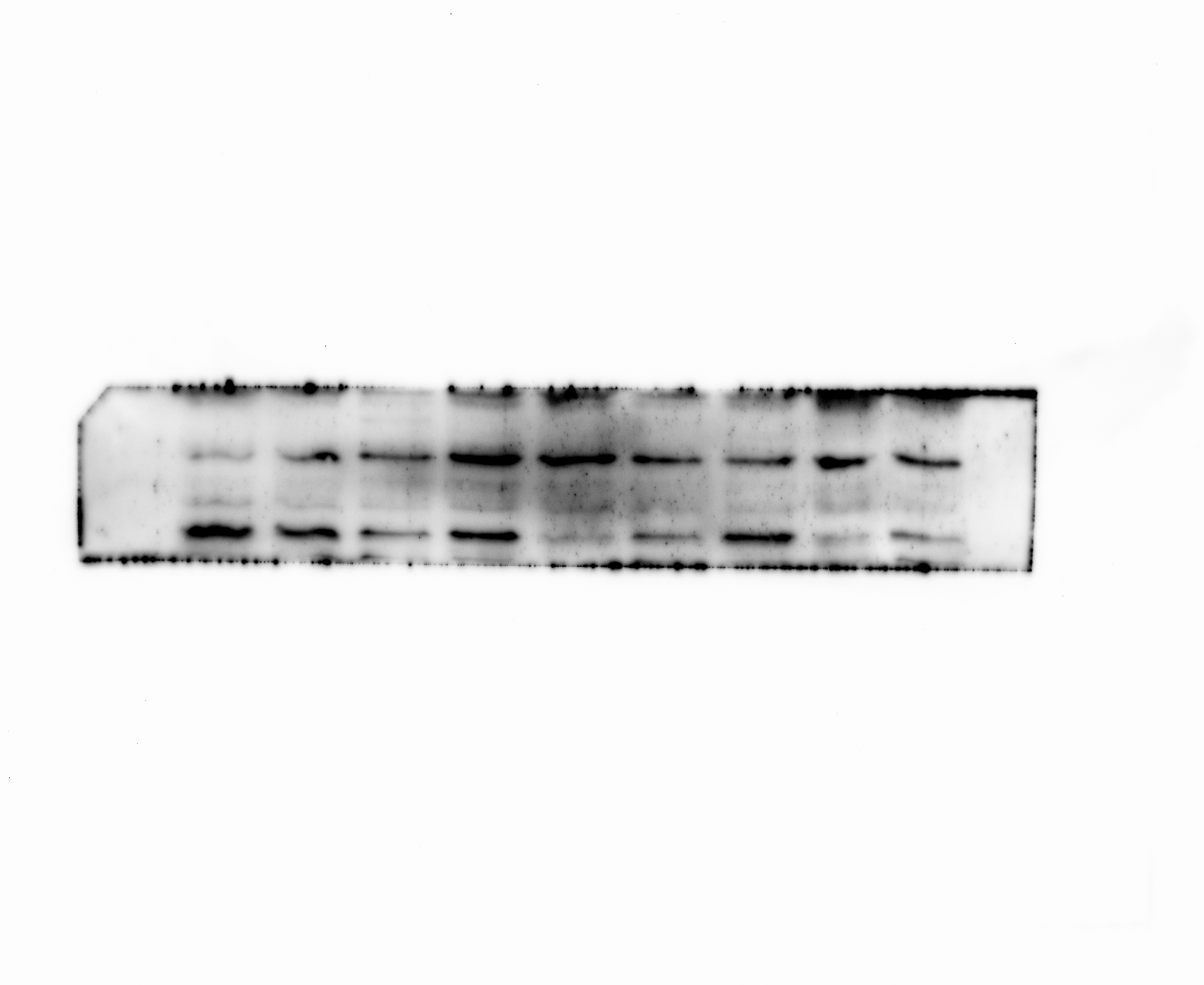

Supplement: Supplementary file 79 — Additional file 79. [file 13020_2026_1383_MOESM79_ESM.tif]

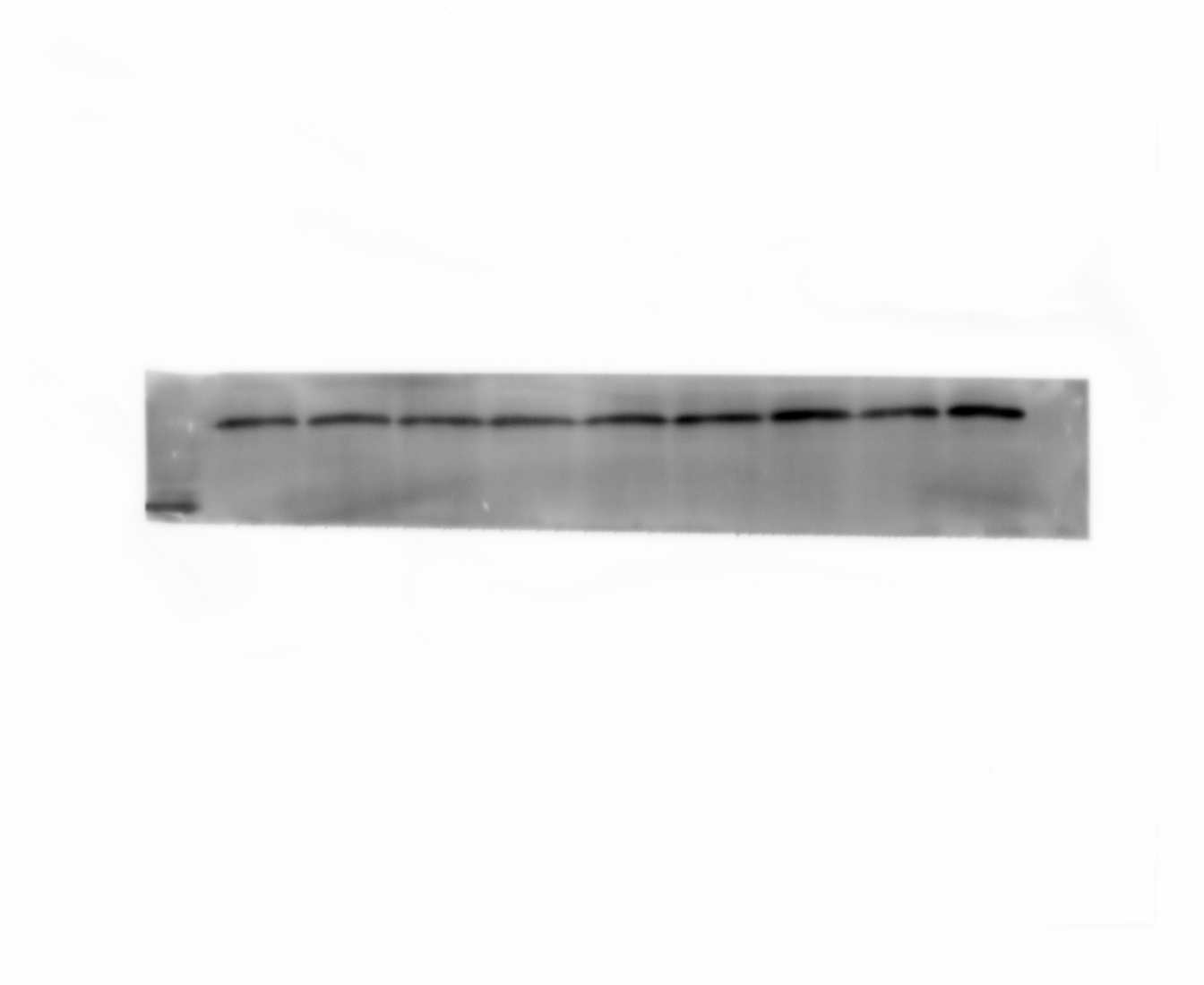

Supplement: Supplementary file 80 — Additional file 80. [file 13020_2026_1383_MOESM80_ESM.tif]

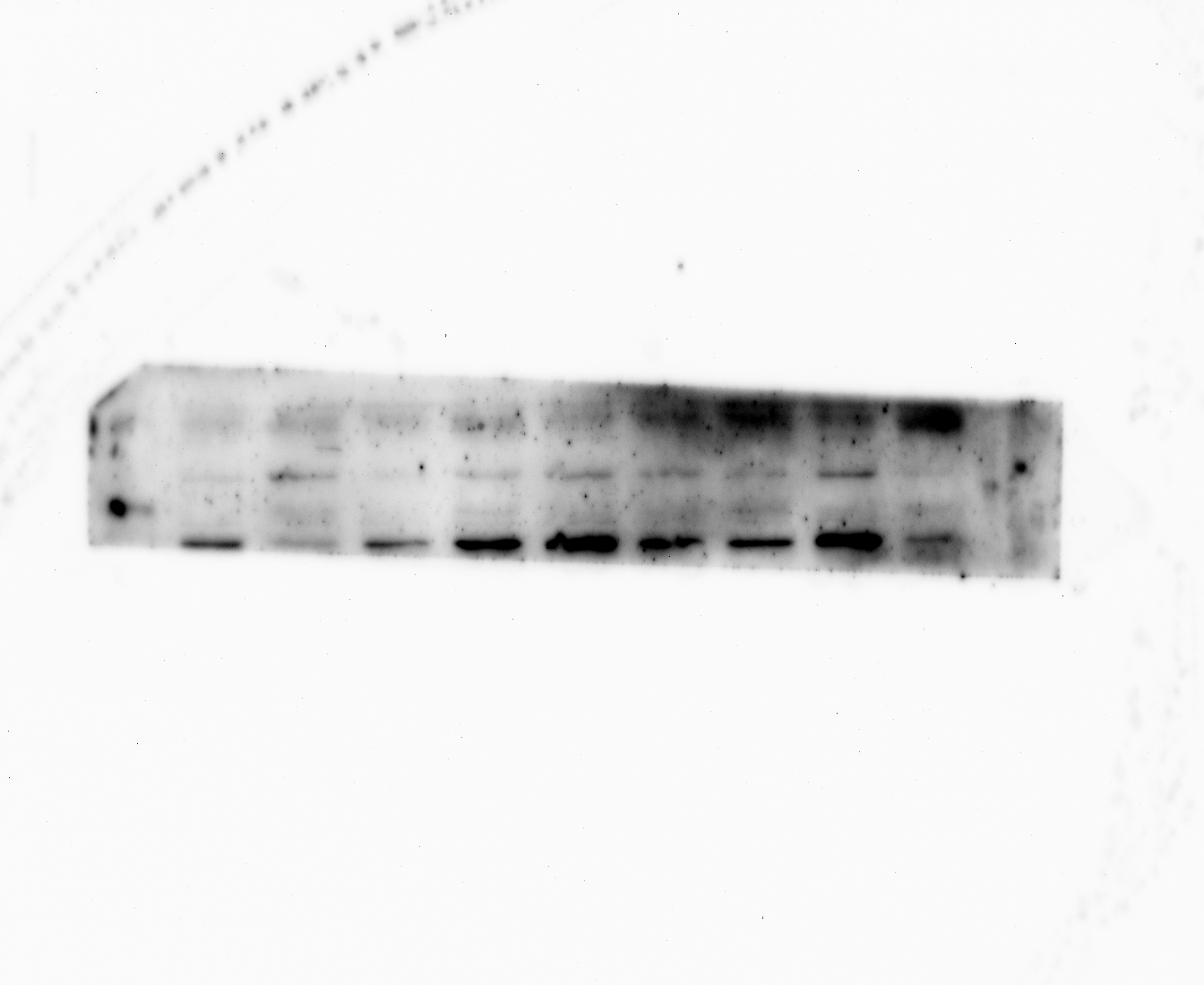

Supplement: Supplementary file 81 — Additional file 81. [file 13020_2026_1383_MOESM81_ESM.tif]

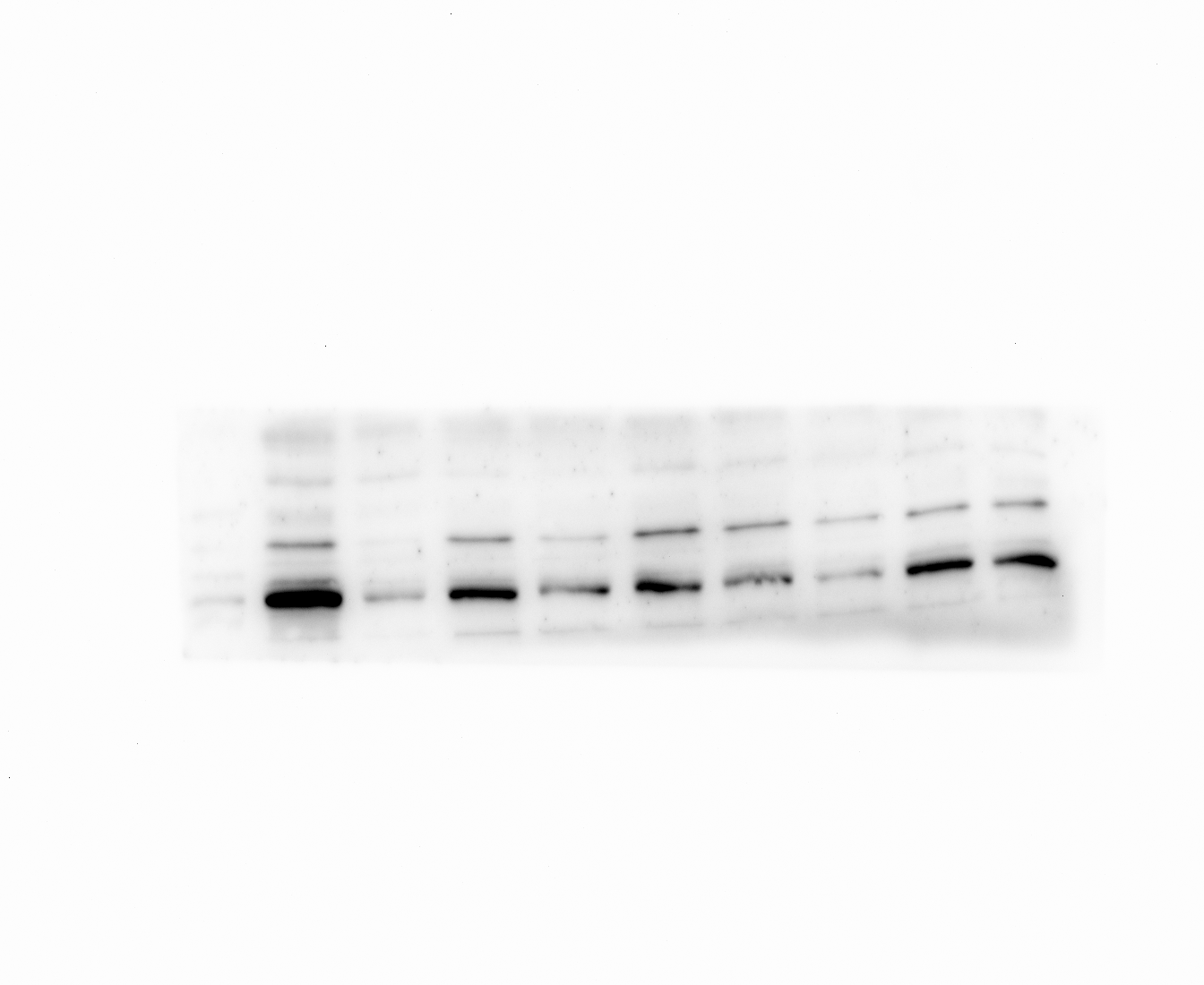

Supplement: Supplementary file 82 — Additional file 82. [file 13020_2026_1383_MOESM82_ESM.tif]

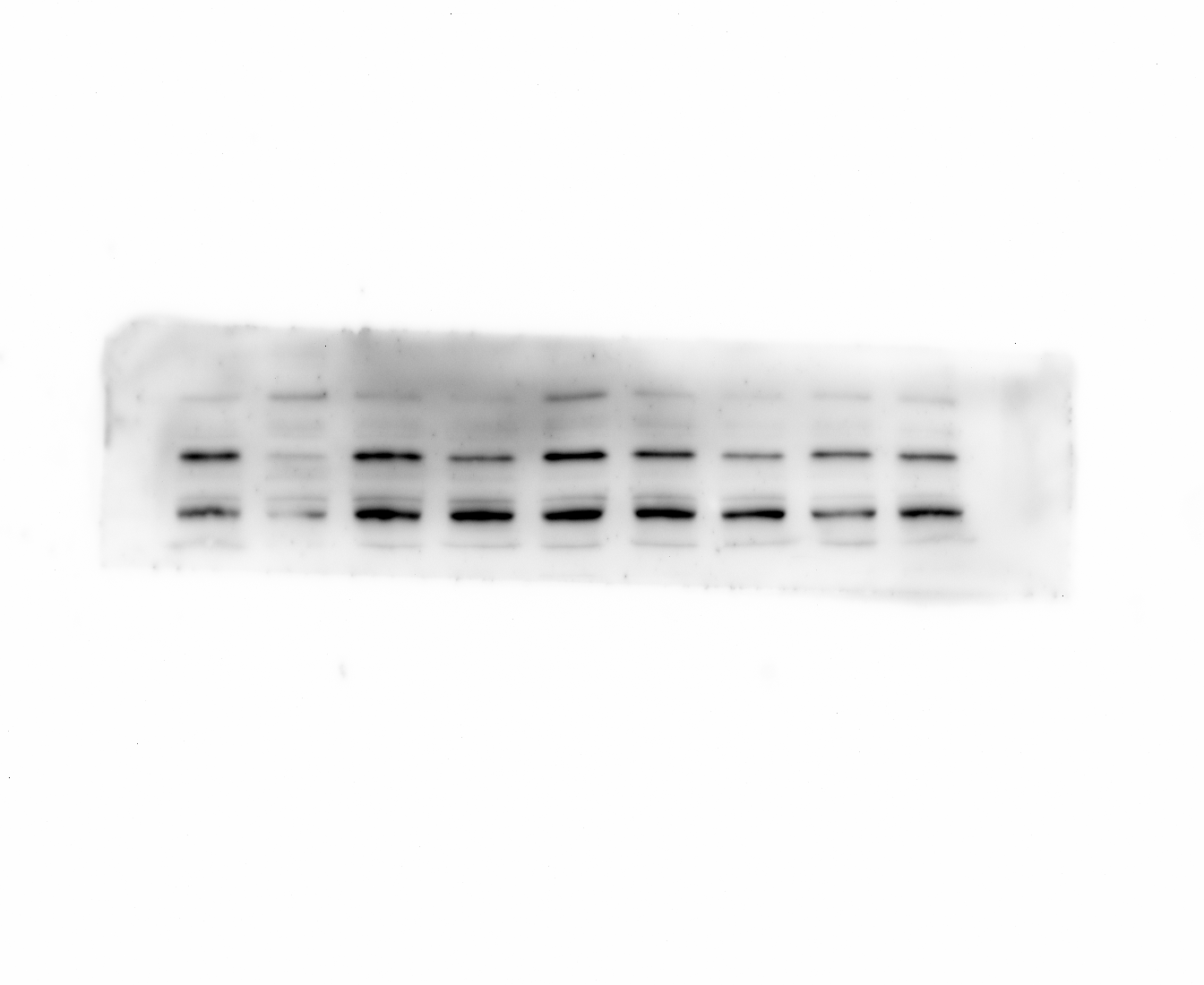

Supplement: Supplementary file 83 — Additional file 83. [file 13020_2026_1383_MOESM83_ESM.tif]

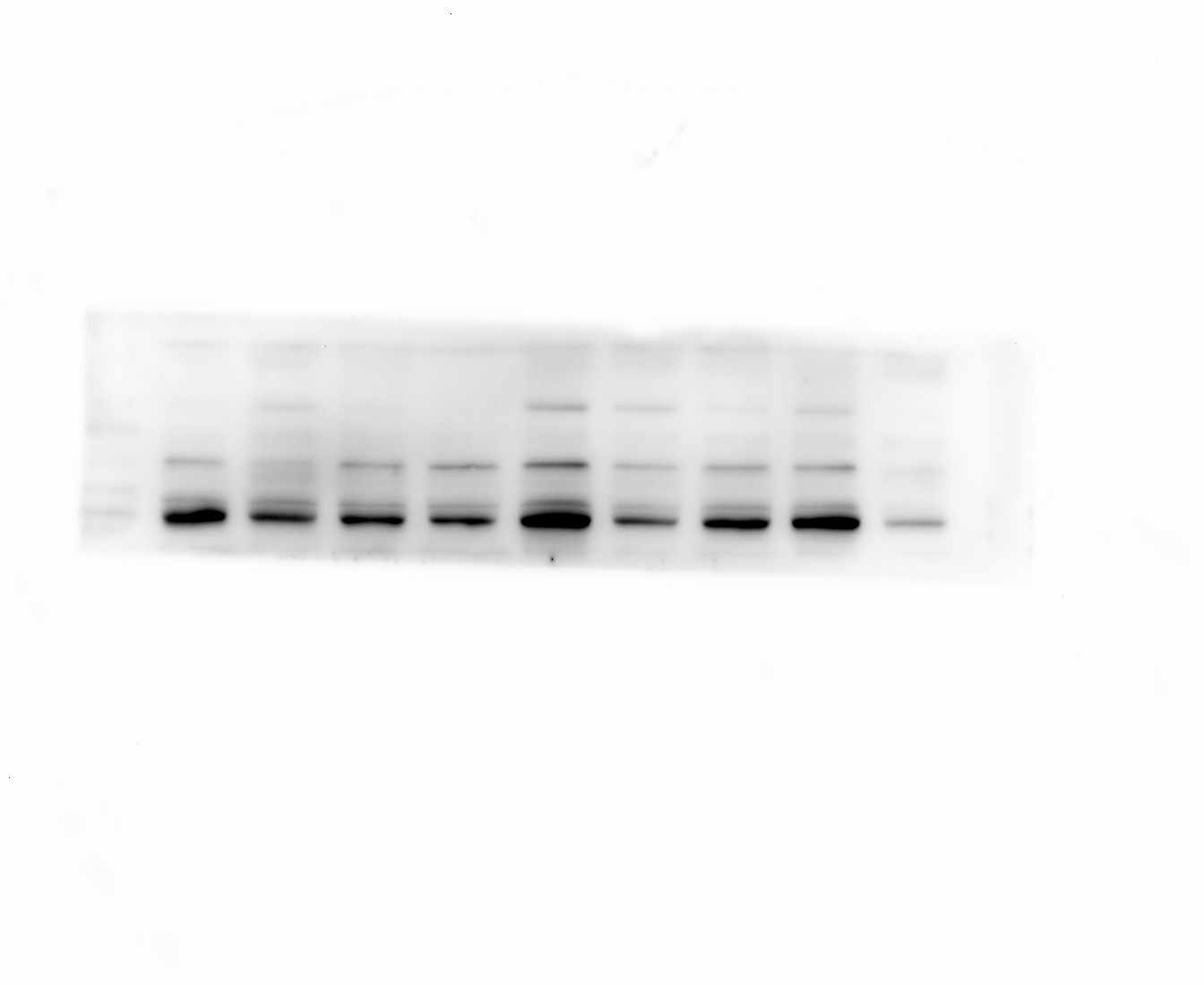

Supplement: Supplementary file 84 — Additional file 84. [file 13020_2026_1383_MOESM84_ESM.tif]

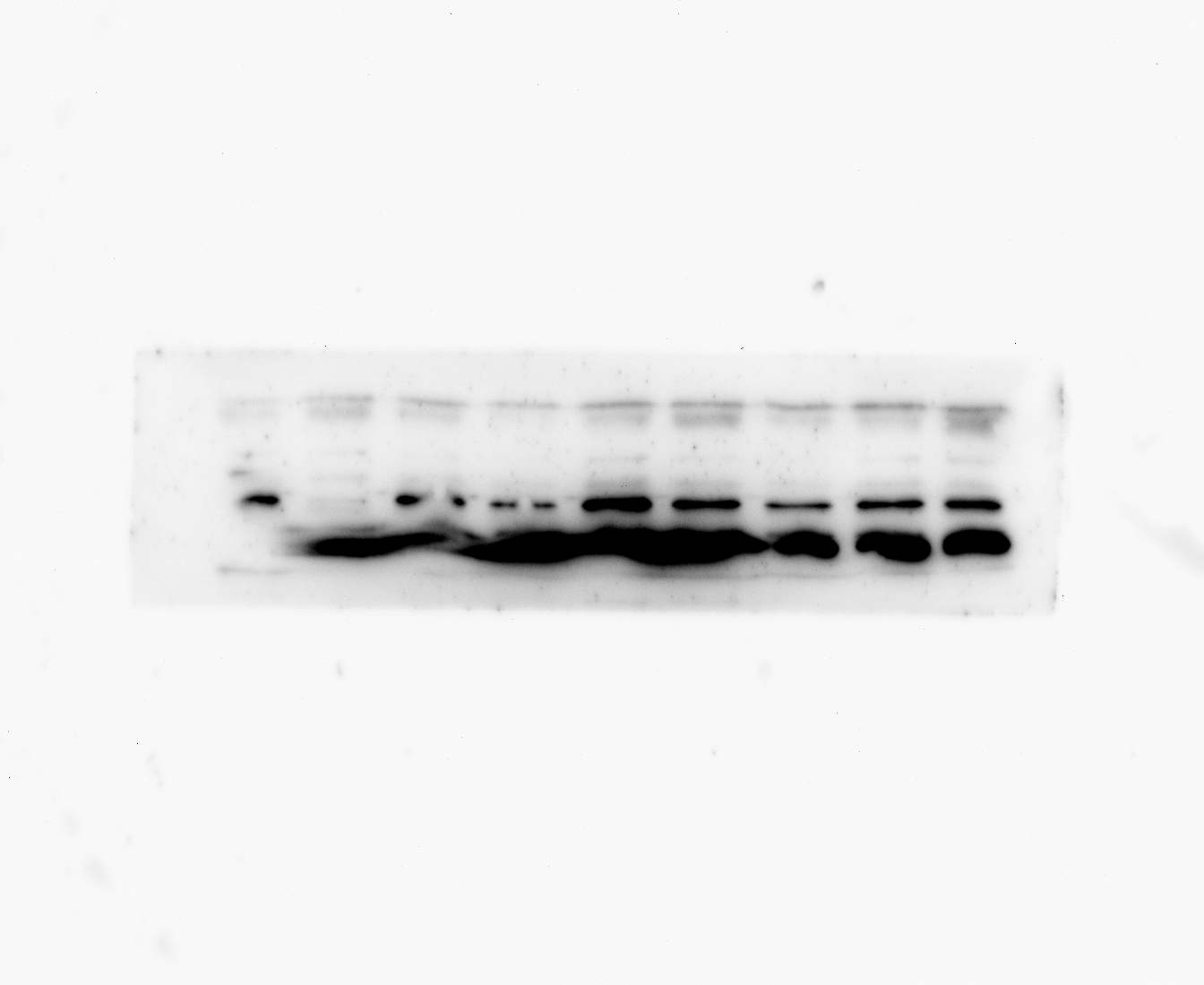

Supplement: Supplementary file 85 — Additional file 85. [file 13020_2026_1383_MOESM85_ESM.tif]

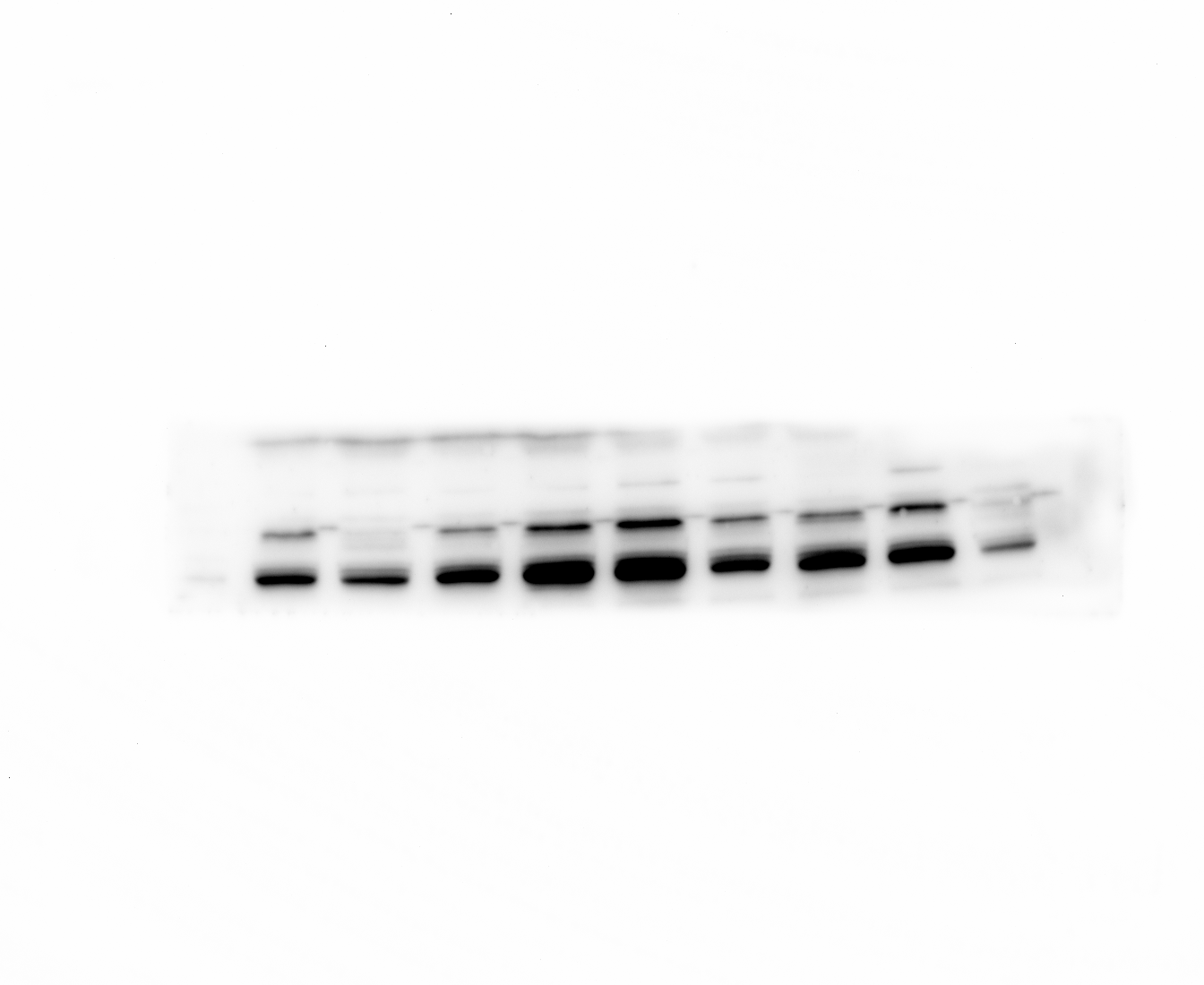

Supplement: Supplementary file 86 — Additional file 86. [file 13020_2026_1383_MOESM86_ESM.tif]

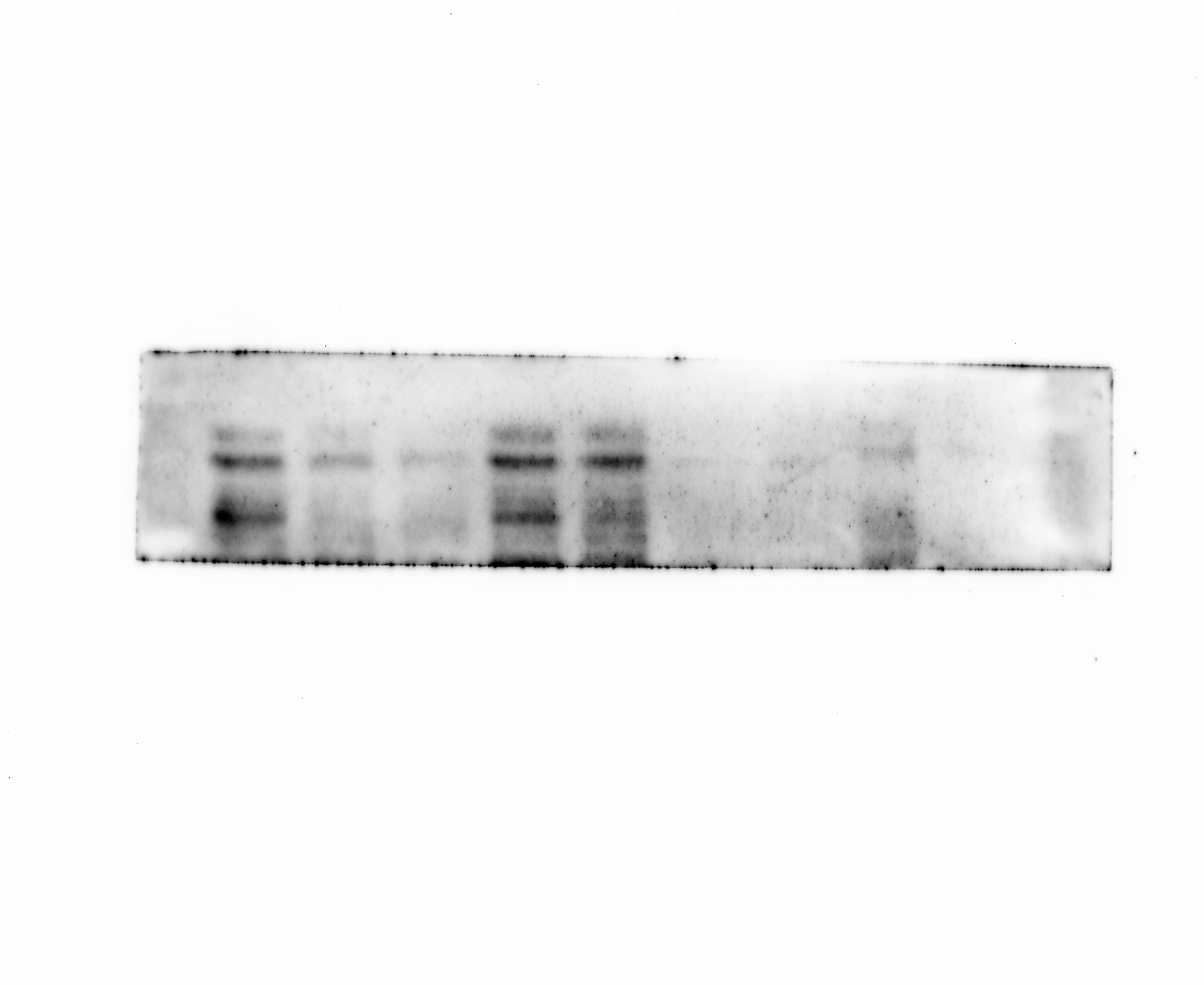

Supplement: Supplementary file 87 — Additional file 87. [file 13020_2026_1383_MOESM87_ESM.tif]

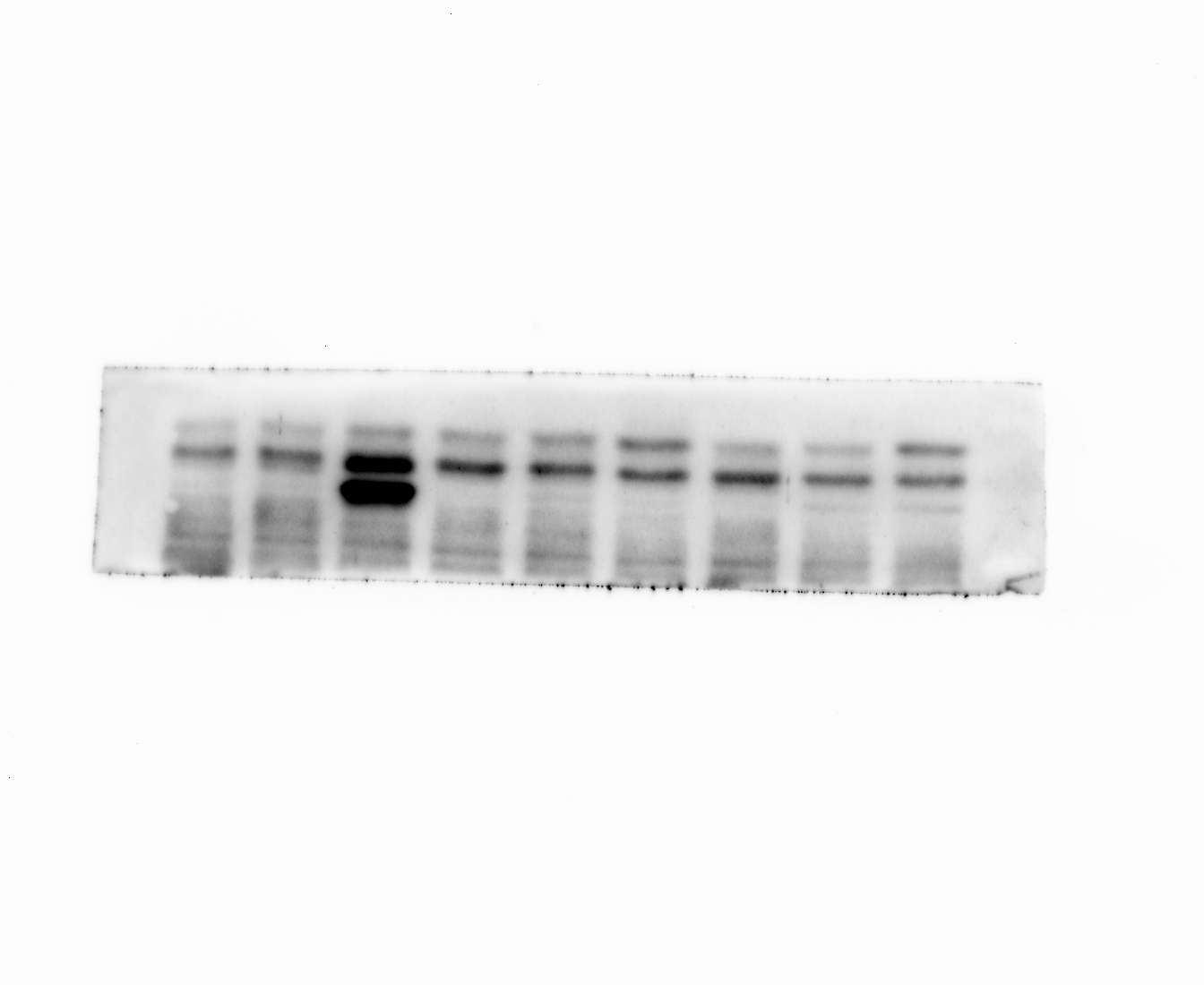

Supplement: Supplementary file 88 — Additional file 88. [file 13020_2026_1383_MOESM88_ESM.tif]

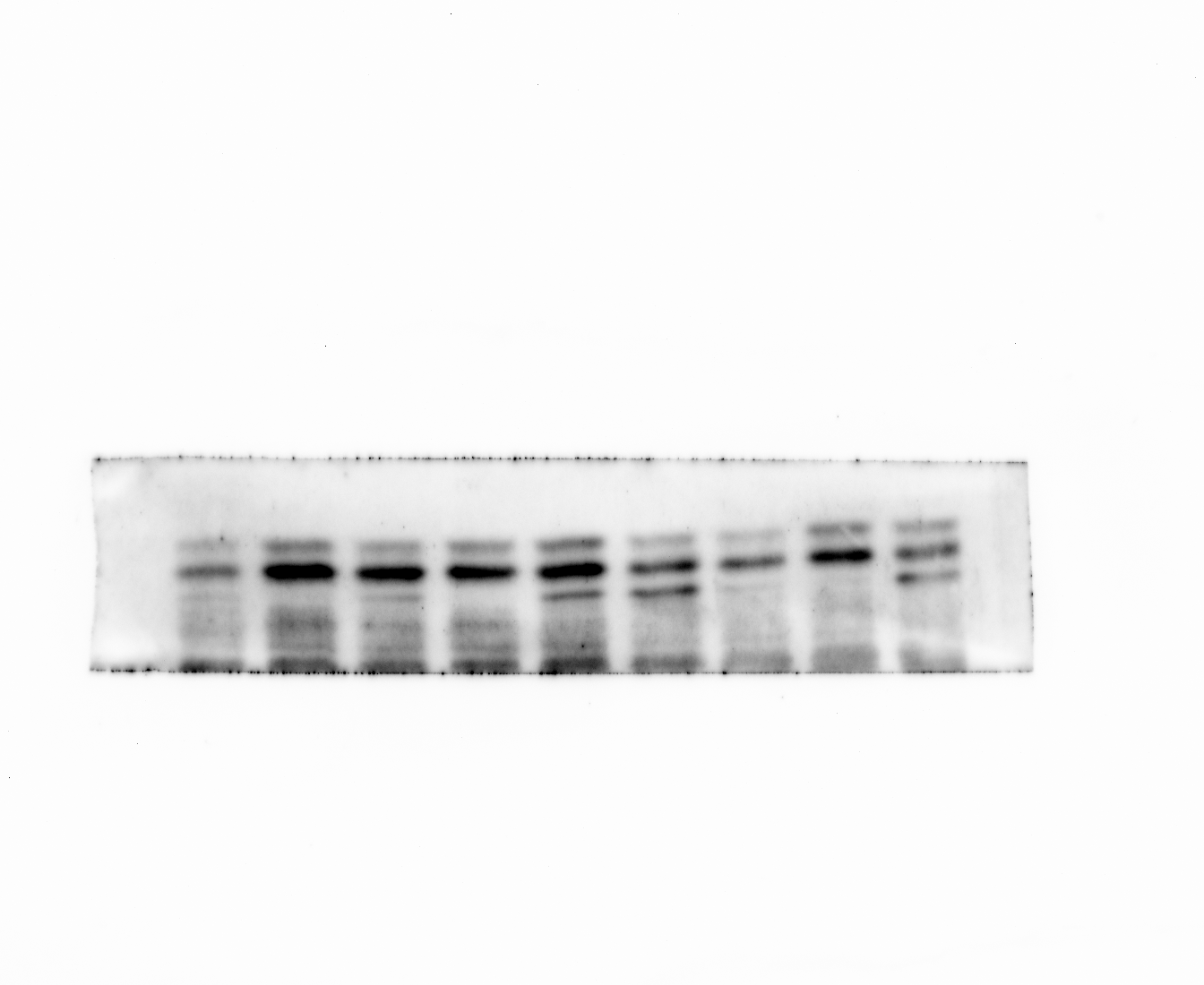

Supplement: Supplementary file 89 — Additional file 89. [file 13020_2026_1383_MOESM89_ESM.tif]

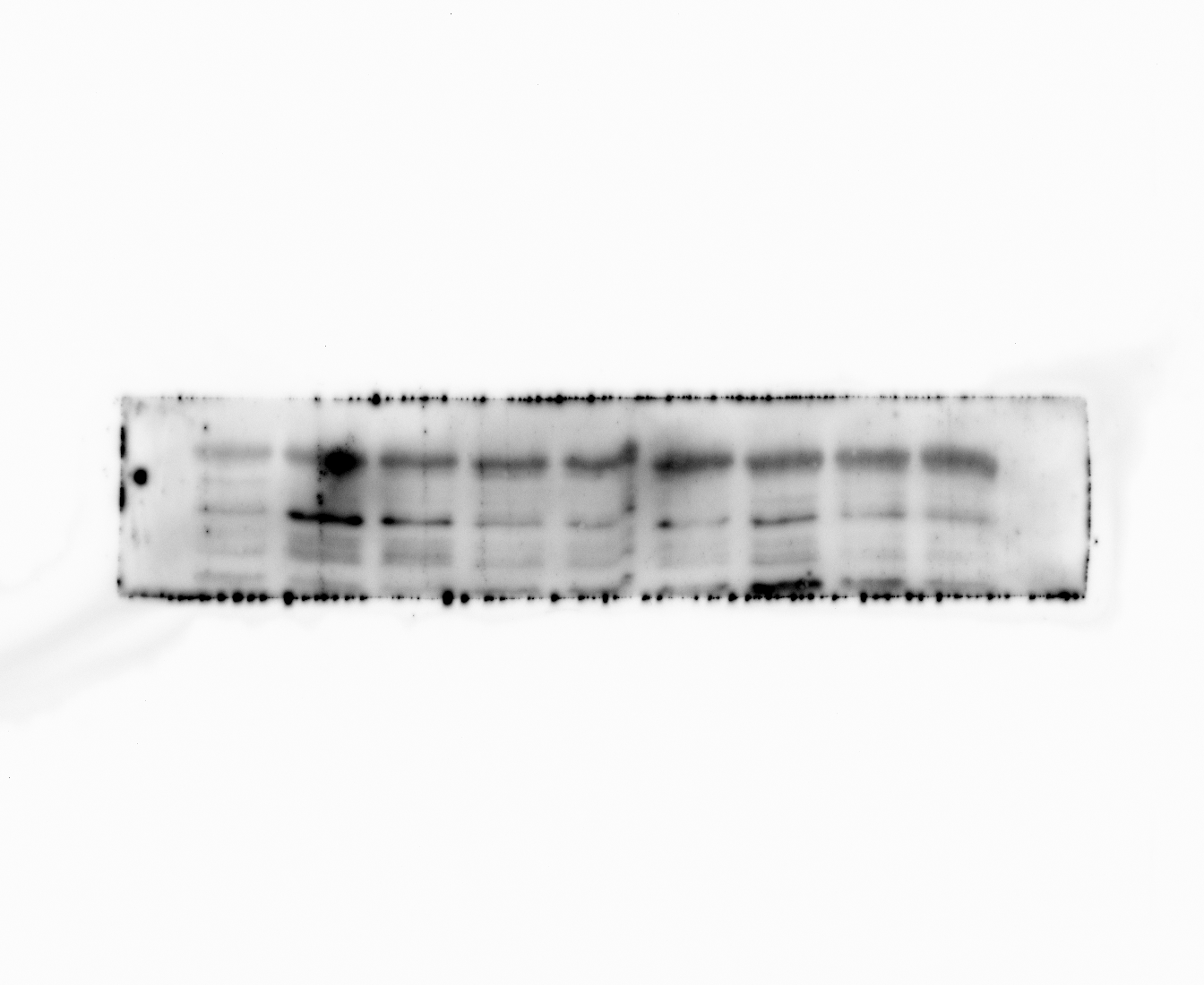

Supplement: Supplementary file 90 — Additional file 90. [file 13020_2026_1383_MOESM90_ESM.tif]

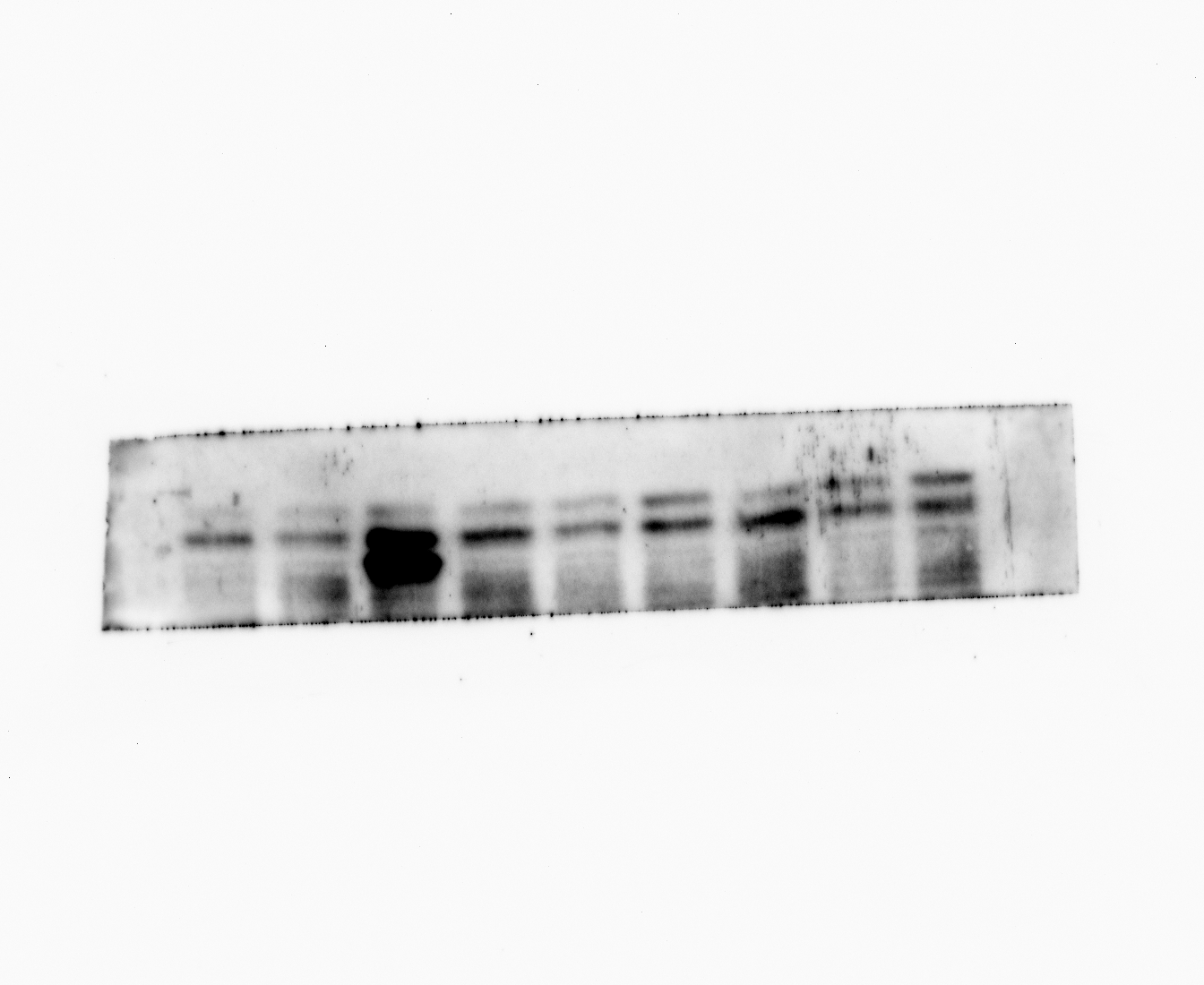

Supplement: Supplementary file 91 — Additional file 91. [file 13020_2026_1383_MOESM91_ESM.tif]

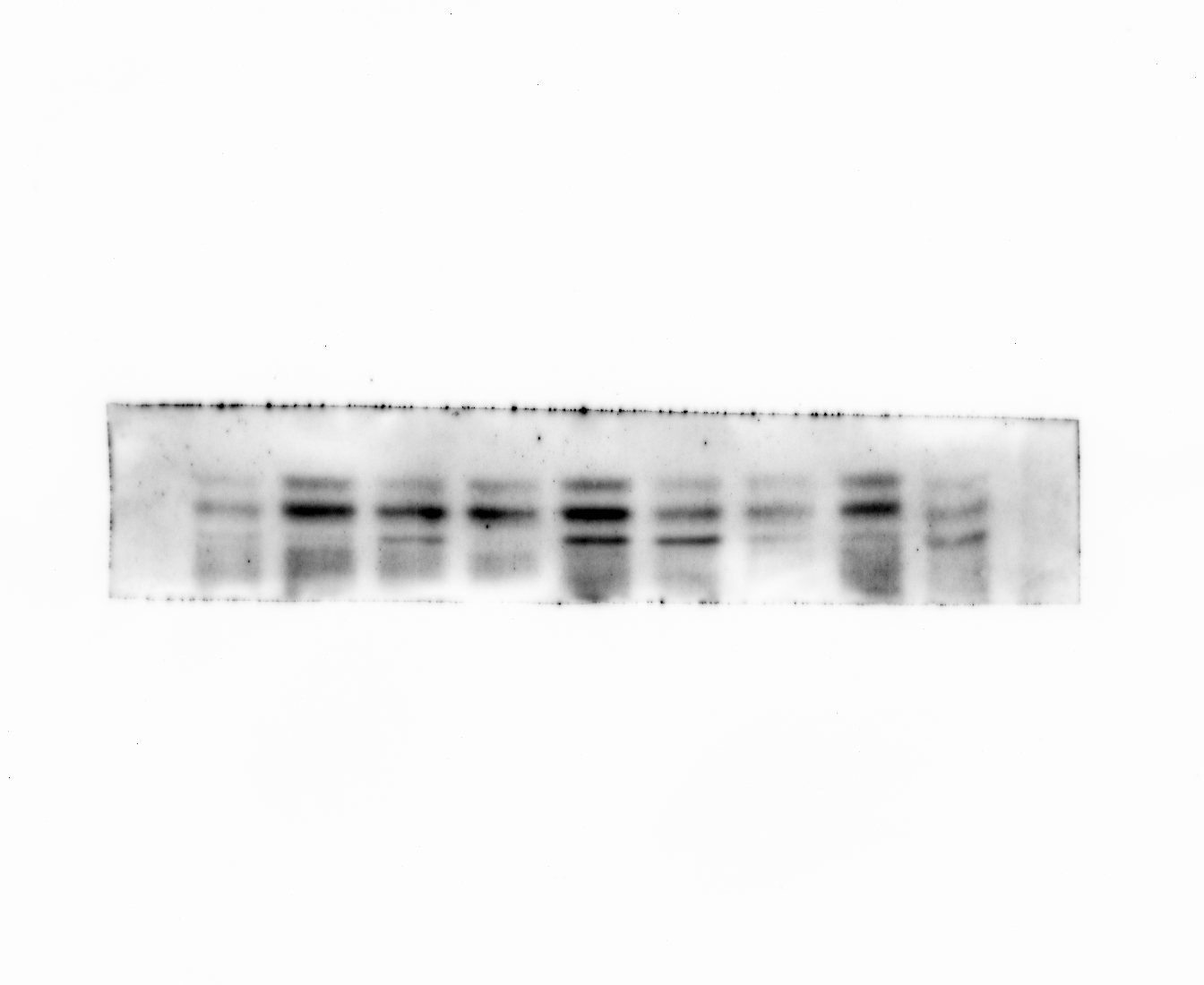

Supplement: Supplementary file 92 — Additional file 92. [file 13020_2026_1383_MOESM92_ESM.tif]

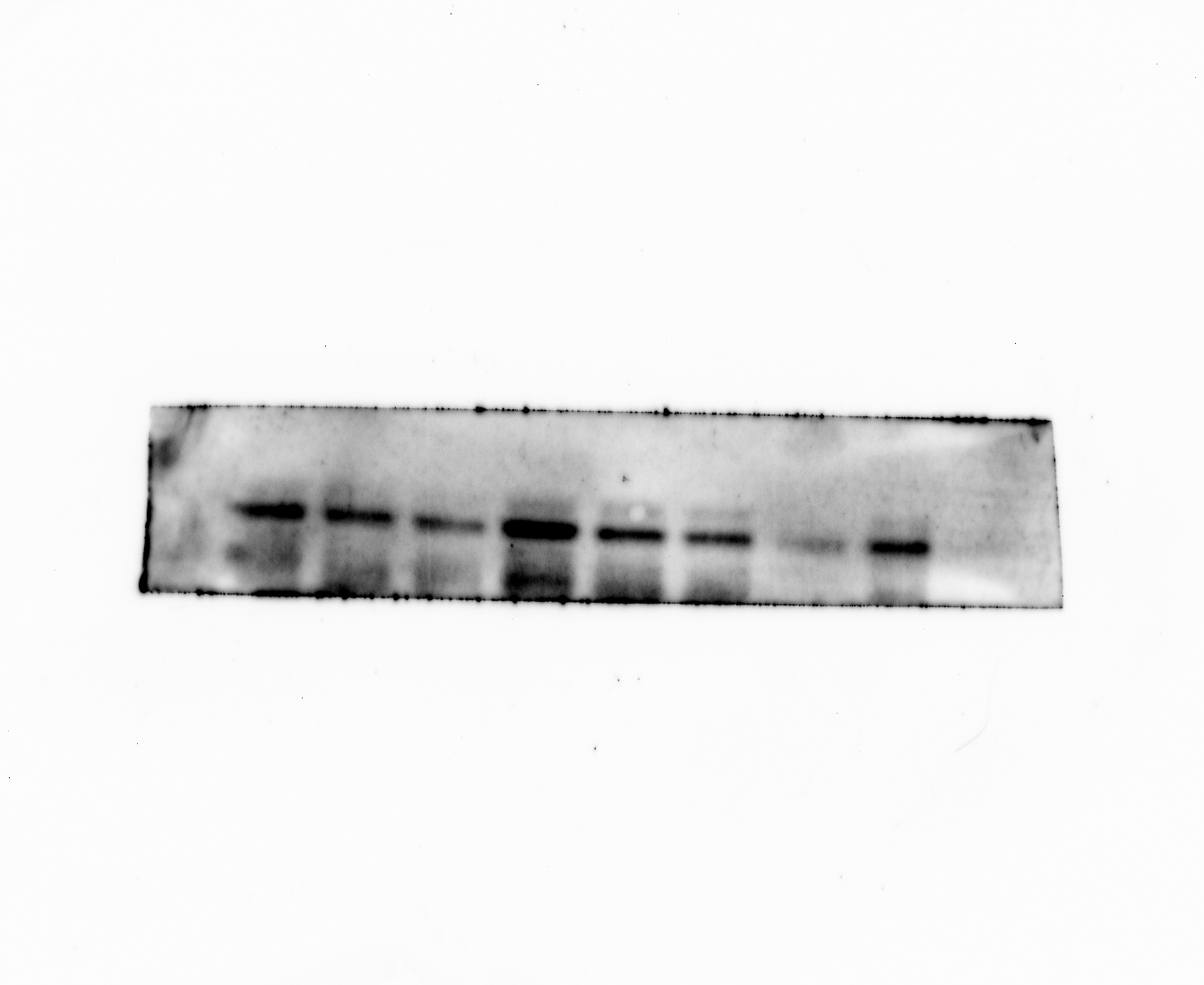

Supplement: Supplementary file 93 — Additional file 93. [file 13020_2026_1383_MOESM93_ESM.tif]

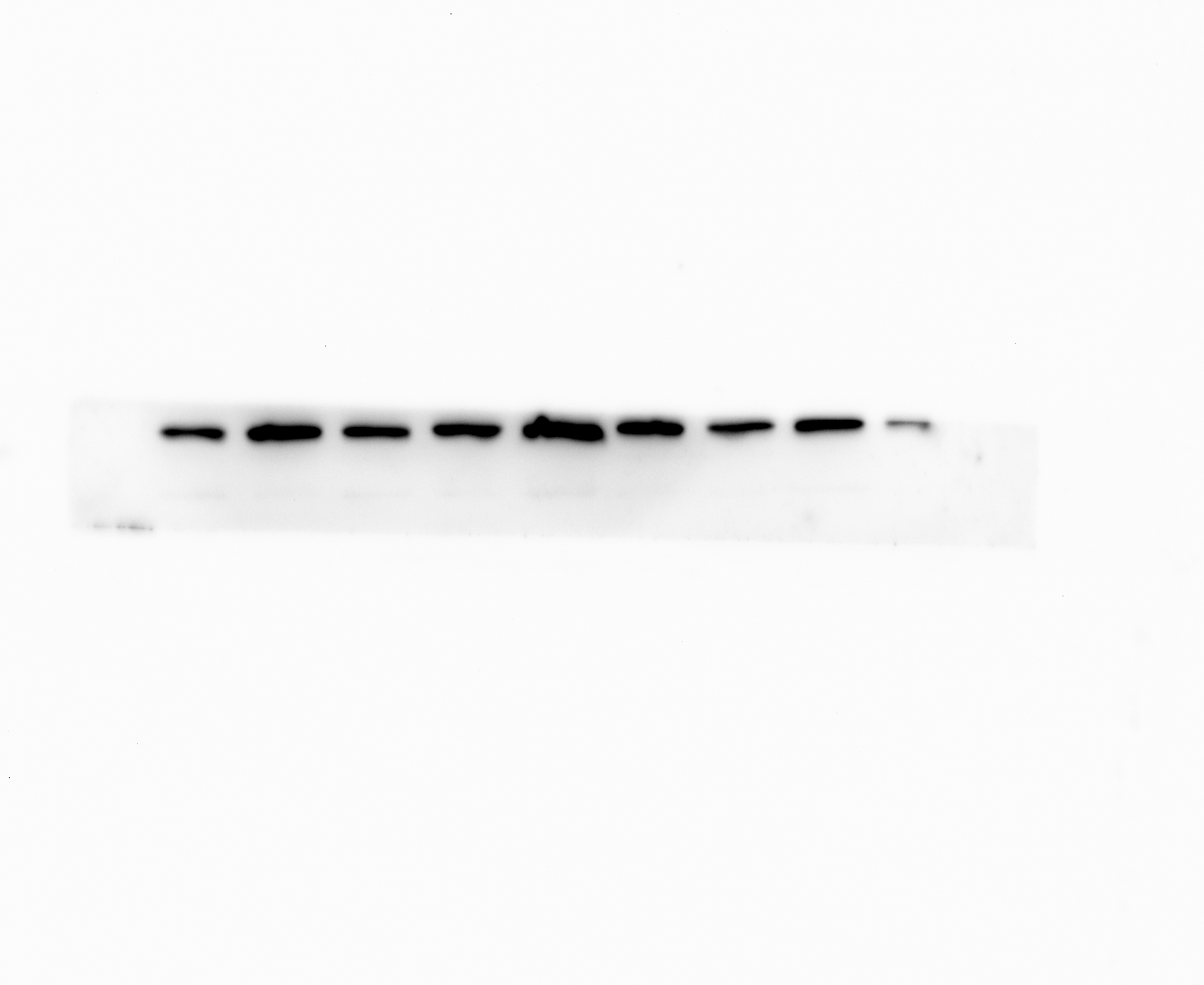

Supplement: Supplementary file 94 — Additional file 94. [file 13020_2026_1383_MOESM94_ESM.tif]

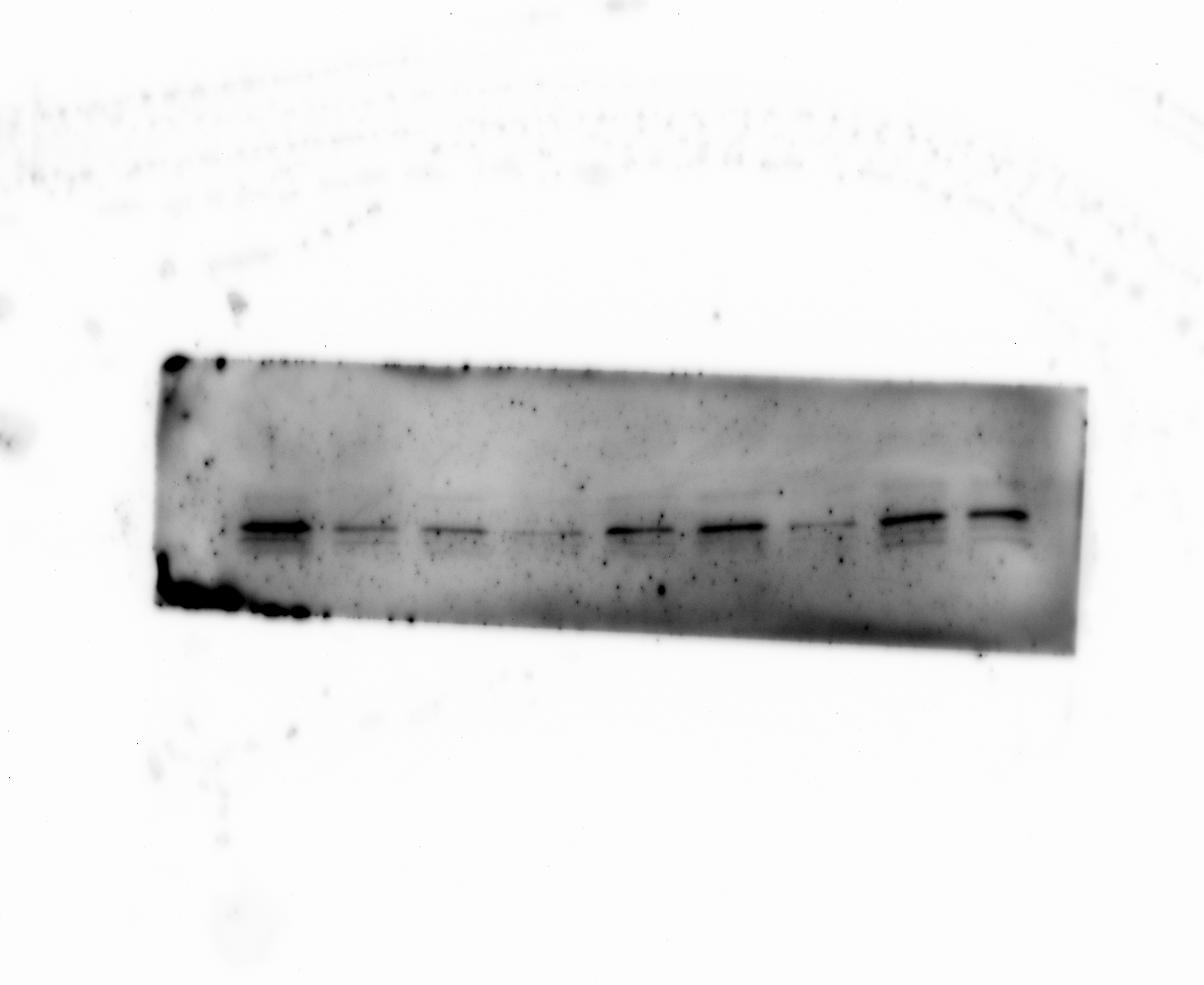

Supplement: Supplementary file 95 — Additional file 95. [file 13020_2026_1383_MOESM95_ESM.tif]

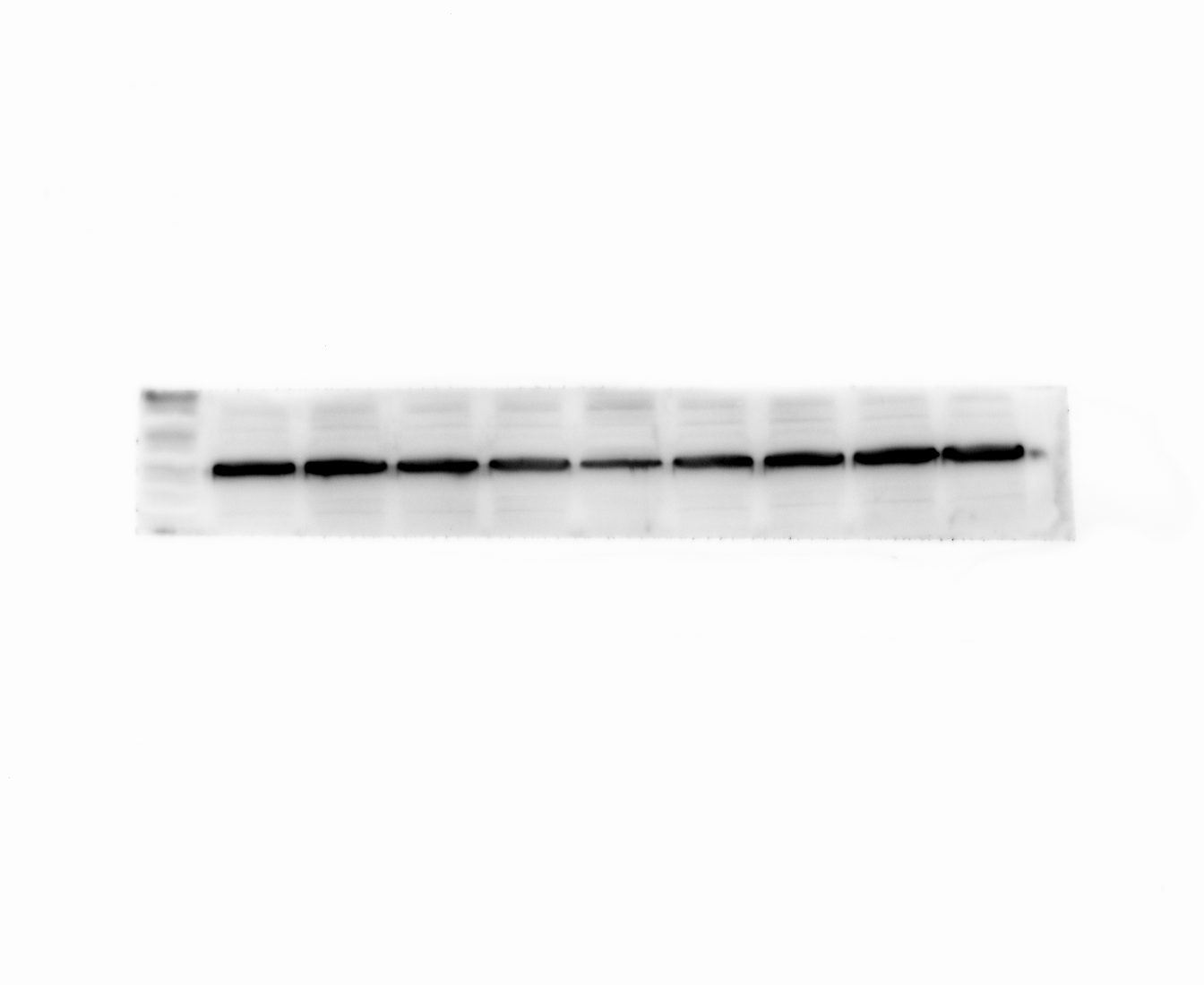

Supplement: Supplementary file 96 — Additional file 96. [file 13020_2026_1383_MOESM96_ESM.tif]

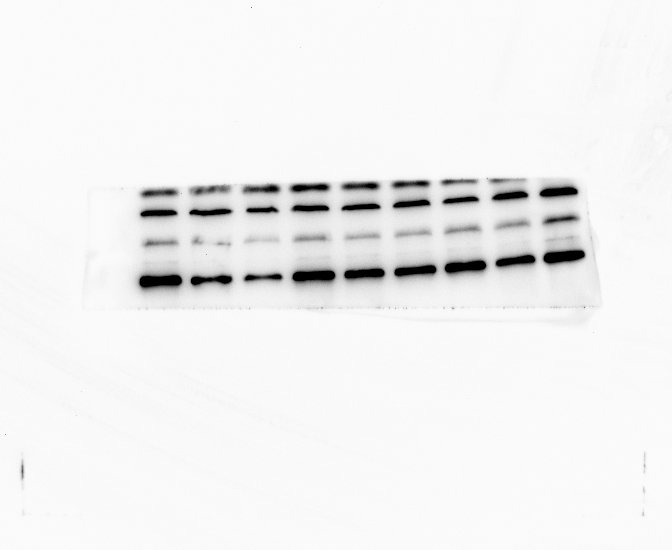

Supplement: Supplementary file 97 — Additional file 97. [file 13020_2026_1383_MOESM97_ESM.tif]

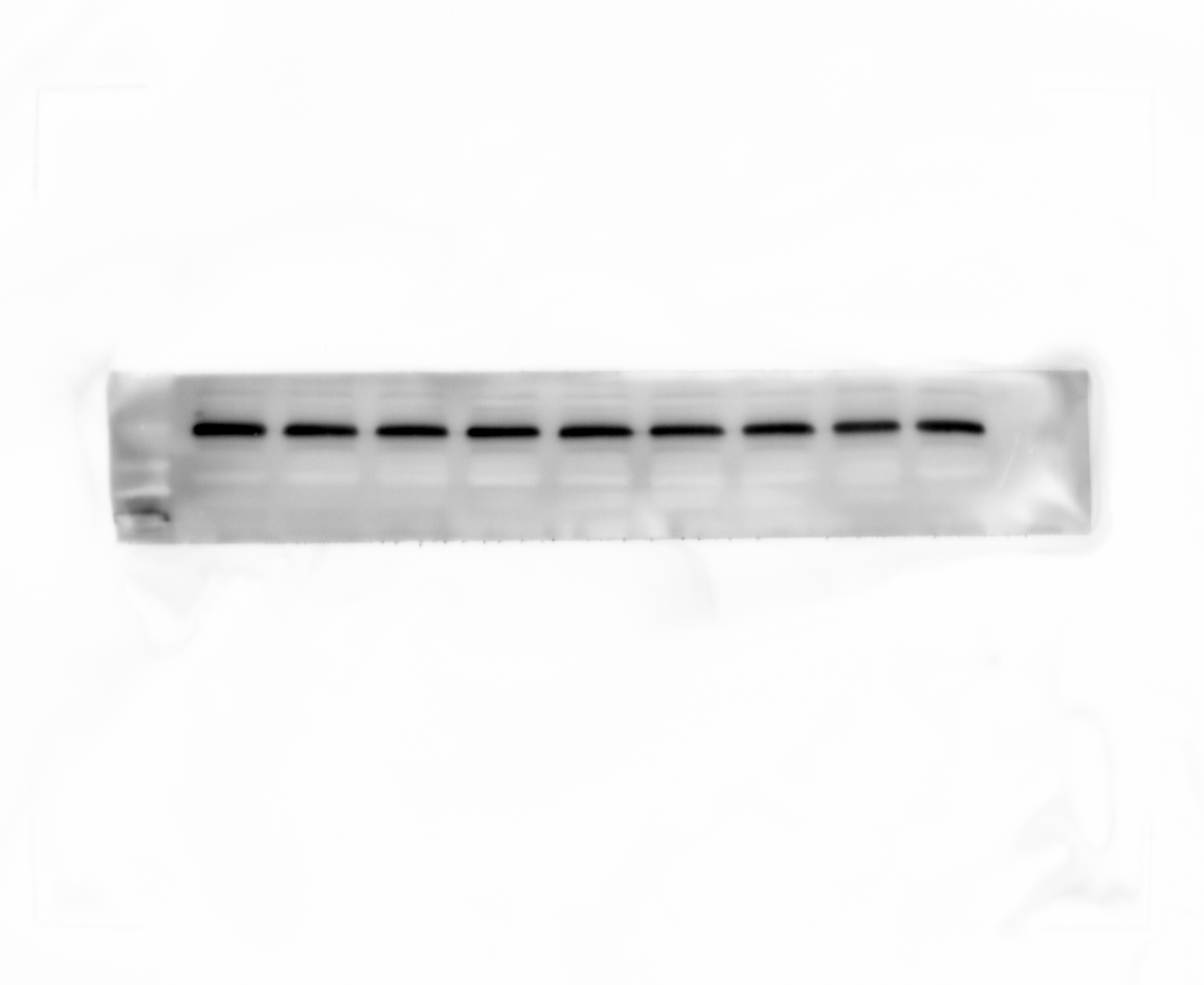

Supplement: Supplementary file 98 — Additional file 98. [file 13020_2026_1383_MOESM98_ESM.tif]

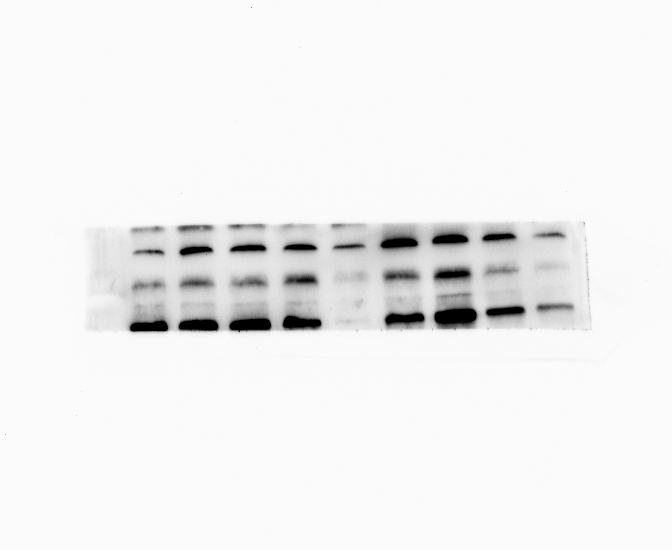

Supplement: Supplementary file 99 — Additional file 99. [file 13020_2026_1383_MOESM99_ESM.tif]

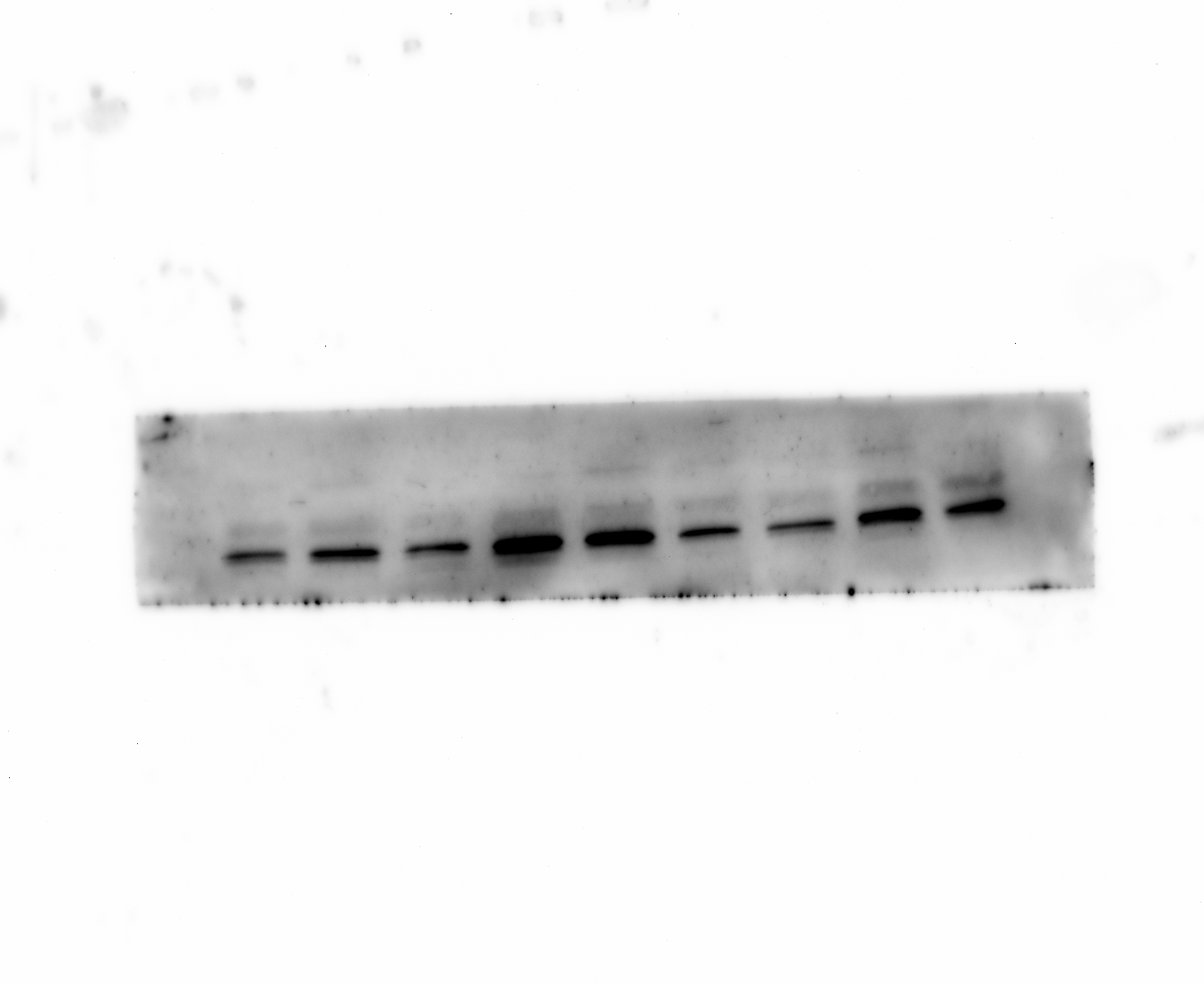

Supplement: Supplementary file 100 — Additional file 100. [file 13020_2026_1383_MOESM100_ESM.tif]
