# Supplementary figures and images for: Electroacupuncture inhibited neuronal apoptosis through PGAM5/FUNDC1-dependent mitophagy after ischemic stroke (part 2 of 2)
Source: Chin Med. 2026 Apr 3;21:110. doi: 10.1186/s13020-026-01383-3 (PMC13047806; doi:10.1186/s13020-026-01383-3)

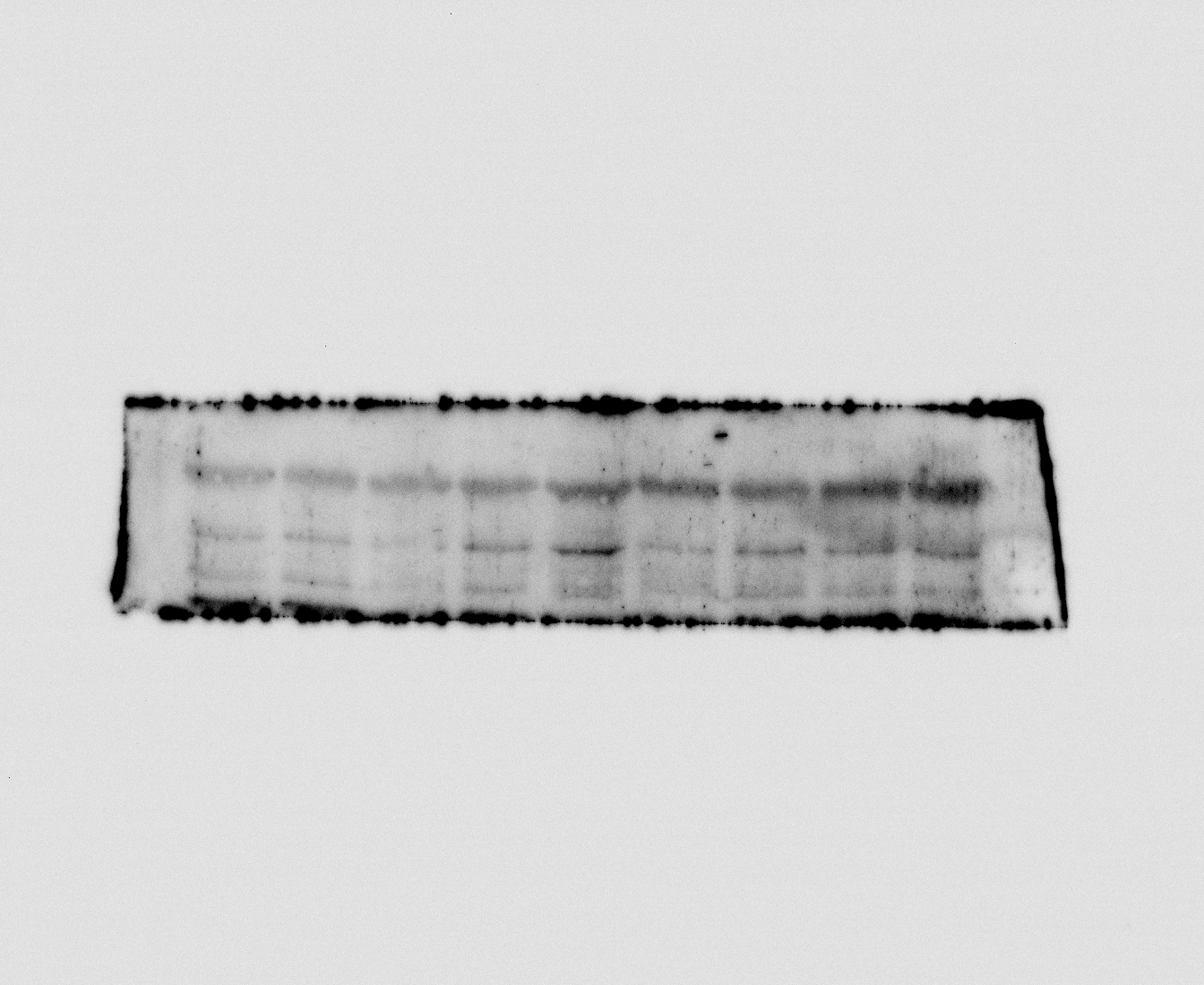

Supplement: Supplementary file 101 — Additional file 101. [file 13020_2026_1383_MOESM101_ESM.tif]

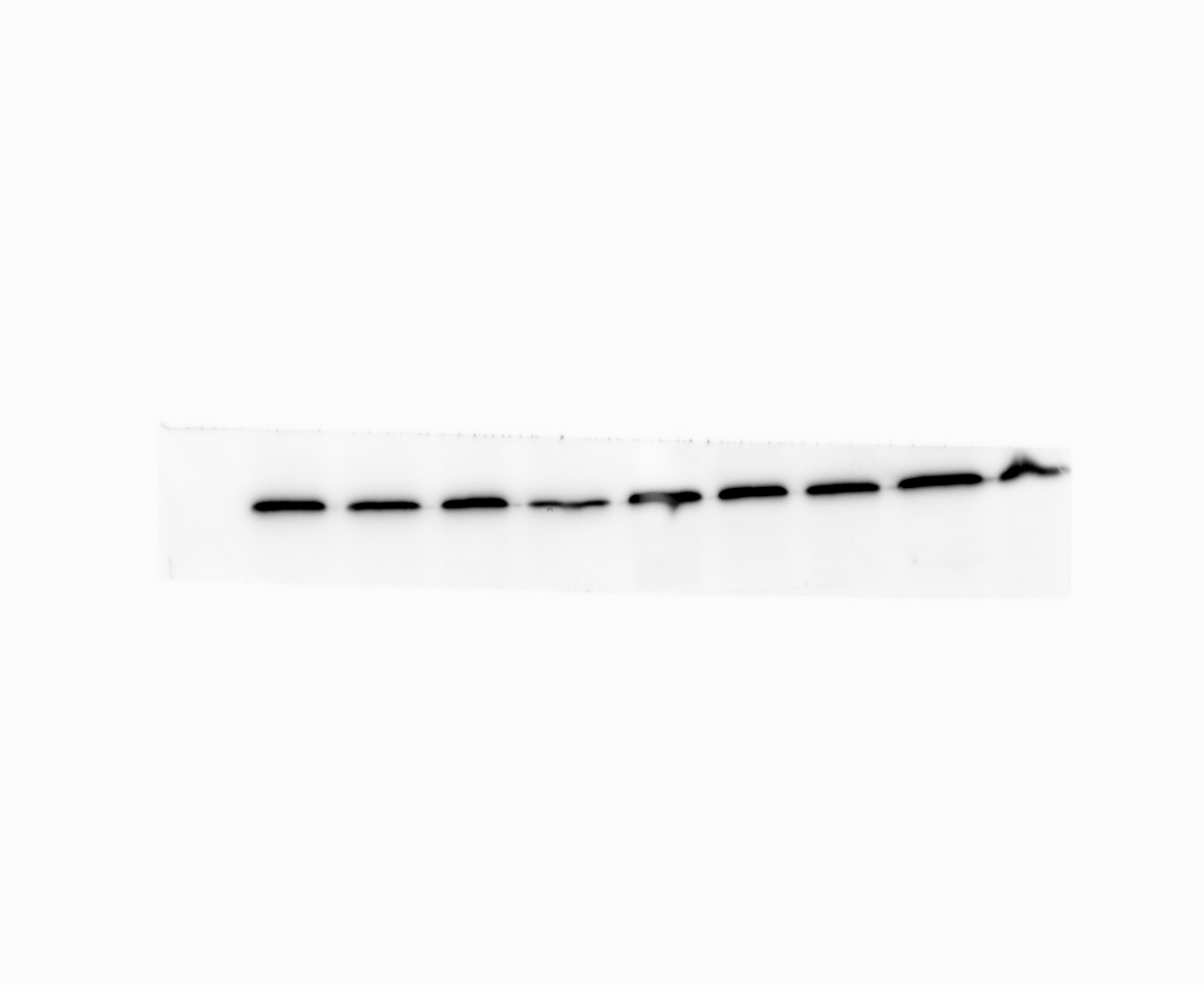

Supplement: Supplementary file 102 — Additional file 102. [file 13020_2026_1383_MOESM102_ESM.tif]

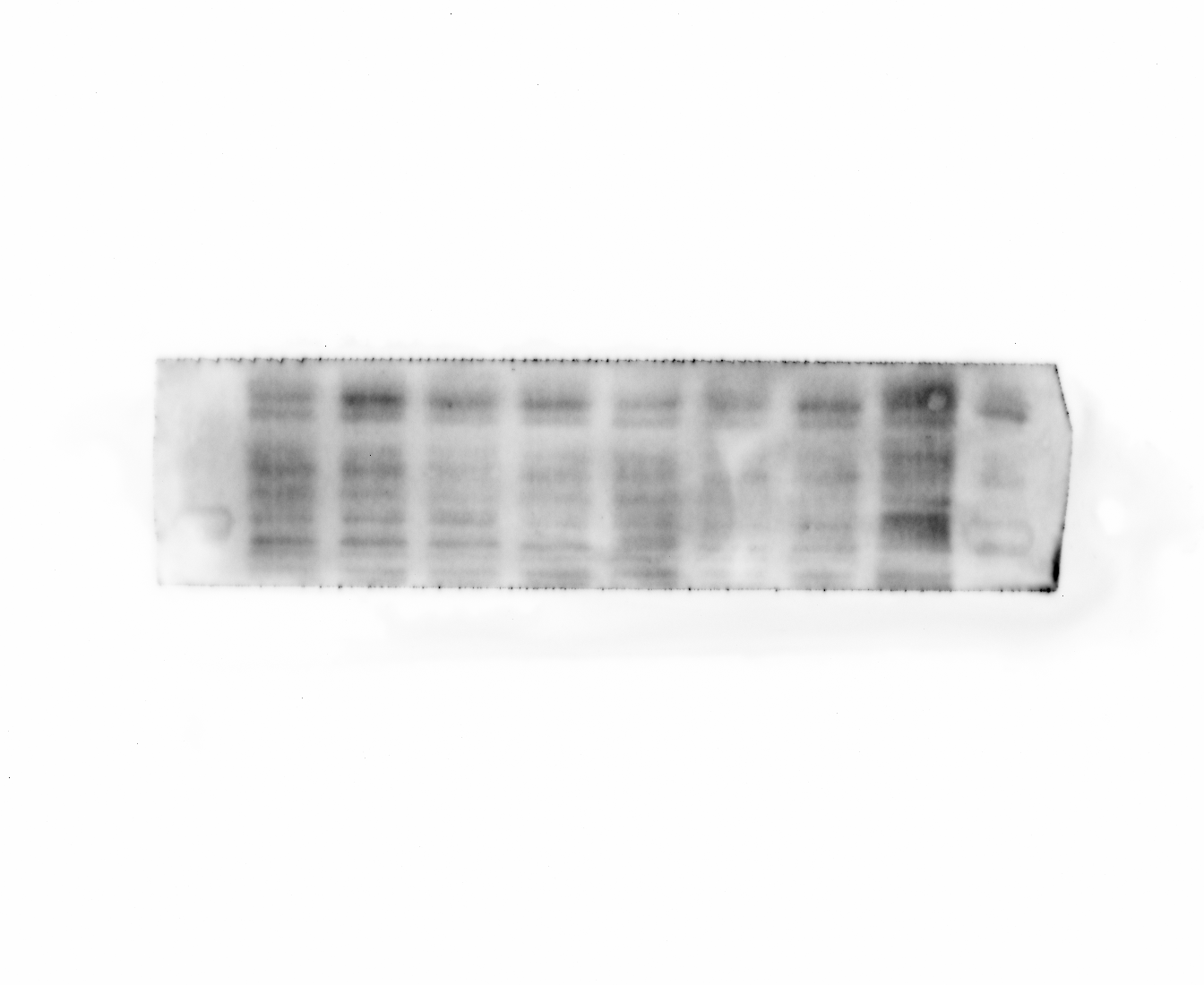

Supplement: Supplementary file 103 — Additional file 103. [file 13020_2026_1383_MOESM103_ESM.tif]

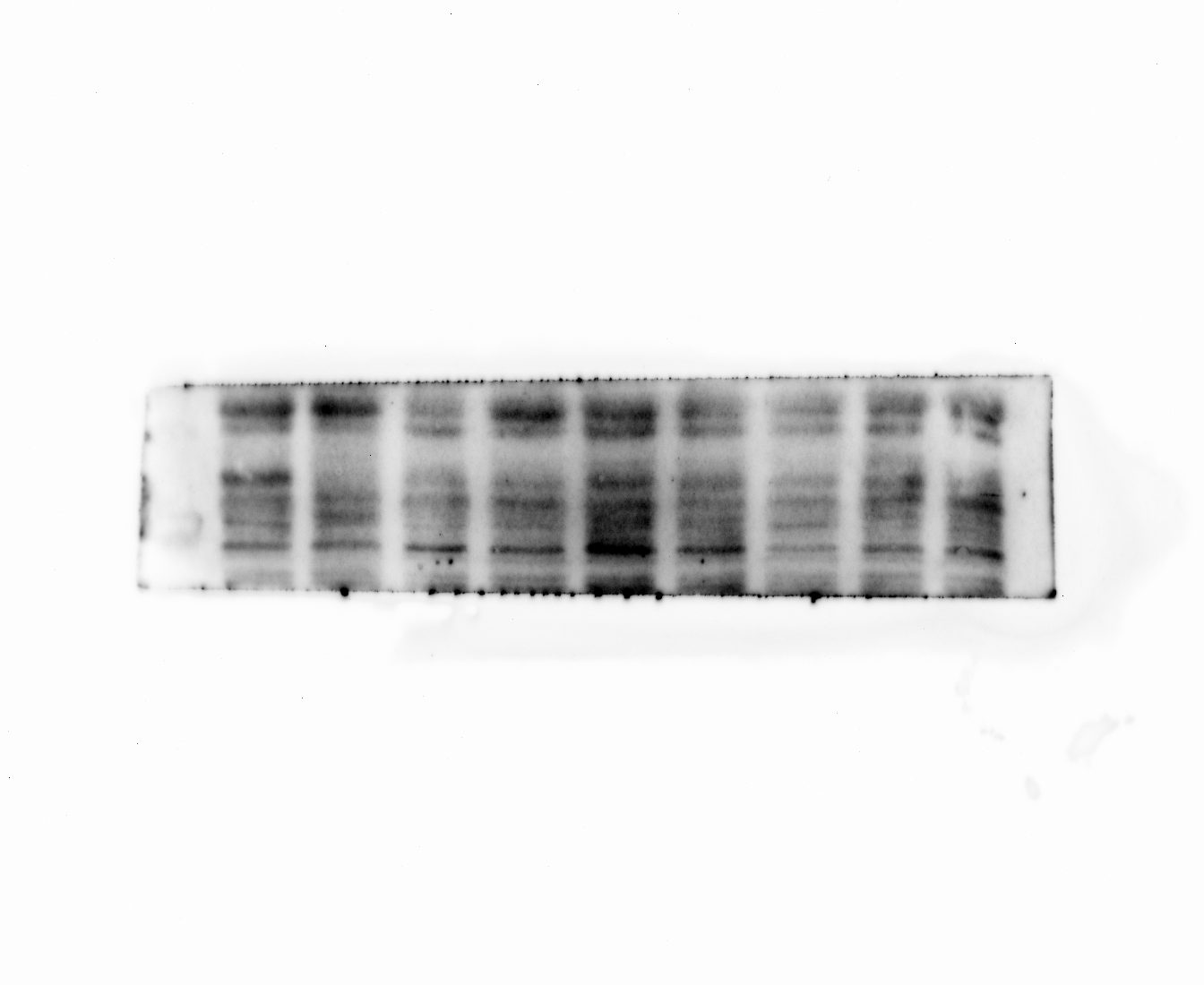

Supplement: Supplementary file 104 — Additional file 104. [file 13020_2026_1383_MOESM104_ESM.tif]

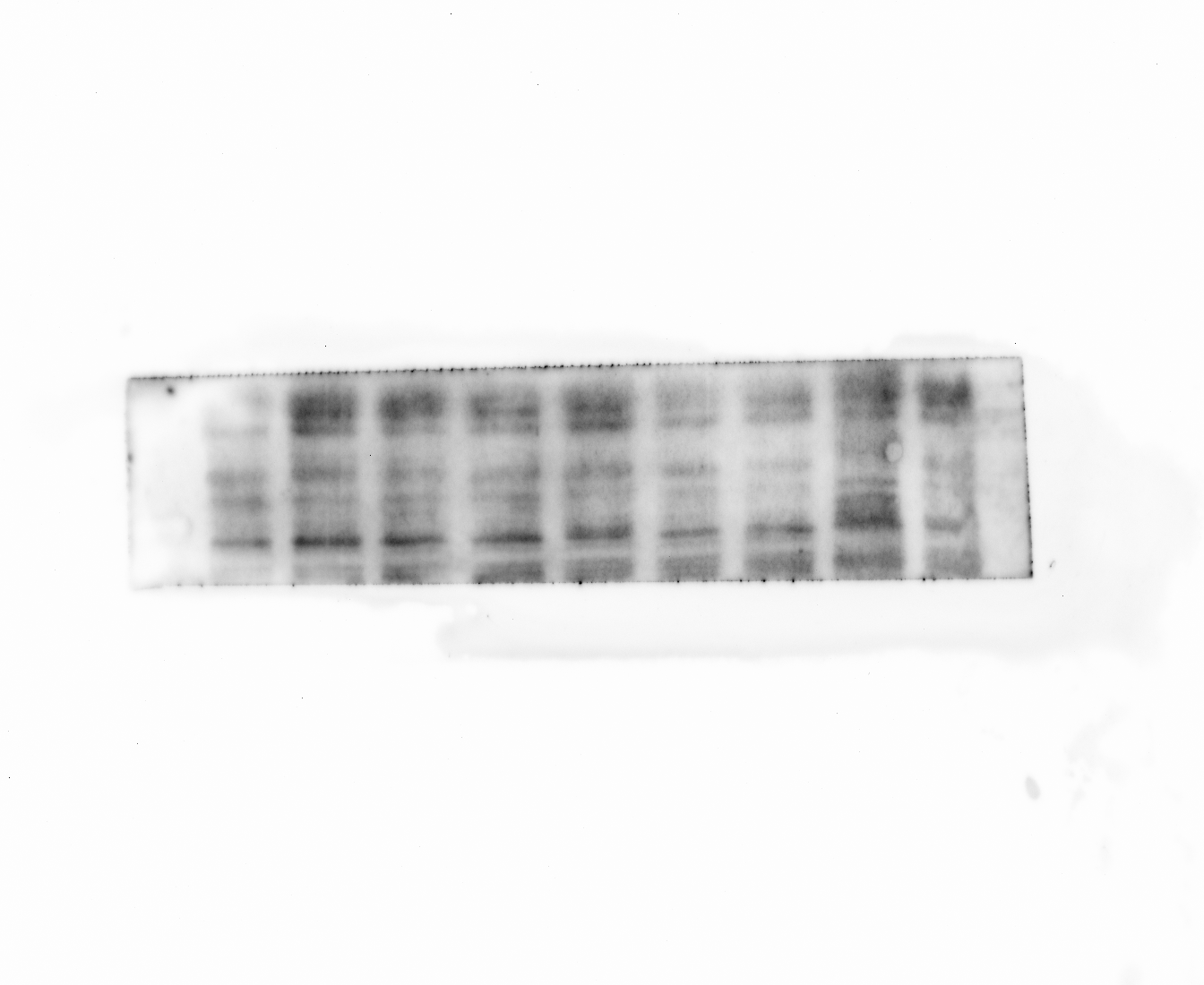

Supplement: Supplementary file 105 — Additional file 105. [file 13020_2026_1383_MOESM105_ESM.tif]

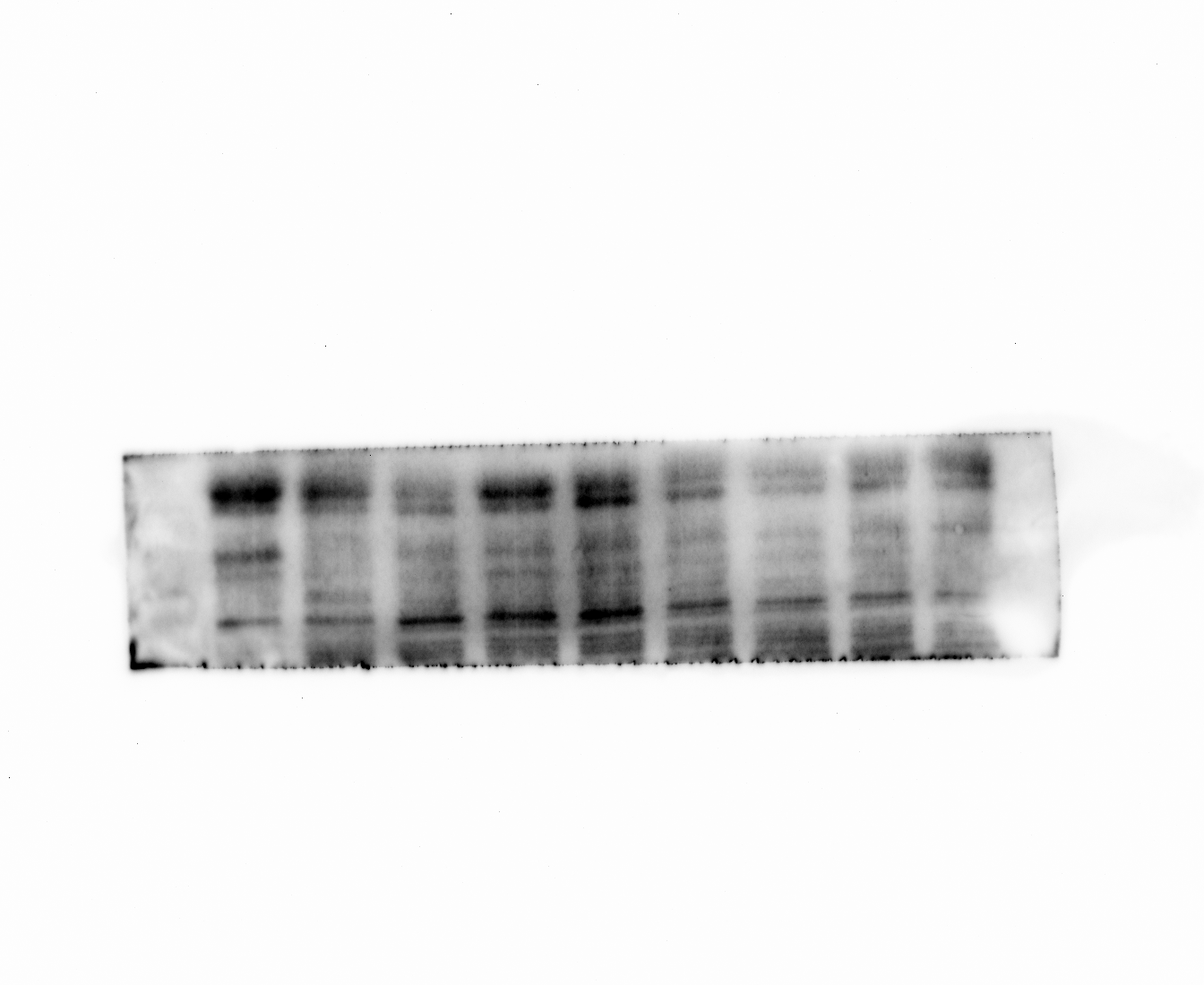

Supplement: Supplementary file 106 — Additional file 106. [file 13020_2026_1383_MOESM106_ESM.tif]

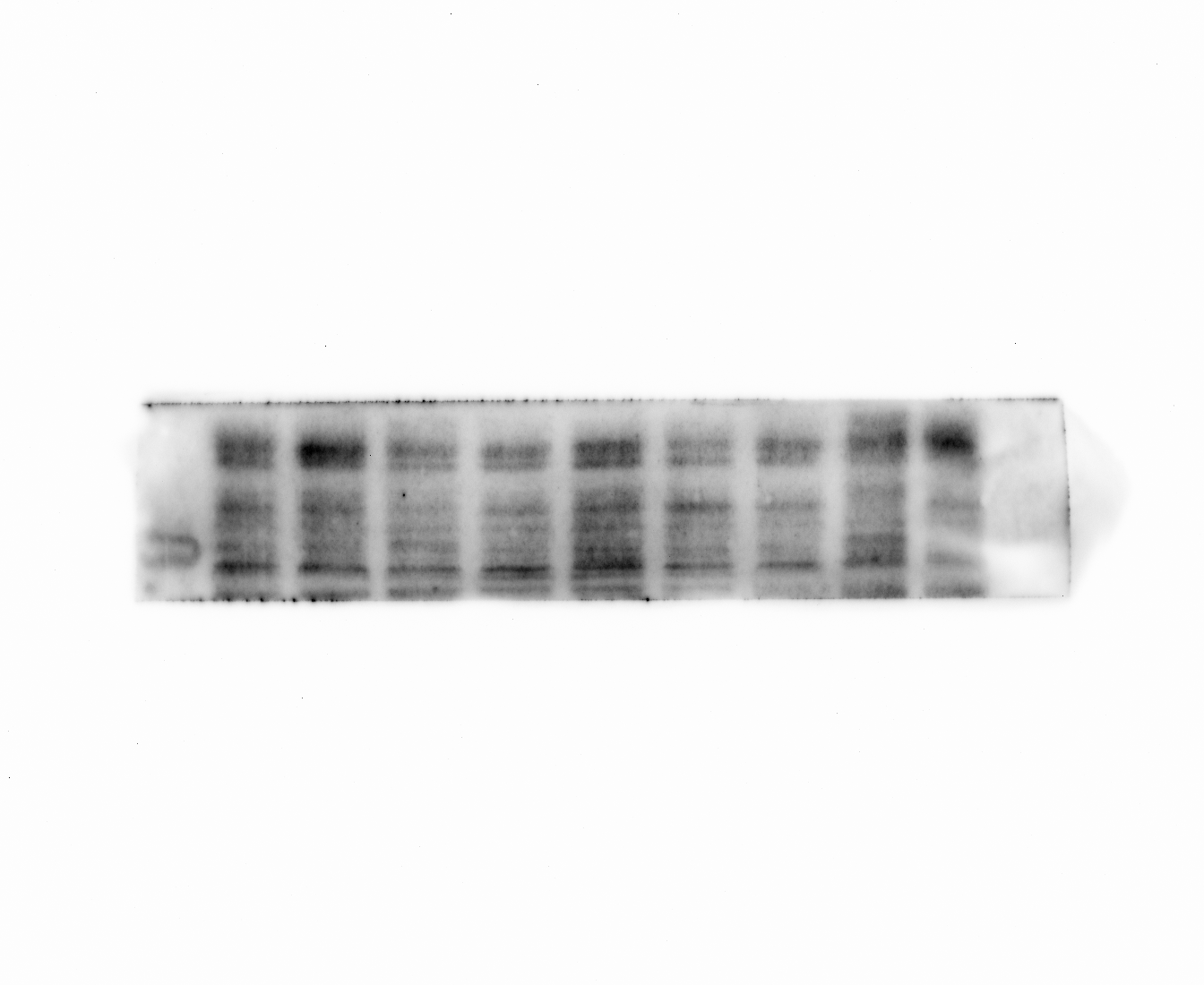

Supplement: Supplementary file 107 — Additional file 107. [file 13020_2026_1383_MOESM107_ESM.tif]

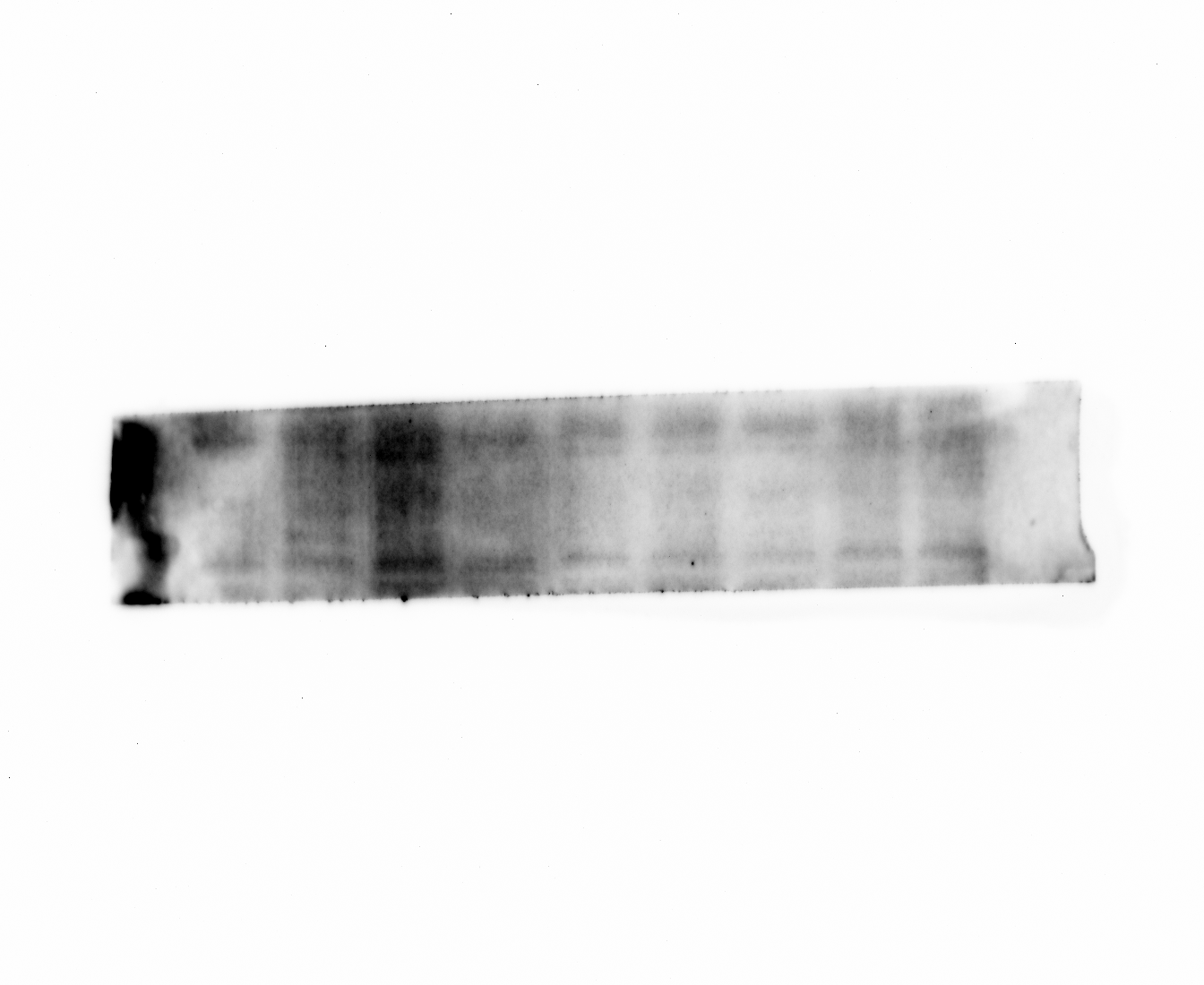

Supplement: Supplementary file 108 — Additional file 108. [file 13020_2026_1383_MOESM108_ESM.tif]

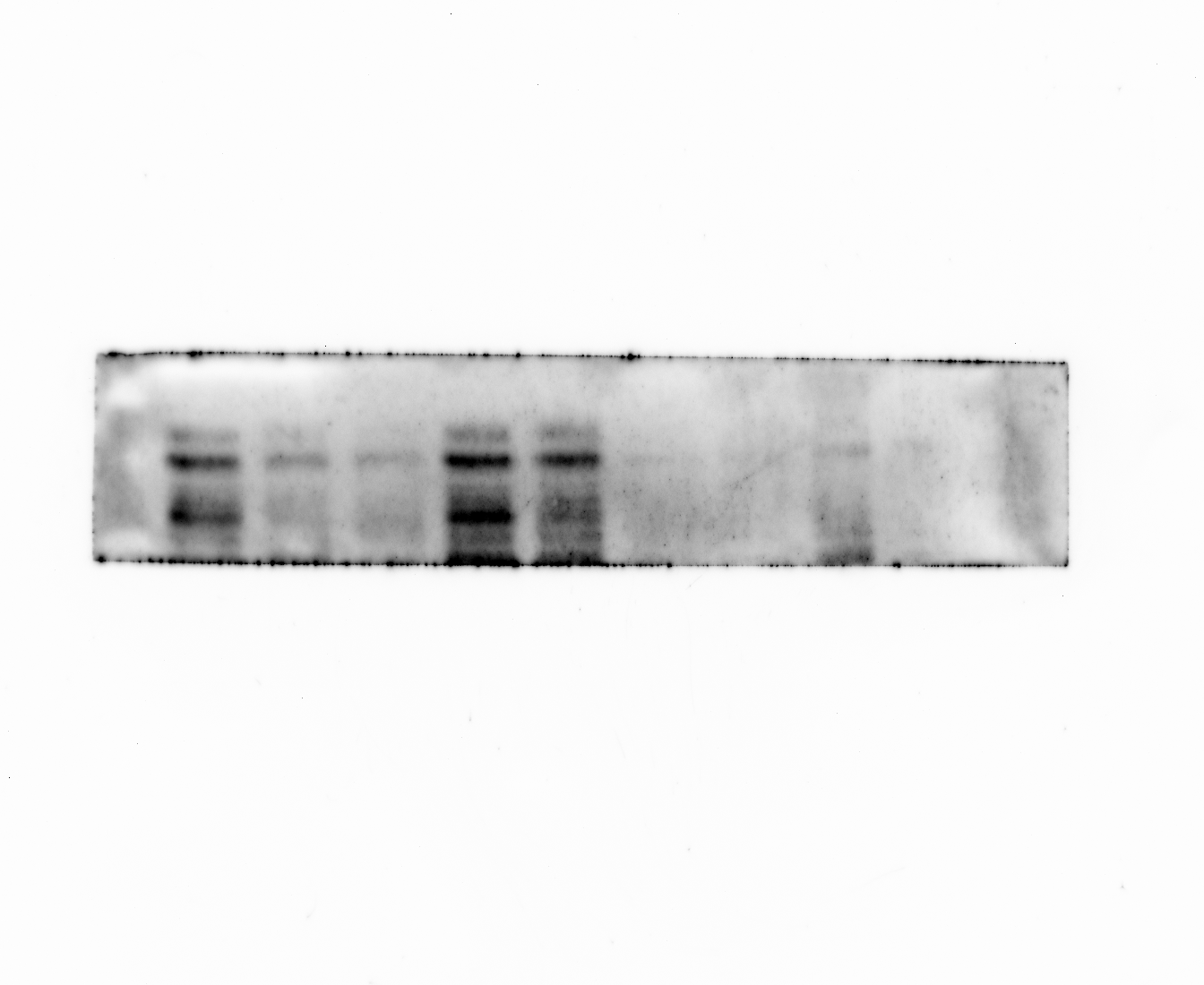

Supplement: Supplementary file 109 — Additional file 109. [file 13020_2026_1383_MOESM109_ESM.tif]

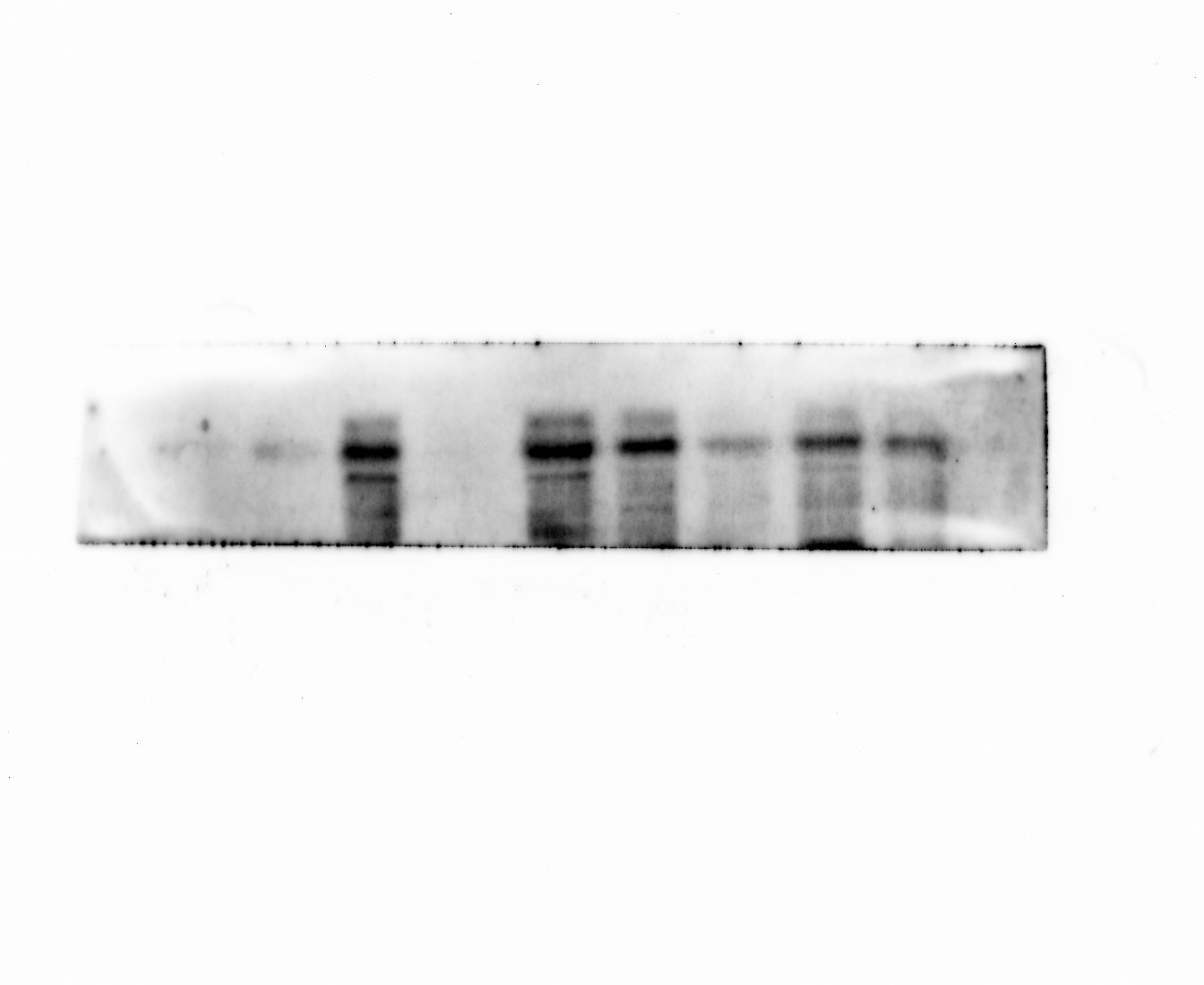

Supplement: Supplementary file 110 — Additional file 110. [file 13020_2026_1383_MOESM110_ESM.tif]

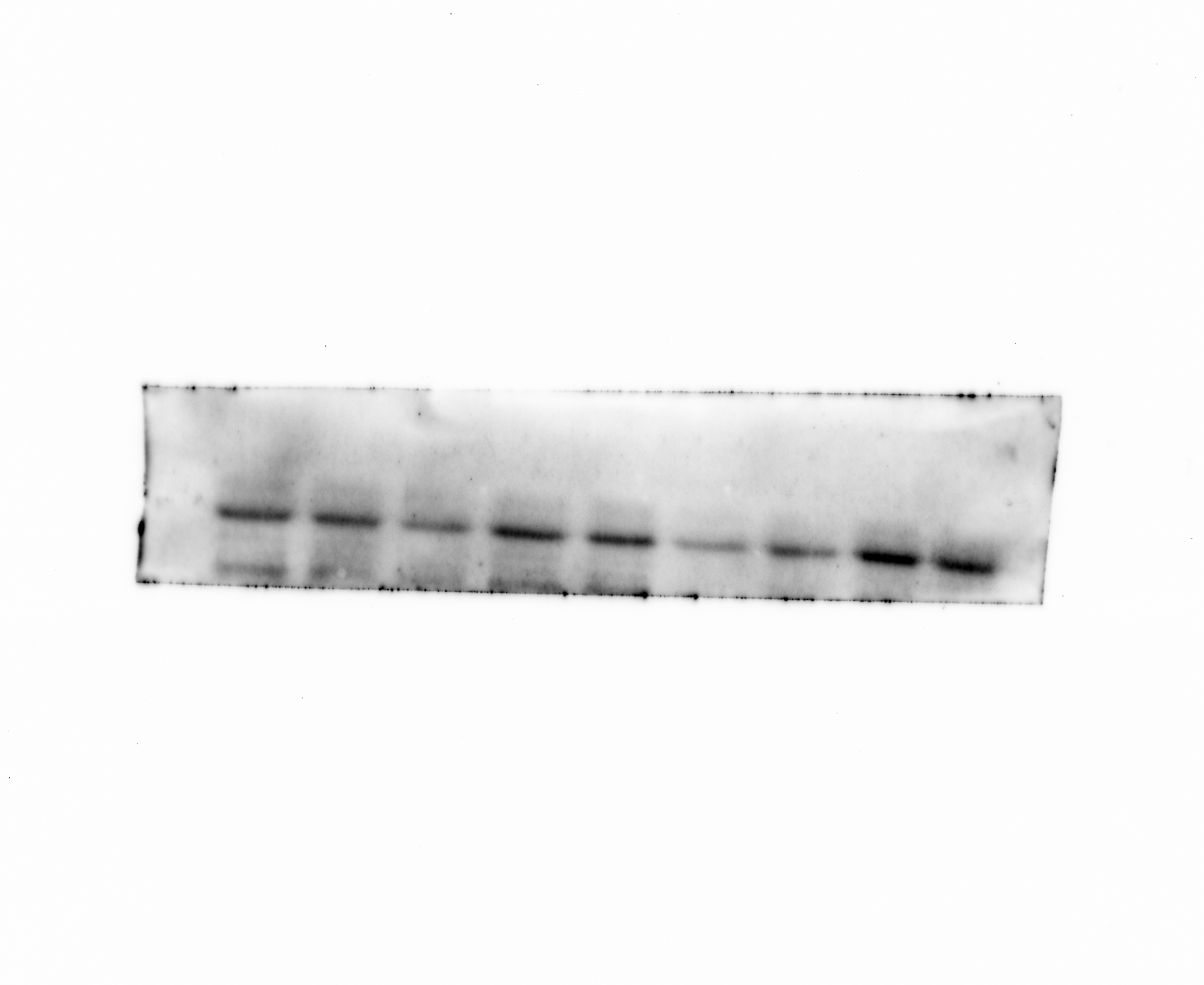

Supplement: Supplementary file 111 — Additional file 111. [file 13020_2026_1383_MOESM111_ESM.tif]

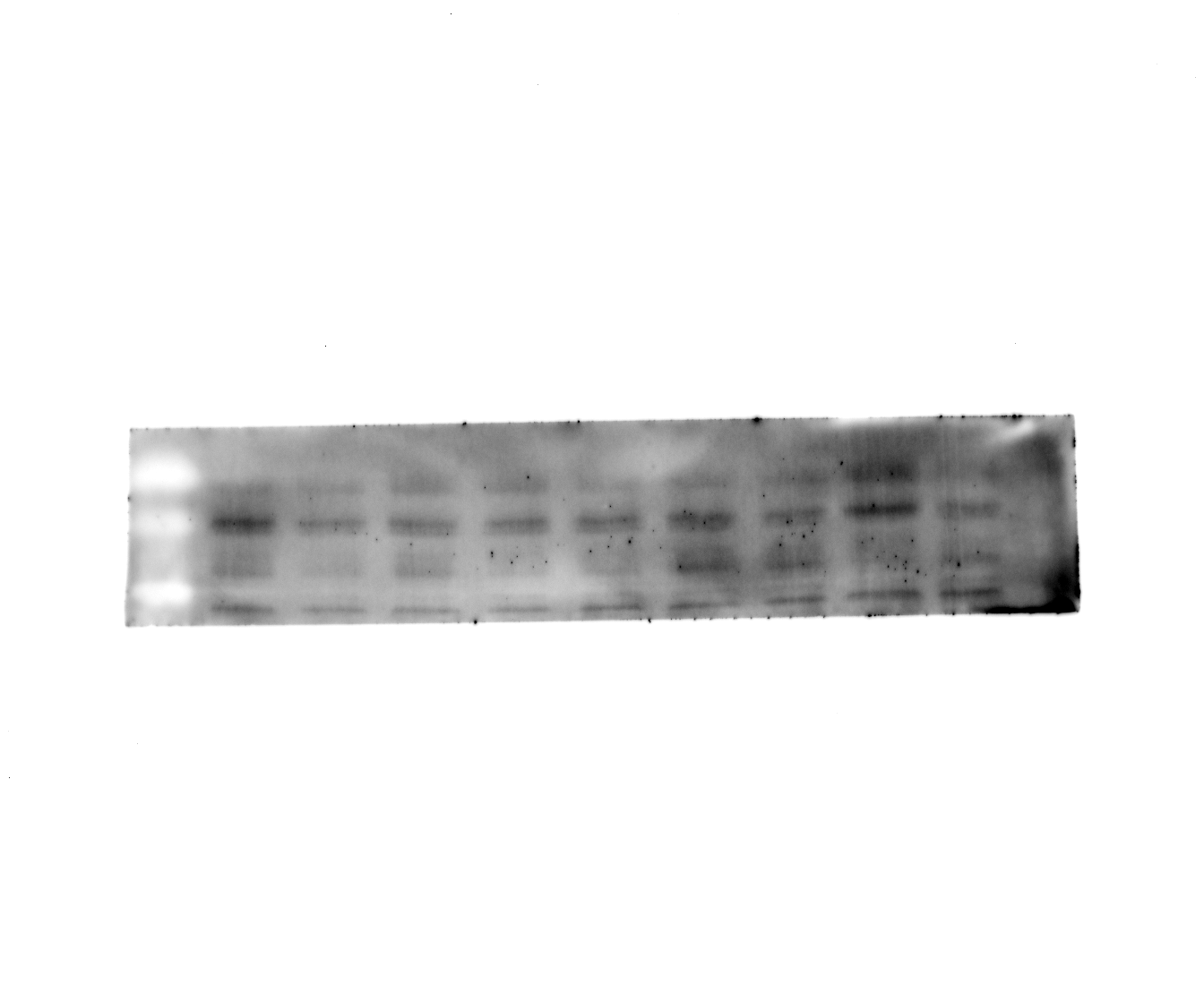

Supplement: Supplementary file 112 — Additional file 112. [file 13020_2026_1383_MOESM112_ESM.tif]

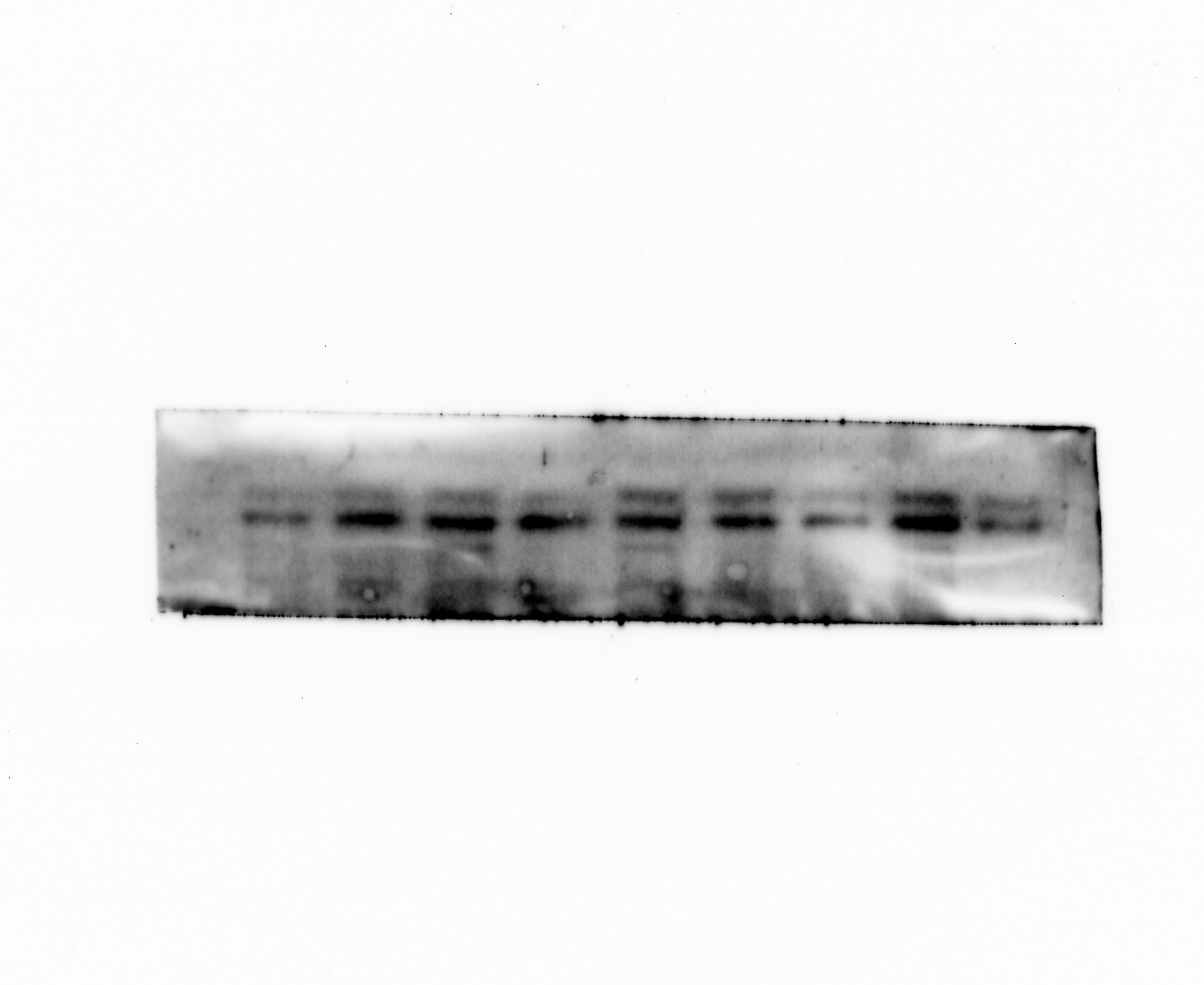

Supplement: Supplementary file 113 — Additional file 113. [file 13020_2026_1383_MOESM113_ESM.tif]

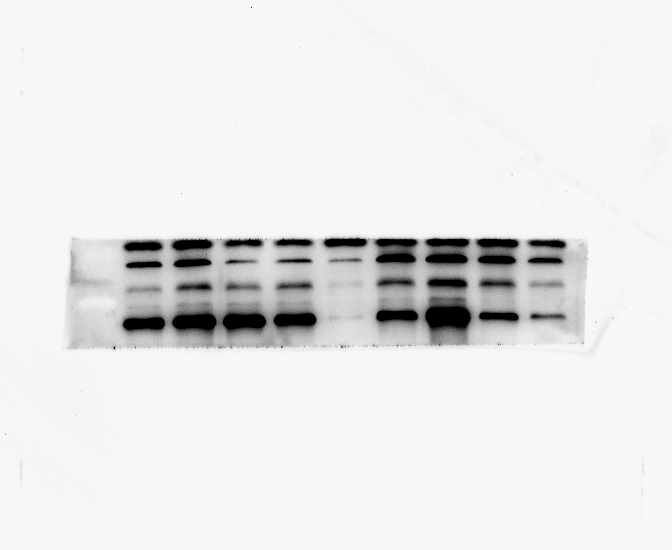

Supplement: Supplementary file 114 — Additional file 114. [file 13020_2026_1383_MOESM114_ESM.tif]

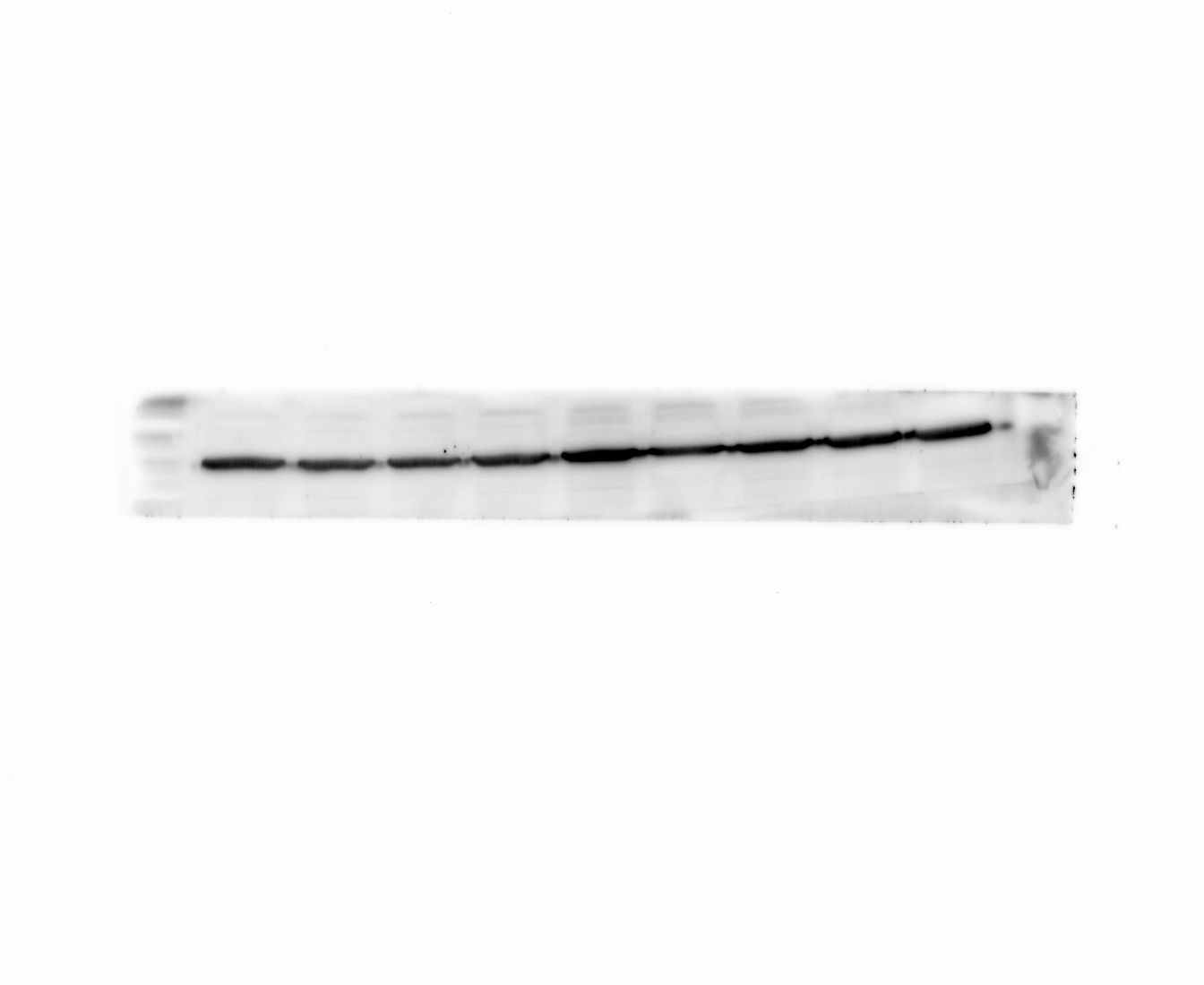

Supplement: Supplementary file 115 — Additional file 115. [file 13020_2026_1383_MOESM115_ESM.tif]

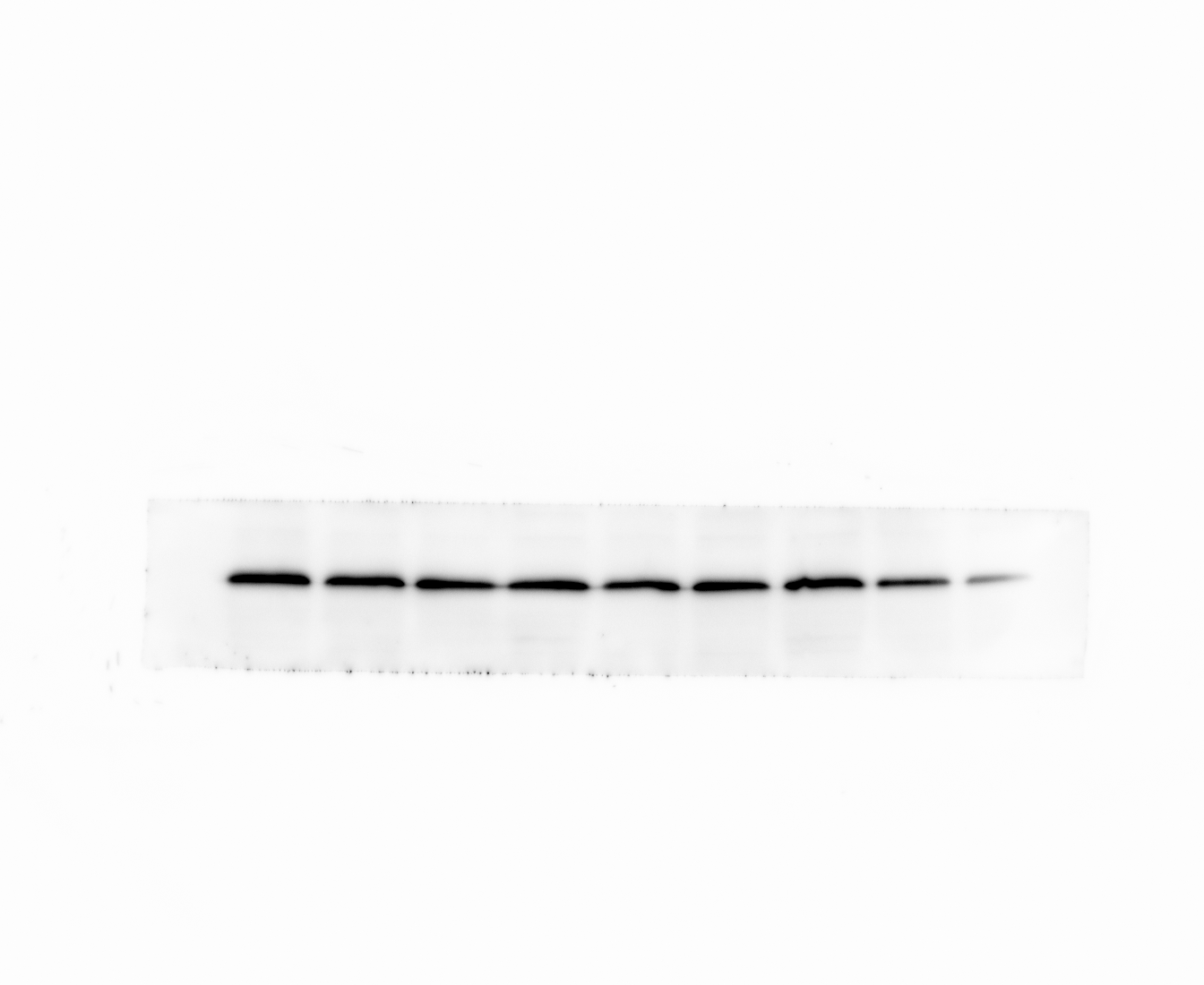

Supplement: Supplementary file 116 — Additional file 116. [file 13020_2026_1383_MOESM116_ESM.tif]

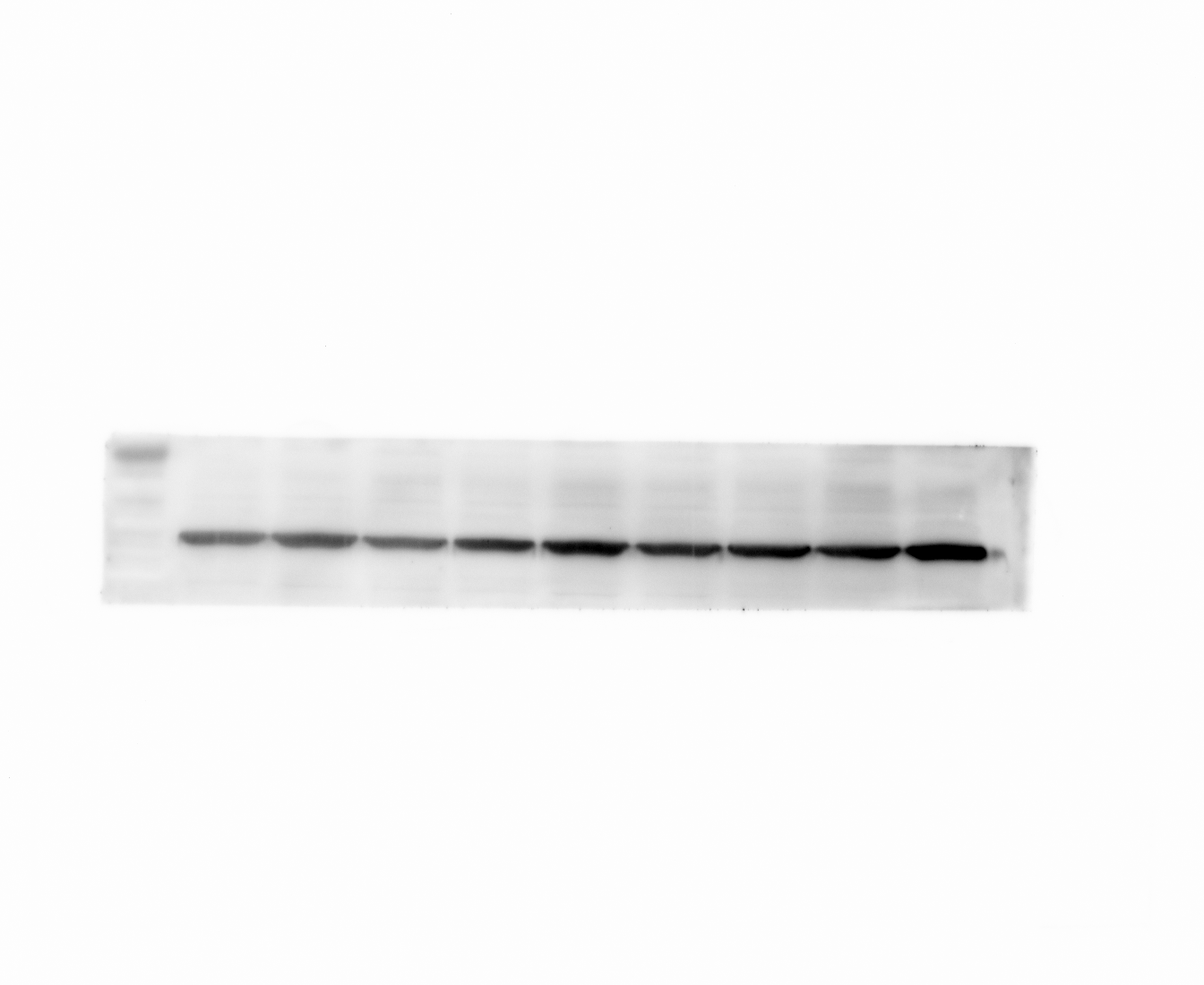

Supplement: Supplementary file 117 — Additional file 117. [file 13020_2026_1383_MOESM117_ESM.tif]

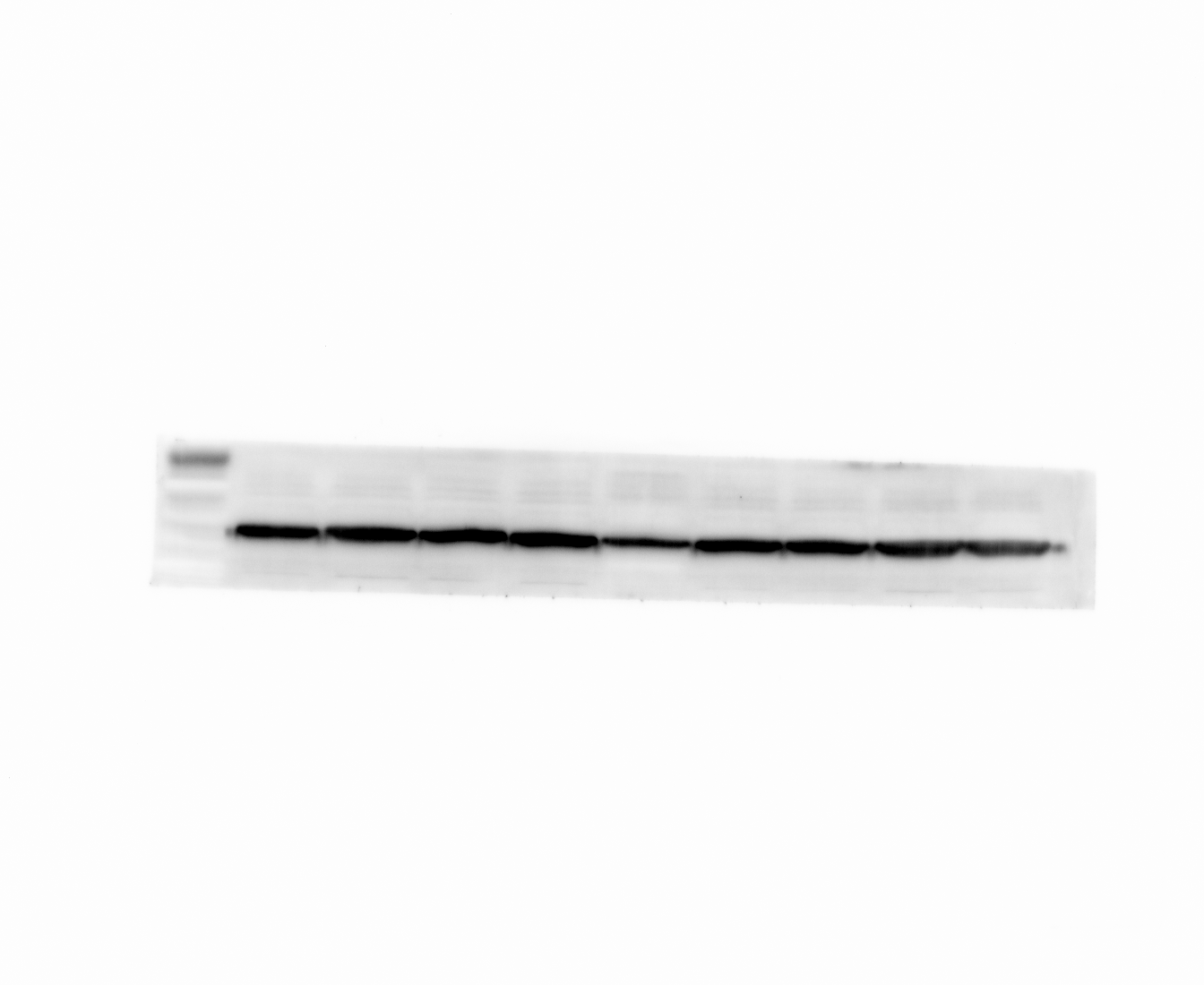

Supplement: Supplementary file 118 — Additional file 118. [file 13020_2026_1383_MOESM118_ESM.tif]

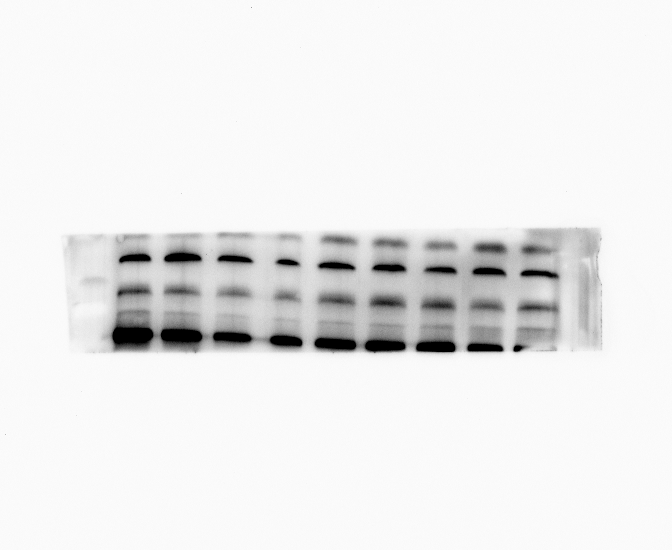

Supplement: Supplementary file 119 — Additional file 119. [file 13020_2026_1383_MOESM119_ESM.tif]

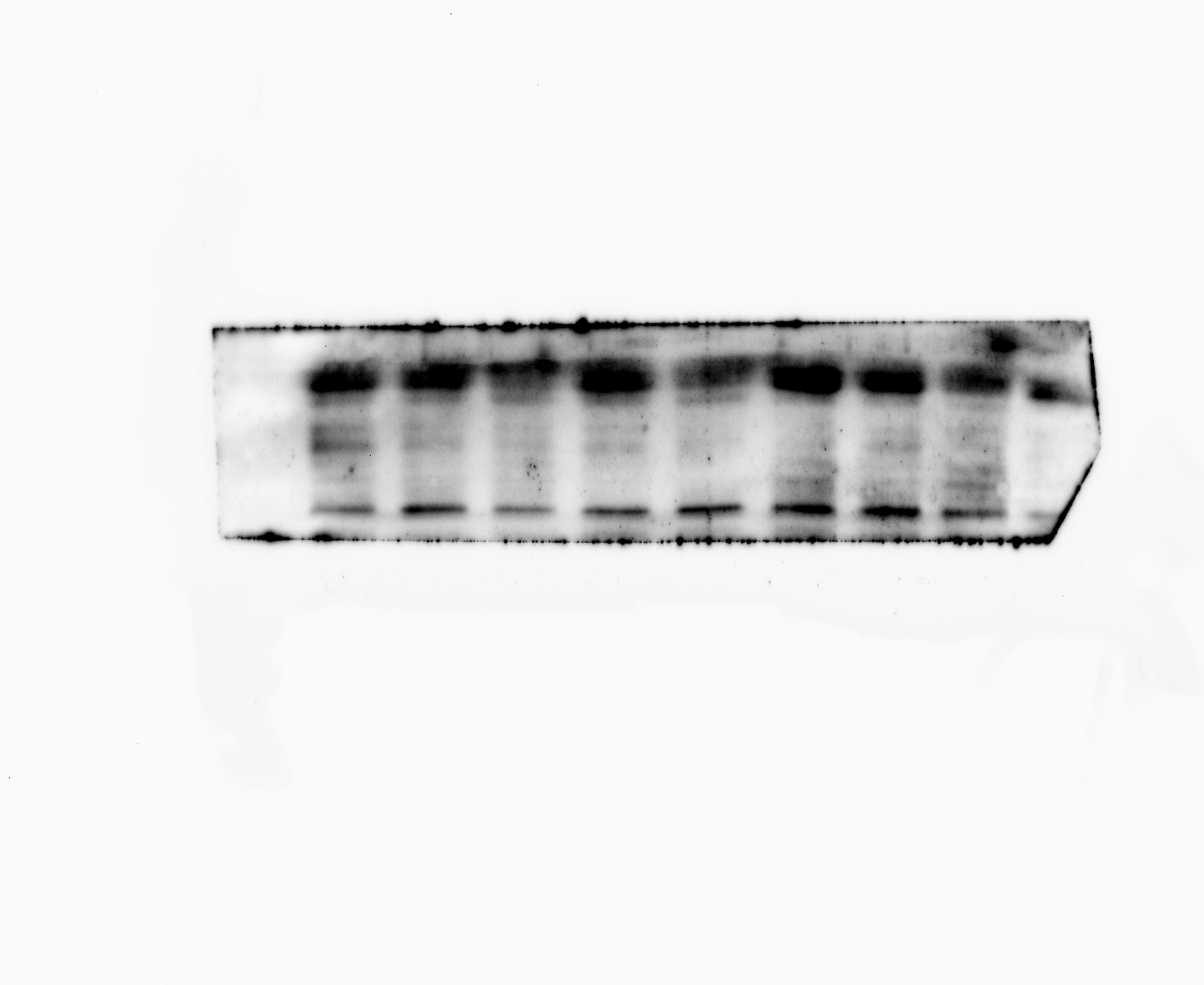

Supplement: Supplementary file 120 — Additional file 120. [file 13020_2026_1383_MOESM120_ESM.tif]

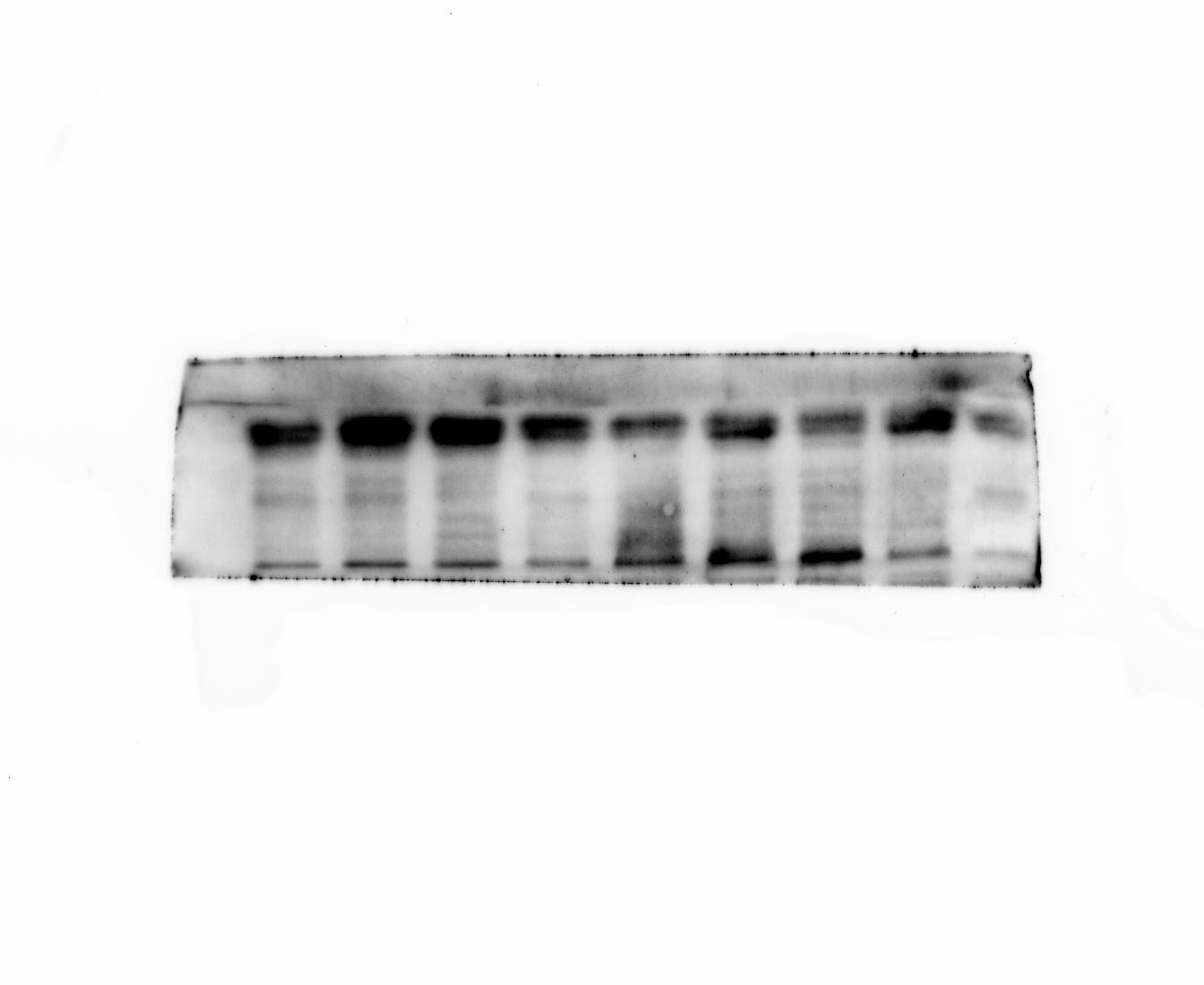

Supplement: Supplementary file 121 — Additional file 121. [file 13020_2026_1383_MOESM121_ESM.tif]

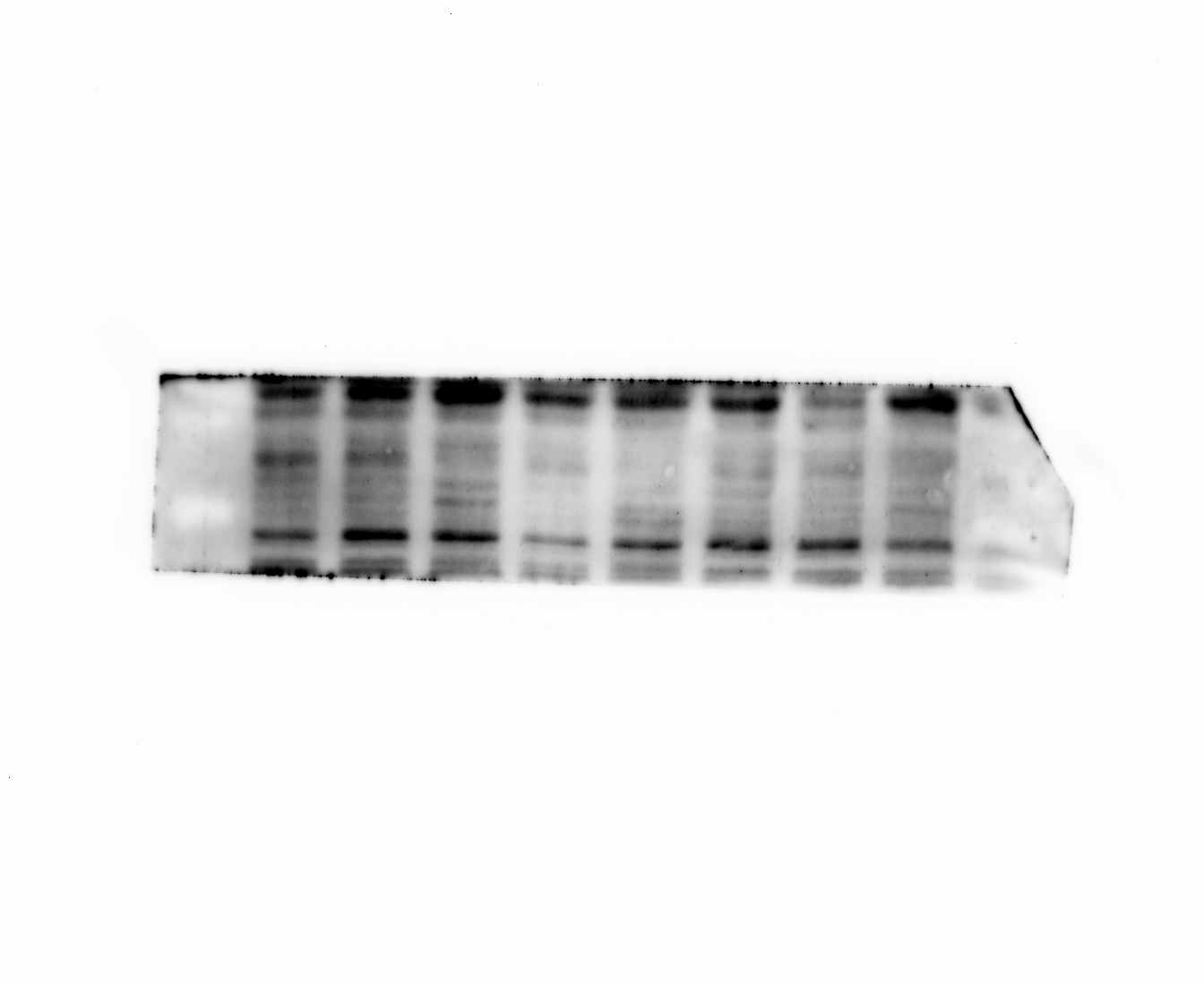

Supplement: Supplementary file 122 — Additional file 122. [file 13020_2026_1383_MOESM122_ESM.tif]

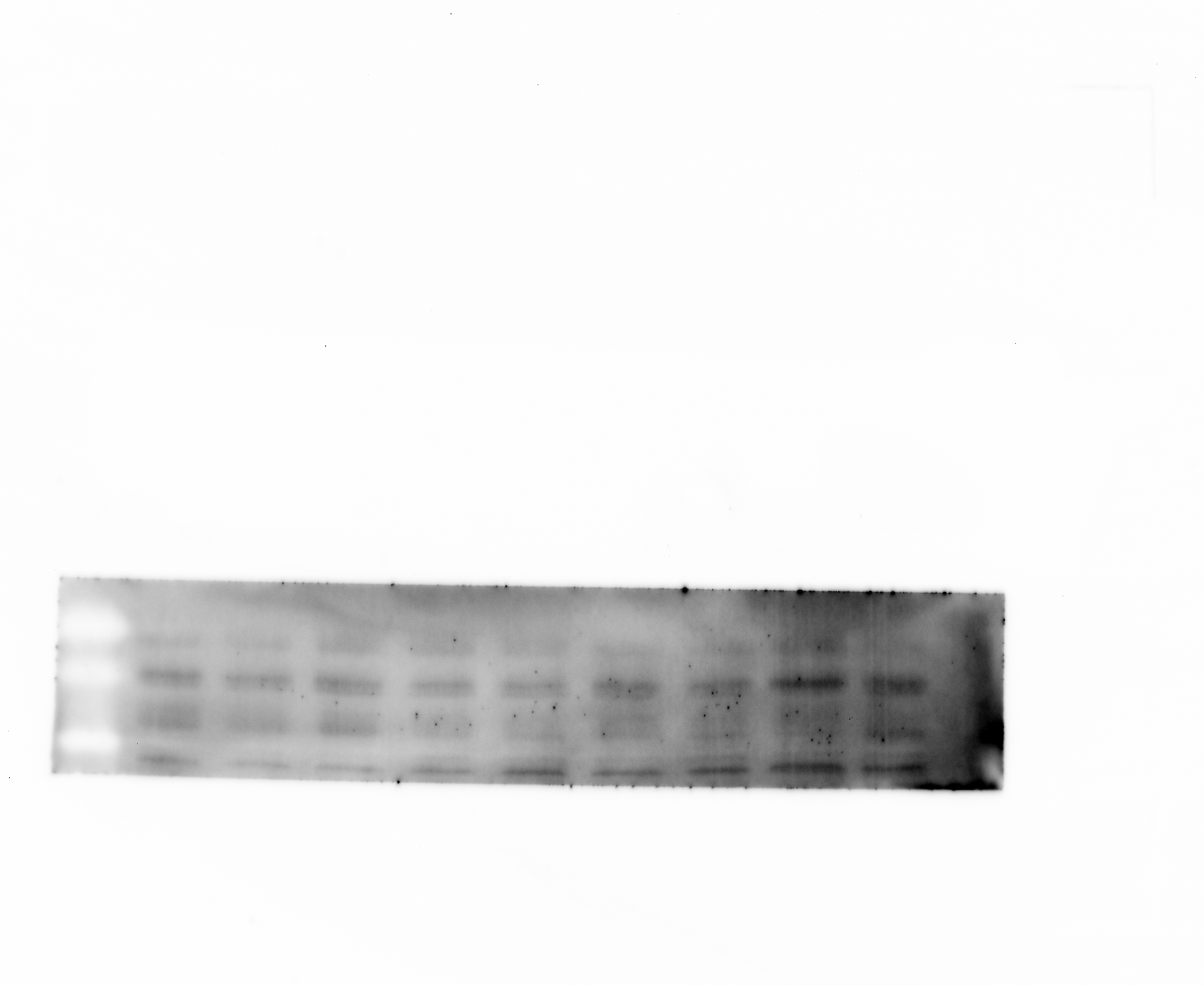

Supplement: Supplementary file 123 — Additional file 123. [file 13020_2026_1383_MOESM123_ESM.tif]

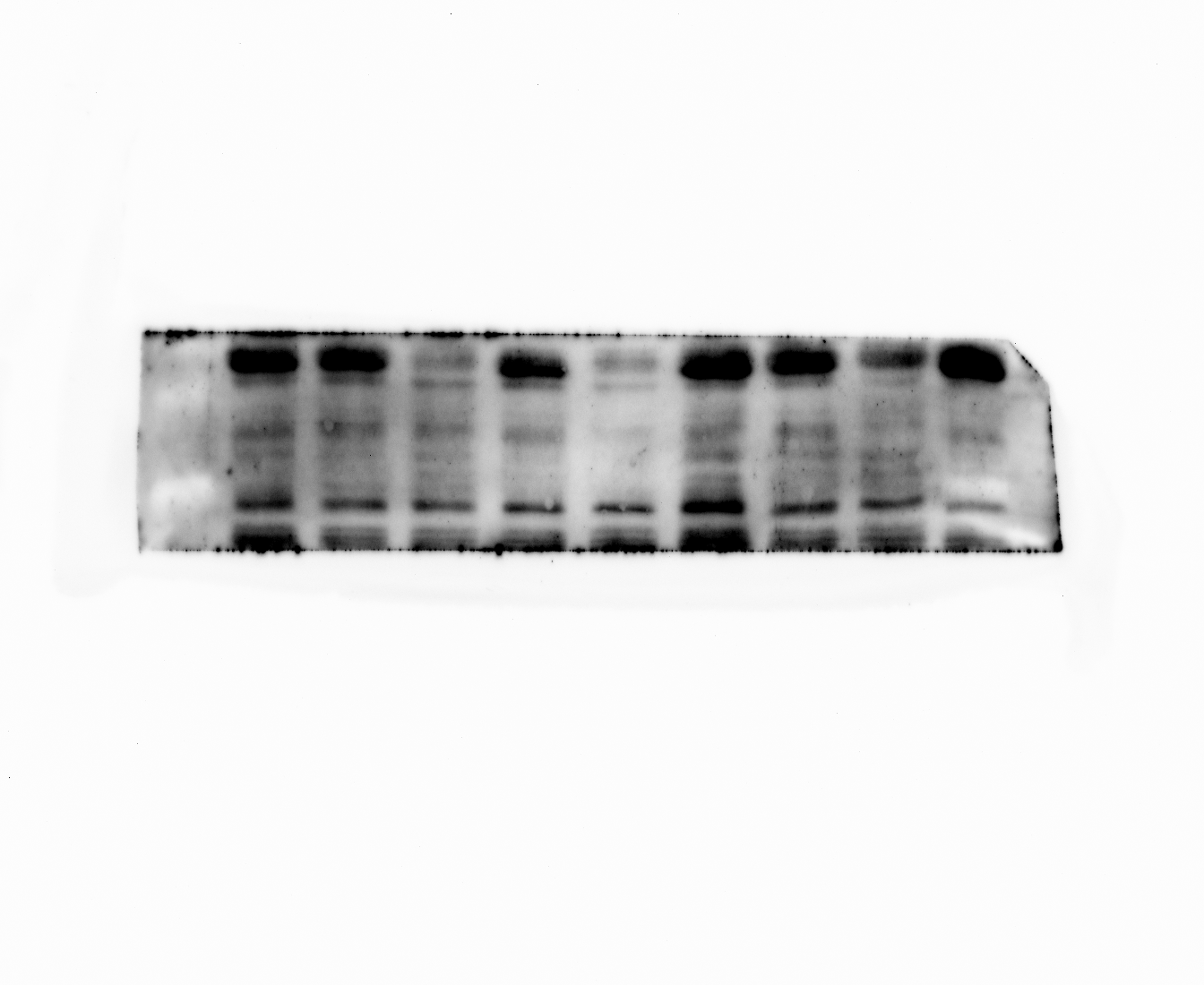

Supplement: Supplementary file 124 — Additional file 124. [file 13020_2026_1383_MOESM124_ESM.tif]

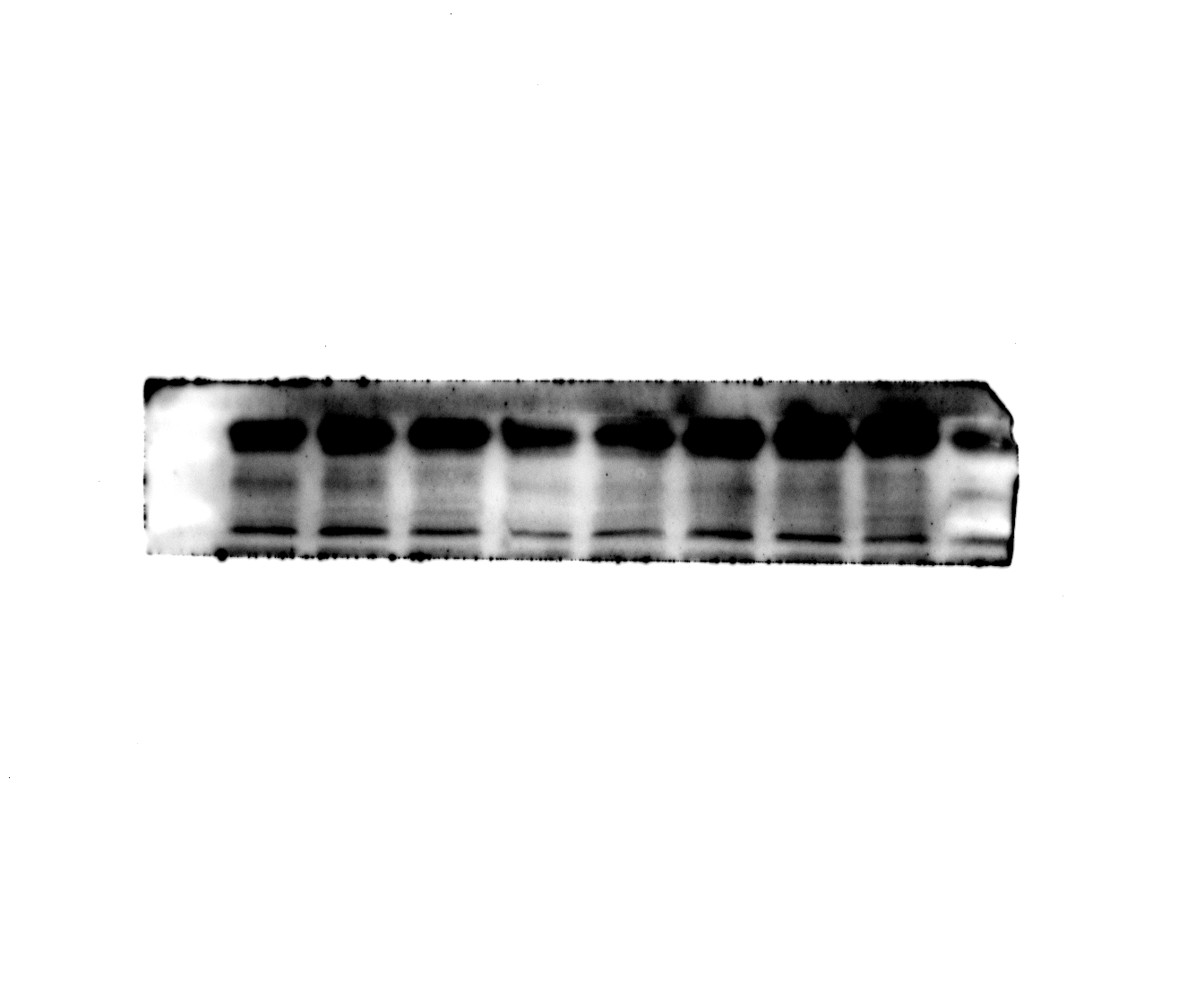

Supplement: Supplementary file 125 — Additional file 125. [file 13020_2026_1383_MOESM125_ESM.tif]

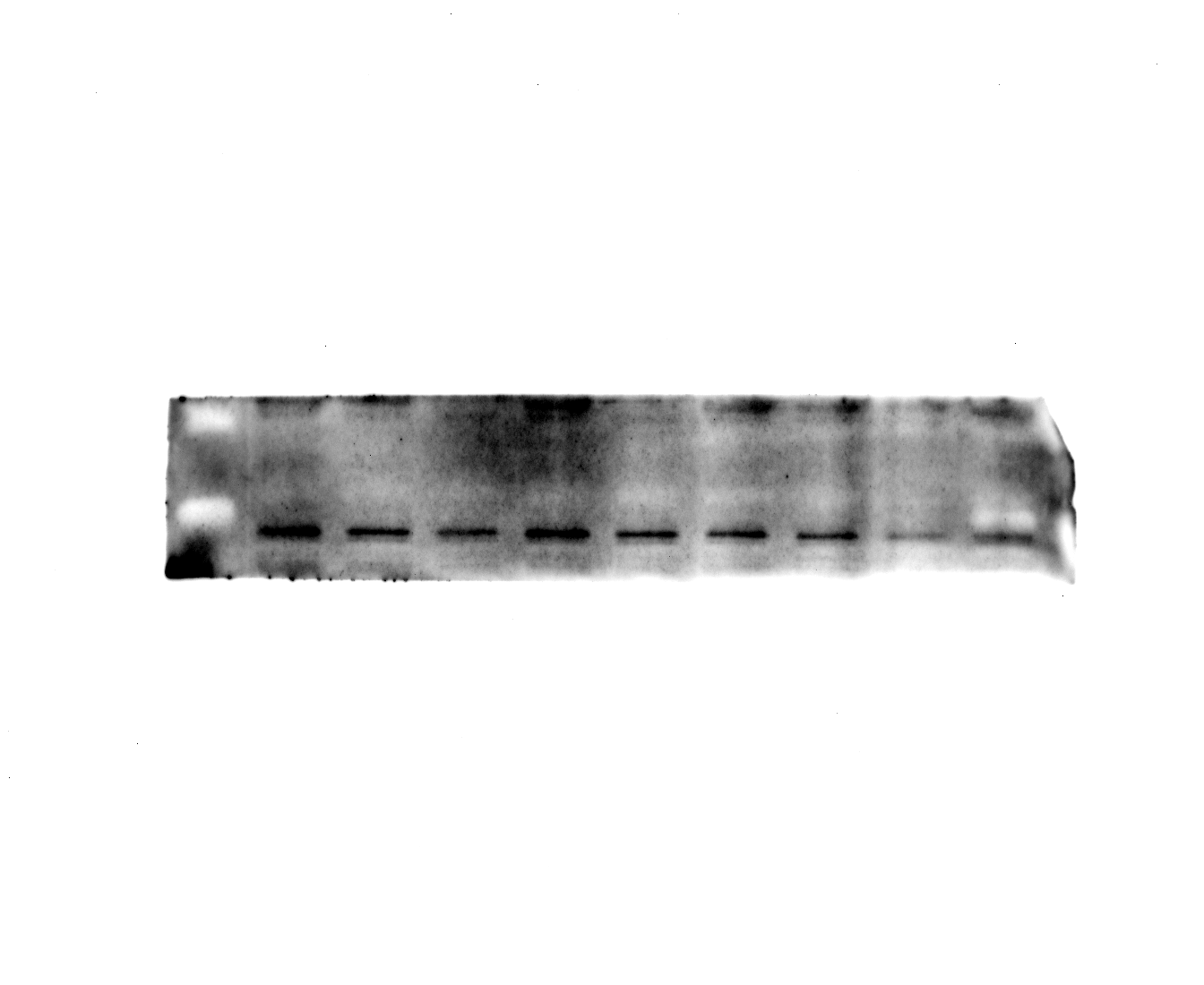

Supplement: Supplementary file 126 — Additional file 126. [file 13020_2026_1383_MOESM126_ESM.tif]

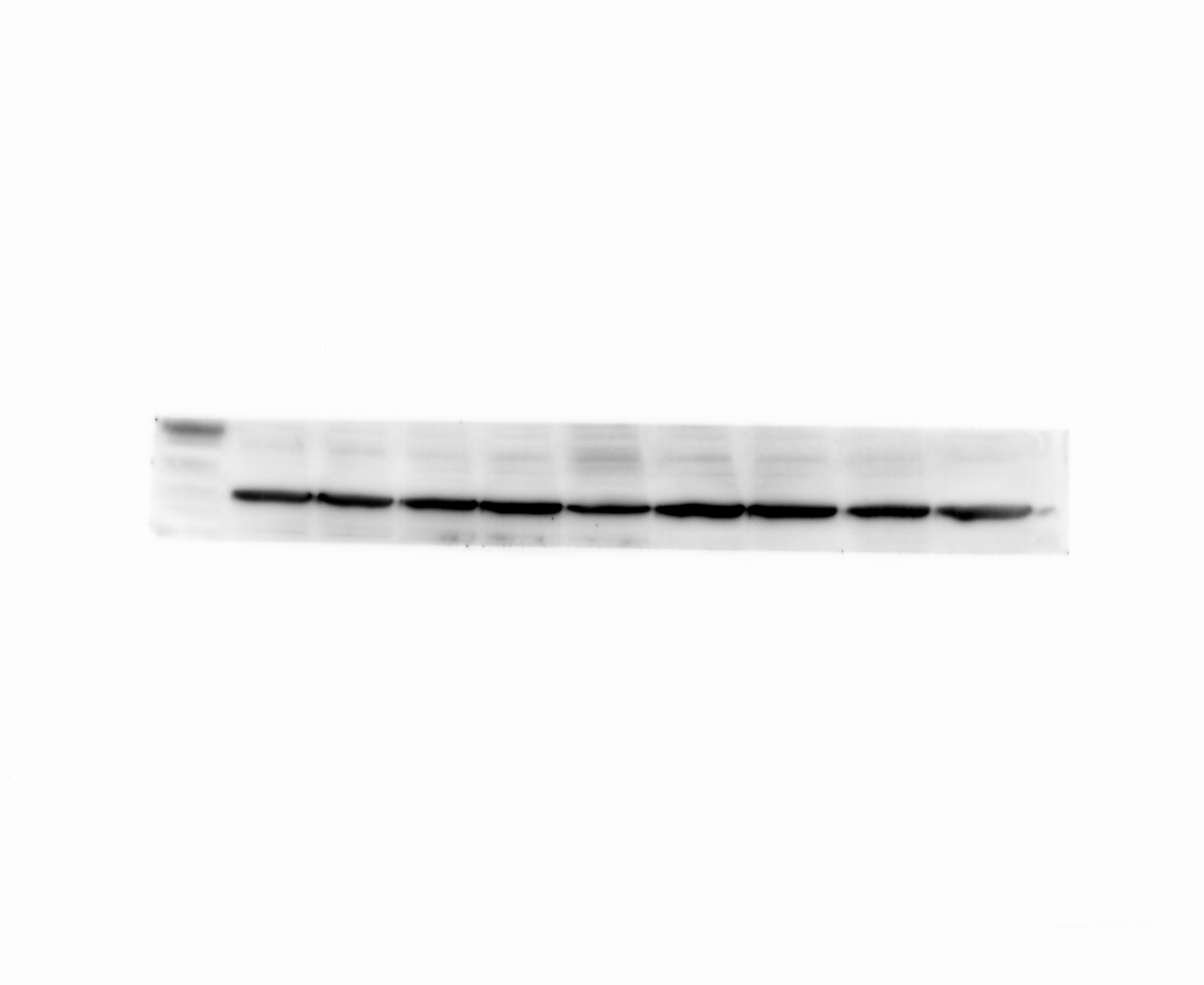

Supplement: Supplementary file 127 — Additional file 127. [file 13020_2026_1383_MOESM127_ESM.tif]

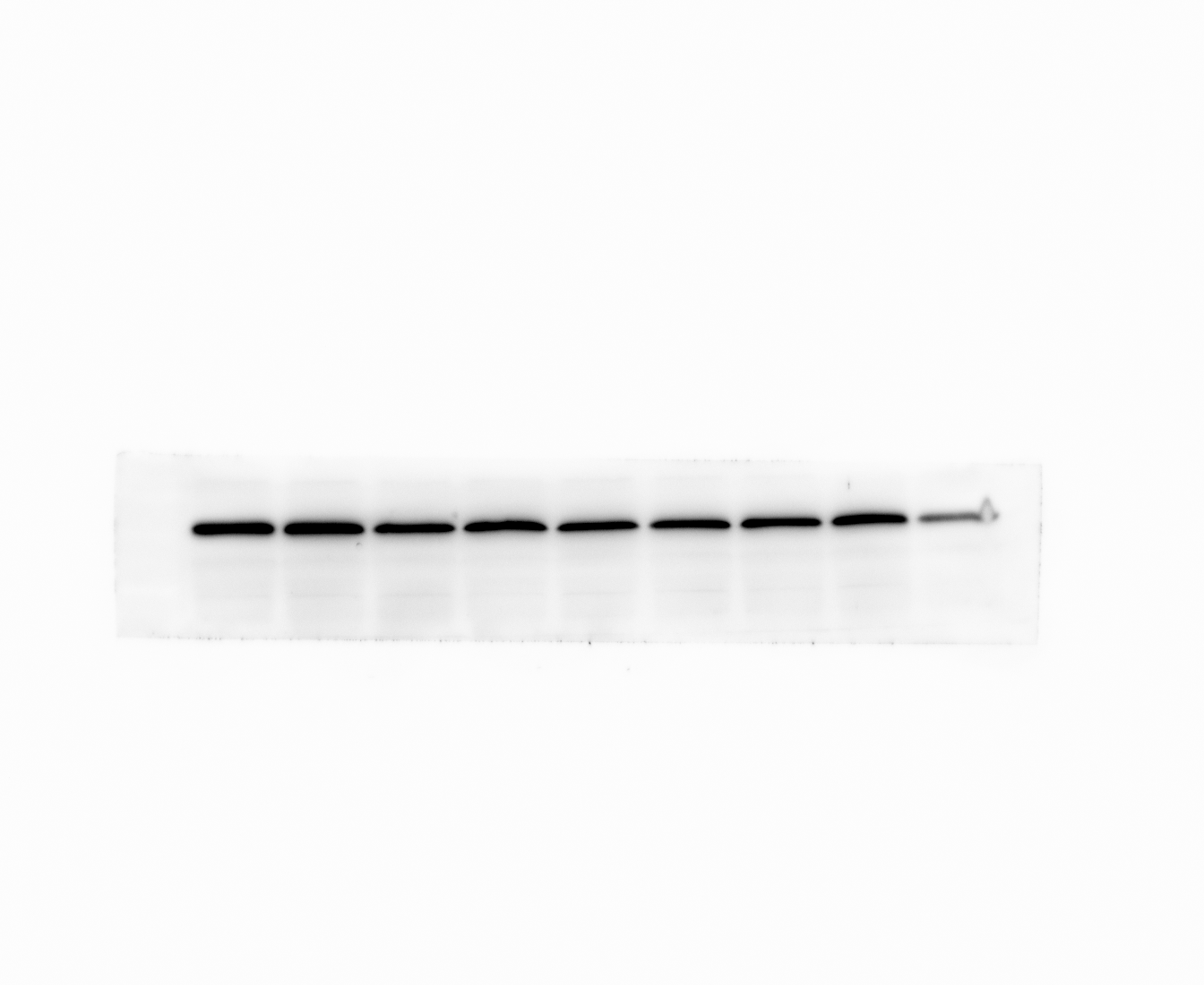

Supplement: Supplementary file 128 — Additional file 128. [file 13020_2026_1383_MOESM128_ESM.tif]

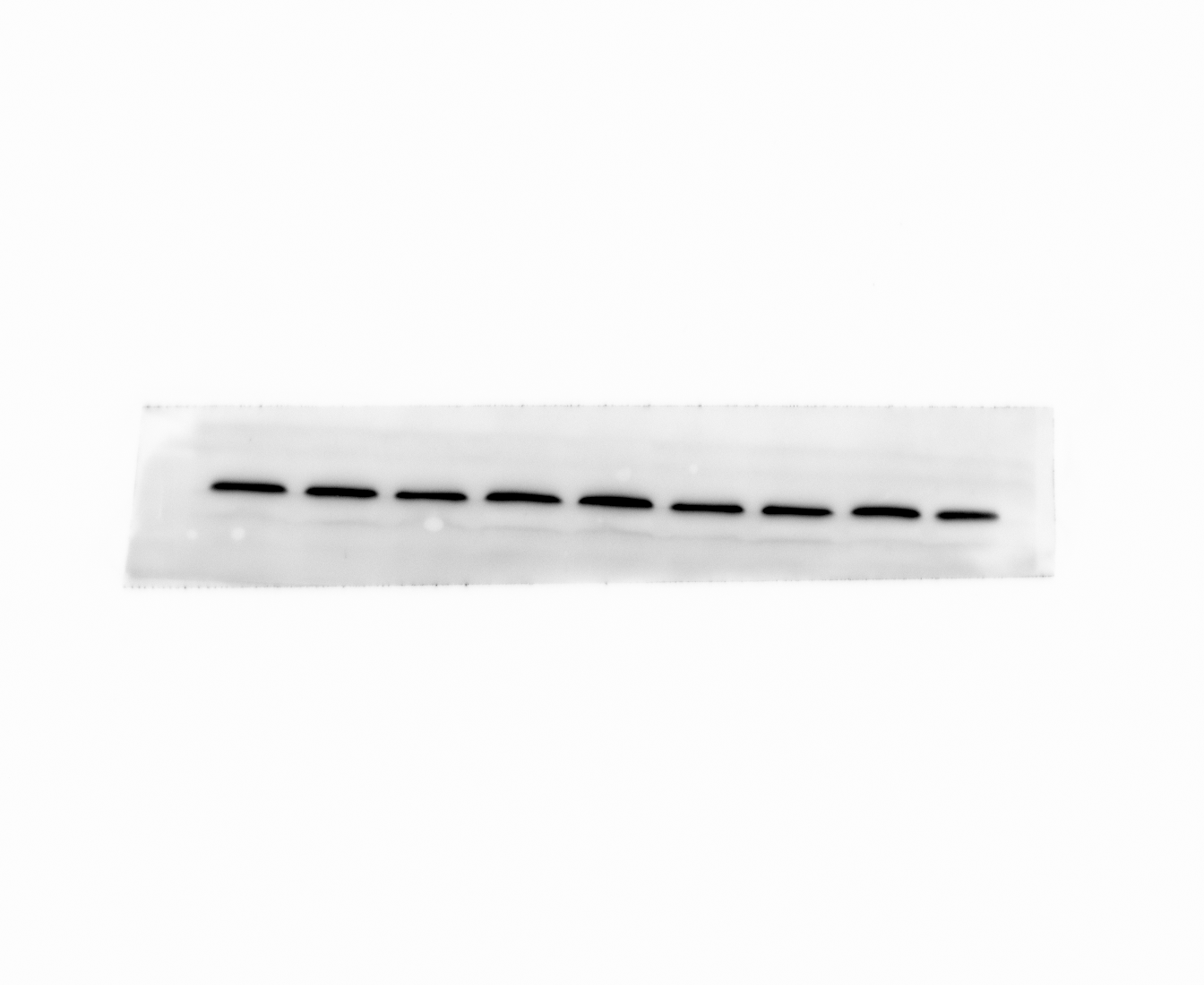

Supplement: Supplementary file 129 — Additional file 129. [file 13020_2026_1383_MOESM129_ESM.tif]

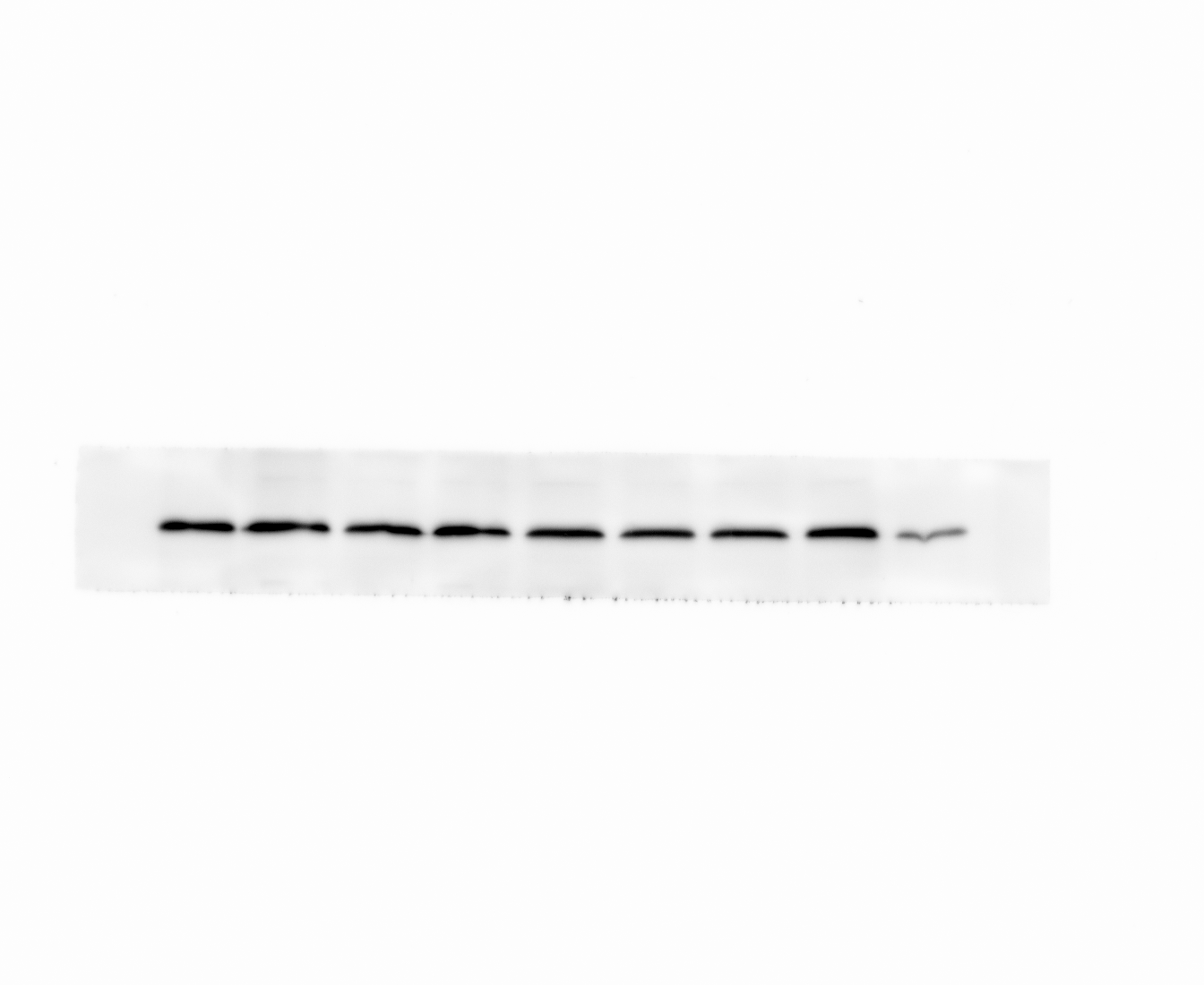

Supplement: Supplementary file 130 — Additional file 130. [file 13020_2026_1383_MOESM130_ESM.tif]

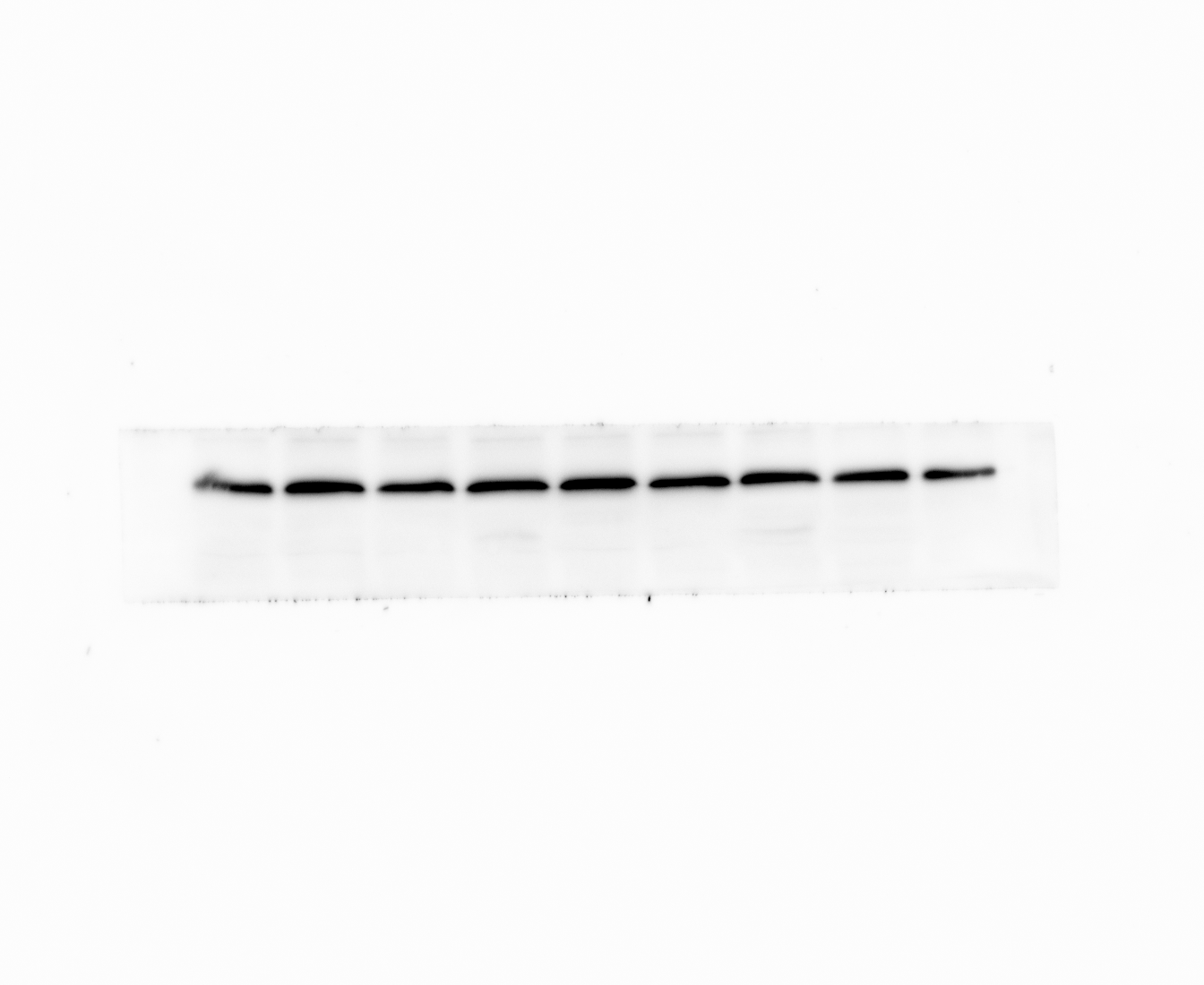

Supplement: Supplementary file 131 — Additional file 131. [file 13020_2026_1383_MOESM131_ESM.tif]

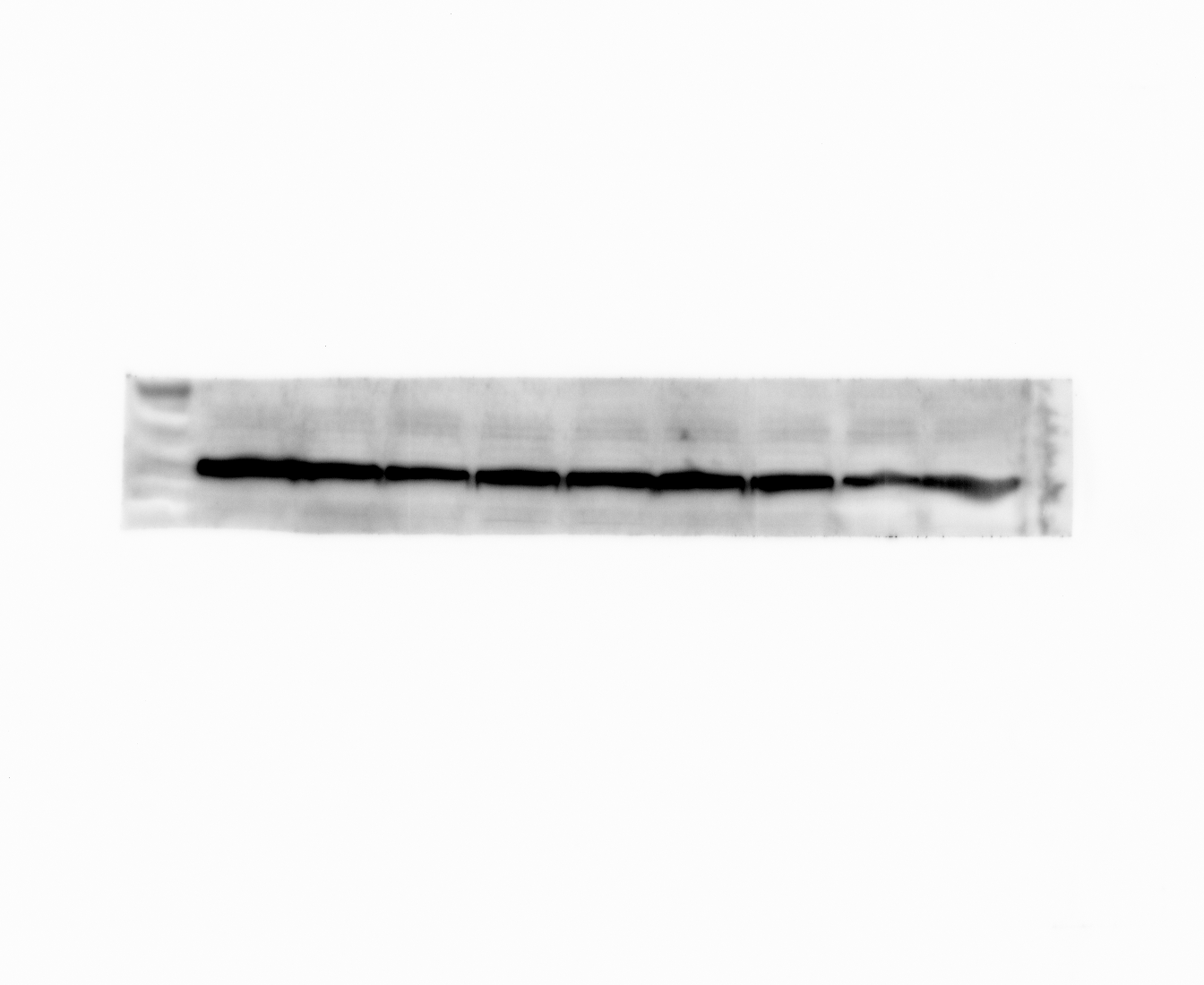

Supplement: Supplementary file 132 — Additional file 132. [file 13020_2026_1383_MOESM132_ESM.tif]

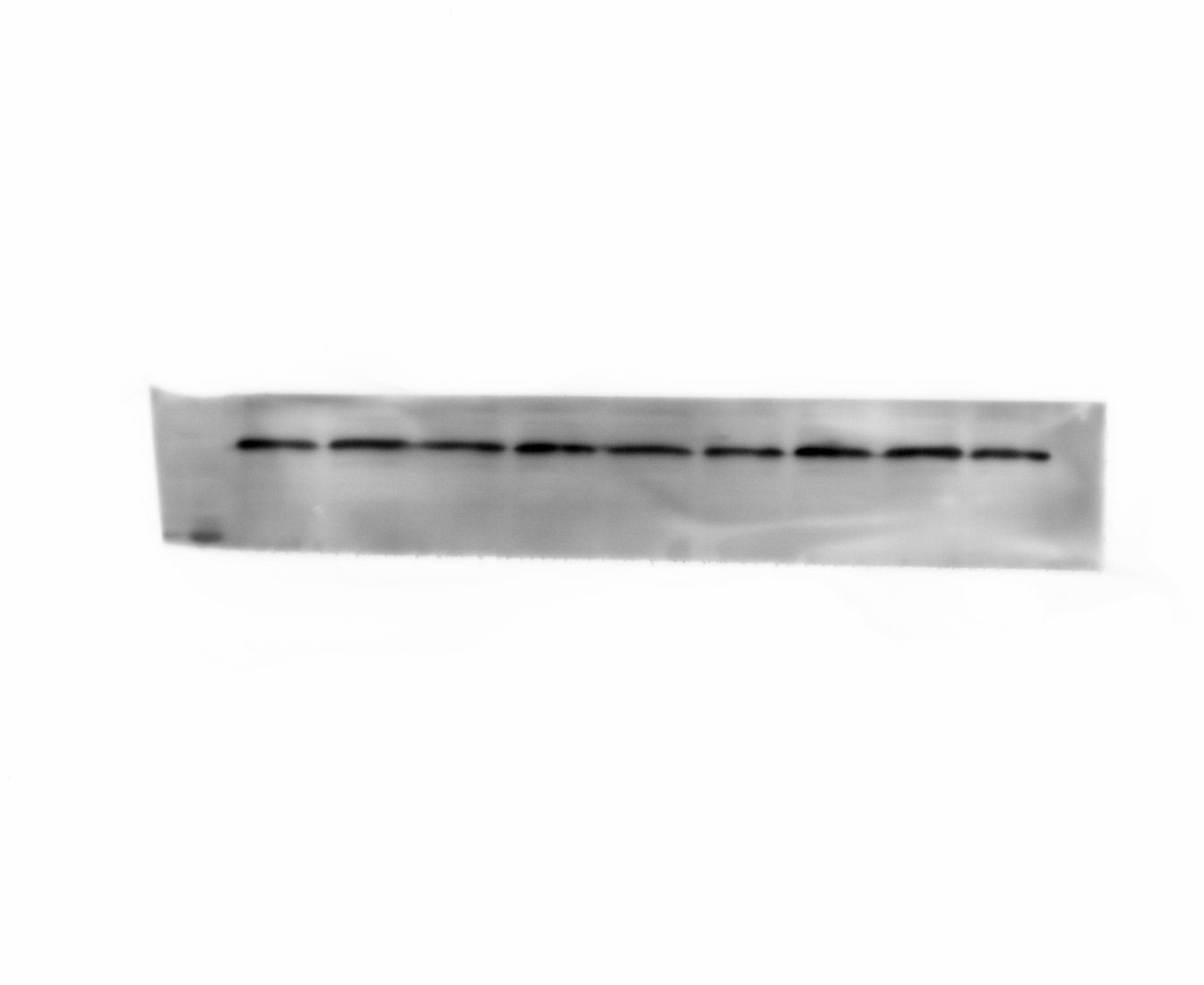

Supplement: Supplementary file 133 — Additional file 133. [file 13020_2026_1383_MOESM133_ESM.tif]

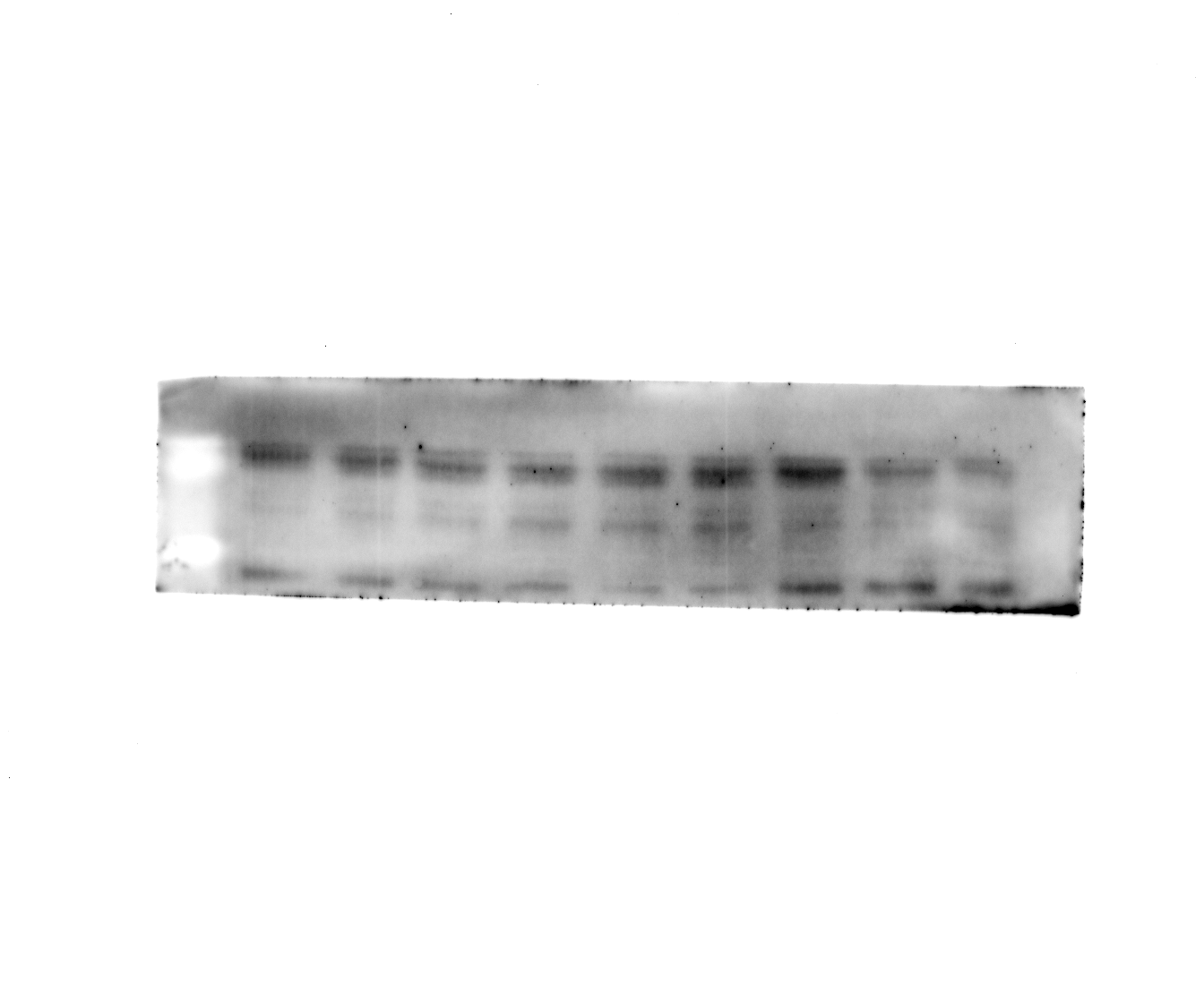

Supplement: Supplementary file 134 — Additional file 134. [file 13020_2026_1383_MOESM134_ESM.tif]
